# Ring Expansion of Alkylidenecarbenes Derived from Lactams, Lactones, and Thiolactones into Strained Heterocyclic Alkynes: A Theoretical Study

**DOI:** 10.3390/molecules24030593

**Published:** 2019-02-07

**Authors:** Nguyen Nhat Thu Le, Josefine Just, Jonathan M. Pankauski, Paul R. Rablen, Dasan M. Thamattoor

**Affiliations:** 1 Department of Chemistry, Colby College, Waterville, ME 04901, USA; nnle@colby.edu (N.N.T.L.); jjust@colby.edu (J.J.); jmpank21@colby.edu (J.M.P.); 2 Department of Chemistry and Biochemistry, Swarthmore College, 500 College Avenue, Swarthmore, PA 19081, USA

**Keywords:** alkylidenecarbenes, rearrangements, strained cycloalkynes, calculations

## Abstract

Strained cycloalkynes are of considerable interest to theoreticians and experimentalists, and possess much synthetic value as well. Herein, a series of cyclic alkylidenecarbenes—formally obtained by replacing the carbonyl oxygen of four-, five-, and six-membered lactams, lactones, and thiolactones with a divalent carbon—were modeled at the CCSD(T)/cc-pVTZ//B3LYP/6-311+G** and CCSD(T)/cc-pVTZ//CCSD/6-311+G** levels of theory. The singlet carbenes were found to be more stable than the triplets. The strained heterocyclic alkynes formed by ring expansion of these singlet carbenes were also modeled. Interestingly, the C≡C bonds in the five-membered heterocycles, obtained from the rearrangement of β-lactam- and β-lactone-derived alkylidenecarbenes, displayed lengths intermediate between formal double and triple bonds. Furthermore, 2-(1-azacyclobutylidene)carbene was found to be nearly isoenergetic with its ring-expanded isomer, and 1-oxacyclopent-2-yne was notably higher in energy than its precursor carbene. In all other cases, the cycloalkynes were lower in energy than the corresponding carbenes. The transition states for ring-expansion were always lower for the 1,2-carbon shifts than for 1,2-nitrogen or oxygen shifts, but higher than for the 1,2-sulfur shifts. These predictions should be verifiable using carbenes bearing appropriate isotopic labels. Computed vibrational spectra for the carbenes, and their ring-expanded isomers, are presented and could be of value to matrix isolation experiments.

## 1. Introduction

**Scheme 1:**
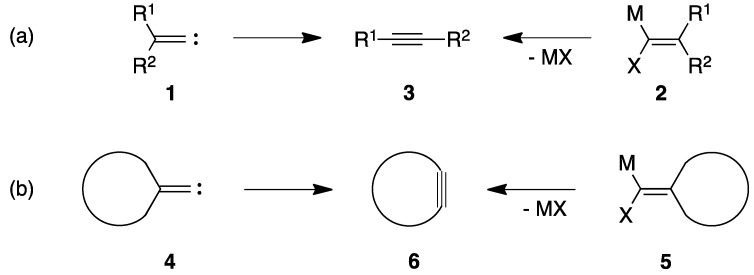
(**a**) The ring expansion of alkylidene carbenes and carbenoids to linear alkynes. R^1^ and R^2^ may be the same or different. M is a metal and X a leaving group. (**b**) An analogous approach to the generation of cycloalkynes from cyclic alkylidenecarbenes and carbenoids.

**Scheme 2:**
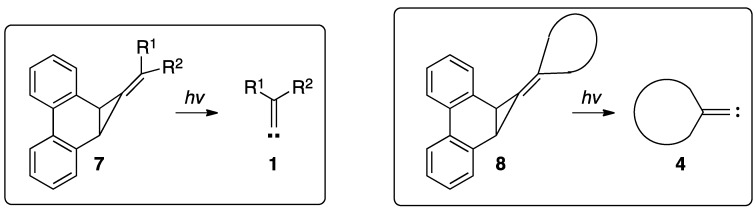
Phenanthrene-based methylenecyclopropanes, exemplified by **7** and **8**, can be used as photochemical sources of acyclic and cyclic alkylidenecarbenes respectively.

Alkylidenecarbenes (**1**), and surrogate carbenoids (**2**), often undergo 1,2-shifts to furnish alkynes (**3**) by a process commonly referred to as the Fritsch-Buttenberg-Wiechell (FBW) rearrangement (Scheme 1a) [1,2,3,4,5,6]. By extending this method to cyclic alkylidenecarbenes (**4**) and carbenoids (**5**), in which the formally sp^2^-hybridized carbon connected to the carbene center is part of a ring, strained cycloalkynes (**6**) have also been generated (Scheme 1b) [4,7,8,9]. In recent years, our laboratory has reported that cyclopropanated phenanthrene systems such as **7** [10,11,12,13] and **8 [14,15]** can serve as photochemical precursors to **1** and **4** respectively (Scheme 2), and thus provide light-induced routes to the corresponding linear and cyclic alkynes.

**Scheme 3:**
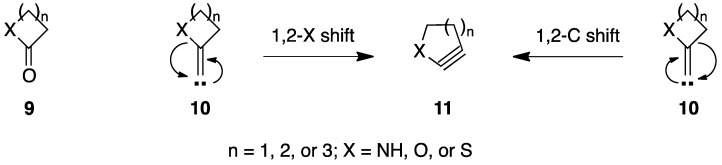
The ring expansion of alkylidenecarbenes (**10**), derived from lactams, lactones, and thiolactones (**9**), into strained heterocyclic compounds (**11**).

Strained cycloalkynes are no longer esoteric chemical curiosities of interest primarily to physical organic chemists and theoreticians [16,17,18]. In recent years, they have emerged as useful intermediates of substantial synthetic value [19,20,21,22,23,24,25,26]. Medium-sized, moderately strained carbocyclic and heterocyclic alkynes have proven to be of particular importance in the field of bioorthogonal chemistry [27,28,29,30,31,32,33,34,35,36,37,38,39]. Larger carbocyclic and oxygen incorporated enediynes have also been prepared, and their Bergman cyclization activity investigated for potential therapeutic use [40,41]. Herein, we describe our computational investigations into the chemistry of cyclic alkylidenecarbenes (**10**) derived by formally replacing the carbonyl oxygen of lactams, lactones, and thiolactones (**9**) with a divalent carbon (Scheme 3). The singlet-triplet energy gaps in these carbenes, and their ability to undergo ring expansion to the corresponding strained heterocyclic alkynes (**11**) have been investigated using density functional theory (B3LYP) [42,43,44] and coupled-cluster (CC) [45,46,47] methods. As the ring expansion could occur by a 1,2-shift of either the heteroatom or carbon, transition states for both pathways were computed and compared. Experiments with appropriate isotope labeling should be able to verify the predictions made below for the preferred rearrangement pathways. Vibrational spectra of the carbenes and the corresponding ring-expanded heterocyclic alkynes, which may be of potential value to matrix isolation experiments [48,49,50], are also presented.

## 2. Results and Discussion

### 2.1. Alkylidenecarbenes Derived from β-Lactam, β-Lactone, and β-Thiolactone

**Scheme 4:**
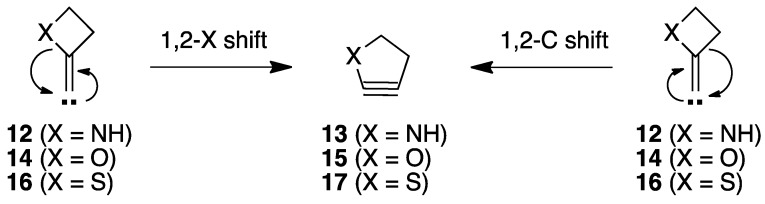
Two pathways for the ring expansion of singlet alkylidenecarbenes derived from β-lactam, β-lactone, and β-thiolactone.

The singlet-triplet energy gap (ΔE_S-T_) in the alkylidenecarbenes derived from *β*-lactam, *β*-lactone, and β-thiolactone were computed at the CCSD(T)/cc-pVTZ//B3LYP/6-311+G** and CCSD(T)/cc-pVTZ//CCSD/6-311+G** levels of theory. In all calculations, the singlets were found to be significantly more stable than the triplets. This gap is consistent with the known preference of alkylidenecarbenes to adopt the singlet state, in which the lone pair can be nested in an approximately sp-hybridized orbital of the divalent carbon [6]. The PES for ring expansion of the singlet carbenes into the corresponding 1-X-cyclopent-2-ynes, which could occur by a 1,2-shift of either the heteroatom X or carbon (Scheme 4), were also modeled. Results are discussed below.

#### 2.1.1. Ring Expansion of 2-(1-Azacyclobutylidene)carbene (**12**) into 1-Azacyclopent-2-yne (**13**)

**Figure 1:**
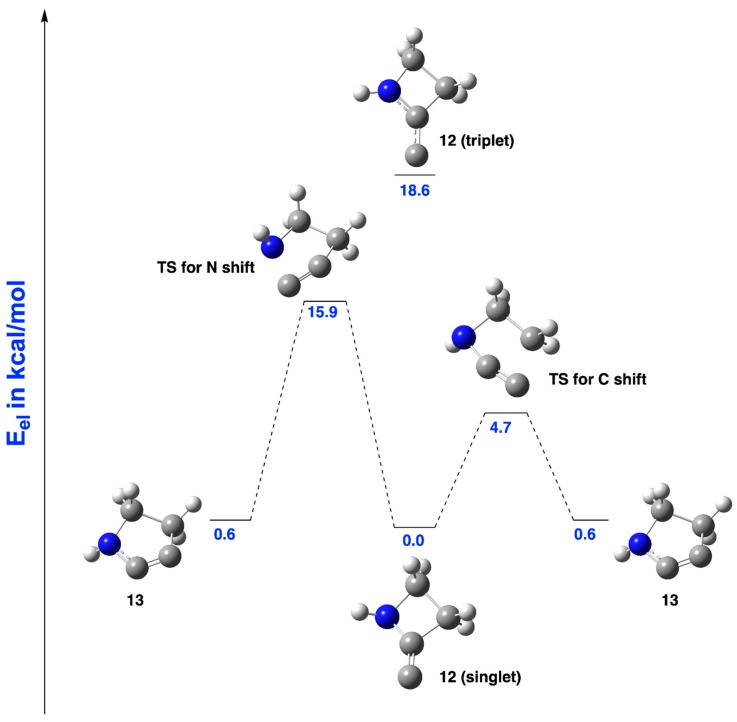
CCSD(T)/cc-pVTZ//CCSD/6-311+G** structures and energies of singlet and triplet **12**, and the PES for the conversion of singlet **12** into 1-azacyclopent-2-yne (**13**) by two different pathways.

The PES for ring expansion of singlet **12** into 1-aza-cyclopent-2-yne (**13**), which could occur by a 1,2-shift of either the nitrogen or carbon (Scheme 4), is depicted in Figure 1 using CCSD(T)/cc-pVTZ//CCSD/6-311+G** energies and structures. A similar diagram, based on CCSD(T)/cc-pVTZ//B3LYP/6-311+G** calculations, is provided in the Supplementary Materials (Figure S1).

**Figure 2:**
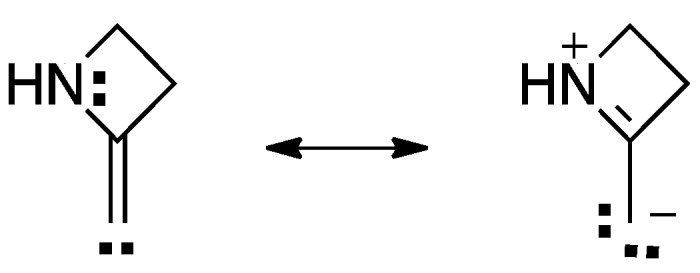
Resonance contributions in **12** showing a C=N bond in the zwitterionic form.

As seen in Figure 1, singlet **12** is virtually isoenergetic with **13** and lies 18.6 kcal/mol below the triplet. Another distinctive feature of **13** is that its triple bond is significantly elongated with a calculated length of 1.28 Å. This value is intermediate between the length of a C=C bond in cyclopentene (~1.32 Å) [51] and our calculated value for the C≡C bond (1.22 Å) in cyclopentyne [15]. Thus, **13** appears to display a partial triple bond, rather than a formal one, in order to alleviate ring strain. Furthermore, the conversion of singlet **12** into **13** appears to favor a 1,2-carbon shift, which has a significantly lower barrier than for a 1,2-nitrogen shift. This preference could stem from at least two factors. One is the “bystander effect” [52] of nitrogen that accelerates the 1,2-migration of carbon. In other words, as the carbon begins to shift, a decrease in electron density at the migration origin in the transition state, as evident from a Natural Population Analysis (NPA) [53,54] (see Supplementary Materials), could be stabilized by electron donation from the nitrogen. A second possible explanation is that the resonance contributions in **12** imparts a partial double bond character to the N-C (sp^2^) bond (Figure 2) which would thus become even more resistant to cleavage. Importantly, such conjugation can be maintained during the 1,2-carbon shift.

**Figure 3:**
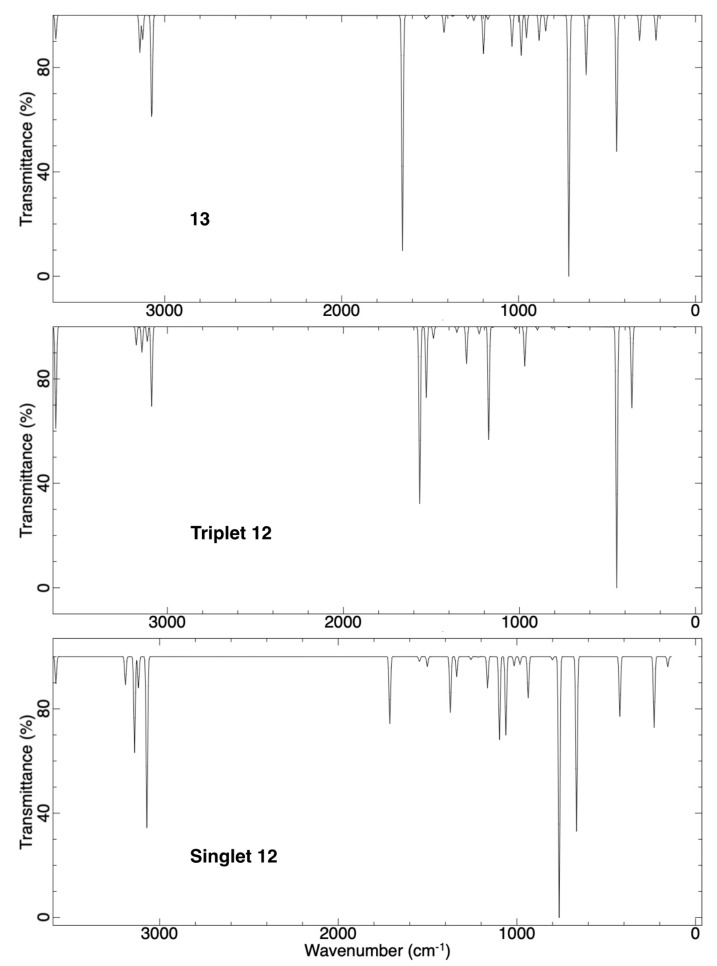
Vibrational spectra computed at CCSD/6-311+G** for singlet **12** (bottom), triplet **12** (middle), and **13** (top).

Vibrational spectra of **12** and **13** computed at CCSD/6-311+G** are shown in Figure 3. The C=C stretch in singlet **12** appears as a weak absorbance at 1711 cm^−1^. The two prominent bands at 763 cm^−1^ and 665 cm^−1^ are associated with the wagging motion of the NH bond coupled to ring oscillations. Triplet **12**, on the other hand, shows a much stronger C=C stretch at 1566 cm^−1^, and another strong absorbance at 447 cm^−1^ corresponding to the out of-plane wagging motions of the N-H bond and the methylene groups. The wagging of these two moieties in the plane of the ring is observed at 1175 cm^−1^. The spectrum of **13** shows a strong absorption at 1656 cm^−1^ associated with the stretching of the NC≡C moiety. The wagging vibrations of the NH and methylene groups, in a direction perpendicular to the plane of the ring also show a strong absorbance at 716 cm^−1^.

#### 2.1.2. Ring Expansion of 2-(1-Oxacyclobutylidene)carbene (**14**) into 1-Oxacyclopent-2-yne (**15**)

**Figure 4:**
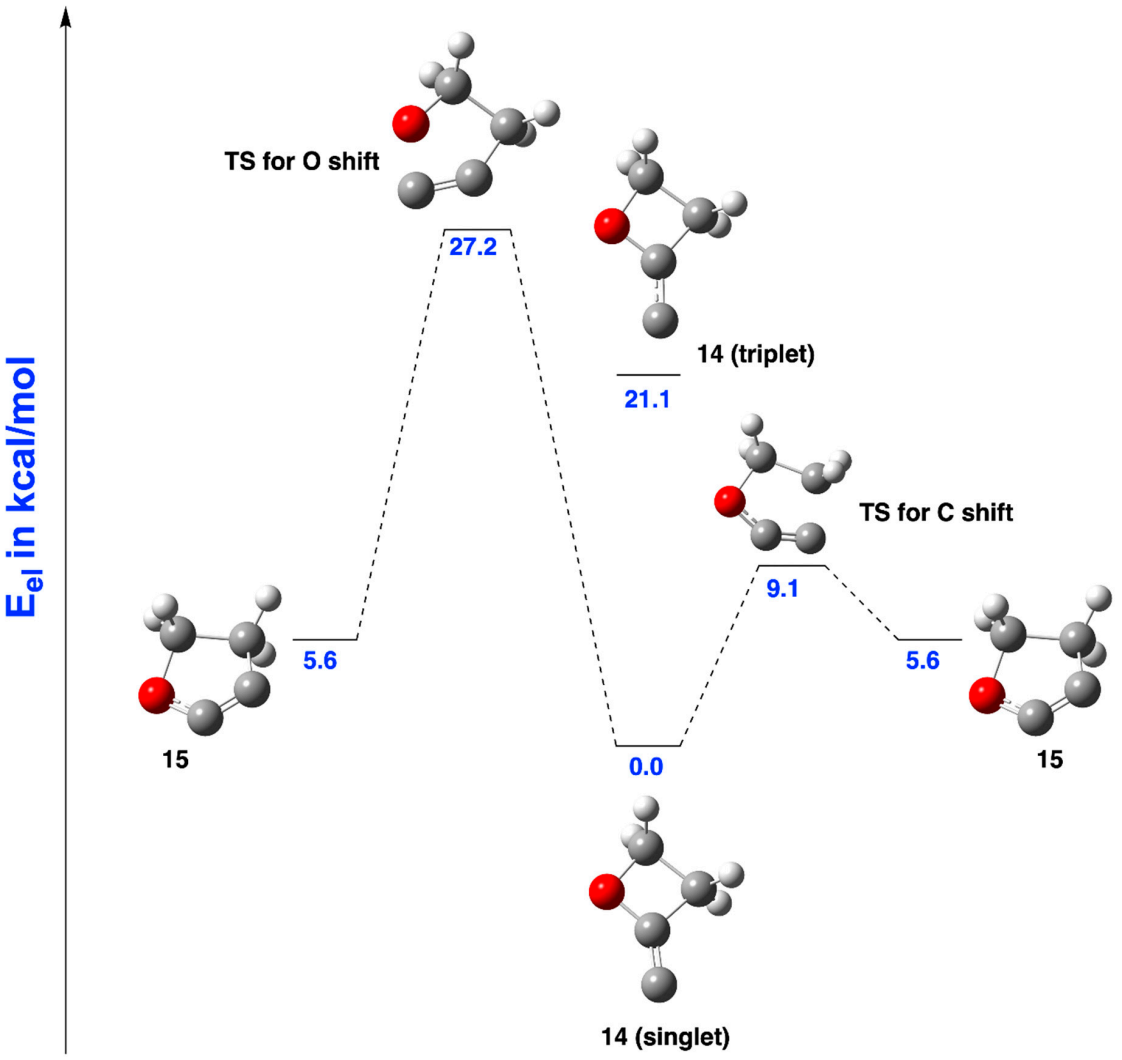
CCSD(T)/cc-pVTZ//CCSD/6-311+G** structures and energies of singlet and triplet **13**, and the PES for the conversion of singlet **14** into 1-oxacyclopent-2-yne (**15**) by two different pathways.

Singlet **14** was found to lie 21.1 kcal/mol below the triplet according to CCSD(T)/cc-pVTZ//B3LYP/6-311+G** calculations (Supplementary Materials, Figure S2). The cycloalkyne **15**, however, was found to be not a minimum at the DFT level of theory, instead reverting without barrier to **14**. On the other hand, singlet and triplet **14**, as well as **15**, were all found to be minima on the CCSD/6-311+G** PES. Their structures and CCSD(T)/cc-pVTZ//CCSD/6-311+G** energies, along with the transition states for converting singlet **14** into **15** are depicted in Figure 4. Notably, **15** was found to be 5.6 kcal/mol higher in energy than singlet **14**, and features a partial triple bond (~1.26 Å) much like 12. Similar to observations with the **12** to **13** rearrangement, the 1,2-carbon shift in singlet **14** is considerably more facile than the 1,2-oxygen shift. Thus, the reactivity of **14** appears to mirror that of **12**, for the same reasons as those discussed above.

**Figure 5:**
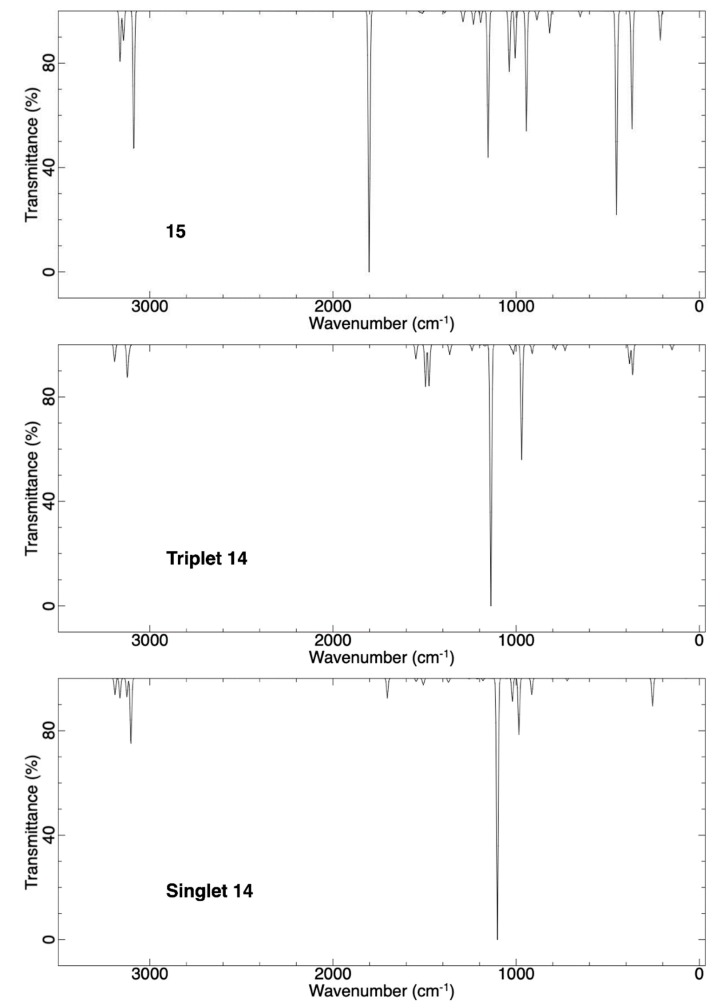
Vibrational spectra computed at CCSD/6-311+G** for singlet **14** (bottom), triplet **14** (middle), and **15** (top).

Vibrational spectra of **14** and **15** computed at CCSD/6-311+G** are shown in Figure 5. In the spectrum of singlet **14**, an especially strong absorbance was found at 1102 cm^−1^, which corresponds to the in-plane, side-to-side motion of the endocyclic sp^2^-hybridized carbon. A similar motion was also observed for triplet **14**, with a strong absorbance at 1138 cm^−1^. The spectrum of triplet **14** also displays another moderately strong absorbance at 970 cm^−1^ corresponding to the in-plane oscillation of the oxygen. Conspicuously, only very weak absorbances were found for C=C stretch in both singlet and triplet **14**. The spectrum of **15** shows strong bands at 1803 cm^−1^ and 453 cm^−1^ associated with vibrations of the OC≡C moiety, and a moderate absorbance at 1153 cm^−1^ corresponding to the in-plane stretching of the OC(sp)bond.

#### 2.1.3. Ring Expansion of 2-(1-Thiocyclobutylidene)carbene (**16**) into 1-Thiocyclopent-2-yne (**17**)

**Figure 6:**
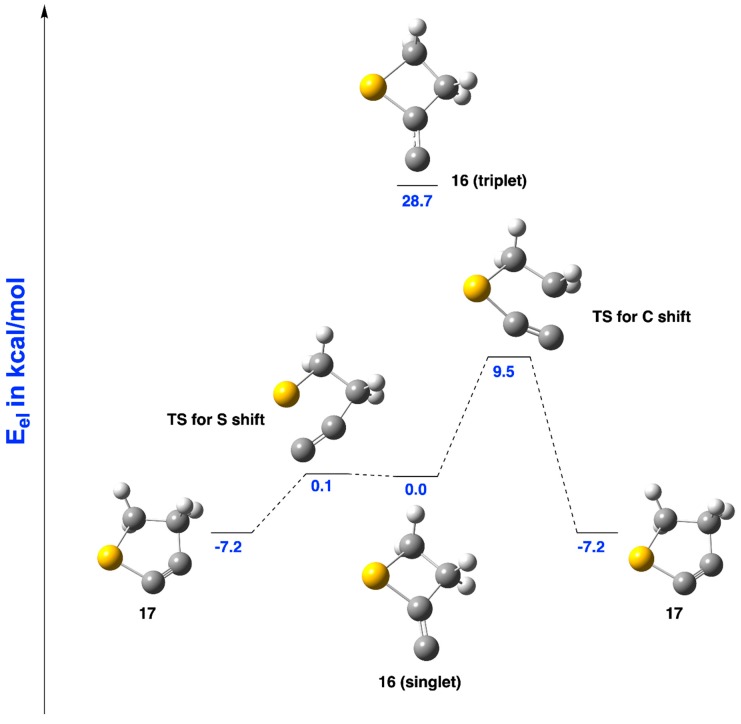
CCSD(T)/cc-pVTZ//CCSD/6-311+G** structures and energies of singlet and triplet **15**, and the PES for the conversion of singlet **16** into 1-thiocyclopent-2-yne (**17**) by two different pathways.

Given the larger size of sulfur, and the attendant incorporation of longer bonds in the ring, one might expect that the thio carbene **16** and 1-thiocyclopent-2-yne (**17**) might behave somewhat differently than the aza and oxa analogs discussed above. These expectations are indeed borne out by CCSD(T)/cc-pVTZ//CCSD/6-311+G** calculations (Figure 6). As with **12** and **14**, singlet **16** is more stable than the triplet (ΔE_S-T_ = −28.7 kcal/mol). The PES for the conversion of singlet **16** into **17**, however, does show some important differences compared to the profiles seen in Figure 1 and Figure 4. For one, the carbon–carbon triple bond **16** is shorter (~1.24 Å) than in either **12** or **14**. Furthermore, **16** is lower in energy than singlet **15** by −7.2 kcal/mol. This could be attributed to lesser ring strain in **16** compared to **12** and **14,** a benefit accorded by the incorporation of longer carbon–sulfur bonds in the ring. A second important difference is that the ring expansion of **16** to **17** prefers to occur by a 1,2-sulfur shift, which is almost barrier-free, compared to a 1,2-carbon shift that needs an activation energy of almost 10 kcal/mol. This behavior may be attributed to the greater nucleophilicity and size of sulfur. The 3p orbital on sulfur is less effective for resonance stabilization due to poor overlap with the 2p orbital on the adjacent carbon. On the other hand, a lone pair on the larger sulfur atom can interact with the empty orbital on the carbenic center to initiate bonding at a longer distance than possible with either nitrogen or oxygen. A similar profile, obtained at the CCSD(T)/cc-pVTZ//B3LYP/6-311+G** level of theory, is provided in the Supplementary Materials (Figure S3).

**Figure 7:**
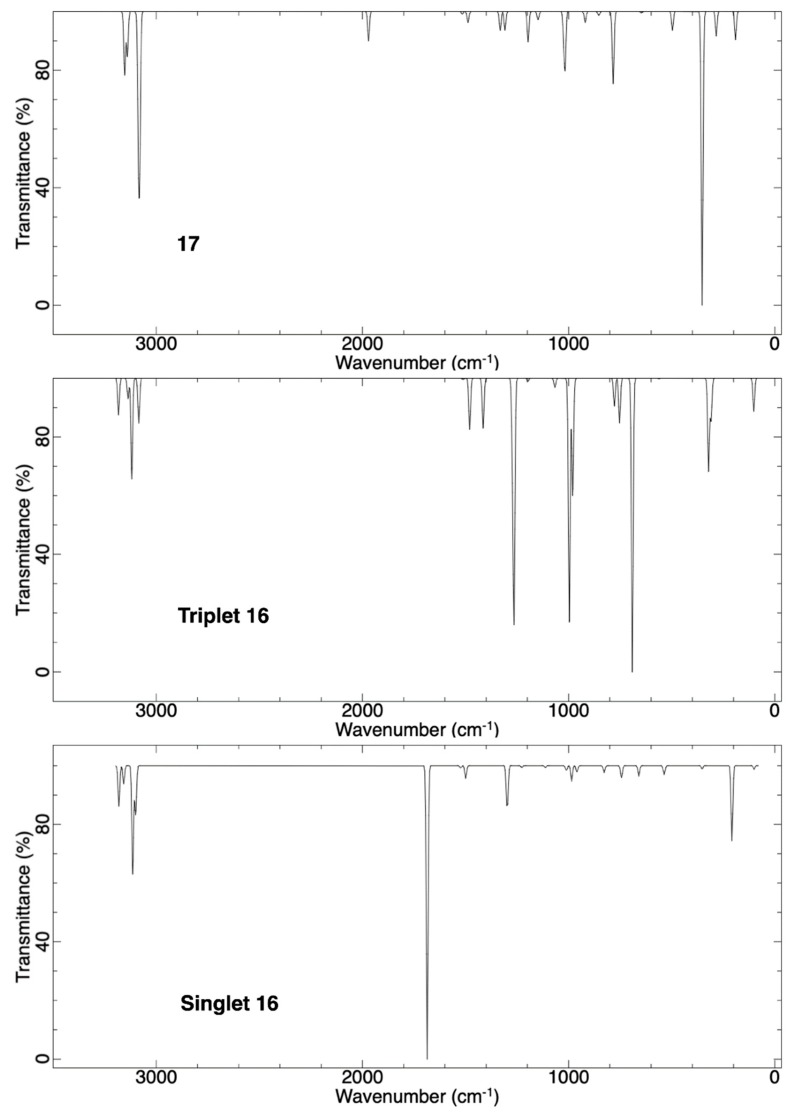
Vibrational spectra computed at CCSD/6-311+G** for singlet **16** (bottom), triplet **16** (middle), and **17** (top).

Vibrational spectra of **16** and **17** computed at CCSD/6-311+G** are shown in Figure 7. The IR spectrum of singlet **16** is relatively clean and shows a strong absorbance at 1686 cm^−1^ for the C=C stretch. The spectrum of triplet **16** shows a moderately strong absorbance at 1265 cm^−1^ for the wagging motions of the two methylene groups in the ring. The stretching vibrations of the bond between O and the endocyclic sp^2^ carbon have an absorbance at 691 cm^−1^, and another absorbance at 997 cm^−1^ is seen for the stretching vibrations of the bond between the CH_2_ group and the double bond. The alkynyl group in **17** shows two distinct bands in the IR spectrum, a weak absorbance at 1970 cm^−1^ for its stretching vibrations and a strong absorbance at 353 cm^−1^ for its wagging motions in the plane of the ring. Absorbances due to C-H stretches are seen 3080 cm^−1^, and the stretching motion of the bond between O and the alkynyl group absorbs at 784 cm^−1^.

### 2.2. Alkylidenecarbenes Derived from γ-Lactam, γ-Lactone, and γ-Thiolactone

**Scheme 5:**
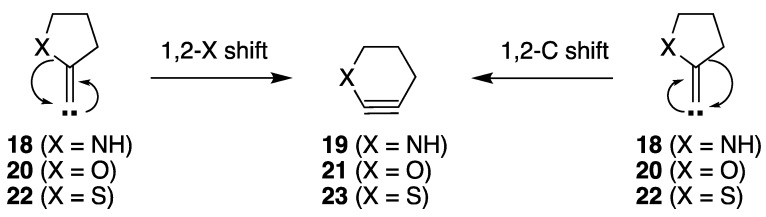
Ring expansion of singlet alkylidenecarbenes, derived from γ-lactam, γ-lactone, and γ-thiolactone, by a 1,2 shift of either X or carbon.

The ΔE_S-T_ gaps of the the alkylidenecarbenes in this series also favor the singlets at the CCSD(T)/cc-pVTZ//B3LYP/6-311+G** and CCSD(T)/cc-pVTZ//CCSD/6-311+G** levels of theory. Structures and energies of these carbenes, and the PES for conversion of the singlet carbenes into corresponding 1-X-cyclohex-2-ynes, by a 1,2-shift of X or carbon (Scheme 5), are described below.

#### 2.2.1. Ring Expansion of 2-(1-Azacyclopentylidene)carbene (**18**) into 1-Azacyclohex-2-yne (**19**)

**Figure 8:**
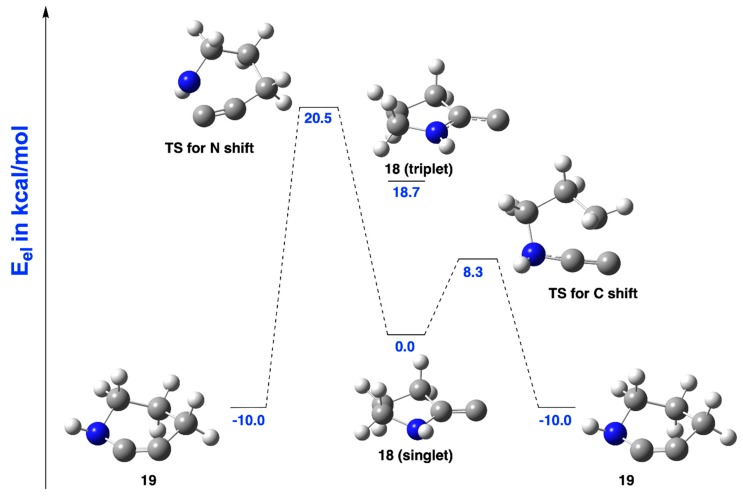
CCSD(T)/cc-pVTZ//CCSD/6-311+G** structures and energies of singlet and triplet **18**, and the PES for the conversion of singlet **18** into 1-azacyclohex-2-yne (**19**) by two different pathways.

As depicted in Figure 8, CCSD(T)/cc-pVTZ//CCSD/6-311+G** calculations show that singlet **18** is about 18.7 kcal/mol lower in energy than the triplet. Furthermore, in sharp contrast to what has been described above with **12** and **13**, the cyclic alkyne **19** is lower in energy than singlet **18** by 10.0 kcal/mol. This result is understandable given that **19**, which features a six-membered ring, is much less strained than the five-membered ring in **13**. The conversion of singlet **18** into **19** preferentially occurs by a 1,2-carbon shift, which needs to overcome a barrier of 8.3 kcal/mol. A 1,2-nitrogen shift, on the other hand, has a much higher activation energy of 20.5 kcal/mol. A similar profile, obtained by CCSD(T)/cc-pVTZ//B3LYP/6-311+G** calculations, is provided in the (Figure S4).

**Figure 9:**
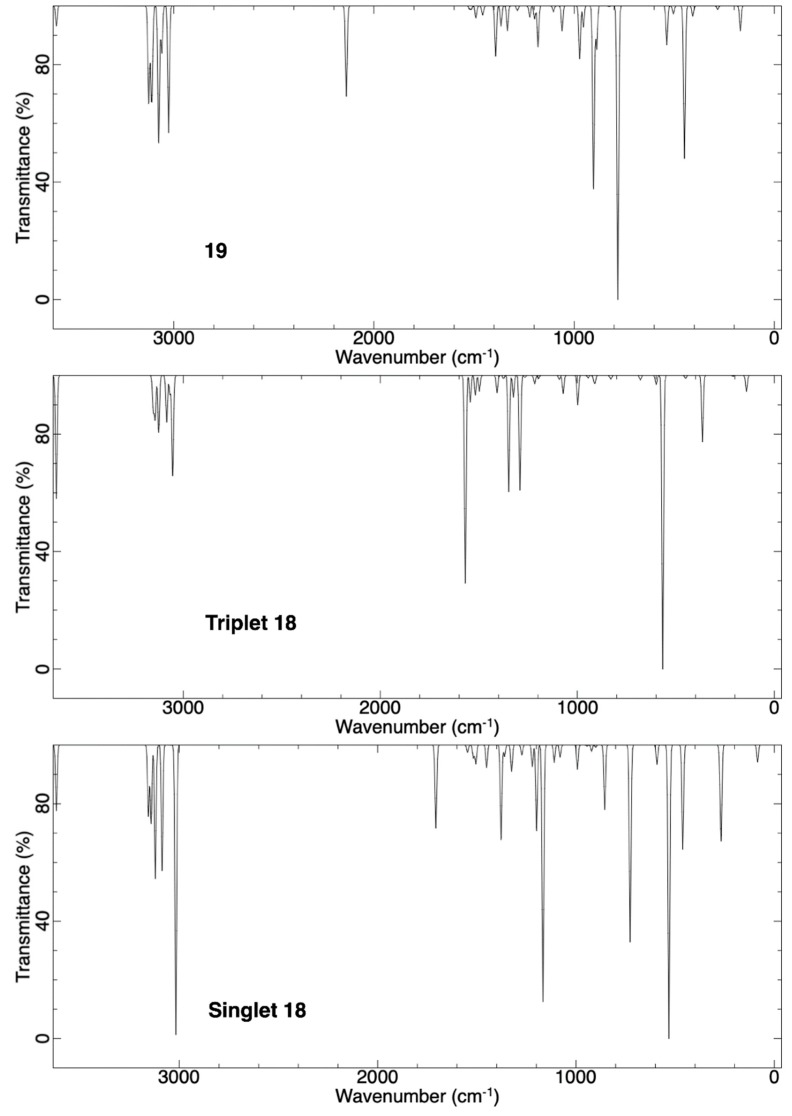
Vibrational spectra computed at CCSD/6-311+G** for singlet **18** (bottom), triplet **18** (middle), and **19** (top).

CCSD/6-311+G** calculated vibrational spectra of singlet and triplet **18**, as well as **19**, are shown in Figure 9. The spectrum of singlet **18** shows strong absorbances at 1167 cm^−1^, for the in-plane motion of the endocyclic sp^2^ carbon, and 531 cm^−1^ for the out of plane N-H bend. The C=C stretch in singlet **18** appears at a frequency of 1706 cm^−1^. Triplet **18** shows a band at 1568 cm^−1^, for the vibrations of the C=C-N moiety, and another strong absorbance at 566 cm^−1^ for the out-of-plane wagging of the N-H bond. The spectrum of **19** shows the familiar stretching frequency of the alkynyl group at 2137 cm^−1^ and strong absorbances at 903 cm^−1^ and 782 cm^−1^ for the wagging motions of the N-H bond coupled to the vibrations of the methylene groups.

#### 2.2.2. Ring Expansion of 2-(1-Oxacyclopentylidene)carbene (**20**) into 1-Oxacyclohex-2-yne (**21**)

**Figure 10:**
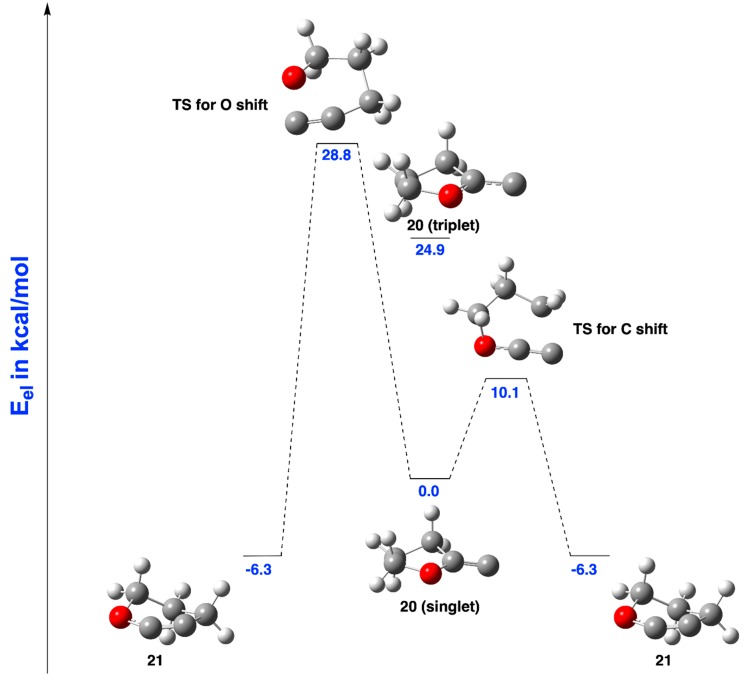
CCSD(T)/cc-pVTZ//CCSD/6-311+G** structures and energies of singlet and triplet **20**, and the PES for the conversion of singlet **20** into 1-oxacyclohex-2-yne (**21**) by two different pathways.

Singlet **20** was found to be 24.9 kcal/mol below the triplet, and 6.3 kcal/mol above cycloalkyne **21**, according to CCSD(T)/cc-pVTZ//CCSD/6-311+G** (Figure 10). These calculations also reveal that the barrier for converting singlet **20** into **21** by a 1,2-carbon shift is 10.1 kcal/mol, whereas the transition state for accomplishing the ring expansion by a 1,2-oxygen shift is much higher at 28.8 kcal/mol. These results are also summarized in Figure 10. Structures and energies from the CCSD(T)/cc-pVTZ//B3LYP/6-311+G** calculations on this system are provided in the Supplementary Materials (Figure S5).

**Figure 11:**
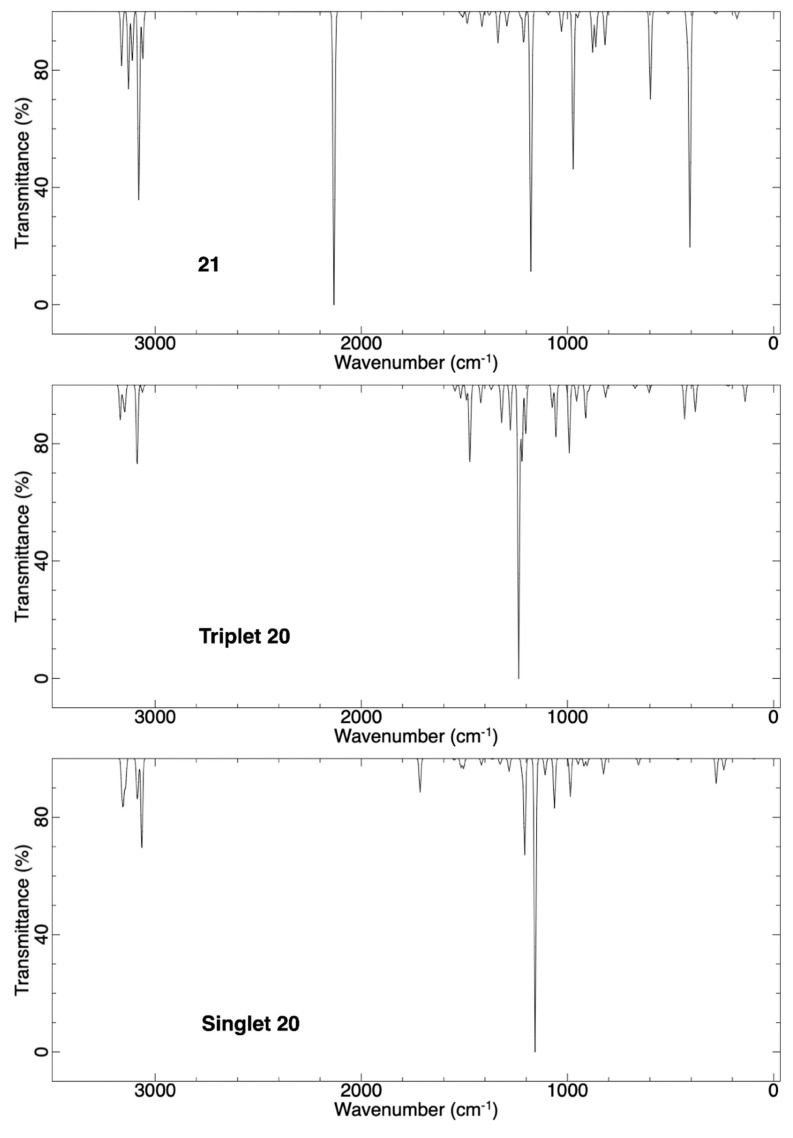
Vibrational spectra computed at CCSD/6-311+G** for singlet **20** (bottom), triplet **20** (middle), and **21** (top).

The computed vibrational spectra (CCSD/6-311+G**) of **20** (singlet and triplet) and **21** are displayed in Figure 11. The strongest absorbances in the spectrum of singlet and triplet **20** appear at 1157 cm^−1^ and 1236 cm^−1^ respectively and correspond to the stretching vibration of the bond between oxygen and the endocyclic sp^2^ carbon. The spectrum of **21** shows a strong absorbance at 2132 cm^−1^ characteristic of the alkynyl group. Stretching vibrations of the bond between oxygen and the alkyne moiety absorb at 1178 cm^−1^, and the in-plane motions of the OC≡C group absorb at 407 cm^−1^.

#### 2.2.3. Ring Expansion of 2-(1-Thiacyclopentylidene)carbene (**22**) into 1-Thiacyclohex-2-yne (**23**)

**Figure 12:**
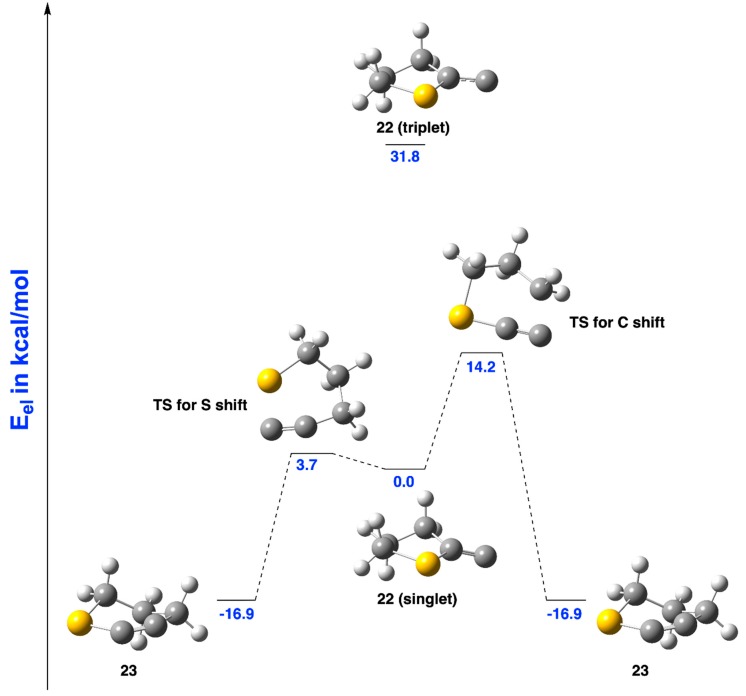
CCSD(T)/cc-pVTZ//CCSD/6-311+G** structures and energies of singlet and triplet **22**, and the PES for the conversion of singlet **22** into 1-thiocyclohex-2-yne (**23**) by two different pathways.

According to CCSD(T)/cc-pVTZ//CCSD/6-311+G** calculations, singlet **22** lies 31.8 kcal/mol below the triplet, and 16.9 kcal/mol above thiocycloakyne **23** (Figure 12). The PES for conversion of singlet **22** into **23** is also shown in Figure 12 at this level of theory. Unlike the aza- and oxa-substituted analogs, and consistent with previous observations in the case of **16**, carbene **22** prefers to form **23** by shifting the heteroatom. The 1,2-sulfur shift, which has a barrier of only 3.7 kcal/mol, requires much less energy than a 1,2-carbon shift (barrier of 14.2 kcal/mol) to form **23**. Similar results were obtained with CCSD(T)/cc-pVTZ//B3LYP/6-311+G** calculations and are provided in the Supplementary Materials (Figure S6).

**Figure 13:**
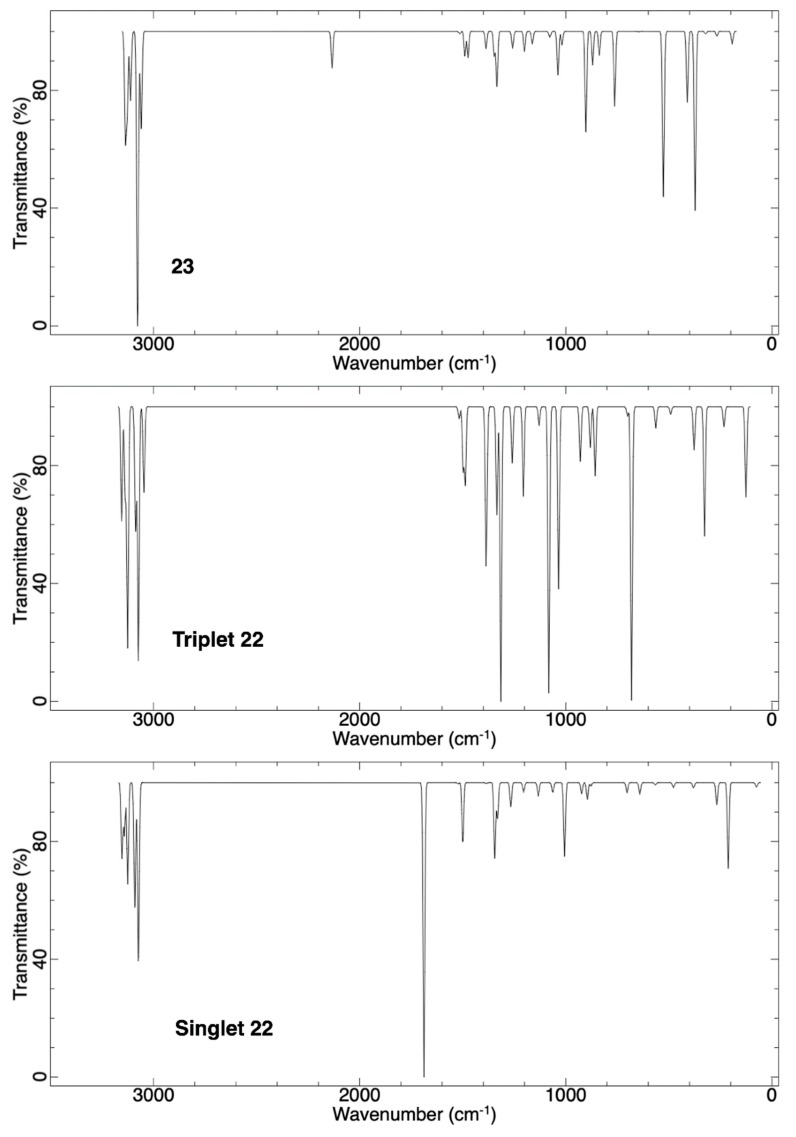
Vibrational spectra computed at CCSD/6-311+G** for singlet **22** (bottom), triplet **22** (middle), and **23** (top).

Vibrational spectra of **22** and **23** computed at CCSD/6-311+G** are shown in Figure 13. Singlet **22** shows a particularly strong absorbance at 1688 cm^−1^ for the C=C stretch. Triplet **22** shows a moderate absorbance at 1389 cm^−1^ due to the C=C stretch, whereas the stretching vibration of the bond between S and the sp^2^ carbon shows somewhat stronger absorbances at 1082 cm^−1^ and 681 cm^−1^. The C≡C stretching frequency for **23** appears at 2134 cm^−1^ but is rather weak compared to the other members of the series, **19** and **21**. This may be attributable to the diminished change in dipole moment, which is associated with that vibrational mode due to the lower electronegativity of sulfur compared to oxygen and nitrogen. The spectrum of **23** also shows moderately strong absorbances at 527 cm^−1^ for the coupled motions of the carbons in the ring, and 373 cm^−1^ for the in-plane vibrations of the S-C≡C group.

### 2.3. Alkylidenecarbenes Derived from δ-Lactam, δ-Lactone, and δ-Thiolactone

**Scheme 6:**
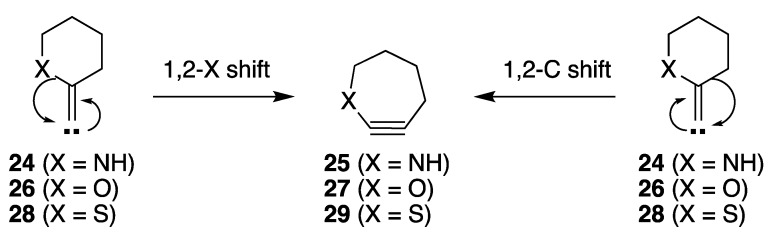
Ring expansion of singlet alkylidenecarbenes, derived from δ-lactam, δ-lactone, and δ-thiolactone, by a 1,2 shift of either X or carbon.

Members in this group are considerably less strained than their counterparts in the β- and γ-series. CCSD(T)/cc-pVTZ//B3LYP/6-311+G** and CCSD(T)/cc-pVTZ//CCSD/6-311+G** calculations reveal that the ΔE_S-T_ gaps of the alkylidenecarbenes in the δ-series are much larger than seen for analogously substituted species in the previous two series, and favor the singlet. The cycloalkynes in this series are also substantially more stable than their corresponding alkylidencarbene isomers. Structures and energies of these carbenes as well the PES for conversion of singlet carbenes into the corresponding 1-X-cyclohept-2-ynes, by a 1,2-shift of X or carbon (Scheme 6), are described below.

#### 2.3.1. Ring Expansion of 2-(1-Azacyclohexylidene)carbene (**24**) into 1-Azacyclohept-2-yne (**25**)

**Figure 14:**
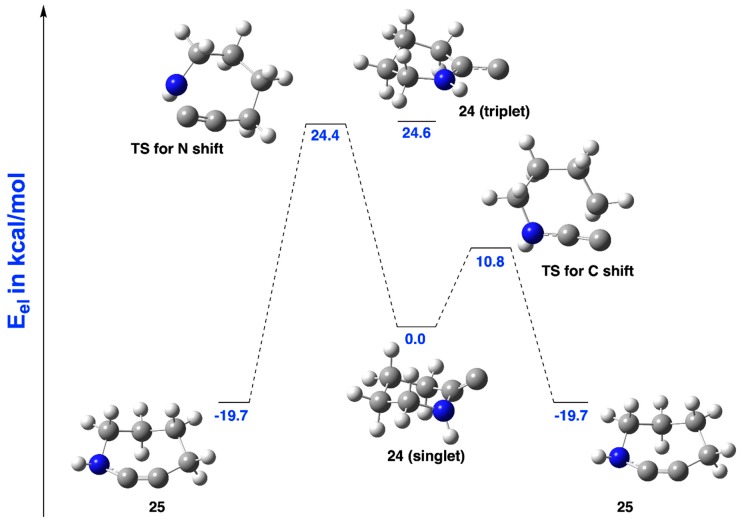
CCSD(T)/cc-pVTZ//CCSD/6-311+G** structures and energies of singlet and triplet **24**, and the PES for the conversion of singlet **24** into 1-azacyclohept-2-yne (**25**) by two different pathways.

As seen in Figure 14, CCSD(T)/cc-pVTZ//CCSD/6-311+G** calculations show that singlet **24** lies 24.6 kcal/mol below the triplet and 19.7 kcal/mol above the azacycloheptyne **25**. Structurally, the ring in singlet **24** adopts the familiar chair conformation but in the triplet, the portion of the ring in the vicinity of the double bond is essentially planar. Another interesting feature in singlet **24** is that the double bond appears to sharply bend toward the nitrogen with the N-C=C angle compressing to 78.5° and the C-C=C angle increasing to 154.5°. This could be rationalized as a stabilizing interaction between the lone pair on nitrogen and the empty p orbital on the carbenic carbon. Somewhat counterintuitively, despite this structural distortion, the formation of **25** by a 1,2-nitrogen shift still has a rather large barrier of 24.4 kcal/mol compared to a much smaller activation energy of 10.8 kcal/mol required for the 1,2-carbon shift. Results obtained with CCSD(T)/cc-pVTZ//B3LYP/6-311+G** calculations are provided in the Supplementary Materials (Figure S7).

**Figure 15:**
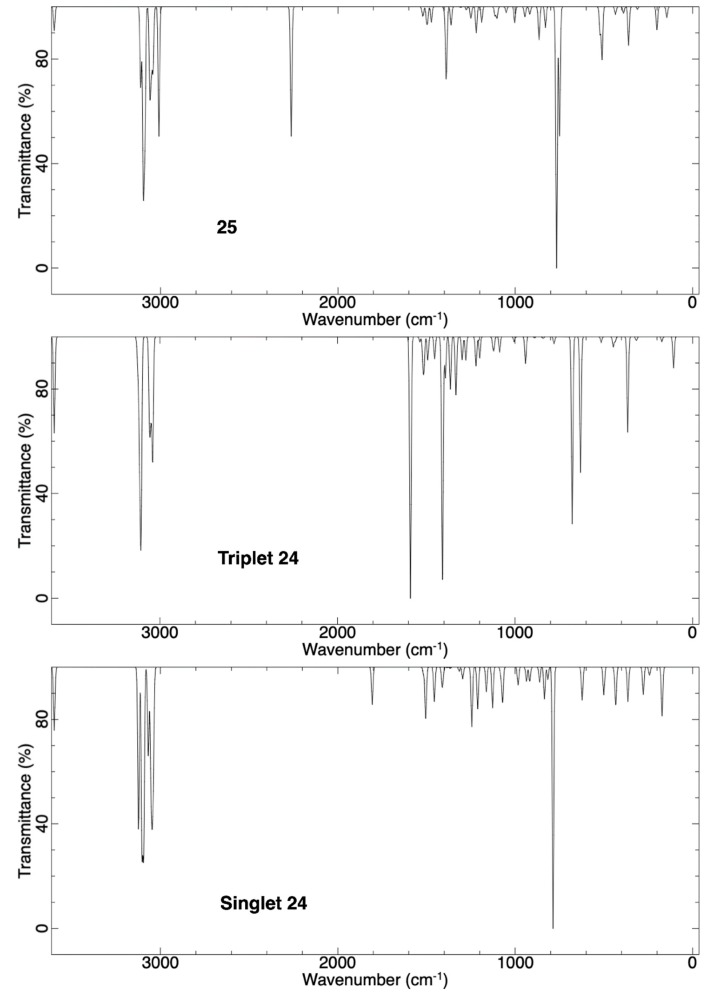
Vibrational spectra computed at CCSD/6-311+G** for singlet **24** (bottom), triplet **24** (middle), and **25** (top).

The vibrational spectra of **24** and **25** computed at CCSD/6-311+G** are shown in Figure 15. Singlet **24** has a strong band at 786 cm^−1^ caused by the out-of-plane wagging motion of the N-H bond. The spectrum of triplet **24** shows a strong band at 1589 cm^−1^ that corresponds to the in-plane vibrations of the C=C-N moiety, and another band at 1409 cm^−1^ that is associated with the coupled motions of atoms in the ring. The characteristic C≡C stretching frequency in **25** appears as a moderately strong band at 2264 cm^−1^. Cycloalkyne **25** also shows a strong absorbance at 767 cm^−1^ and a weaker band at 750 cm^−1^, both of which are associated with the out-of-plane bending motions of the N-H bond.

#### 2.3.2. Ring Expansion of 2-(1-Oxacyclohexylidene)carbene (**26**) into 1-Oxacyclohept-2-yne (**27**)

**Figure 16:**
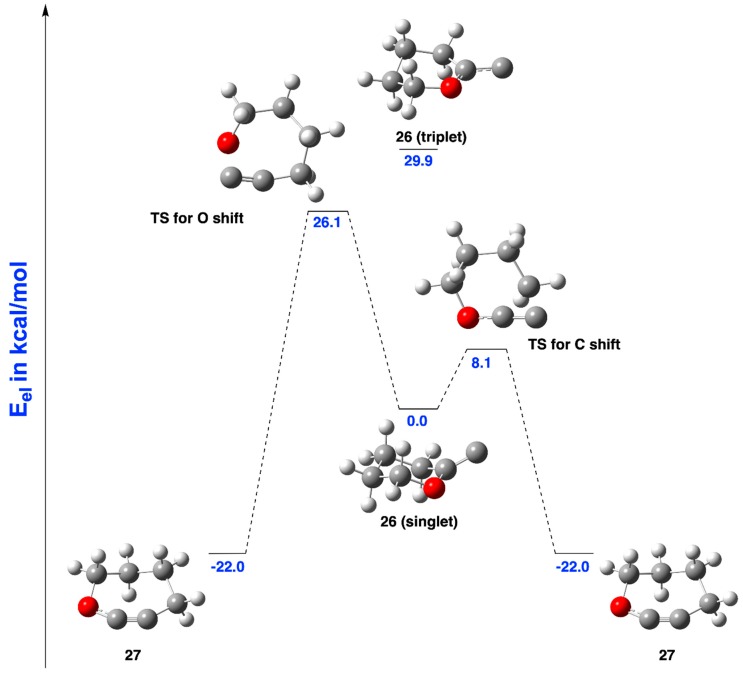
CCSD(T)/cc-pVTZ//CCSD/6-311+G** structures and energies of singlet and triplet **26**, and the PES for the conversion of singlet **26** into 1-oxacyclohept-2-yne (**27**) by two different pathways.

CCSD(T)/cc-pVTZ//CCSD/6-311+G** structures and energies of **26** (singlet and triplet), and the PES for the rearrangement of singlet **26** into **27**, are represented in Figure 16. These calculations show that singlet **26** is lower in energy than the triplet by 29.9 kcal/mol. Similar to what was observed with **24** above, the six-membered ring in singlet **26** adopts a chair conformation whereas the triplet features a somewhat flattened portion of the ring near the double bond. Unlike singlet **24**, however, there is no inclination for the double bond in singlet **26** to ‘lean’ toward the oxygen. The cyclohexyne **27** is 22 kcal/mol below singlet **26**. The transition state for the 1,2-carbon shift to rearrange from singlet **25** into **26** lies 8.1 kcal/mol above the carbene, whereas the barrier to form **27** by a 1,2-oxygen shift is much higher at 26.1 kcal/mol. Structures and energies for this system calculated at CCSD(T)/cc-pVTZ//B3LYP/6-311+G** are provided in the Supplementary Materials (Figure S8).

**Figure 17:**
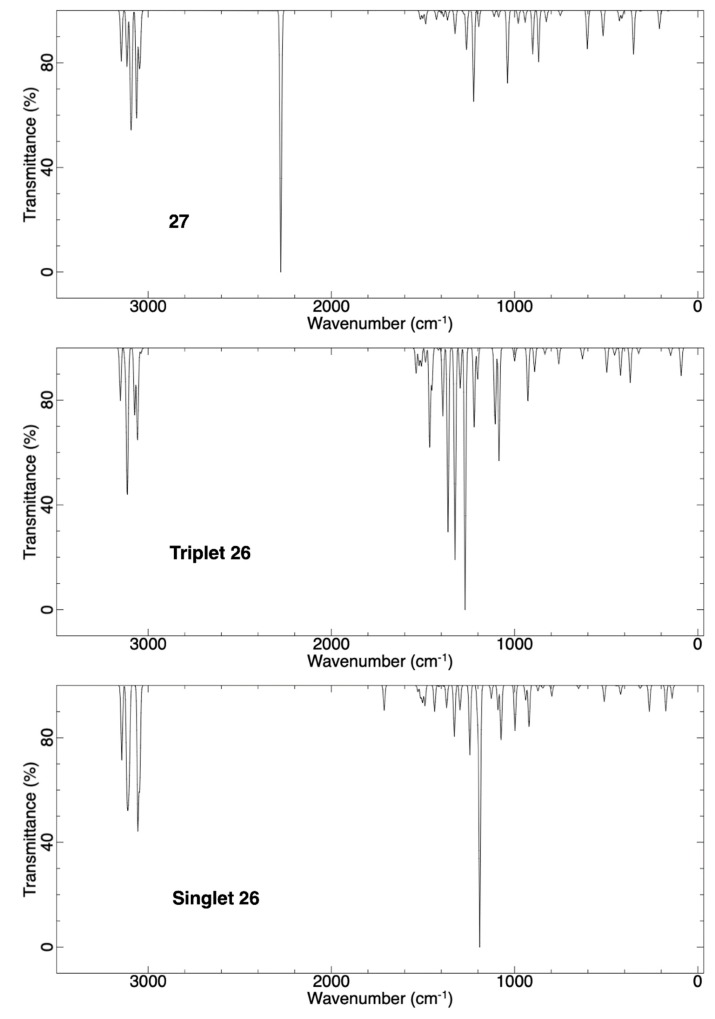
Vibrational spectra computed at CCSD/6-311+G** for singlet **26** (bottom), triplet **26** (middle), and **27** (top).

Vibrational spectra of **26** and **27** computed at CCSD/6-311+G** are shown in Figure 17. Singlet **26** shows a strong band at 1190 cm^−1^ for the stretching vibrations of the bond connecting oxygen with the endocyclic sp^2^ carbon. Triplet **26** displays bands at 1325 cm^−1^ for the wagging motions of the methylene groups and 1270 cm^−1^ for vibrations associated with the O-C=C group. The most distinctive absorbance in the spectrum of cyclohexyne **27** is a strong band at 2276 cm^−1^ corresponding to the alkynyl stretch.

#### 2.3.3. Ring Expansion of 2-(1-Thiocyclohexylidene)carbene (**28**) into 1-Thiocyclohept-2-yne (**29**)

**Figure 18:**
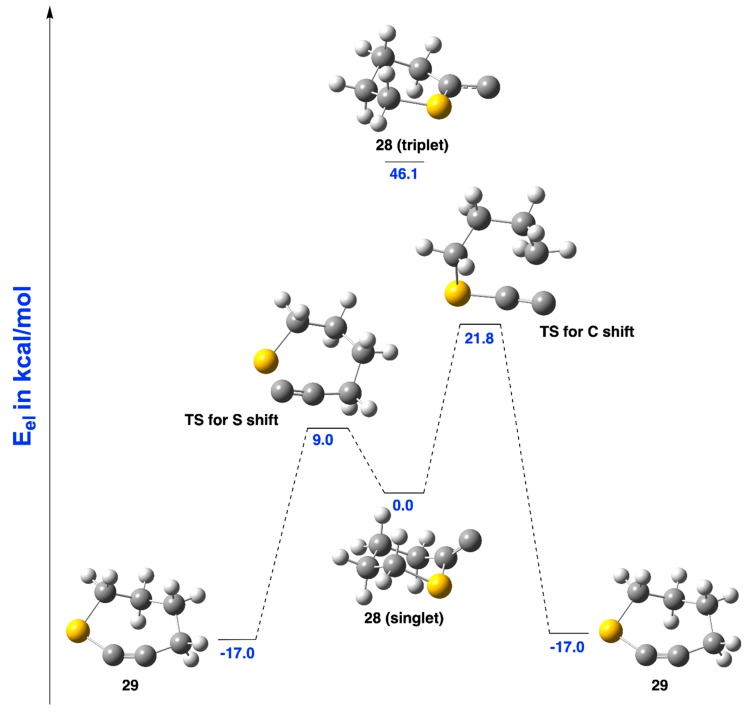
CCSD(T)/cc-pVTZ//CCSD/6-311+G** structures and energies of singlet and triplet **28**, and the PES for the conversion of singlet **28** into 1-thiocyclohept-2-yne (**29**) by two different pathways.

CCSD(T)/cc-pVTZ//CCSD/6-311+G** calculations show that singlet **28** is 46.1 kcal/mol lower in energy than the triplet, and this ΔE_S-T_ gap is the largest observed for all carbene species in this study (Figure 18). Singlet **28** also shows a chair conformation in the six-membered ring while the triplet has a significantly planarized ring around the double bond. The structure of singlet **28** closely resembles that of the aza analog **24** in that the carbenic center is tilted toward the sulfur. This leads to an unusually small S-C=C bond angle of 83.1° whereas the C-C=C angle widens to 150.8°. As also shown in Figure 18, cycloheptyne **29** lies 17.2 kcal/mol below singlet **28**. Consistent with the behavior of the other two sulfur-containing singlet carbenes discussed above (**16** and **22**), the 1,2-sulfur shift in singlet **28** to form **29** has a lower barrier of 9.02 kcal/mol relative to a 1,2-carbon shift, which needs to surmount a barrier of 21.8 kcal/mol. Results of calculations on this system at the CCSD(T)/cc-pVTZ//B3LYP/6-311+G** level of theory are reported in the Supplementary Materials (Figure S9).

**Figure 19:**
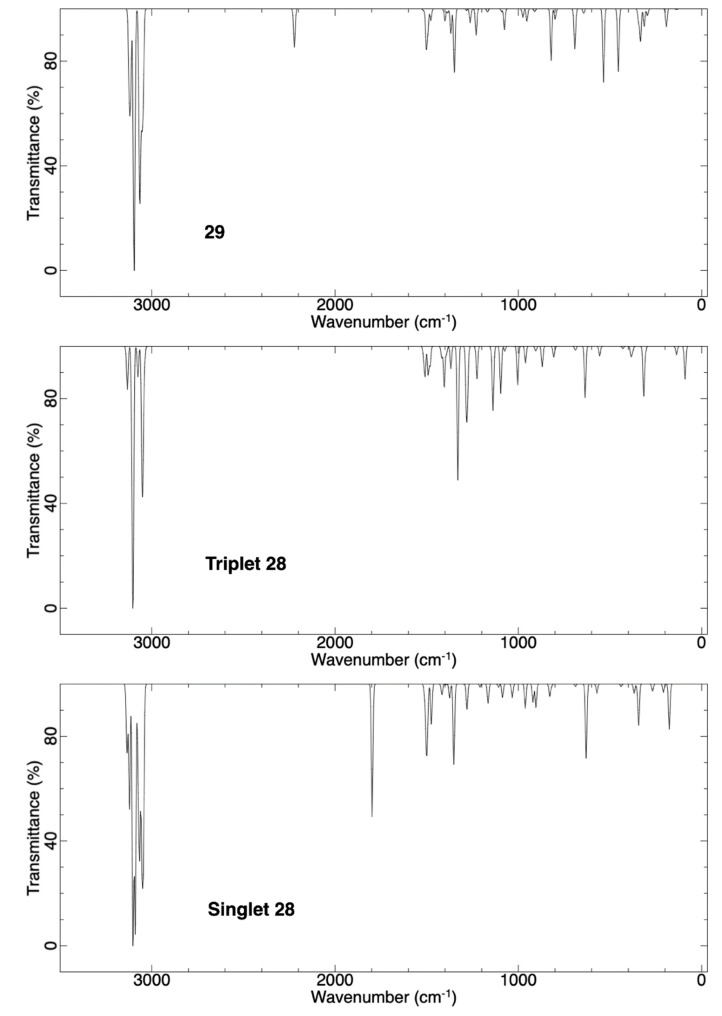
Vibrational spectra computed at CCSD/6-311+G** for singlet **28** (bottom), triplet **28** (middle), and **29** (top).

Vibrational spectra of **28** and **29**, computed at CCSD/6-311+G**, are shown in Figure 19. The spectrum of singlet **28** shows a prominent band for the C=C stretch at 1798 cm^−1^ and another weak absorbance at 630 cm^−1^ for the stretching of the bond between sulfur and the endocyclic sp^2^ carbon. Triplet **28** shows bands at 1330 cm^−1^ and 1284 cm^−1^ that correspond to the C=C stretch coupled to motions of the ring carbons. The C≡C stretch in **29** shows a weak band at 2222 cm^−1^, and the absorbance for the stretching vibrations of the bond connecting sulfur and the alkynyl group appears at 692 cm^−1^.

## 3. Computational Methods

All calculations were carried out with Gaussian 09 [55] and Gaussian 16 [56], using methods and basis sets described in the text. Graphics of structures used in the potential energy diagrams were generated with GaussView6 [57]. The vibrational spectra were produced using Avogadro [58]. Restricted methods were used for singlet species and unrestricted for triplet. The spin expectation values, <S^2^>, were found to be 2.000 for all the triplet carbenes. The stationary points obtained from geometry optimizations were verified as minima (zero imaginary frequency) or transition states (one imaginary frequency) by subsequent frequency calculations. Intrinsic reaction coordinate calculations were also performed on all transition states found at the B3LYP/6-311+G** level of theory to ensure that they connected the correct minima.

The CCSD/6-311+G** and CCSD/cc-pVTZ//CCSD/6-311+G** T1 diagnostic [59] was computed for all structures, and the values were generally less than 0.02, as recommended. However, for a few cases, specifically in the beta series, the T1 value was in the range of 0.02–0.05: not alarmingly high, but slightly above the ideal range. In order to confirm that the computed energies were still reliable, we carried out additional single-point calculations for the beta series. We chose for this purpose the Brueckner doubles method, with quadruples and triples: BD(TQ) [60,61,62]. It has the advantage of representing a slightly different approach to configuration interaction than coupled cluster, and especially with quadruple as well as triple excitations included, should provide even more reliable energies [63]. Since BD(TQ)/cc-pVTZ would be quite demanding computationally, we instead performed BD(TQ)/cc-pVDZ and BD(T)/cc-pVTZ calculations, and estimated the BD(TQ)/cc-pVTZ energy as BD(TQ)/cc-pVDZ + BD(T)/cc-pVTZ – BD(T)/cc-pVDZ. The energy differences computed using this procedure differed from those obtained using CCSD/cc-pVTZ//CCSD/6-311+G** by −0.1 to +0.7 kcal/mol, i.e., they were generally 0.3–0.5 kcal/mol higher, but never more than 0.7 higher (or more than 0.1 kcal/mol lower). The similarity of results using a significantly different correlation procedure lends confidence to the energies reported above, despite the somewhat higher than ideal T1 values for a limited number of cases. A spreadsheet summarizing the computational results is provided in the Supplementary Materials.

## 4. Conclusions

A series of cyclic alkylidenecarbenes, formally obtained by replacing the carbonyl oxygen of four-, five-, and six-membered lactams, lactones, and thiolactones with a divalent carbon, were modeled at the CCSD(T)/cc-pVTZ//B3LYP/6-311+G** and CCSD(T)/cc-pVTZ//CCSD/6-311+G** levels of theory. In all cases, the singlet carbenes were found to be considerably more stable than the triplets. The ΔE_S-T_ gap increased with increasing ring size for each type of heteroatom substituent. Structures and energies of the cycloalkynes formed by ring expansion of the singlet carbenes were modeled using both levels of theory described above, although 1-oxacyclopent-2-yne (**15**) was found to be a minimum by CCSD/6-311+G** calculations but not at the B3LYP/6-311+G* level. The structures of 1-azacyclopent-2-yne (**13**) and **15** displayed elongated lengths for the alkynl bonds. Furthermore, 2-(1-azacyclobutylidene)carbene (**12**) was found to be nearly isoenergetic with its ring-expanded isomer **13**, and **15** was notably higher in energy than 2-(1-oxacyclobutylidene)carbene (**14**). In all other cases, the cycloalkynes were lower in energy than the corresponding carbenes.

**Figure 20:**
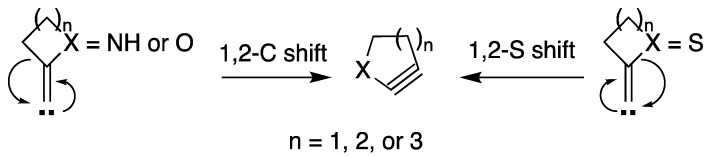
Summary of results depicting preferred rearrangement modes for the alkylidenecarbenes discussed in this study.

As ring expansion of the title alkylidenenecarbenes in this study could occur by a 1,2-shift of either the heteroatom or carbon, both pathways were modeled. For the nitrogen- and oxygen-substituted systems, the barrier for 1,2-carbon shifts were always lower in energy than those for the corresponding 1,2-nitrogen or oxygen shifts. In the case of sulfur-substituted carbenes, however, sulfur migration was significantly more facile than the carbon shift. These predictions, summarized in Figure 20, offer a platform for experimental verification using carbenes bearing appropriate isotopic labels.

**Figure 21:**
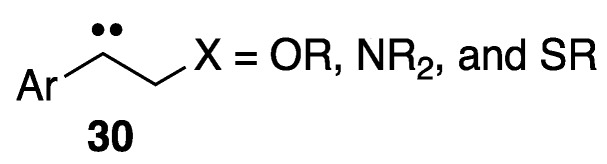
Carbenes studied by Robson and Shechter [64] to probe migratory aptitudes of substituents at the β-position.

This work bears a striking parallel to the experimental observations of Robson and Shechter who investigated the migratory aptitudes in carbenes of the type **30** (Figure 21) generated from the corresponding diazo compounds [64]. They noted that neither nitrogen nor oxygen substituents migrated to the carbenic center in **30** but the sulfur group did undergo a 1,2-shift.

As alluded to above, one explanation for the “nonmigration” of nitrogen and oxygen may be due to their ability to stabilize the transition state for 1,2-carbon shift. Results of NPA calculations [53,54] (see Supplementary Materials) reveal that in all of the title carbenes in this study (with the sole exception of the sulfur-containing 28), there is a depletion of electron density at the migration origin and an accumulation of negative charge at the migration terminus, during the carbon shift. Perhaps the nitrogen and oxygen, both of which are effective π donors, stabilize the developing positive charge at the adjacent carbon (migration origin). Another explanation is that the nitrogen- and oxygen-containing alkylidene carbenes reported in this study enjoy resonance stabilization as exemplified in Figure 2. Such resonance effects strengthen the X-C_sp2_ bond making it harder to cleave. Furthermore, the lone pair on oxygen and nitrogen can remain in conjugation with the adjacent carbon-carbon π bond even during the 1,2-carbon shift. Sulfur, on the other hand, is less effective at π conjugation with carbon due to significant differences in the sizes and energies of the interacting p orbitals in the two atoms (3p vs. 2p). Furthermore, given the larger size and nucleophilicity of sulfur, it can interact with the empty orbital on the carbenic center to initiate bonding *en route* to a 1,2-shift.

Vibrational spectra were calculated for all carbenes (singlets and triplets), and their ring-expanded isomers. These spectra are of potential value in matrix isolation experiments aimed at generating these species. They allow comparison of experimentally determined vibrational frequencies with those that are computed to facilitate identification.

## Supplementary Materials

The Supplementary Materials are available online.
