# Supplementary material for: Ring Expansion of Alkylidenecarbenes Derived from Lactams, Lactones, and Thiolactones into Strained Heterocyclic Alkynes: A Theoretical Study
Source: Molecules. 2019 Feb 7;24(3):593. doi: 10.3390/molecules24030593 (PMC6384652; doi:10.3390/molecules24030593)
Supplement: Supplementary file 1 [file molecules-24-00593-s001.zip › molecules-427496-revise-SM/molecules-427496-revise-SM.pdf]

ELECTRONIC SUPPORTING INFORMATION

The Ring Expansion of Alkylidenecarbenes derived from Lactams, Lactones, and Thiolactones into Strained Heterocyclic Alkynes: A Theoretical Study

Nguyen Nhat Thu Le,[1] Josefine Just,[1] Jonathan M. Pankauski,[1] Paul R. Rablen,[2,*] and Dasan M. Thamattoor[1,*]

[1] Department of Chemistry, Colby College, Waterville, ME 04901, USA; nnle@colby.edu (N.N.T.L.); jjust@colby.edu (J.J.); jmpank21@colby.edu (J.M.P.); dmthamat@colby.edy (D.M.T.)
[2] Department of Chemistry and Biochemistry, Swarthmore College, 500 College Avenue, Swarthmore, PA 19081, USA; prablen1@swarthmore.edu (P.R.R.)
* Correspondence: prablen1@swarthmore.edu (P.R.R.); dmthamat@colby.edy (D.M.T.)

Table of Contents

(1) **Figure 1S.** CCSD(T)/cc-pVTZ//B3LYP/6-311+G** structures and energies of singlet and triplet **12**, and the PES for the conversion of singlet **12** into 1-azacyclopent-2-yne (**13**) by two different pathways. S2
(2) **Figure 2S.** CCSD(T)/cc-pVTZ//B3LYP/6-311+G** structures and energies of singlet and triplet **14**. S3
(3) **Figure 3S.** CCSD(T)/cc-pVTZ//B3LYP/6-311+G** structures and energies of singlet and triplet **16**, and the PES for the conversion of singlet **16** into 1-thiocyclopent-2-yne (**17**) by two different pathways. S3
(4) **Figure 4S.** CCSD(T)/cc-pVTZ//B3LYP/6-311+G** structures and energies of singlet and triplet **18**, and the PES for the conversion of singlet **18** into 1-azacyclohex-2-yne (**19**) by two different pathways. S4
(5) **Figure 5S.** CCSD(T)/cc-pVTZ//B3LYP/6-311+G** structures and energies of singlet and triplet **20**, and the PES for the conversion of singlet **20** into 1-oxacyclohex-2-yne (**21**) by two different pathways. S5
(6) **Figure 6S.** CCSD(T)/cc-pVTZ//B3LYP/6-311+G** structures and energies of singlet and triplet **22**, and the PES for the conversion of singlet **21** into 1-thiocyclohex-2-yne (**23**) by two different pathways. S6
(7) **Figure 7S.** CCSD(T)/cc-pVTZ//B3LYP/6-311+G** structures and energies of singlet and triplet **24**, and the PES for the conversion of singlet **24** into 1-azacyclohept-2-yne (**25**) by two different pathways. S7
(8) **Figure 8S.** CCSD(T)/cc-pVTZ//B3LYP/6-311+G** structures and energies of singlet and triplet **26**, and the PES for the conversion of singlet **26** into 1-oxacyclohept-2-yne (**27**) by two different pathways. S8
(9) **Figure 9S.** CCSD(T)/cc-pVTZ//B3LYP/6-311+G** structures and energies of singlet and triplet **28**, and the PES for the conversion of singlet **28** into 1-thiocyclohept-2-yne (**29**) by two different pathways. S9
(10) Coordinates and energies for (R/U)B3LYP/6-311+G** optimized structures S10
(11) Summary output of (R/U)CCSD(T)/cc-pVTZ//B3LYP/6-311+G**calculations S32
(12) Coordinates and energies for (R/U)CCSD/6-311+G** optimized structures S46
(13) Summary output of (R/U)CCSD(T)/cc-pVTZ//CCSD/6-311+G** calculations S69
(14) Summary of Natural Population Analysis at CCSD/6-311+G** S84

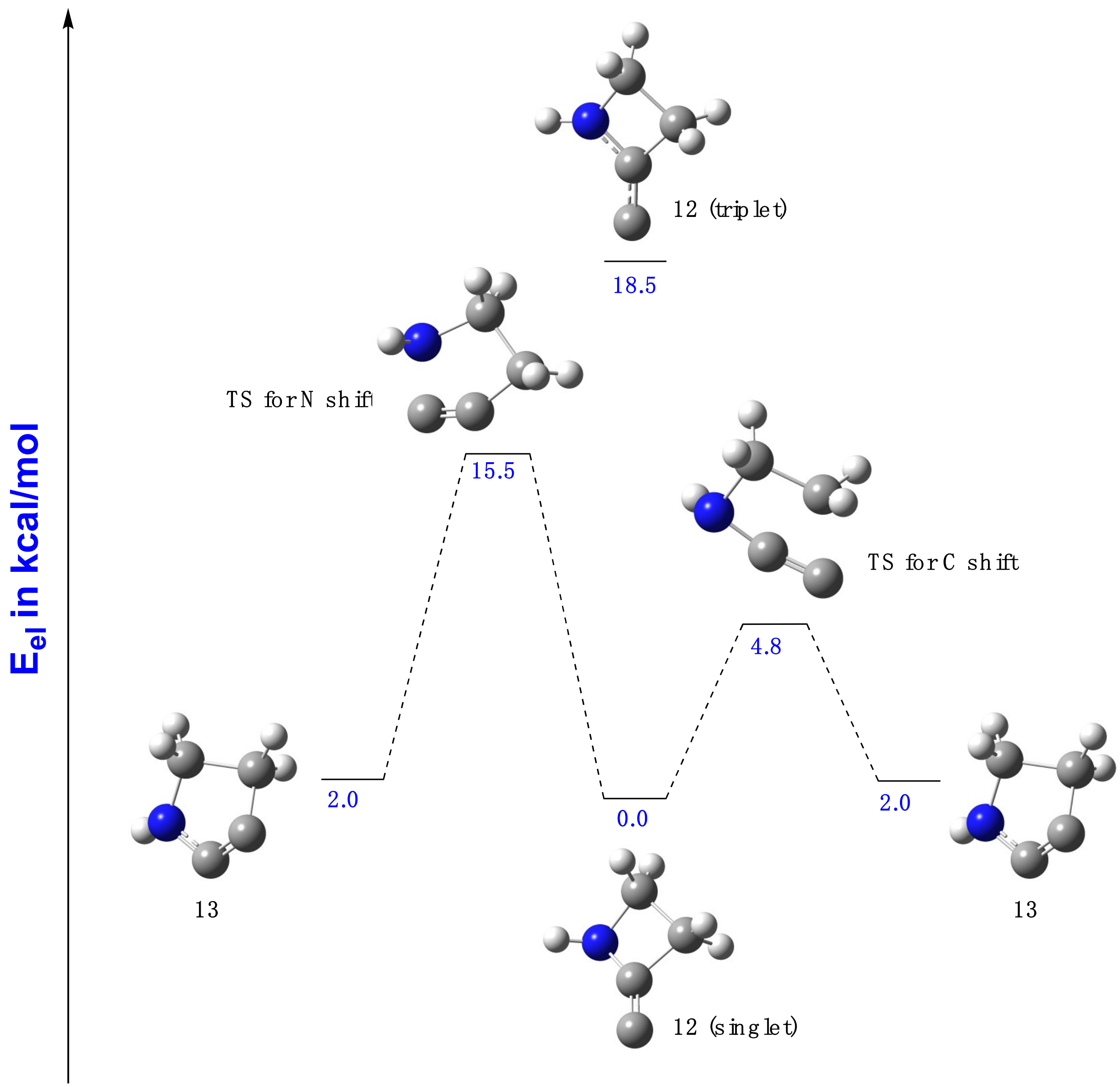

Figure 1S. CCSD(T)/cc-pVTZ//B3LYP/6-311+G** structures and energies of singlet and triplet **12**, and the PES for the conversion of singlet **12** into 1-azacyclopent-2-yne (**13**) by two different pathways.

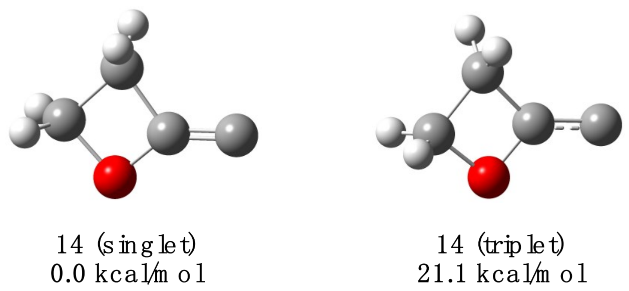

Figure 2S. CCSD(T)/cc-pVTZ//B3LYP/6-311+G** structures and energies of singlet and triplet **14**.

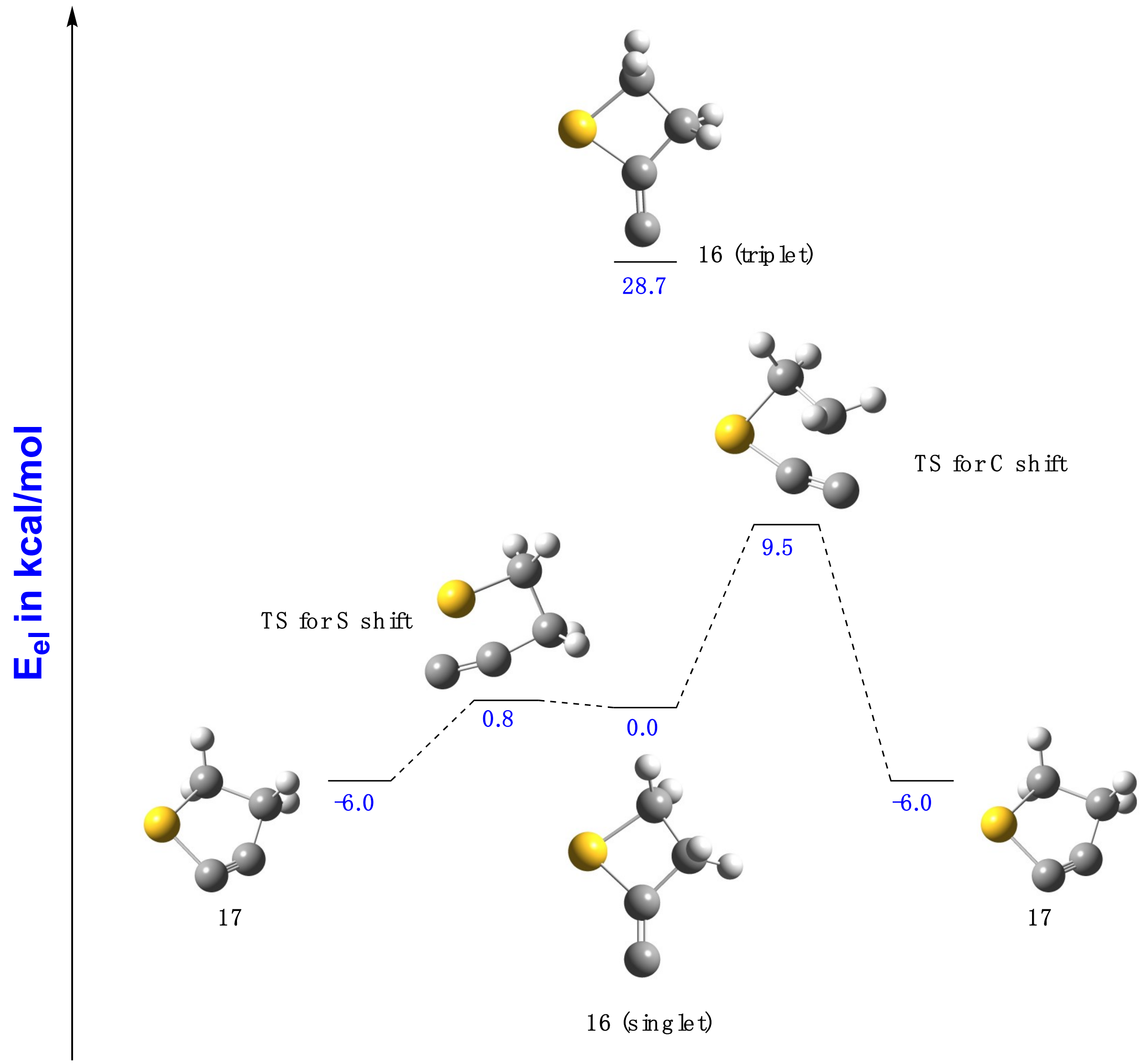

Figure 3S. CCSD(T)/cc-pVTZ//B3LYP/6-311+G** structures and energies of singlet and triplet **16**, and the PES for the conversion of singlet **16** into 1-thiocyclopent-2-yne (**17**) by two different pathways.

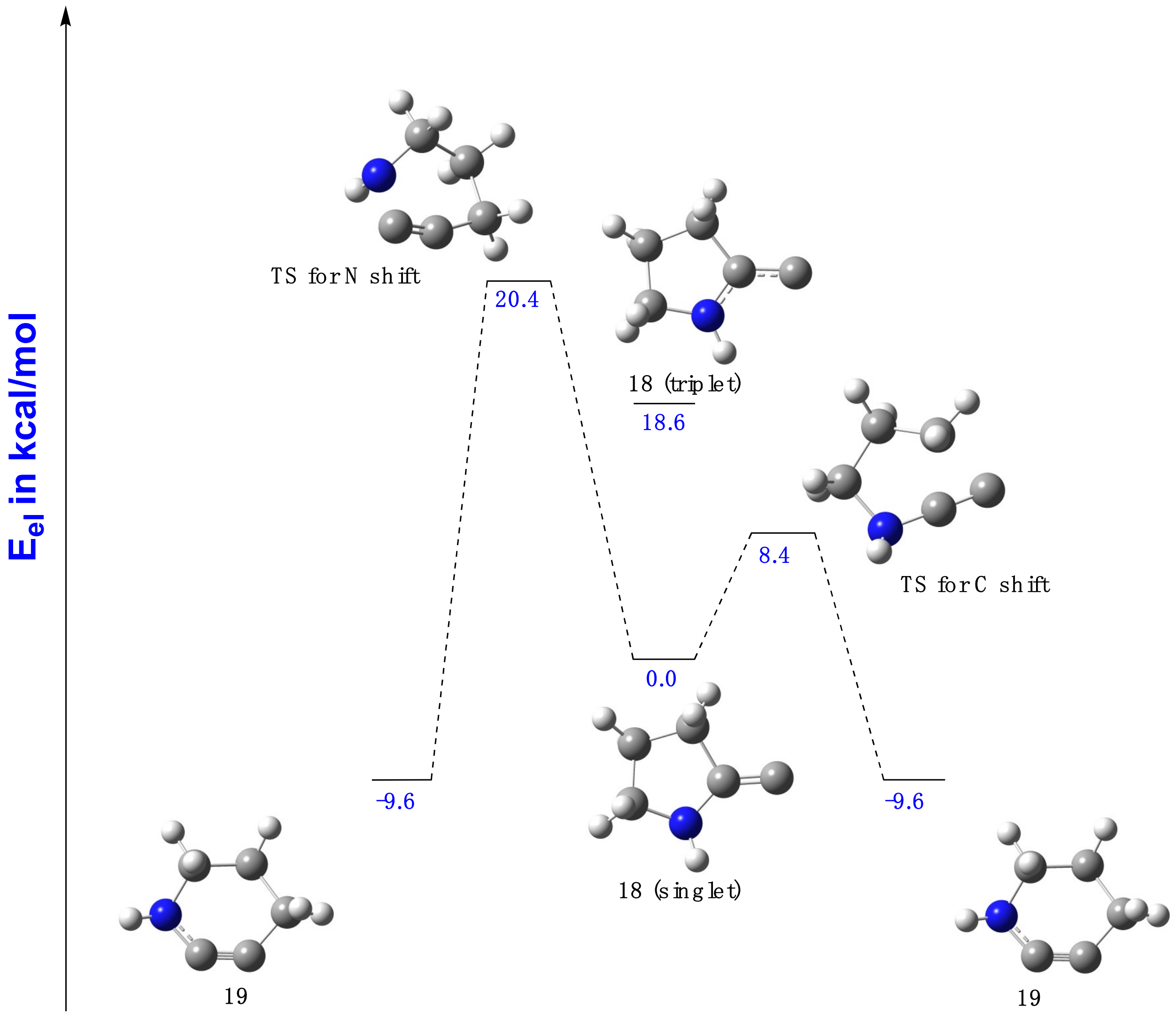

Figure 4S. CCSD(T)/cc-pVTZ//B3LYP/6-311+G** structures and energies of singlet and triplet **18**, and the PES for the conversion of singlet **18** into 1-azacyclohex-2-yne (**19**) by two different pathways.

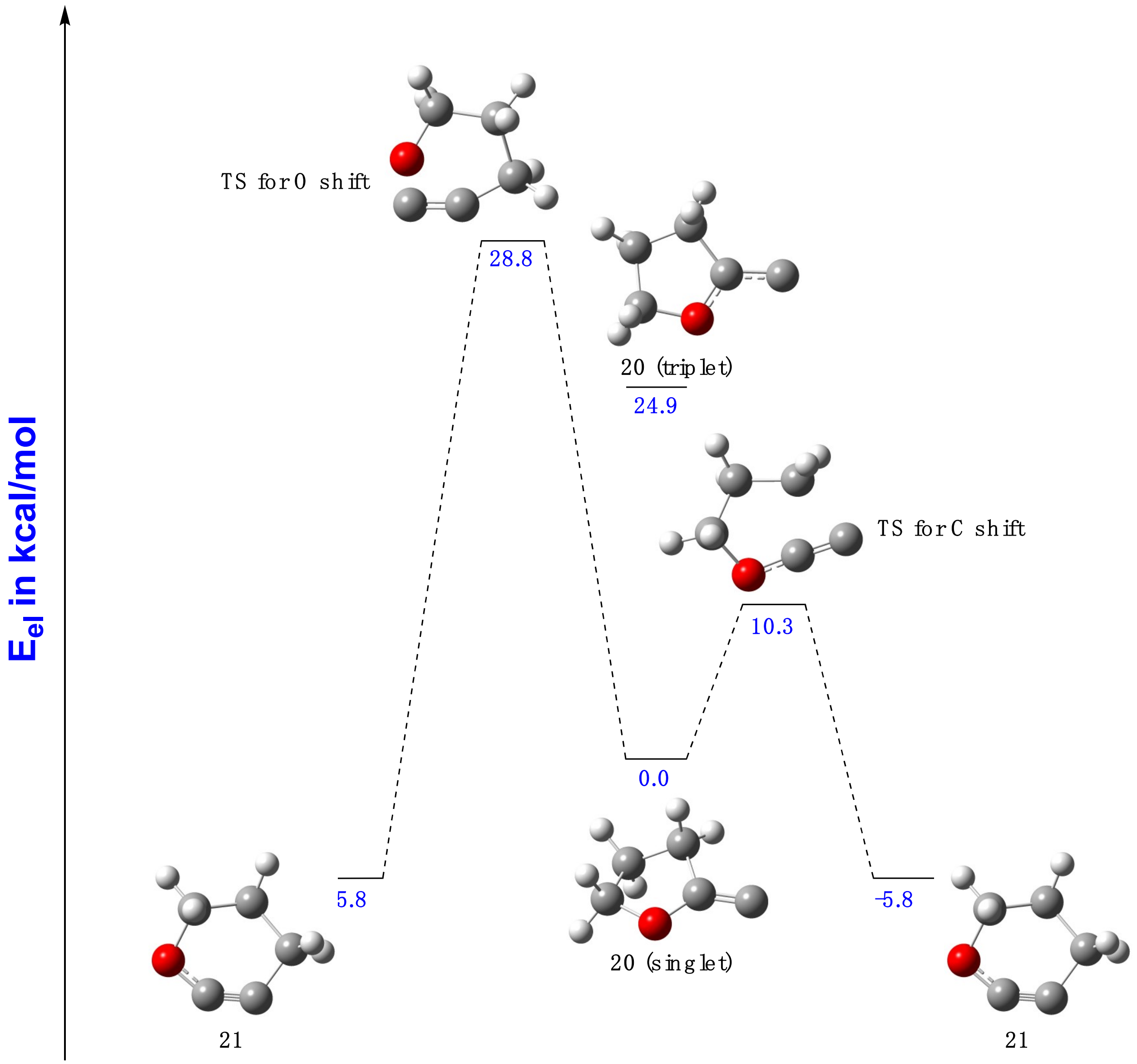

Figure 5S. CCSD(T)/cc-pVTZ//B3LYP/6-311+G** structures and energies of singlet and triplet **20**, and the PES for the conversion of singlet **20** into 1-oxacyclohex-2-yne (**21**) by the two different pathways.

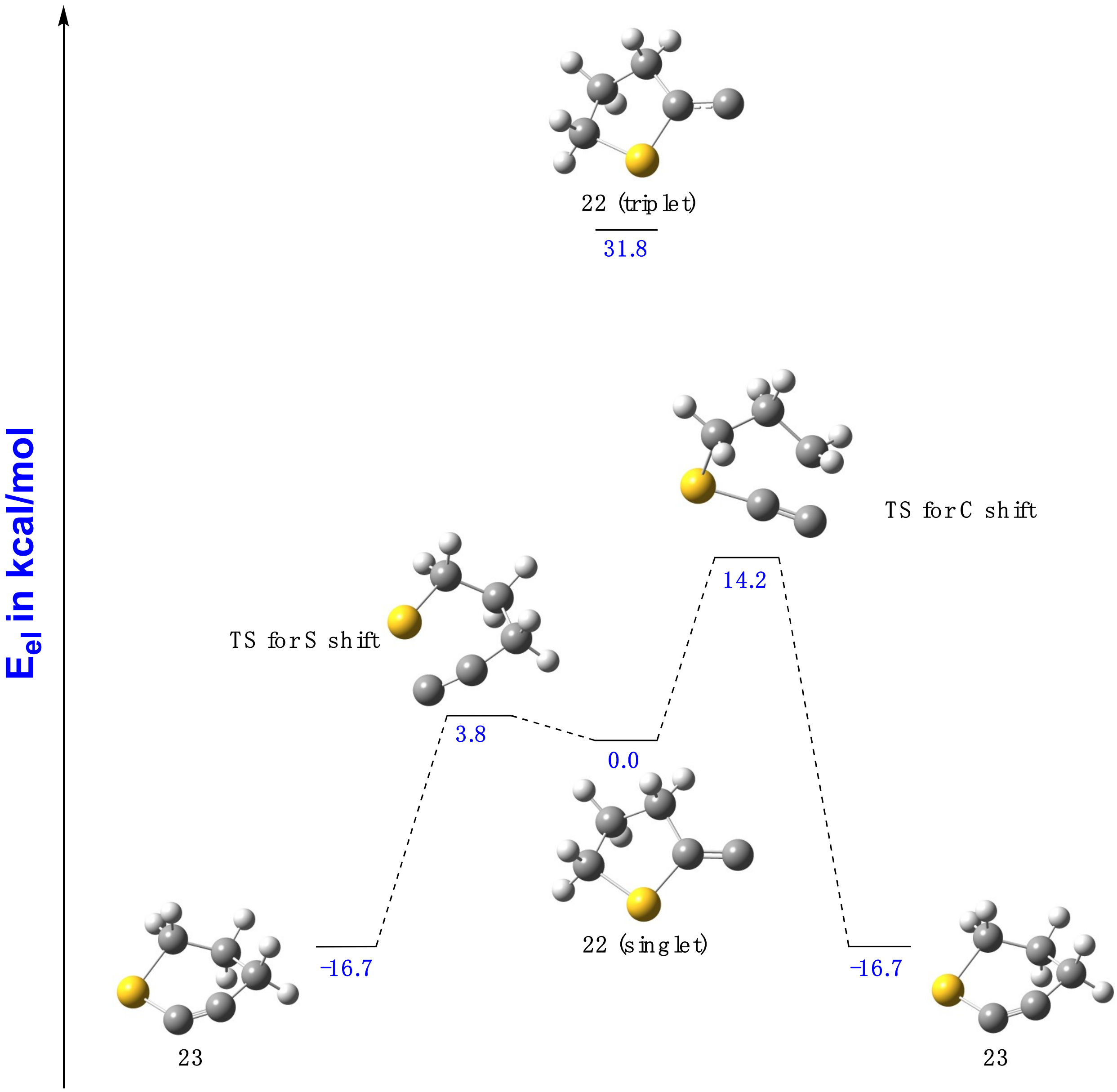

Figure 6S. CCSD(T)/cc-pVTZ//B3LYP/6-311+G** structures and energies of singlet and triplet **22**, and the PES for the conversion of singlet **22** into 1-thiacyclohex-2-yne (**23**) by the two different pathways.

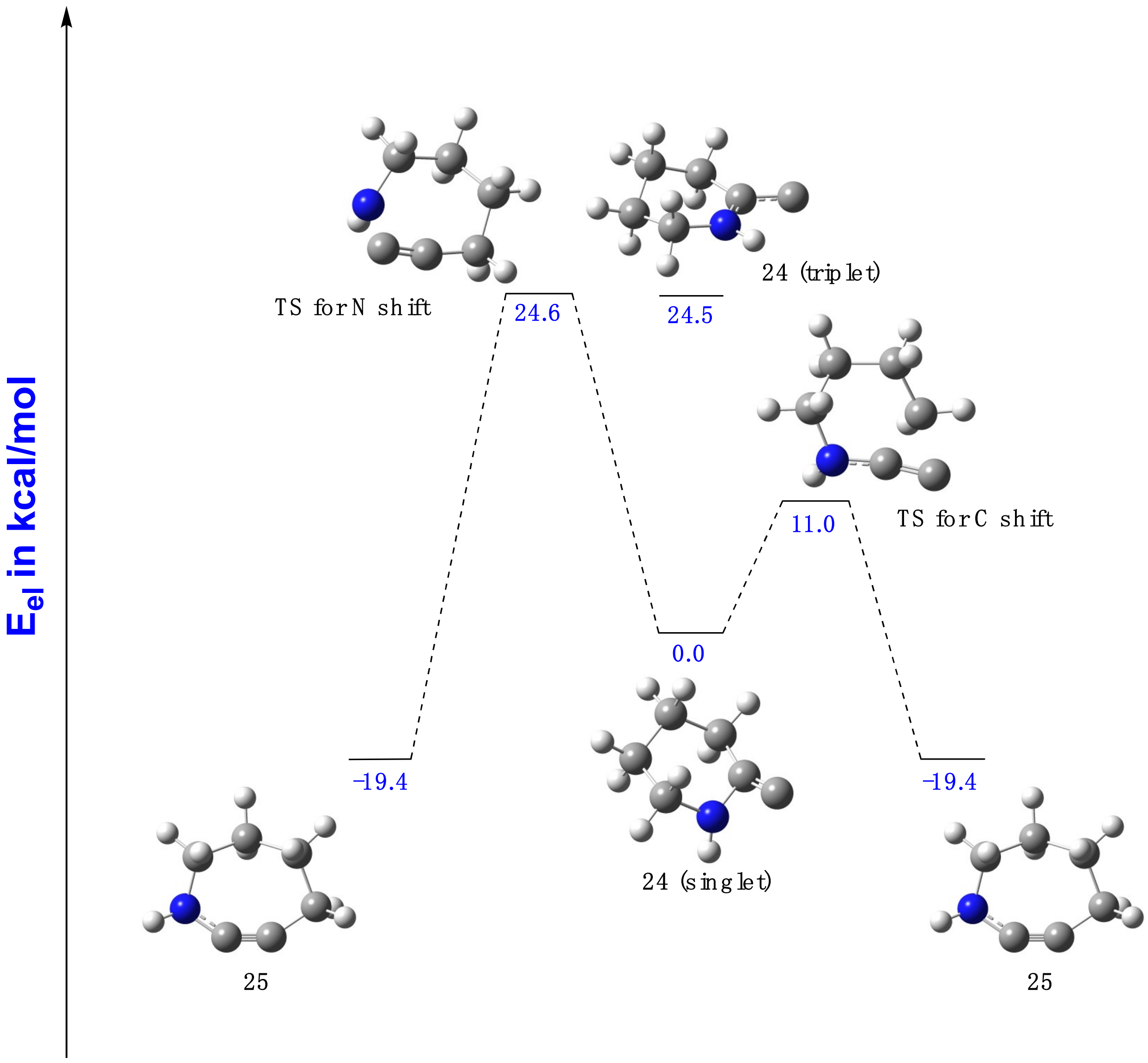

Figure 7S. CCSD(T)/cc-pVTZ//B3LYP/6-311+G** structures and energies of singlet and triplet **24**, and the PES for the conversion of singlet **24** into 1-azacyclohept-2-yne (**25**) by two different pathways.

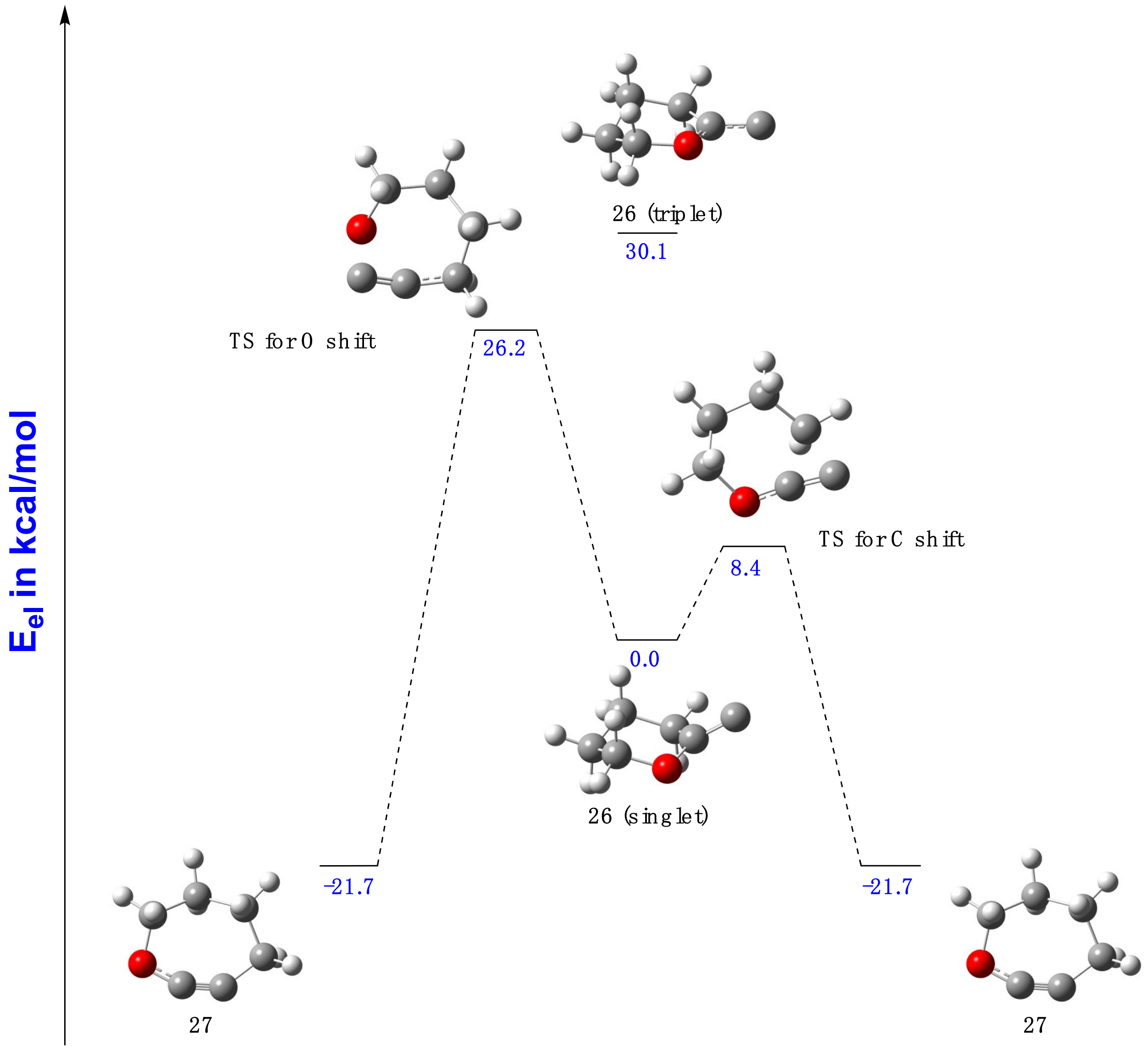

Figure 8S. CCSD(T)/cc-pVTZ//B3LYP/6-311+G** structures and energies of singlet and triplet **26**, and the PES for the conversion of singlet **26** into 1-oxacyclohept-2-yne (**27**) by two different pathways.

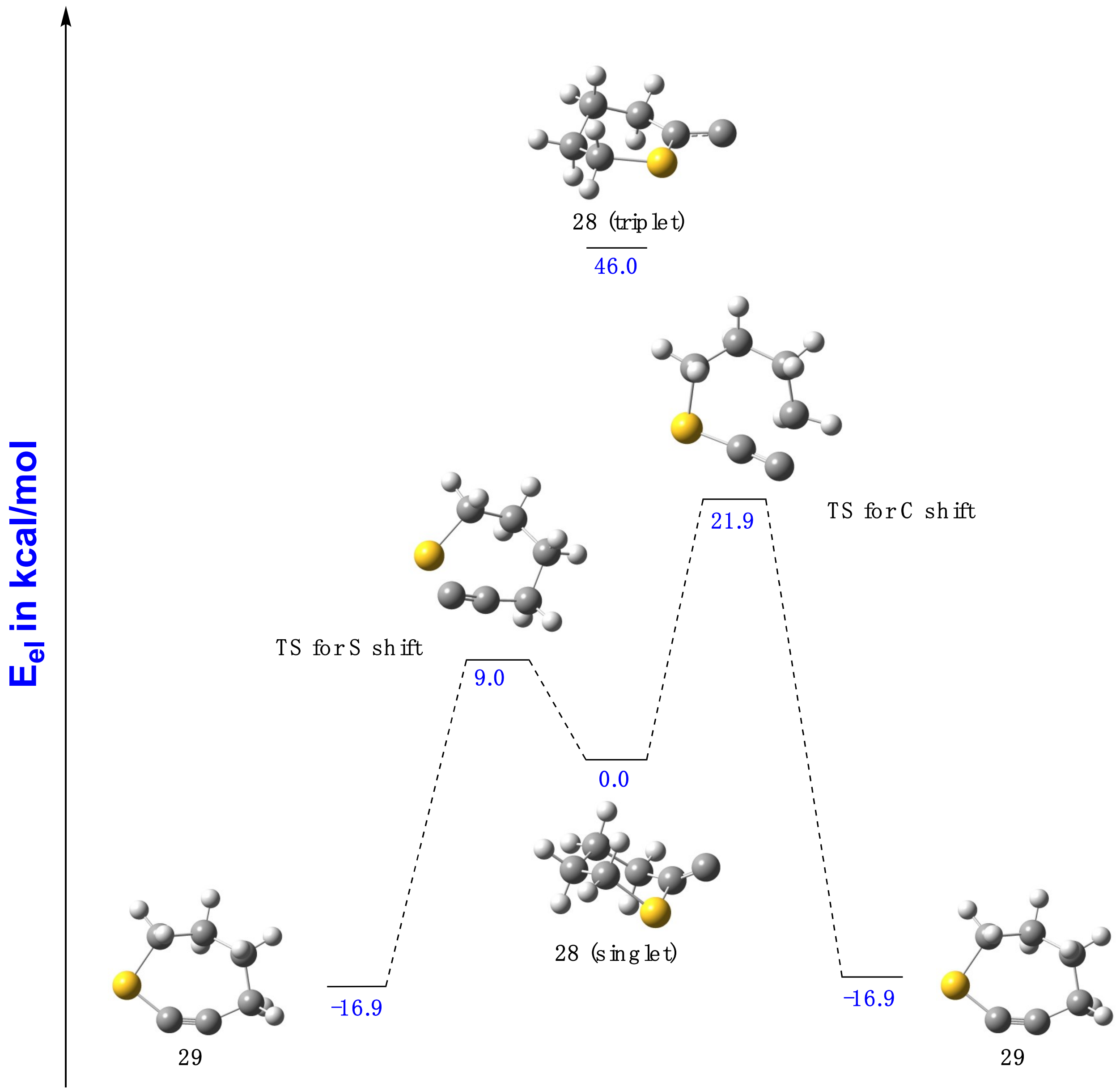

Figure 9S. CCSD(T)/cc-pVTZ//B3LYP/6-311+G** structures and energies of singlet and triplet **28**, and the PES for the conversion of singlet **28** into 1-thiocyclohept-2-yne (**29**) by two different pathways.

Coordinates and energies for (R/U)B3LYP/6-311+G optimized structures**

2-(1-azacyclobutylidene)carbene (singlet 12)

Charge = 0 Multiplicity = 1
Redundant internal coordinates found in file. (old form).
C,0,-1.3434815232,0.0147533323,0.1402852988
C,0,-0.2379572499,1.0896565029,-0.0747266241
H,0,-1.6830348883,-0.0242641438,1.1796170693
H,0,-2.2027682157,0.0437783301,-0.5312170138
H,0,-0.0990219156,1.8224868533,0.7152555112
H,0,-0.2730083851,1.5724541489,-1.0504527386
C,0,0.7281045072,-0.1295351108,-0.0596359057
C,0,2.0215570052,-0.0181197386,0.123912723
N,0,-0.3477653565,-1.0369935763,-0.2036358798
H,0,-0.3188259781,-1.8967225979,0.3323185597

Zero-point correction= 0.079194 (Hartree/Particle)
Thermal correction to Energy= 0.084095
Thermal correction to Enthalpy= 0.085039
Thermal correction to Gibbs Free Energy= 0.051655
Sum of electronic and zero-point Energies= -209.969948
Sum of electronic and thermal Energies= -209.965047
Sum of electronic and thermal Enthalpies= -209.964103
Sum of electronic and thermal Free Energies= -209.997487

2-(1-azacyclobutylidene)carbene (triplet 12)

Charge = 0 Multiplicity = 3
Redundant internal coordinates found in file. (old form).
C,0,-1.3862301421,-0.1517682689,0.0004573112
C,0,-0.4259379986,1.0743919483,0.0000769363
H,0,-2.0002535077,-0.2767213623,0.8950689186
H,0,-2.0007174549,-0.2769129977,-0.8938089894
H,0,-0.4635073267,1.699540639,0.8921286289
H,0,-0.4639698004,1.6993491644,-0.8920893321
C,0,0.7245300461,0.0405062884,-0.0001105863
C,0,2.1151718622,0.042496872,-0.0004717578
N,0,-0.1743093164,-0.9846932569,0.0002320563
H,0,-0.0175153615,-1.9861750263,0.0002988143

Zero-point correction= 0.078286 (Hartree/Particle)
Thermal correction to Energy= 0.083137
Thermal correction to Enthalpy= 0.084081
Thermal correction to Gibbs Free Energy= 0.049861
Sum of electronic and zero-point Energies= -209.950101
Sum of electronic and thermal Energies= -209.945250
Sum of electronic and thermal Enthalpies= -209.944306
Sum of electronic and thermal Free Energies= -209.978526

S**2 before annihilation 2.0085, after 2.0000

1-azacyclopent-2-yne (13)

Charge = 0 Multiplicity = 1
Redundant internal coordinates found in file. (old form).
C,0,-0.0134651444,1.090101456,0.1880164957
C,0,1.2120788901,0.1466390134,-0.2396048864
H,0,0.0787423647,1.3302768723,1.2516502472
H,0,-0.103401599,2.0218007251,-0.377836391
H,0,2.1434859555,0.4364235766,0.2486299962
H,0,1.3558679457,0.1702248526,-1.3254182741
C,0,0.5748107749,-1.119299598,0.3180465006
C,0,-0.6555943474,-1.058271952,-0.1000602607
N,0,-1.1738074709,0.1757870522,-0.0290783944
H,0,-1.9640023693,0.4570490018,-0.5903210332

Zero-point correction= 0.079286 (Hartree/Particle)
Thermal correction to Energy= 0.083900
Thermal correction to Enthalpy= 0.084845
Thermal correction to Gibbs Free Energy= 0.052323
Sum of electronic and zero-point Energies= -209.967193
Sum of electronic and thermal Energies= -209.962579
Sum of electronic and thermal Enthalpies= -209.961635
Sum of electronic and thermal Free Energies= -209.994156

TS from singlet 12 to 13 by C shift

Charge = 0 Multiplicity = 1
Redundant internal coordinates found in file. (old form).
C,0,-1.0508075951,0.601288151,0.0670123149
H,0,-1.6561330026,0.9520666993,-0.768768218
H,0,-1.47955625,0.9493807823,1.0063364359
C,0,1.6587950421,-0.3771606182,0.0523225319
C,0,0.4640457065,-0.7900647225,0.0038276945
C,0,0.479523942,1.0027283925,-0.0835173536
H,0,0.7116488876,1.4608488112,-1.0406552075
H,0,0.8299367984,1.6042079988,0.7473029666
N,0,-0.913381317,-0.8922185794,0.0789286198
H,0,-1.3230682121,-1.3422479149,-0.7345947845

Zero-point correction= 0.078555 (Hartree/Particle)
Thermal correction to Energy= 0.083153
Thermal correction to Enthalpy= 0.084098
Thermal correction to Gibbs Free Energy= 0.051118
Sum of electronic and zero-point Energies= -209.959693
Sum of electronic and thermal Energies= -209.955095
Sum of electronic and thermal Enthalpies= -209.954151
Sum of electronic and thermal Free Energies= -209.987130

****** 1 imaginary frequencies (negative Signs) ****** -214.9654 cm^{-1}

TS from singlet 12 to 13 by N shift

Charge = 0 Multiplicity = 1
Redundant internal coordinates found in file. (old form).
C,0,-0.7981904401,0.8207452227,0.1434498704
C,0,-1.1186886546,-0.6753214085,-0.0554293063
H,0,-1.2893157018,1.4735223308,-0.5774795277
H,0,-1.0108084147,1.1696529027,1.1536164188
H,0,-1.6603086997,-0.9042386417,-0.9754118204
H,0,-1.6133262548,-1.1569658704,0.7887611033
C,0,1.4964687103,-0.5123433683,0.209740181
C,0,0.3354869228,-0.951785444,-0.1333990396
N,0,0.7324674282,0.8780382005,-0.0539310289
H,0,0.9578251042,1.1820040762,-0.9993418505

Zero-point correction= 0.077837 (Hartree/Particle)
Thermal correction to Energy= 0.082367
Thermal correction to Enthalpy= 0.083311
Thermal correction to Gibbs Free Energy= 0.050786
Sum of electronic and zero-point Energies= -209.943380
Sum of electronic and thermal Energies= -209.938850
Sum of electronic and thermal Enthalpies= -209.937906
Sum of electronic and thermal Free Energies= -209.970431

****** 1 imaginary frequencies (negative Signs) ****** -177.4897 cm^{-1}

2-(1-oxacyclobutylidene)carbene (singlet 14)

Charge = 0 Multiplicity = 1
Redundant internal coordinates found in file. (old form).
C,0,1.3329680689,-0.0430833077,0.0000387537
C,0,0.2697108711,1.0825019075,-0.000063949
H,0,1.946750788,-0.1195303541,-0.8977177715
H,0,1.9466911572,-0.1194229312,0.8978451924
H,0,0.2354549277,1.6997020611,-0.8953376502
H,0,0.2353955651,1.6998096096,0.8951333144
C,0,-0.6997665874,-0.1145477956,-0.0000242849
C,0,-2.0140048194,-0.0203072869,-0.0000732756
O,0,0.2877380288,-1.0735359027,0.0000656707

Zero-point correction= 0.066643 (Hartree/Particle)
Thermal correction to Energy= 0.071438
Thermal correction to Enthalpy= 0.072382
Thermal correction to Gibbs Free Energy= 0.038972
Sum of electronic and zero-point Energies= -229.846258
Sum of electronic and thermal Energies= -229.841464
Sum of electronic and thermal Enthalpies= -229.840520
Sum of electronic and thermal Free Energies= -229.873930

2-(1-oxacyclobutylidene)carbene (triplet 14)

Charge = 0 Multiplicity = 3
Redundant internal coordinates found in file. (old form).
C,0,1.3455341023,-0.1732413204,-0.0001468612
C,0,0.4320278514,1.0623497026,0.0000018132
H,0,1.9330857494,-0.3571673838,-0.8985637283
H,0,1.9332391796,-0.3572703193,0.8981485782
H,0,0.4674092284,1.6909738018,-0.8907498049
H,0,0.4675612746,1.6908719201,0.8908192475
C,0,-0.691232416,0.0359890661,0.0000389666
C,0,-2.0833941972,0.0138692594,0.0001609859
O,0,0.1475442276,-1.0377447265,-0.0000941971

Zero-point correction= 0.065925 (Hartree/Particle)
Thermal correction to Energy= 0.070551
Thermal correction to Enthalpy= 0.071495
Thermal correction to Gibbs Free Energy= 0.037612
Sum of electronic and zero-point Energies= -229.821298
Sum of electronic and thermal Energies= -229.816672
Sum of electronic and thermal Enthalpies= -229.815728
Sum of electronic and thermal Free Energies= -229.849611

S**2 before annihilation 2.0061, after 2.0000

2-(1-thiocyclobutylidene)carbene (singlet 16)

Charge = 0 Multiplicity = 1
Redundant internal coordinates found in file. (old form).
C,0,1.2762343398,0.6728282913,0.0796624426
C,0,-0.1123381118,1.3380978301,-0.0663406191
H,0,1.9787464138,0.8789287927,-0.7250371778
H,0,1.7493577167,0.8362298021,1.0461528807
H,0,-0.2418412372,1.8373387775,-1.0272447382
H,0,-0.3692568679,2.0310119646,0.734657213
C,0,-0.9179477315,0.0306644103,-0.0120657973
C,0,-2.1977218051,-0.2111053983,0.0701176835
S,0,0.5379252832,-1.0350834702,-0.0317428876

Zero-point correction= 0.063888 (Hartree/Particle)
Thermal correction to Energy= 0.069249
Thermal correction to Enthalpy= 0.070193
Thermal correction to Gibbs Free Energy= 0.034423
Sum of electronic and zero-point Energies= -552.849794
Sum of electronic and thermal Energies= -552.844433
Sum of electronic and thermal Enthalpies= -552.843489
Sum of electronic and thermal Free Energies= -552.879259

2-(1-thiocyclobutylidene)carbene (triplet 16)

Charge = 0 Multiplicity = 3
Redundant internal coordinates found in file. (old form).
C,0,1.3385051591,0.5731893366,0.0000315234
C,0,0.0014194279,1.3474805834,-0.0000233131
H,0,1.9512159853,0.6934347918,-0.892067176

H,0,1.9511722132,0.6934855504,0.892153441
H,0,-0.1536793272,1.9727536935,-0.8835599886
H,0,-0.1537227691,1.9728037518,0.8834703153
C,0,-0.8974865948,0.1191915481,-0.0000105758
C,0,-2.249550458,-0.1163037502,-0.0000383151
S,0,0.4530043635,-1.0546325054,0.000056089

Zero-point correction= 0.062845 (Hartree/Particle)
Thermal correction to Energy= 0.067985
Thermal correction to Enthalpy= 0.068929
Thermal correction to Gibbs Free Energy= 0.033280
Sum of electronic and zero-point Energies= -552.815247
Sum of electronic and thermal Energies= -552.810108
Sum of electronic and thermal Enthalpies= -552.809164
Sum of electronic and thermal Free Energies= -552.844813

S**2 before annihilation 2.0076, after 2.0000

1-thiocyclopent-2-yne (17)

Charge = 0 Multiplicity = 1
Redundant internal coordinates found in file. (old form).
C,0,-0.501734839,1.1279700589,0.1474603321
C,0,-1.5723720238,0.021486764,-0.1748002788
H,0,-0.5690369669,2.0082026147,-0.4923766803
H,0,-0.5866824124,1.4260185432,1.1924217591
H,0,-1.8483331517,0.0126082704,-1.2339152071
H,0,-2.4665185751,0.1621805563,0.4326610898
C,0,-0.7151893378,-1.1232095049,0.194002348
C,0,0.495502539,-1.3463391895,0.0191558852
S,0,1.2023049576,0.2692427569,-0.062877618

Zero-point correction= 0.064385 (Hartree/Particle)
Thermal correction to Energy= 0.069498
Thermal correction to Enthalpy= 0.070442
Thermal correction to Gibbs Free Energy= 0.036087
Sum of electronic and zero-point Energies= -552.854953
Sum of electronic and thermal Energies= -552.849840
Sum of electronic and thermal Enthalpies= -552.848895
Sum of electronic and thermal Free Energies= -552.883251

TS from singlet 16 to 17 by C shift

Charge = 0 Multiplicity = 1
Redundant internal coordinates found in file. (old form).
C,0,0.2382316316,1.2162003554,0.1546082513
C,0,-1.2066288394,0.6919410836,-0.1708375193
H,0,0.567919829,1.9999986033,-0.5252392274
H,0,0.3209628877,1.5473062051,1.1883268096
H,0,-1.4674395597,0.8561597048,-1.2130386373
H,0,-1.9314021673,1.1488280111,0.4962262831
C,0,-0.3596170726,-0.9489696869,0.0641635132
C,0,-1.6187047928,-1.0030232455,0.1116168326
S,0,1.2618960834,-0.3305490308,-0.0562403056

Zero-point correction= 0.063074 (Hartree/Particle)
Thermal correction to Energy= 0.067927
Thermal correction to Enthalpy= 0.068871
Thermal correction to Gibbs Free Energy= 0.034801
Sum of electronic and zero-point Energies= -552.831095
Sum of electronic and thermal Energies= -552.826242
Sum of electronic and thermal Enthalpies= -552.825298
Sum of electronic and thermal Free Energies= -552.859368

****** 1 imaginary frequencies (negative Signs) ****** -322.2747 cm^{-1}

TS from singlet 16 to 17 by S shift

Charge = 0 Multiplicity = 1
Redundant internal coordinates found in file. (old form).
C,0,1.0839889987,-0.7302133491,0.3379619976
C,0,1.2934530751,0.7013548745,-0.2046436451
H,0,1.6549930175,-1.5148280949,-0.1550361397
H,0,1.1841184868,-0.7904347561,1.4208593357
H,0,1.6701275462,0.688320986,-1.2303074566
H,0,1.918574232,1.3557940776,0.4051659911
C,0,-0.1517248201,1.0479838293,-0.1482538389
C,0,-1.3212357328,1.2517309979,0.3359073751
S,0,-0.7403759134,-0.8357556852,-0.1499532293

Zero-point correction= 0.063334 (Hartree/Particle)
Thermal correction to Energy= 0.068149
Thermal correction to Enthalpy= 0.069093
Thermal correction to Gibbs Free Energy= 0.035138
Sum of electronic and zero-point Energies= -552.845288
Sum of electronic and thermal Energies= -552.840473
Sum of electronic and thermal Enthalpies= -552.839529
Sum of electronic and thermal Free Energies= -552.873484

****** 1 imaginary frequencies (negative Signs) ****** -145.7311 cm^{-1}

2-(1-azacyclopentylidene)carbene (singlet 18)

Charge = 0 Multiplicity = 1
Redundant internal coordinates found in file. (old form).
C,0,0.204379168,1.1953633373,0.1616022746
C,0,-1.2313074195,0.7790540418,-0.2319740067
C,0,-1.3040813368,-0.6883707232,0.1928631825
H,0,0.6007227346,2.0079658705,-0.444785303
H,0,0.247889326,1.4928184723,1.2116929624
H,0,-1.3645071445,0.8591060529,-1.3130448331
H,0,-1.9811857444,1.4020170407,0.258914908
H,0,-1.4989737158,-0.7720433863,1.2720353271
H,0,-2.0702950594,-1.2538812752,-0.3418984517
C,0,0.9690278167,-0.1382029342,-0.0239504344
C,0,2.2817732804,-0.054583648,-0.0284677879
N,0,0.0339640788,-1.1732435467,-0.1758278253
H,0,0.310070016,-2.0821983019,0.1679479876

Zero-point correction= 0.108975 (Hartree/Particle)
Thermal correction to Energy= 0.114712
Thermal correction to Enthalpy= 0.115656
Thermal correction to Gibbs Free Energy= 0.079884
Sum of electronic and zero-point Energies= -249.300248
Sum of electronic and thermal Energies= -249.294511
Sum of electronic and thermal Enthalpies= -249.293566
Sum of electronic and thermal Free Energies= -249.329338

2-(1-azacyclopentylidene)carbene (triplet 18)

Charge = 0 Multiplicity = 3
Redundant internal coordinates found in file. (old form).
C,0,0.04501099,1.2250798078,0.1506510568
C,0,-1.3538987917,0.6727353707,-0.1984288753
C,0,-1.2487483735,-0.8336728046,0.1138151363
H,0,0.3622761135,2.0516397587,-0.4848884235
H,0,0.0984978092,1.5753111397,1.1876771229
H,0,-1.5535013005,0.8115741954,-1.2636984198
H,0,-2.1596659737,1.1514714351,0.3591721472
H,0,-1.5660752985,-1.0660843108,1.1377957616
H,0,-1.823294786,-1.4611130379,-0.570936861
C,0,0.972035858,0.0250184646,-0.0006052411
C,0,2.3744833036,-0.0090923939,-0.0766819961
N,0,0.1867081398,-1.0777267538,-0.0336476845
H,0,0.6019443098,-1.999904871,-0.0604757235

Zero-point correction= 0.108790 (Hartree/Particle)
Thermal correction to Energy= 0.114388
Thermal correction to Enthalpy= 0.115333
Thermal correction to Gibbs Free Energy= 0.078919
Sum of electronic and zero-point Energies= -249.279114
Sum of electronic and thermal Energies= -249.273515
Sum of electronic and thermal Enthalpies= -249.272571
Sum of electronic and thermal Free Energies= -249.308985

S**2 before annihilation 2.0084, after 2.0000

1-azacyclohex-2-yne (19)

Charge = 0 Multiplicity = 1
Redundant internal coordinates found in file. (old form).
C,0,1.5801199842,-0.1711593695,0.154686294
C,0,0.6764763933,1.053711776,-0.2734712036
C,0,-0.7789794766,1.0165966826,0.2388616292
H,0,2.4678792862,-0.2126947698,-0.4816635095
H,0,1.9203627714,-0.0780343107,1.1906334777
H,0,0.6643938607,1.0992500263,-1.3655685671
H,0,1.1359727407,1.9798275459,0.0894974271
H,0,-0.7798162732,1.0932894028,1.3341093258
H,0,-1.3269062506,1.8705644388,-0.1675990901
C,0,0.6153236727,-1.2780437356,-0.0304408689
C,0,-0.6045433896,-1.2490334028,-0.0238053568
N,0,-1.5155951705,-0.2351992862,-0.1863090515
H,0,-2.4037331488,-0.3407059977,0.2891664938

Zero-point correction= 0.109574 (Hartree/Particle)
Thermal correction to Energy= 0.115199
Thermal correction to Enthalpy= 0.116143
Thermal correction to Gibbs Free Energy= 0.080765
Sum of electronic and zero-point Energies= -249.312124
Sum of electronic and thermal Energies= -249.306498
Sum of electronic and thermal Enthalpies= -249.305554
Sum of electronic and thermal Free Energies= -249.340933

TS from singlet 18 to 19 by C shift

Charge = 0 Multiplicity = 1
Redundant internal coordinates found in file. (old form).
C,0,0.7379620457,0.957767942,0.4160518772
C,0,-0.6462199006,1.1600434603,-0.2138604095
C,0,-1.4659201088,-0.1475098909,-0.0984367656
H,0,1.4315142125,1.7350422499,0.1137558038
H,0,0.7274989674,0.8906236897,1.504589675
H,0,-0.5123326632,1.424808252,-1.2646327513
H,0,-1.1653521085,1.992272287,0.2723125513
H,0,-2.1447566002,-0.1325976597,0.7573768472
H,0,-2.0595533056,-0.321896811,-0.997863129
C,0,0.7655960805,-0.7894115341,-0.0601168739
C,0,1.9079355719,-0.3420070543,-0.3793588594
N,0,-0.4912819323,-1.2634124156,0.0960205072
H,0,-0.6346212586,-1.7789765153,0.956380527

Zero-point correction= 0.107746 (Hartree/Particle)
Thermal correction to Energy= 0.113225
Thermal correction to Enthalpy= 0.114169
Thermal correction to Gibbs Free Energy= 0.078937
Sum of electronic and zero-point Energies= -249.286835
Sum of electronic and thermal Energies= -249.281356
Sum of electronic and thermal Enthalpies= -249.280411
Sum of electronic and thermal Free Energies= -249.315643

****** 1 imaginary frequencies (negative Signs) ****** -371.7409 cm^{-1}

TS from singlet 18 to 19 by N shift

Charge = 0 Multiplicity = 1
Redundant internal coordinates found in file. (old form).
C,0,-0.1559886935,1.2380260113,-0.312835786
C,0,-1.3592741415,0.4073033433,0.1485284961
H,0,-0.0660258128,1.2139966119,-1.4021516228
H,0,-0.2585085983,2.2853527431,-0.0098325972
H,0,-1.5513057704,0.5953738322,1.2089139527
H,0,-2.2750755554,0.6402082916,-0.4031489732
C,0,0.4739795814,-1.0845474194,0.0384050371
C,0,1.6707064184,-0.7223541797,-0.1965627876
N,0,1.1194619027,0.7537082528,0.2368813335
H,0,1.1650826057,0.8436087556,1.2476017254
C,0,-0.978390235,-1.1134175493,-0.021734596
H,0,-1.3114794445,-1.4876382078,-0.9947305891
H,0,-1.4452212568,-1.7168704857,0.7603964069

Zero-point correction= 0.107442 (Hartree/Particle)
Thermal correction to Energy= 0.112765
Thermal correction to Enthalpy= 0.113709
Thermal correction to Gibbs Free Energy= 0.078989
Sum of electronic and zero-point Energies= -249.268946
Sum of electronic and thermal Energies= -249.263623
Sum of electronic and thermal Enthalpies= -249.262679
Sum of electronic and thermal Free Energies= -249.297399

****** 1 imaginary frequencies (negative Signs) ****** -437.7413 cm^{-1}

2-(1-oxacyclopentylidene)carbene (singlet 20)

Charge = 0 Multiplicity = 1
Redundant internal coordinates found in file. (old form).
C,0,0.2087729331,1.1685864567,0.2411367587
C,0,-1.1897776504,0.7821773645,-0.283983928
C,0,-1.2918310993,-0.6866587101,0.1158120142
H,0,0.6573295913,2.0111021805,-0.2823234713
H,0,0.1803517348,1.3942616901,1.310788696
H,0,-1.229866693,0.8875609837,-1.3703672414
H,0,-1.9777020584,1.3956874821,0.1573826539
H,0,-1.6182391816,-0.8105279529,1.153408344
H,0,-1.9245462534,-1.2893331171,-0.535478451
O,0,0.0600988548,-1.2061985219,-0.0032342022
C,0,0.9365646437,-0.1633400623,0.0152748799
C,0,2.2416361784,-0.0907127932,-0.1559990528

Zero-point correction= 0.096347 (Hartree/Particle)
Thermal correction to Energy= 0.101896
Thermal correction to Enthalpy= 0.102840
Thermal correction to Gibbs Free Energy= 0.067392
Sum of electronic and zero-point Energies= -269.175921
Sum of electronic and thermal Energies= -269.170372
Sum of electronic and thermal Enthalpies= -269.169428
Sum of electronic and thermal Free Energies= -269.204876

2-(1-oxacyclopentylidene)carbene (triplet 20)

Charge = 0 Multiplicity = 3
Redundant internal coordinates found in file. (old form).
C,0,0.0437227641,1.2043256594,0.1646821882
C,0,-1.3436495503,0.6604929813,-0.2158071001
C,0,-1.2097558894,-0.825622935,0.1196305138
H,0,0.3866395943,2.0399285942,-0.445269442
H,0,0.0841547337,1.5308897936,1.2112287395
H,0,-1.524376051,0.7936087123,-1.2849475886
H,0,-2.1612041924,1.1330420497,0.3299759148
H,0,-1.4683617635,-1.0534663537,1.1573671639
H,0,-1.7493827121,-1.5008996341,-0.5424641883
O,0,0.2111544968,-1.1199936653,-0.0380323713
C,0,0.9482874064,0.0012088257,0.002938429
C,0,2.3520011634,-0.0377020281,-0.0914192588

Zero-point correction= 0.095879 (Hartree/Particle)
Thermal correction to Energy= 0.101324
Thermal correction to Enthalpy= 0.102268
Thermal correction to Gibbs Free Energy= 0.066076
Sum of electronic and zero-point Energies= -269.144925
Sum of electronic and thermal Energies= -269.139481
Sum of electronic and thermal Enthalpies= -269.138536
Sum of electronic and thermal Free Energies= -269.174728

S**2 before annihilation 2.0060, after 2.0000

1-oxacyclohex-2-yne (21)

Charge = 0 Multiplicity = 1
Redundant internal coordinates found in file. (old form).
C,0,1.5625390274,-0.0688921083,0.0735734479
C,0,0.5670270373,1.101748721,-0.2616312055
C,0,-0.8588523436,0.9325484628,0.2968527262
H,0,2.3350341408,-0.1248401392,-0.6970509432
H,0,2.0750889139,0.0871340593,1.0270772542
H,0,0.500710951,1.2116551611,-1.3477163974
H,0,0.9525894029,2.0468895061,0.1378460466
H,0,-0.8652794736,0.8987743806,1.3888251616
H,0,-1.5282465909,1.7127679354,-0.0625153623
O,0,-1.5078627164,-0.3445003116,-0.1631043206
C,0,0.6766213477,-1.2812849859,0.1422488914
C,0,-0.5151676966,-1.1968286813,-0.107892299

Zero-point correction= 0.096780 (Hartree/Particle)
Thermal correction to Energy= 0.102216
Thermal correction to Enthalpy= 0.103160
Thermal correction to Gibbs Free Energy= 0.068293
Sum of electronic and zero-point Energies= -269.182101
Sum of electronic and thermal Energies= -269.176666
Sum of electronic and thermal Enthalpies= -269.175722
Sum of electronic and thermal Free Energies= -269.210588

TS from singlet 20 to 21 by C shift

Charge = 0 Multiplicity = 1
Redundant internal coordinates found in file. (old form).
C,0,0.7388575093,1.084288393,0.0535966108
C,0,-0.7885113387,1.0735884174,-0.2082080981
C,0,-1.3756554285,-0.2366491077,0.3028467111
H,0,1.2546716003,1.6617428787,-0.7059548513
H,0,0.967802734,1.4599581836,1.045949454
H,0,-0.9823575004,1.1521773275,-1.2800459556
H,0,-1.2476716901,1.9348113353,0.2855178559
H,0,-1.3656647998,-0.3053230362,1.3943503438
H,0,-2.3660236442,-0.4750975999,-0.0799561073
O,0,-0.4659389468,-1.2525237585,-0.2317865969
C,0,0.7433544684,-0.7768146341,-0.0742453787
C,0,1.9256040366,-0.3787033992,0.1238980123

Zero-point correction= 0.095460 (Hartree/Particle)
Thermal correction to Energy= 0.100693
Thermal correction to Enthalpy= 0.101637
Thermal correction to Gibbs Free Energy= 0.066827
Sum of electronic and zero-point Energies= -269.158799
Sum of electronic and thermal Energies= -269.153567
Sum of electronic and thermal Enthalpies= -269.152622
Sum of electronic and thermal Free Energies= -269.187432

****** 1 imaginary frequencies (negative Signs) ****** -350.7228 cm^{-1}

TS from singlet 20 to 21 by O shift

Charge = 0 Multiplicity = 1
Redundant internal coordinates found in file. (old form).
C,0,1.0803852866,-1.0172040215,-0.0031061124
C,0,1.2768184249,0.5573960609,0.0759093903
C,0,-0.0429865471,1.2365830487,-0.2801526366
H,0,1.4304767921,-1.3932463458,-0.9697325139
H,0,1.635024741,-1.516220777,0.7936657749
H,0,2.0935242162,0.8660558171,-0.5817140532
H,0,1.548111924,0.805352173,1.1036506041
H,0,-0.0645764775,2.2738591383,0.064900472
H,0,-0.2390870763,1.2109126999,-1.3572439051
O,0,-1.1124250748,0.5880590649,0.4249953534
C,0,-0.3597119826,-1.1518529508,0.0807951632
C,0,-1.5384342264,-0.7834449076,-0.2825535368

Zero-point correction= 0.094429 (Hartree/Particle)
Thermal correction to Energy= 0.099618
Thermal correction to Enthalpy= 0.100562
Thermal correction to Gibbs Free Energy= 0.066078
Sum of electronic and zero-point Energies= -269.133868
Sum of electronic and thermal Energies= -269.128679
Sum of electronic and thermal Enthalpies= -269.127735
Sum of electronic and thermal Free Energies= -269.162218

****** 1 imaginary frequencies (negative Signs) ****** -440.9571 cm^{-1}

2-(1-thiocyclopentylidene)carbene (singlet 22)

Charge = 0 Multiplicity = 1
Redundant internal coordinates found in file. (old form).
C,0,0.2829570169,1.3880765267,0.3054378565
C,0,-1.0952757094,1.1459823809,-0.3277961893
C,0,-1.5374614623,-0.2539465166,0.0945935172
H,0,0.7864228914,2.265180294,-0.1028709177
H,0,0.1952511433,1.5151103706,1.3889720342
H,0,-1.0150166602,1.2001655137,-1.4162525425
H,0,-1.8146858543,1.9032384746,-0.0010603277
H,0,-1.9174401087,-0.2685129645,1.117292798
H,0,-2.2856291626,-0.683620438,-0.5714667059
C,0,1.0635571561,0.0967911805,0.0294520533
C,0,2.3473540219,0.1630305317,-0.2014891915
S,0,-0.019155272,-1.3232693537,0.0144566154

Zero-point correction= 0.093289 (Hartree/Particle)
Thermal correction to Energy= 0.099375
Thermal correction to Enthalpy= 0.100319
Thermal correction to Gibbs Free Energy= 0.063139
Sum of electronic and zero-point Energies= -592.174055
Sum of electronic and thermal Energies= -592.167969
Sum of electronic and thermal Enthalpies= -592.167025
Sum of electronic and thermal Free Energies= -592.204205

2-(1-thiocyclopentylidene)carbene (triplet 22)

Charge = 0 Multiplicity = 3
Redundant internal coordinates found in file. (old form).
C,0,0.0324947051,1.4314869789,0.2016221376
C,0,-1.3369975979,0.9431741628,-0.287245427
C,0,-1.4843979272,-0.5158292391,0.1619671626
H,0,0.3866523774,2.316603978,-0.3293151624
H,0,-0.0016352086,1.6902234297,1.2684557664
H,0,-1.373872599,0.9914077622,-1.379260095
H,0,-2.1568129813,1.5531561478,0.1010010703
H,0,-1.763689149,-0.5995750804,1.214994925
H,0,-2.1915262716,-1.0899669182,-0.4365569235
C,0,1.0281144952,0.2940573252,0.0262116726
C,0,2.41220224,0.3616764627,-0.0923710943
S,0,0.1996819169,-1.2468440096,-0.0304150322

Zero-point correction= 0.092570 (Hartree/Particle)
Thermal correction to Energy= 0.098460
Thermal correction to Enthalpy= 0.099404
Thermal correction to Gibbs Free Energy= 0.061872
Sum of electronic and zero-point Energies= -592.134052
Sum of electronic and thermal Energies= -592.128162
Sum of electronic and thermal Enthalpies= -592.127217
Sum of electronic and thermal Free Energies= -592.164750

S**2 before annihilation 2.0070, after 2.0000

1-thiocyclohex-2-yne (23)

Charge = 0 Multiplicity = 1
Redundant internal coordinates found in file. (old form).
C,0,1.8552487155,-0.3718766797,0.0828027618
C,0,1.1352458761,0.975649674,-0.3262845583
C,0,-0.2322109873,1.2328416327,0.3269386092
H,0,2.6170330278,-0.6074840261,-0.6650713865
H,0,2.3576220013,-0.2832954401,1.0507592807
H,0,1.0221527393,0.9857006251,-1.4129830376
H,0,1.7980639859,1.8043628721,-0.0539050773
H,0,-0.1465729517,1.2605664566,1.4153751157
H,0,-0.6365954684,2.1877582749,-0.0169618075
C,0,0.7336964774,-1.3036259925,0.1188967933
C,0,-0.4768832606,-1.3547119677,-0.0255062013
S,0,-1.568988155,-0.0262724292,-0.0859754922

Zero-point correction= 0.094213 (Hartree/Particle)
Thermal correction to Energy= 0.100057
Thermal correction to Enthalpy= 0.101001
Thermal correction to Gibbs Free Energy= 0.064826
Sum of electronic and zero-point Energies= -592.197745
Sum of electronic and thermal Energies= -592.191900
Sum of electronic and thermal Enthalpies= -592.190956
Sum of electronic and thermal Free Energies= -592.227131

TS from singlet 22 to 23 by C shift

Charge = 0 Multiplicity = 1
Redundant internal coordinates found in file. (old form).
C,0,1.5556213118,0.4074265101,-0.0231084347
C,0,0.4252492382,1.4217228251,-0.2974535564
C,0,-0.8728616958,1.0833160487,0.4287673719
H,0,2.295953292,0.4465649693,-0.8157711835
H,0,2.0152899814,0.5928628366,0.9429991802
H,0,0.2262029393,1.4731433103,-1.3700091706
H,0,0.7902097534,2.4066942213,0.0183380309
H,0,-0.756081879,1.0745450443,1.5135112652
H,0,-1.7016173287,1.7371801026,0.1558079741
C,0,0.3453183541,-1.0800057136,0.0039475828
C,0,1.5382882853,-1.4418163974,0.1996912681
S,0,-1.301828252,-0.6305857573,-0.143178328

Zero-point correction= 0.092311 (Hartree/Particle)
Thermal correction to Energy= 0.098023
Thermal correction to Enthalpy= 0.098967
Thermal correction to Gibbs Free Energy= 0.062662
Sum of electronic and zero-point Energies= -592.150162
Sum of electronic and thermal Energies= -592.144450
Sum of electronic and thermal Enthalpies= -592.143506
Sum of electronic and thermal Free Energies= -592.179811

****** 1 imaginary frequencies (negative Signs) ****** -402.6099 cm^{-1}

TS from singlet 22 to 23 by S shift

Charge = 0 Multiplicity = 1
Redundant internal coordinates found in file. (old form).
C,0,-1.4080397055,0.8068128264,0.3916688735
C,0,-1.4926576329,-0.571706169,-0.3264805448
C,0,-0.2252813587,-1.399392987,-0.0728661301
H,0,-2.1715543054,1.4940571138,0.0145066633
H,0,-1.5438442154,0.7144071387,1.4736357863
H,0,-1.5963906083,-0.3831479845,-1.3963752787
H,0,-2.3780316354,-1.1235786352,0.0035188286
H,0,-0.3059612606,-1.9806101322,0.8472133954
H,0,-0.0336628028,-2.0791518605,-0.9028964357
C,0,-0.0796454326,1.2915061708,0.0621471969
C,0,1.0082819713,1.3828024577,-0.5809147135
S,0,1.3256218163,-0.356100509,0.1912460089

Zero-point correction= 0.092575 (Hartree/Particle)
Thermal correction to Energy= 0.098224
Thermal correction to Enthalpy= 0.099168
Thermal correction to Gibbs Free Energy= 0.063090
Sum of electronic and zero-point Energies= -592.168769
Sum of electronic and thermal Energies= -592.163119
Sum of electronic and thermal Enthalpies= -592.162175
Sum of electronic and thermal Free Energies= -592.198253

****** 1 imaginary frequencies (negative Signs) ****** -294.0750 cm^{-1}

2-(1-azacyclohexylidene)carbene (singlet 24)

Charge = 0 Multiplicity = 1
Redundant internal coordinates found in file. (old form).
C,0,1.3862145681,0.762912824,-0.3248493105
C,0,0.1752632336,1.5151824202,0.2855326324
C,0,0.143924951,-1.4742375228,-0.1920327481
C,0,1.4465874953,-0.7152644454,0.113978509
H,0,0.3481126669,1.695983249,1.3542486138
H,0,0.0370464027,2.4868182186,-0.1940240154
H,0,1.3069284391,0.8102257862,-1.4161595103
H,0,2.3159502512,1.268101041,-0.0474700284
H,0,0.1674340842,-2.4898513645,0.211577214
H,0,-0.0222269872,-1.5428591385,-1.2713346749
H,0,1.6429410071,-0.7693931839,1.1922476568
H,0,2.2839933715,-1.2151269266,-0.3832046944
C,0,-1.0216065341,0.6638577678,0.0934040454
C,0,-2.0917563363,0.2643004462,-0.5501837014
N,0,-1.0000030741,-0.756051324,0.3852311428
H,0,-1.3113925392,-1.0522078473,1.3018758692

Zero-point correction= 0.138648 (Hartree/Particle)
Thermal correction to Energy= 0.145001
Thermal correction to Enthalpy= 0.145945
Thermal correction to Gibbs Free Energy= 0.108938
Sum of electronic and zero-point Energies= -288.604834
Sum of electronic and thermal Energies= -288.598481
Sum of electronic and thermal Enthalpies= -288.597537
Sum of electronic and thermal Free Energies= -288.634544

2-(1-azacyclohexylidene)carbene (triplet 24)

Charge = 0 Multiplicity = 3
Redundant internal coordinates found in file. (old form).
C,0,1.0352933971,1.1976696921,-0.3202902861
C,0,-0.4283832672,1.310233324,0.1268308305
C,0,0.9519689519,-1.3016486704,-0.1238808294
C,0,1.6842998321,-0.0357381387,0.3143312504
H,0,-0.4722587332,1.633166243,1.1751533459
H,0,-0.9692907335,2.0694052957,-0.441801343
H,0,1.085794768,1.1169463225,-1.4126213848
H,0,1.5809409869,2.1043298462,-0.0456220783
H,0,1.2498744559,-2.1625401785,0.4809205088
H,0,1.197862184,-1.5362064801,-1.1682820616

H,0,1.6494833709,0.0502432647,1.406094488
H,0,2.736670009,-0.1223547442,0.0314173737
C,0,-1.2066919594,0.0127616973,0.0274621078
C,0,-2.6152906511,-0.0452671965,-0.0034249509
N,0,-0.501409246,-1.1436083368,-0.0094134737
H,0,-1.0761243655,-1.9762249402,-0.0854844975

Zero-point correction= 0.138109 (Hartree/Particle)
Thermal correction to Energy= 0.144643
Thermal correction to Enthalpy= 0.145587
Thermal correction to Gibbs Free Energy= 0.106818
Sum of electronic and zero-point Energies= -288.577245
Sum of electronic and thermal Energies= -288.570712
Sum of electronic and thermal Enthalpies= -288.569767
Sum of electronic and thermal Free Energies= -288.608537

S**2 before annihilation 2.0087, after 2.0000

1-azacyclohepty-2-yne (25)

Charge = 0 Multiplicity = 1
Redundant internal coordinates found in file. (old form).
C,0,1.297120596,0.8898948122,0.2659987912
C,0,1.8208291752,-0.547370371,-0.0512367639
C,0,-1.376495639,0.7979812873,0.242602418
C,0,-0.0527172057,1.2932081889,-0.4008334049
H,0,2.2742502164,-0.5763021779,-1.0493136084
H,0,2.5982576657,-0.8326254143,0.6632741957
H,0,1.1904342566,0.9848765267,1.3518782326
H,0,2.062875017,1.6117017651,-0.0399794536
H,0,-2.1982349447,1.4339688086,-0.0995210998
H,0,-1.2973216207,0.9026776304,1.335249279
H,0,-0.0498257065,1.015045958,-1.4605869538
H,0,-0.1032575988,2.3866987103,-0.3716263477
C,0,0.6069614695,-1.3646198395,0.0088130792
C,0,-0.6036568792,-1.3665688995,-0.018117717
N,0,-1.7213615523,-0.5992494598,-0.1268941757
H,0,-2.5783682494,-0.9491575254,0.280487529

Zero-point correction= 0.138825 (Hartree/Particle)
Thermal correction to Energy= 0.145440
Thermal correction to Enthalpy= 0.146384
Thermal correction to Gibbs Free Energy= 0.108732
Sum of electronic and zero-point Energies= -288.637116
Sum of electronic and thermal Energies= -288.630501
Sum of electronic and thermal Enthalpies= -288.629557
Sum of electronic and thermal Free Energies= -288.667209

TS from singlet 24 to 25 by C shift

Charge = 0 Multiplicity = 1
Redundant internal coordinates found in file. (old form).
C,0,-0.2469644039,1.4644667703,0.2379758039
C,0,1.0566821461,0.9557718775,-0.3841701014
C,0,-1.3717117858,-0.8260805944,0.2849507264

C,0,-1.4939757364,0.6425018716,-0.1305244041
H,0,1.0226636785,0.8916594658,-1.4740359724
H,0,1.8773180628,1.6132237404,-0.1156985043
H,0,-0.1269458428,1.4932381709,1.326621343
H,0,-0.3912379009,2.5008836558,-0.0889897544
H,0,-2.2268454934,-1.4097260145,-0.0622898257
H,0,-1.3342397186,-0.9107694956,1.3751275853
H,0,-1.6614058374,0.700022194,-1.2138164177
H,0,-2.3766094156,1.0789468033,0.3482138936
C,0,2.1394061301,-0.4282007655,0.4419496366
C,0,1.0009661721,-0.798753164,0.0220613576
N,0,-0.1506818057,-1.4632868814,-0.2451052864
H,0,-0.2356242492,-1.7722816343,-1.20756708

Zero-point correction= 0.136619 (Hartree/Particle)
Thermal correction to Energy= 0.142983
Thermal correction to Enthalpy= 0.143927
Thermal correction to Gibbs Free Energy= 0.106772
Sum of electronic and zero-point Energies= -288.591095
Sum of electronic and thermal Energies= -288.584731
Sum of electronic and thermal Enthalpies= -288.583786
Sum of electronic and thermal Free Energies= -288.620942

****** 1 imaginary frequencies (negative Signs) ****** -409.2975 cm^{-1}

TS from singlet 24 to 25 by N shift

Charge = 0 Multiplicity = 1
Redundant internal coordinates found in file. (old form).
C,0,1.6167215333,-0.0145144208,-0.285591666
C,0,0.9967695902,1.3072688887,0.2320774082
C,0,-0.6065372753,-1.3554247294,-0.2349493671
C,0,0.8717292416,-1.2626311546,0.2062155651
H,0,1.2232787261,1.4587447902,1.2944131572
H,0,1.4180770819,2.1638433147,-0.3072576493
H,0,1.6166387923,0.0032290122,-1.3799037493
H,0,2.6598364945,-0.0556402303,0.0408807414
H,0,-1.0014787498,-2.3280748778,0.0820376732
H,0,-0.6748917736,-1.3233103762,-1.3264981302
H,0,0.9187841187,-1.3132152803,1.3023986074
H,0,1.3917521833,-2.1528567885,-0.164814341
C,0,-0.4358511717,1.2889301546,-0.0033034847
C,0,-1.6316989195,1.1011601027,-0.375919322
N,0,-1.5472904561,-0.3603634987,0.2769161323
H,0,-1.5886934159,-0.3188299064,1.2897344249

Zero-point correction= 0.136077 (Hartree/Particle)
Thermal correction to Energy= 0.142451
Thermal correction to Enthalpy= 0.143395
Thermal correction to Gibbs Free Energy= 0.106211
Sum of electronic and zero-point Energies= -288.572636
Sum of electronic and thermal Energies= -288.566261
Sum of electronic and thermal Enthalpies= -288.565317
Sum of electronic and thermal Free Energies= -288.602501

****** 1 imaginary frequencies (negative Signs) ****** -494.6920 cm^{-1}

2-(1-oxacyclohexylidene)carbene (singlet 26)

Charge = 0 Multiplicity = 1
Redundant internal coordinates found in file. (old form).
C,0,-0.8157336321,1.3025908077,0.2971731786
C,0,0.5767966066,1.2057018669,-0.3467547365
C,0,-0.9431956009,-1.2128728845,0.2310745391
C,0,-1.6640619366,0.0880124957,-0.1046528432
H,0,0.5100172816,1.3147888158,-1.4356781579
H,0,1.2471026687,1.9871570804,0.0208691112
H,0,-0.7111834009,1.3424516277,1.3868623878
H,0,-1.3000562072,2.2322513164,-0.0157928348
H,0,-1.4626026832,-2.0829733044,-0.170081665
H,0,-0.8392415469,-1.3333507436,1.3163706154
H,0,-1.8712043882,0.1187328863,-1.1800964756
H,0,-2.629211928,0.0995995112,0.4114234836
C,0,1.1474448067,-0.1836471243,-0.0644015697
C,0,2.3713123439,-0.1161297521,0.4112473408
O,0,0.3777716165,-1.272662599,-0.3592603739

Zero-point correction= 0.125149 (Hartree/Particle)
Thermal correction to Energy= 0.131658
Thermal correction to Enthalpy= 0.132603
Thermal correction to Gibbs Free Energy= 0.094998
Sum of electronic and zero-point Energies= -308.475844
Sum of electronic and thermal Energies= -308.469335
Sum of electronic and thermal Enthalpies= -308.468391
Sum of electronic and thermal Free Energies= -308.505996

2-(1-oxacyclohexylidene)carbene (triplet 26)

Charge = 0 Multiplicity = 3
Redundant internal coordinates found in file. (old form).
C,0,-1.0521793684,1.1955359123,0.2955719201
C,0,0.4252423708,1.2856771219,-0.1024858631
C,0,-0.911386142,-1.2831914819,0.1549032574
C,0,-1.6641598735,-0.0585397604,-0.3295688219
H,0,0.5092344419,1.6230422154,-1.1453399054
H,0,0.9686286319,2.0277043552,0.486720487
H,0,-1.1443404159,1.1441489583,1.3863895133
H,0,-1.5821820514,2.0955486576,-0.0255426294
H,0,-1.1774035655,-2.1882304267,-0.3908571975
H,0,-1.0852624098,-1.4582658476,1.2213025336
H,0,-1.617894793,0.0024329788,-1.4225352234
H,0,-2.717563585,-0.1720797071,-0.0570216302
C,0,1.1921610391,-0.014895933,-0.020775426
C,0,2.6031561425,-0.0603228359,-0.0055863206
O,0,0.5363275783,-1.182285207,0.0002933063

Zero-point correction= 0.124716 (Hartree/Particle)
Thermal correction to Energy= 0.131233
Thermal correction to Enthalpy= 0.132177
Thermal correction to Gibbs Free Energy= 0.093261
Sum of electronic and zero-point Energies= -308.437714

Sum of electronic and thermal Energies= -308.431197
Sum of electronic and thermal Enthalpies= -308.430253
Sum of electronic and thermal Free Energies= -308.469169

S**2 before annihilation 2.0060, after 2.0000

1-oxacyclohepty-2-yne (27)

Charge = 0 Multiplicity = 1
Redundant internal coordinates found in file. (old form).
C,0,1.2545456314,0.9282699806,0.259666275
C,0,1.8153080022,-0.4863836614,-0.0722178081
C,0,-1.412998292,0.7240872111,0.2464827203
C,0,-0.1175445049,1.2774721684,-0.3907981157
H,0,2.2106863562,-0.5108626169,-1.0941946157
H,0,2.6440097073,-0.7346454029,0.5962040402
H,0,1.1549626632,1.0138414391,1.3473055303
H,0,1.9862357969,1.684156793,-0.0475265943
H,0,-2.2845778335,1.2635632623,-0.1247603298
H,0,-1.3721940439,0.8005907848,1.3376700571
H,0,-0.1100908283,1.0255875601,-1.4572816826
H,0,-0.2324320724,2.3666035634,-0.339909851
C,0,0.6417543903,-1.3722716054,0.0567585997
C,0,-0.5604389365,-1.303825356,-0.0415350151
O,0,-1.715181036,-0.6893551202,-0.0709182102

Zero-point correction= 0.126376 (Hartree/Particle)
Thermal correction to Energy= 0.132727
Thermal correction to Enthalpy= 0.133671
Thermal correction to Gibbs Free Energy= 0.096541
Sum of electronic and zero-point Energies= -308.507809
Sum of electronic and thermal Energies= -308.501458
Sum of electronic and thermal Enthalpies= -308.500514
Sum of electronic and thermal Free Energies= -308.537644

TS from singlet 26 to 27 by C shift

Charge = 0 Multiplicity = 1
Redundant internal coordinates found in file. (old form).
C,0,-0.1605724764,1.4668659868,0.2499047583
C,0,1.1198173177,0.9081198312,-0.3749069834
C,0,-1.381699053,-0.7792898132,0.2610608245
C,0,-1.4399262465,0.6915834756,-0.11704097
H,0,1.0695315374,0.8187139555,-1.4621768275
H,0,1.9642650933,1.5428728132,-0.1293749924
H,0,-0.0353430926,1.4942500413,1.3375140406
H,0,-0.2741720553,2.5055179035,-0.0813612241
H,0,-2.2356108366,-1.3393411293,-0.1163229418
H,0,-1.2894683213,-0.92631451,1.3408564797
H,0,-1.6233406724,0.7718149987,-1.1943253174
H,0,-2.3025414853,1.1394102864,0.3870062698
C,0,0.9060553125,-0.8835765122,0.0117309019
C,0,2.0338738025,-0.5189302349,0.4531732302
O,0,-0.2186748238,-1.4130520927,-0.3731112484

Zero-point correction= 0.124172 (Hartree/Particle)
Thermal correction to Energy= 0.130329
Thermal correction to Enthalpy= 0.131273
Thermal correction to Gibbs Free Energy= 0.094460
Sum of electronic and zero-point Energies= -308.463018
Sum of electronic and thermal Energies= -308.456862
Sum of electronic and thermal Enthalpies= -308.455917
Sum of electronic and thermal Free Energies= -308.492731

****** 1 imaginary frequencies (negative Signs) ****** -430.4986 cm^{-1}

TS from singlet 26 to 27 by O shift

Charge = 0 Multiplicity = 1
Redundant internal coordinates found in file. (old form).
C,0,-1.5734736644,0.2113437246,-0.3002504313
C,0,-1.1468781566,-1.1797530036,0.2385931605
C,0,0.8110034193,1.2369930496,-0.207932646
C,0,-0.6693625158,1.3590666168,0.180780412
H,0,-1.4020854782,-1.3060185419,1.297099753
H,0,-1.663857482,-1.9790752796,-0.3076100113
H,0,-1.5690436848,0.1741712167,-1.3938253679
H,0,-2.6026219768,0.4024531308,0.0177157187
H,0,1.3549431396,2.1182004352,0.1459774122
H,0,0.9374847503,1.1608119731,-1.293487434
H,0,-0.730821604,1.447424263,1.2716863924
H,0,-1.04865571,2.3003279553,-0.232388251
C,0,0.2734981443,-1.3473831088,0.0194405394
C,0,1.4617431105,-1.1850802153,-0.4248037076
O,0,1.4736007087,0.138735784,0.4324114609

Zero-point correction= 0.123262 (Hartree/Particle)
Thermal correction to Energy= 0.129406
Thermal correction to Enthalpy= 0.130350
Thermal correction to Gibbs Free Energy= 0.093621
Sum of electronic and zero-point Energies= -308.439827
Sum of electronic and thermal Energies= -308.433683
Sum of electronic and thermal Enthalpies= -308.432738
Sum of electronic and thermal Free Energies= -308.469467

****** 1 imaginary frequencies (negative Signs) ****** -468.1541cm^{-1}

2-(1-thiocyclohexylidene)carbene (singlet 28)

Charge = 0 Multiplicity = 1
Redundant internal coordinates found in file. (old form).
C,0,-1.7694379561,0.4277200646,0.2240685371
C,0,-0.7985958611,1.4690749695,-0.3842721009
C,0,-0.0294657316,-1.4908429217,0.4060893538
C,0,-1.4122215611,-1.0399705831,-0.0856375777
H,0,-0.8898442722,1.4905017519,-1.4752309418
H,0,-1.0366513695,2.4691084513,-0.0111362215
H,0,-1.7881339664,0.571472994,1.3094883006
H,0,-2.7805662346,0.6295403307,-0.1421003785
H,0,0.1572766057,-2.5364653094,0.1492351929

H,0,0.0868992792,-1.3687048127,1.4855686439
H,0,-1.476412975,-1.2143514773,-1.1658480432
H,0,-2.1664651295,-1.6859031361,0.3778465894
C,0,0.5894822012,1.1197009033,0.0019273063
C,0,1.5864860471,1.1501733737,0.8357353625
S,0,1.305984924,-0.5106955988,-0.4073440228

Zero-point correction= 0.122510 (Hartree/Particle)
Thermal correction to Energy= 0.129334
Thermal correction to Enthalpy= 0.130279
Thermal correction to Gibbs Free Energy= 0.091907
Sum of electronic and zero-point Energies= -631.486752
Sum of electronic and thermal Energies= -631.479928
Sum of electronic and thermal Enthalpies= -631.478984
Sum of electronic and thermal Free Energies= -631.517355

2-(1-thiocyclohexylidene)carbene (triplet 28)

Charge = 0 Multiplicity = 3
Redundant internal coordinates found in file. (old form).
C,0,-1.1950663302,1.3021063105,0.3431804799
C,0,0.2442827816,1.5589095427,-0.1183045713
C,0,-1.1905020989,-1.2267504271,0.1208098662
C,0,-1.8190981592,0.0826935277,-0.3435837274
H,0,0.2382657786,1.8787006752,-1.1696266607
H,0,0.6911246987,2.3869332442,0.43916004
H,0,-1.2178971739,1.1605353432,1.4302909891
H,0,-1.7935284535,2.1927183781,0.1323804454
H,0,-1.5252929668,-2.0771605853,-0.4763427942
H,0,-1.4362644699,-1.429799118,1.1664401004
H,0,-1.7225601416,0.178656033,-1.43066007
H,0,-2.8908711522,0.0346617887,-0.1250759314
C,0,1.2082922235,0.3939041391,-0.0258054551
C,0,2.5963200311,0.518210623,-0.0076403984
S,0,0.6621564329,-1.2561824752,0.0140426876

Zero-point correction= 0.121368 (Hartree/Particle)
Thermal correction to Energy= 0.128407
Thermal correction to Enthalpy= 0.129351
Thermal correction to Gibbs Free Energy= 0.089026
Sum of electronic and zero-point Energies= -631.427492
Sum of electronic and thermal Energies= -631.420454
Sum of electronic and thermal Enthalpies= -631.419510
Sum of electronic and thermal Free Energies= -631.459835

S**2 before annihilation 2.0072, after 2.0000

1-thiocyclohepty-2-yne (29)

Charge = 0 Multiplicity = 1
Redundant internal coordinates found in file. (old form).
C,0,1.763681238,0.6903511646,0.2422869733
C,0,1.9959529863,-0.8239899285,-0.0509076135
C,0,-0.8374255759,1.2328619098,0.290959068
C,0,0.5251360517,1.3380244322,-0.440971706

H,0,2.4039818612,-0.954323524,-1.0600217226
H,0,2.7261154903,-1.2373993,0.6506199011
H,0,1.6799934002,0.8257132967,1.325427997
H,0,2.6608822178,1.2332408164,-0.0727973813
H,0,-1.4882455444,2.0586062656,-0.0077954104
H,0,-0.6931684771,1.2895128602,1.372566656
H,0,0.4240496469,0.9759009908,-1.4691258832
H,0,0.7324162223,2.4109469006,-0.5172726181
C,0,0.6678949334,-1.4163251351,0.050027066
C,0,-0.5418848845,-1.3556926055,-0.0077625447
S,0,-1.8677295661,-0.2870401438,-0.0450337816

Zero-point correction= 0.123350 (Hartree/Particle)
Thermal correction to Energy= 0.130262
Thermal correction to Enthalpy= 0.131206
Thermal correction to Gibbs Free Energy= 0.092442
Sum of electronic and zero-point Energies= -631.513818
Sum of electronic and thermal Energies= -631.506906
Sum of electronic and thermal Enthalpies= -631.505962
Sum of electronic and thermal Free Energies= -631.544726

TS from singlet 28 to 29 by C shift

Charge = 0 Multiplicity = 1
Redundant internal coordinates found in file. (old form).
C,0,1.3834327722,1.0692424489,0.124601873
C,0,1.5966577575,-0.3173546163,-0.4739908425
C,0,-1.1991060669,0.9952844454,0.4551297747
C,0,0.0108793003,1.7207820163,-0.1350859015
H,0,1.2941341819,-0.4001970138,-1.5187888282
H,0,2.6403924619,-0.6094655938,-0.4091625348
H,0,1.5789569467,1.0177701765,1.2008576027
H,0,2.149789451,1.7305211927,-0.2976358871
H,0,-2.1238110484,1.5256863273,0.2186063496
H,0,-1.1252424284,0.8837250224,1.5383454753
H,0,-0.13743895,1.8357342811,-1.2147257978
H,0,0.026129676,2.732489238,0.2865601323
C,0,0.1080947719,-1.2903551572,0.1572817355
C,0,1.1812649421,-1.7757407004,0.6191388532
S,0,-1.4246427678,-0.695520067,-0.2679150047

Zero-point correction= 0.120845 (Hartree/Particle)
Thermal correction to Energy= 0.127561
Thermal correction to Enthalpy= 0.128505
Thermal correction to Gibbs Free Energy= 0.090135
Sum of electronic and zero-point Energies= -631.454859
Sum of electronic and thermal Energies= -631.448143
Sum of electronic and thermal Enthalpies= -631.447199
Sum of electronic and thermal Free Energies= -631.485569

****** 1 imaginary frequencies (negative Signs) ****** -476.4702 cm^{-1}

TS from singlet 28 to 29 by S shift

Charge = 0 Multiplicity = 1
Redundant internal coordinates found in file. (old form).
C,0,1.8900214565,0.0401400227,0.2089748649
C,0,1.2678496407,-1.2754387478,-0.3137657887
C,0,-0.3123475676,1.4095136914,0.3950000924
C,0,1.1088818763,1.3051669352,-0.1800482756
H,0,1.3446671388,-1.3385125955,-1.4057784471
H,0,1.8042098499,-2.1408286905,0.0926116351
H,0,1.9691349621,-0.0196382851,1.2993155231
H,0,2.9081717296,0.1159428192,-0.1839313154
H,0,-0.731209438,2.3954131685,0.1731995108
H,0,-0.3184357599,1.2809472656,1.4804284591
H,0,1.0588564394,1.3901909702,-1.2714076769
H,0,1.6747668772,2.1746439744,0.1746505588
C,0,-0.1254762028,-1.384499015,0.0869094133
C,0,-1.2835763299,-1.4201163528,0.5827978911
S,0,-1.5599456724,0.2550278396,-0.314875445

Zero-point correction= 0.121562 (Hartree/Particle)
Thermal correction to Energy= 0.128117
Thermal correction to Enthalpy= 0.129062
Thermal correction to Gibbs Free Energy= 0.091023
Sum of electronic and zero-point Energies= -631.476956
Sum of electronic and thermal Energies= -631.470402
Sum of electronic and thermal Enthalpies= -631.469457
Sum of electronic and thermal Free Energies= -631.507496

****** 1 imaginary frequencies (negative Signs) ****** -320.9202 cm^{-1}

Summary output of (R/U)CCSD(T)/cc-pVTZ//B3LYP/6-311+Gcalculations**

2-(1-azacyclobutylidene)carbene (singlet 12)

1\1\GINC-NSCC-N11\SP\RCCSD(T)-FC\CC-pVTZ\C4H5N1\DMTHAMAT\16-Dec-2018\0
\\# rccsd=(t,t1diag)/cc-pvtz geom=connectivity\\Betalactamcarbene sing
let\\0,1\C,0,-1.343404,0.015001,0.14058\C,0,-0.237766,1.089694,-0.0748
95\H,0,-1.682752,-0.023736,1.179989\H,0,-2.202823,0.044003,-0.530754\H
,0,-0.098563,1.822679,0.714897\H,0,-0.272944,1.57228,-1.050721\C,0,0.7
2812,-0.129636,-0.059729\C,0,2.021626,-0.01837,0.123532\N,0,-0.347912,
-1.036968,-0.20331\H,0,-0.31899,-1.896583,0.33283\\Version=ES64L-G16Re
vA.03\State=1-A\HF=-208.7213894\MP2=-209.5331152\MP3=-209.5675915\MP4D
=-209.5877055\MP4DQ=-209.565349\MP4SDQ=-209.5732201\CCSD=-209.5730701\
CCSD(T)=-209.6135694\RMSD=5.120e-09\PG=C01 [X(C4H5N1)]\\@

T1 Diagnostic = 0.01511286

2-(1-azacyclobutylidene)carbene (triplet 12)

1\1\GINC-NSCC-N11\SP\UCCSD(T)-FC\CC-pVTZ\C4H5N1(3)\DMTHAMAT\16-Dec-201
8\0\\# uccsd=(t,t1diag)/cc-pvtz geom=connectivity\\Betalactamcarbene t
riplet\\0,3\C,0,-1.386202,-0.151788,0.\C,0,-0.425928,1.074386,0.\H,0,-
2.000456,-0.276846,0.894439\H,0,-2.000456,-0.276846,-0.894439\H,0,-0.4
63737,1.699439,0.892109\H,0,-0.463737,1.699438,-0.892109\C,0,0.724555,
0.040517,0.\C,0,2.115197,0.042528,0.\N,0,-0.174269,-0.984695,0.\H,0,-0
.017461,-1.986175,0.\\Version=ES64L-G16RevA.03\State=3-A"\HF=-208.7182
948\MP2=-209.5043886\MP3=-209.5408934\MP4D=-209.5589257\MP4DQ=-209.537
9743\PUHF=-208.7226274\PMP2-0=-209.5073561\PMP3-0=-209.5426322\MP4SDQ=
-209.5457531\CCSD=-209.545694\CCSD(T)=-209.584102\S2=2.030644\S2-1=2.0
11153\S2A=2.00043\RMSD=7.258e-09\PG=CS [SG(C4H1N1),X(H4)]\\@

T1 Diagnostic = 0.01728975

1-azacyclopent-2-yne (13)

1\1\GINC-NSCC-N2\SP\RCCSD(T)-FC\CC-pVTZ\C4H5N1\DMTHAMAT\16-Dec-2018\0\
\# rccsd=(t,t1diag)/cc-pvtz geom=connectivity\\Azacyclopentyne singlet
\\0,1\C,0,0.013307,1.090041,-0.188129\C,0,-1.212033,0.146463,0.239823\
H,0,-0.079107,1.330053,-1.251782\H,0,0.103202,2.021833,0.377577\H,0,-2
.14356,0.436046,-0.248301\H,0,-1.355649,0.170182,1.325657\C,0,-0.57467
4,-1.119463,-0.317753\C,0,0.65579,-1.0582,0.100146\N,0,1.173815,0.1759
23,0.028906\H,0,1.964061,0.457377,0.589981\\Version=ES64L-G16RevA.03\S
tate=1-A\HF=-208.6986845\MP2=-209.5235954\MP3=-209.5525679\MP4D=-209.5
721829\MP4DQ=-209.5485655\MP4SDQ=-209.5602967\CCSD=-209.5637483\CCSD(T
)=-209.6104394\RMSD=4.475e-09\PG=C01 [X(C4H5N1)]\\@

T1 Diagnostic = 0.03994815

TS from singlet 12 to 13 by C shift

1\1\GINC-NSCC-N2\SP\RCCSD(T)-FC\CC-pVTZ\C4H5N1\DMTHAMAT\16-Dec-2018\0\
\# rccsd=(t,t1diag)/cc-pvtz geom=connectivity\\TS to azacyclopentyne -
C shift\\0,1\C,0,1.050276,0.602158,-0.067013\H,0,1.655316,0.953428,0.
768768\H,0,1.478742,0.950599,-1.006337\C,0,-1.658529,-0.378496,-0.0523

23\C,0,-0.463444,-0.790427,-0.003828\C,0,-0.480381,1.002352,0.083517\H
,0,-0.712879,1.460284,1.040655\H,0,-0.831283,1.603547,-0.747303\N,0,0.
914065,-0.891461,-0.078929\H,0,1.324118,-1.341156,0.734595\\Version=ES
64L-G16RevA.03\State=1-A\HF=-208.6975103\MP2=-209.5355031\MP3=-209.559
0878\MP4D=-209.5793348\MP4DQ=-209.5545536\MP4SDQ=-209.5625906\CCSD=-20
9.5615427\CCSD(T)=-209.6059138\RMSD=9.650e-09\PG=C01 [X(C4H5N1)]\\@

T1 Diagnostic = 0.01311808

TS from singlet 12 to 13 by N shift

1\1\GINC-NSCC-N2\SP\RCCSD(T)-FC\CC-pVTZ\C4H5N1\DMTHAMAT\16-Dec-2018\0\
\# rccsd=(t,t1diag)/cc-pvtz geom=connectivity\\TS to azacyclopentyne -
N shift\\0,1\C,0,0.797868,0.821219,-0.143157\C,0,1.119061,-0.674717,0
.055582\H,0,1.288484,1.474133,0.577995\H,0,1.010571,1.170383,-1.153217
\H,0,1.660559,-0.903502,0.975669\H,0,1.614154,-1.15599,-0.788553\C,0,-
1.49611,-0.513002,-0.210233\C,0,-0.334997,-0.951917,0.133138\N,0,-0.73
2868,0.877721,0.053841\H,0,-0.958619,1.181433,0.999239\\Version=ES64L-
G16RevA.03\State=1-A\HF=-208.6752787\MP2=-209.5123155\MP3=-209.5365509
\MP4D=-209.5579008\MP4DQ=-209.5328074\MP4SDQ=-209.543394\CCSD=-209.542
6866\CCSD(T)=-209.5887943\RMSD=5.700e-09\PG=C01 [X(C4H5N1)]\\@

T1 Diagnostic = 0.02104273

2-(1-oxacyclobutylidene)carbene (singlet 14)

1\1\GINC-NSCC-N2\SP\RCCSD(T)-FC\CC-pVTZ\C4H4O1\DMTHAMAT\17-Dec-2018\0\
\# rccsd=(t,t1diag)/cc-pvtz geom=connectivity\\Betalactone carbene sin
glet\\0,1\C,0,-1.332982,-0.042939,-0.000047\C,0,-0.269611,1.082539,0.0
00056\H,0,-1.946773,-0.119324,0.89771\H,0,-1.946713,-0.119216,-0.89785
3\H,0,-0.235293,1.699736,0.89533\H,0,-0.235234,1.699843,-0.895141\C,0,
0.699745,-0.114609,0.000016\C,0,2.013993,-0.020501,0.000065\O,0,-0.287
856,-1.073497,-0.000074\\Version=ES64L-G16RevA.03\State=1-A\HF=-228.55
09854\MP2=-229.3841222\MP3=-229.413458\MP4D=-229.4340852\MP4DQ=-229.41
19129\MP4SDQ=-229.4212644\CCSD=-229.420647\CCSD(T)=-229.4612941\RMSD=8
.239e-09\PG=C01 [X(C4H4O1)]\\@

T1 Diagnostic = 0.01588445

2-(1-oxacyclobutylidene)carbene (triplet 14)

1\1\GINC-NSCC-N11\SP\UCCSD(T)-FC\CC-pVTZ\C4H4O1(3)\DMTHAMAT\17-Dec-201
8\0\\# uccsd=(t,t1diag)/cc-pvtz geom=connectivity\\Betalactone carbene
triplet\\0,3\C,0,-1.345507,-0.173587,0.000125\C,0,-0.432338,1.062253,
-0.000023\H,0,-1.933009,-0.357673,0.898542\H,0,-1.933162,-0.357776,-0.
89817\H,0,-0.467891,1.690867,0.890728\H,0,-0.468043,1.690765,-0.890841
\C,0,0.691202,0.036198,-0.00006\C,0,2.08337,0.014458,-0.000182\O,0,-0.
147282,-1.037764,0.000073\\Version=ES64L-G16RevA.03\State=3-A\HF=-228.
5437085\MP2=-229.3506101\MP3=-229.3811329\MP4D=-229.4001667\MP4DQ=-229
.3797524\PUHF=-228.5469165\PMP2-0=-229.3526331\PMP3-0=-229.3822476\MP4
SDQ=-229.389621\CCSD=-229.388996\CCSD(T)=-229.4276439\S2=2.017582\S2-1
=2.004525\S2A=2.000136\RMSD=7.950e-09\PG=C01 [X(C4H4O1)]\\@

T1 Diagnostic = 0.02036099

2-(1-thiocyclobutylidene)carbene (singlet 16)

1\1\GINC-NSCC-N2\SP\RCCSD(T)-FC\CC-pVTZ\C4H4S1\DMTHAMAT\17-Dec-2018\0\
\# rccsd=(t,t1diag)/cc-pvtz geom=connectivity\\Beta thiolactone carben
e singlet\\0,1\C,0,1.274233,0.675772,0.082144\C,0,-0.115561,1.337602,-
0.067815\H,0,1.977873,0.88203,-0.721529\H,0,1.745059,0.841992,1.049276
\H,0,-0.244291,1.834817,-1.029873\H,0,-0.375653,2.031366,0.731421\C,0,
-0.918291,0.02843,-0.012782\C,0,-2.197671,-0.216116,0.067296\S,0,0.540
047,-1.034021,-0.027647\\Version=ES64L-G16RevA.03\State=1-A\HF=-551.23
69181\MP2=-552.0125845\MP3=-552.0553031\MP4D=-552.076663\MP4DQ=-552.05
25421\MP4SDQ=-552.0601524\CCSD=-552.0596781\CCSD(T)=-552.1018986\RMSD=
1.210e-09\PG=C01 [X(C4H4S1)]\\@

T1 Diagnostic = 0.01561639

2-(1-thiocyclobutylidene)carbene (triplet 16)

1\1\GINC-NSCC-N2\SP\UCCSD(T)-FC\CC-pVTZ\C4H4S1(3)\DMTHAMAT\17-Dec-2018
\0\\# uccsd=(t,t1diag)/cc-pvtz geom=connectivity\\Beta thiolactone car
bene triplet\\0,3\C,0,-1.338443,0.57332,-0.000017\C,0,-0.001286,1.3474
87,0.000038\H,0,-1.951143,0.693623,0.892082\H,0,-1.951099,0.693673,-0.
892139\H,0,0.153871,1.972746,0.883575\H,0,0.153915,1.972796,-0.883456\
C,0,0.897506,0.119115,0.000025\C,0,2.249548,-0.116506,0.000053\S,0,-0.
453094,-1.054584,-0.000041\\Version=ES64L-G16RevA.03\State=3-A\HF=-551
.2126042\MP2=-551.963628\MP3=-552.0084682\MP4D=-552.0278381\MP4DQ=-552
.0049926\PUHF=-551.2161134\PMP2-0=-551.9658542\PMP3-0=-552.0097256\MP4
SDQ=-552.0135874\CCSD=-552.0147656\CCSD(T)=-552.0562229\S2=2.021701\S2
-1=2.005714\S2A=2.000245\RMSD=6.305e-09\PG=C01 [X(C4H4S1)]\\@

T1 Diagnostic = 0.02735355

1-thiocyclopent-2-yne (17)

1\1\GINC-NSCC-N11\SP\RCCSD(T)-FC\CC-pVTZ\C4H4S1\DMTHAMAT\16-Dec-2018\0
\\# rccsd=(t,t1diag)/cc-pvtz geom=connectivity\\Thiacyclopentyne singl
et\\0,1\C,0,-0.502017,-1.127988,-0.147096\C,0,-1.572507,-0.021179,0.17
4536\H,0,-0.56969,-2.008018,0.492981\H,0,-0.586733,-1.426326,-1.191994
\H,0,-1.848766,-0.011929,1.23357\H,0,-2.466512,-0.161859,-0.433137\C,0
,-0.714973,1.123224,-0.194361\C,0,0.495718,1.346144,-0.019238\S,0,1.20
2148,-0.269567,0.063471\\Version=ES64L-G16RevA.03\State=1-A\HF=-551.22
0899\MP2=-552.0259986\MP3=-552.0558637\MP4D=-552.0777824\MP4DQ=-552.05
12278\MP4SDQ=-552.0625754\CCSD=-552.0633167\CCSD(T)=-552.1115175\RMSD=
8.137e-09\PG=C01 [X(C4H4S1)]\\@

T1 Diagnostic = 0.03131390

TS from singlet 16 to 17 by C shift

1\1\GINC-NSCC-N11\SP\RCCSD(T)-FC\CC-pVTZ\C4H4S1\DMTHAMAT\17-Dec-2018\0
\\# rccsd=(t,t1diag)/cc-pvtz geom=connectivity\\TS to thiacyclopentyne
 - C shift\\0,1\C,0,-0.238305,1.216177,-0.154523\C,0,1.206588,0.692006
,0.170922\H,0,-0.568041,1.999955,0.525324\H,0,-0.321056,1.547277,-1.18
8242\H,0,1.467388,0.856241,1.213124\H,0,1.931333,1.148938,-0.496141\C,
0,0.359677,-0.948957,-0.064079\C,0,1.618768,-1.002933,-0.111532\S,0,-1
.261874,-0.330636,0.056325\\Version=ES64L-G16RevA.03\State=1-A\HF=-551

.2030422\MP2=-552.0075516\MP3=-552.0373756\MP4D=-552.0593056\MP4DQ=-55
2.0327928\MP4SDQ=-552.0412655\CCSD=-552.0400812\CCSD(T)=-552.0867856\R
MSD=7.211e-09\PG=C01 [X(C4H4S1)]\\@

T1 Diagnostic = 0.01688364

TS from singlet 16 to 17 by S shift

1\1\GINC-NSCC-N11\SP\RCCSD(T)-FC\CC-pVTZ\C4H4S1\DMTHAMAT\17-Dec-2018\0
\\# rccsd=(t,t1diag)/cc-pvtz geom=connectivity\\TS to thiacyclopentyne
- S shift\\0,1\C,0,1.081714,-0.732482,0.340048\C,0,1.295263,0.697336,
-0.205571\H,0,1.650343,-1.519785,-0.151409\H,0,1.181851,-0.790742,1.42
3052\H,0,1.671724,0.681068,-1.231267\H,0,1.922394,1.351216,0.402773\C,
0,-0.148888,1.048298,-0.149667\C,0,-1.317717,1.256464,0.33426\S,0,-0.7
43034,-0.833716,-0.147348\\Version=ES64L-G16RevA.03\State=1-A\HF=-551.
2200207\MP2=-552.0189696\MP3=-552.0523079\MP4D=-552.0743661\MP4DQ=-552
.0476248\MP4SDQ=-552.0557994\CCSD=-552.0544611\CCSD(T)=-552.1006739\RM
SD=3.477e-09\PG=C01 [X(C4H4S1)]\\@

T1 Diagnostic = 0.01502545

2-(1-azacyclopentylidene)carbene (singlet 18)

1\1\GINC-NSCC-N2\SP\RCCSD(T)-FC\CC-pVTZ\C5H7N1\DMTHAMAT\17-Dec-2018\0\
\# rccsd=(t,t1diag)/cc-pvtz geom=connectivity\\glactamcarbene singlet\
\0,1\C,0,0.2044,1.195344,0.161591\C,0,-1.231296,0.779066,-0.231986\C,0
,-1.304101,-0.688357,0.192852\H,0,0.600761,2.007938,-0.444797\H,0,0.24
7917,1.492799,1.211681\H,0,-1.364493,0.859121,-1.313056\H,0,-1.98116,1
.402045,0.258903\H,0,-1.498995,-0.772025,1.272024\H,0,-2.070327,-1.253
851,-0.34191\C,0,0.96902,-0.138238,-0.023962\C,0,2.281767,-0.054648,-0
.028479\N,0,0.033934,-1.173259,-0.175839\H,0,0.31002,-2.08222,0.167936
\\Version=ES64L-G16RevA.03\State=1-A\HF=-247.8069312\MP2=-248.7938586\
MP3=-248.836815\MP4D=-248.8603836\MP4DQ=-248.8339601\MP4SDQ=-248.84285
22\CCSD=-248.8424175\CCSD(T)=-248.8903415\RMSD=7.786e-09\PG=C01 [X(C5H
7N1)]\\@

T1 Diagnostic = 0.01389395

2-(1-azacyclopentylidene)carbene (triplet 18)

1\1\GINC-NSCC-N14\SP\UCCSD(T)-FC\CC-pVTZ\C5H7N1(3)\DMTHAMAT\17-Dec-201
8\0\\# uccsd=(t,t1diag)/cc-pvtz geom=connectivity\\glactamcarbene trip
let\\0,3\C,0,0.045027,1.225097,0.150614\C,0,-1.353895,0.672782,-0.1984
66\C,0,-1.248776,-0.833629,0.113778\H,0,0.362309,2.05165,-0.484925\H,0
,0.098521,1.575327,1.18764\H,0,-1.553494,0.811625,-1.263735\H,0,-2.159
652,1.151535,0.359136\H,0,-1.566108,-1.066034,1.137759\H,0,-1.823335,-
1.461057,-0.570974\C,0,0.972026,0.025016,-0.000642\C,0,2.374473,-0.009
124,-0.076719\N,0,0.186676,-1.077713,-0.033684\H,0,0.601892,-1.9999,-0
.060512\\Version=ES64L-G16RevA.03\State=3-A\HF=-247.8043984\MP2=-248.7
644365\MP3=-248.8102782\MP4D=-248.8317489\MP4DQ=-248.806698\PUHF=-247.
8086284\PMP2-0=-248.7673308\PMP3-0=-248.8119781\MP4SDQ=-248.815376\CCS
D=-248.8150408\CCSD(T)=-248.8606881\S2=2.029776\S2-1=2.010804\S2A=2.00
0399\RMSD=4.123e-09\PG=C01 [X(C5H7N1)]\\@

T1 Diagnostic = 0.01531540

1-azacyclohex-2-yne (19)

1\1\GINC-NSCC-N15\SP\RCCSD(T)-FC\CC-pVTZ\C5H7N1\DMTHAMAT\17-Dec-2018\0
\\# rccsd=(t,t1diag)/cc-pvtz geom=connectivity\\Azacyclohexyne\\0,1\C,
0,1.580058,-0.170935,0.156117\C,0,0.676586,1.053652,-0.273213\C,0,-0.7
79149,1.016744,0.23834\H,0,2.468169,-0.212775,-0.479721\H,0,1.919726,-
0.07722,1.1922\H,0,0.665101,1.098587,-1.365342\H,0,1.135834,1.979992,0
.089496\H,0,-0.780592,1.094042,1.333545\H,0,-1.326897,1.870458,-0.1688
93\C,0,0.615421,-1.277972,-0.028928\C,0,-0.604451,-1.249022,-0.022979\
N,0,-1.515466,-0.235325,-0.186544\H,0,-2.40386,-0.340616,0.288502\\Ver
sion=ES64L-G16RevA.03\State=1-A\HF=-247.7883677\MP2=-248.8210163\MP3=-
248.8460601\MP4D=-248.8722314\MP4DQ=-248.8415963\MP4SDQ=-248.8521721\C
CSD=-248.8514561\CCSD(T)=-248.9056636\RMSD=5.201e-09\PG=C01 [X(C5H7N1)
]\\@

T1 Diagnostic = 0.01623650

TS from singlet 18 to 19 by C shift

1\1\GINC-NSCC-N16\SP\RCCSD(T)-FC\CC-pVTZ\C5H7N1\DMTHAMAT\17-Dec-2018\0
\\# rccsd=(t,t1diag)/cc-pvtz geom=connectivity\\TS to azacyclohexyne -
C shift\\0,1\C,0,0.737979,0.957792,0.416058\C,0,-0.646201,1.160078,-0
.213854\C,0,-1.465911,-0.147469,-0.098431\H,0,1.431537,1.735062,0.1137
62\H,0,0.727516,0.890648,1.504596\H,0,-0.512312,1.424842,-1.264627\H,0
,-1.165327,1.992311,0.272319\H,0,-2.144747,-0.132552,0.757383\H,0,-2.0
59545,-0.321852,-0.997857\C,0,0.765601,-0.789387,-0.060111\C,0,1.90794
3,-0.341991,-0.379353\N,0,-0.491281,-1.263379,0.096026\H,0,-0.634624,-
1.778942,0.956387\\Version=ES64L-G16RevA.03\State=1-A\HF=-247.7761267\
MP2=-248.7901068\MP3=-248.8221714\MP4D=-248.846271\MP4DQ=-248.8171309\
MP4SDQ=-248.826115\CCSD=-248.8247461\CCSD(T)=-248.8769008\RMSD=5.477e-
09\PG=C01 [X(C5H7N1)]\\@

T1 Diagnostic = 0.01181049

TS from singlet 18 to 19 by N shift

1\1\GINC-NSCC-N2\SP\RCCSD(T)-FC\CC-pVTZ\C5H7N1\DMTHAMAT\18-Dec-2018\0\
\# rccsd=(t,t1diag)/cc-pvtz geom=connectivity\\TS to azacyclohexyne -
N shift\\0,1\C,0,-0.155928,1.238032,-0.312836\C,0,-1.359253,0.407366,0
.148528\H,0,-0.065966,1.213999,-1.402152\H,0,-0.258398,2.285364,-0.009
833\H,0,-1.551275,0.595446,1.208914\H,0,-2.275043,0.640315,-0.403149\C
,0,0.473931,-1.084571,0.038405\C,0,1.670675,-0.722434,-0.196563\N,0,1.
1195,0.753654,0.236881\H,0,1.165125,0.843553,1.247601\C,0,-0.978441,-1
.113372,-0.021735\H,0,-1.311547,-1.487577,-0.994731\H,0,-1.4453,-1.716
803,0.760396\\Version=ES64L-G16RevA.03\State=1-A\HF=-247.7519843\MP2=-
248.7663994\MP3=-248.7984755\MP4D=-248.8234061\MP4DQ=-248.7938727\MP4S
DQ=-248.8053005\CCSD=-248.8041825\CCSD(T)=-248.8577619\RMSD=6.730e-09\
PG=C01 [X(C5H7N1)]\\@

T1 Diagnostic = 0.01822496

2-(1-oxacyclopentylidene)carbene (singlet 20)

1\1\GINC-NSCC-N17\SP\RCCSD(T)-FC\CC-pVTZ\C5H6O1\DMTHAMAT\17-Dec-2018\0
\\# rccsd=(t,t1diag)/cc-pvtz geom=connectivity\\glactonecarbene single
t\\0,1\C,0,0.20873,1.168605,0.241114\C,0,-1.189809,0.782153,-0.284006\
C,0,-1.291817,-0.686687,0.11579\H,0,0.657261,2.011134,-0.282346\H,0,0.
180302,1.394279,1.310766\H,0,-1.229901,0.887535,-1.37039\H,0,-1.977752
,1.395638,0.15736\H,0,-1.618221,-0.810566,1.153386\H,0,-1.924514,-1.28
938,-0.535501\O,0,0.060129,-1.206185,-0.003256\C,0,0.936563,-0.163299,
0.015253\C,0,2.241632,-0.090632,-0.156021\\Version=ES64L-G16RevA.03\St
ate=1-A\HF=-267.6362096\MP2=-268.6441399\MP3=-268.6819997\MP4D=-268.70
5934\MP4DQ=-268.679683\MP4SDQ=-268.6899217\CCSD=-268.6890633\CCSD(T)=-
268.7371711\RMSD=5.596e-09\PG=C01 [X(C5H6O1)]\\@

T1 Diagnostic = 0.01431870

2-(1-oxacyclopentylidene)carbene (triplet 20)

1\1\GINC-NSCC-N11\SP\UCCSD(T)-FC\CC-pVTZ\C5H6O1(3)\DMTHAMAT\17-Dec-201
8\0\\# uccsd=(t,t1diag)/cc-pvtz geom=connectivity\\glactonecarbene tri
plet\\0,3\C,0,0.043658,1.204342,0.164642\C,0,-1.343689,0.660443,-0.215
848\C,0,-1.209724,-0.825666,0.11959\H,0,0.386534,2.039961,-0.44531\H,0
,0.084074,1.530908,1.211188\H,0,-1.524422,0.79355,-1.284988\H,0,-2.161
266,1.132953,0.329935\H,0,-1.468319,-1.053522,1.157327\H,0,-1.749319,-
1.500969,-0.542505\O,0,0.2112,-1.119969,-0.038073\C,0,0.94828,0.001268
,0.002898\C,0,2.351995,-0.037575,-0.09146\\Version=ES64L-G16RevA.03\St
ate=3-A\HF=-267.624864\MP2=-268.6039126\MP3=-268.6444239\MP4D=-268.666
6204\MP4DQ=-268.6421805\PUHF=-267.6280305\PMP2-0=-268.605907\PMP3-0=-2
68.6455256\MP4SDQ=-268.6526278\CCSD=-268.6517545\CCSD(T)=-268.6974321\
S2=2.017281\S2-1=2.004428\S2A=2.000122\RMSD=5.331e-09\PG=C01 [X(C5H6O1
)]\\@

T1 Diagnostic = 0.01716699

1-oxacyclohex-2-yne (21)

1\1\GINC-NSCC-N12\SP\RCCSD(T)-FC\CC-pVTZ\C5H6O1\DMTHAMAT\17-Dec-2018\0
\\# rccsd=(t,t1diag)/cc-pvtz geom=connectivity\\oxacyclohexyne\\0,1\C,
0,1.562535,-0.068986,0.073561\C,0,0.567091,1.101713,-0.261643\C,0,-0.8
58798,0.932596,0.29684\H,0,2.335027,-0.124979,-0.697063\H,0,2.075094,0
.08701,1.027065\H,0,0.500782,1.211623,-1.347729\H,0,0.952709,2.046831,
0.137834\H,0,-0.865227,0.898822,1.388813\H,0,-1.528147,1.712854,-0.062
528\O,0,-1.507883,-0.344415,-0.163117\C,0,0.676547,-1.281327,0.142237\
C,0,-0.515238,-1.196801,-0.107905\\Version=ES64L-G16RevA.03\State=1-A\
HF=-267.6104986\MP2=-268.6651423\MP3=-268.6842269\MP4D=-268.7107043\MP
4DQ=-268.6802411\MP4SDQ=-268.6926509\CCSD=-268.6914683\CCSD(T)=-268.74
63496\RMSD=6.884e-09\PG=C01 [X(C5H6O1)]\\@

T1 Diagnostic = 0.01795460

TS from singlet 20 to 21 by C shift

1\1\GINC-NSCC-N13\SP\RCCSD(T)-FC\CC-pVTZ\C5H6O1\DMTHAMAT\17-Dec-2018\0
\\# rccsd=(t,t1diag)/cc-pvtz geom=connectivity\\TS to oxacyclohexyne -
C shift\\0,1\C,0,0.739143,1.084128,0.053499\C,0,-0.788227,1.073613,-0
.208302\C,0,-1.375583,-0.236407,0.303065\H,0,1.255051,1.661318,-0.7061
9\H,0,0.96815,1.459998,1.045763\H,0,-0.982061,1.151978,-1.280159\H,0,-
1.247248,1.935028,0.285218\H,0,-1.365603,-0.304823,1.394585\H,0,-2.365
99,-0.474787,-0.079681\O,0,-0.466031,-1.252557,-0.231326\C,0,0.743339,
-0.777006,-0.073899\C,0,1.925653,-0.379039,0.124149\\Version=ES64L-G16
RevA.03\State=1-A\HF=-267.6017502\MP2=-268.6382461\MP3=-268.6638764\MP
4D=-268.6883411\MP4DQ=-268.6592591\MP4SDQ=-268.6699417\CCSD=-268.66802
53\CCSD(T)=-268.7207067\RMSD=3.295e-09\PG=C01 [X(C5H6O1)]\\@

T1 Diagnostic = 0.01308362

TS from singlet 20 to 21 by O shift

1\1\GINC-NSCC-N2\SP\RCCSD(T)-FC\CC-pVTZ\C5H6O1\DMTHAMAT\18-Dec-2018\0\
\# rccsd=(t,t1diag)/cc-pvtz geom=connectivity\\TS to oxacyclohexyne -
O shift\\0,1\C,0,-1.081121,-1.016411,0.00308\C,0,-1.276397,0.558333,-0
.075936\C,0,0.043906,1.23655,0.280126\H,0,-1.431489,-1.392196,0.969706
\H,0,-1.636127,-1.51502,-0.793692\H,0,-2.092876,0.867593,0.581688\H,0,
-1.547508,0.806488,-1.103677\H,0,0.066258,2.27381,-0.064927\H,0,0.2399
88,1.210736,1.357218\O,0,1.112868,0.58724,-0.425022\C,0,0.358877,-1.15
2118,-0.080821\C,0,1.53787,-0.784576,0.282527\\Version=ES64L-G16RevA.0
3\State=1-A\HF=-267.5674117\MP2=-268.6011544\MP3=-268.6284907\MP4D=-26
8.6545315\MP4DQ=-268.6251949\MP4SDQ=-268.6394257\CCSD=-268.6370968\CCS
D(T)=-268.6913092\RMSD=5.220e-09\PG=C01 [X(C5H6O1)]\\@

T1 Diagnostic = 0.02045211

2-(1-thiocyclopentylidene)carbene (singlet 22)

1\1\GINC-NSCC-N11\SP\RCCSD(T)-FC\CC-pVTZ\C5H6S1\DMTHAMAT\18-Dec-2018\0
\\# rccsd=(t,t1diag)/cc-pvtz geom=connectivity\\gthiolactonecarbene si
nglet\\0,1\C,0,0.279663,1.388223,0.306835\C,0,-1.097512,1.144241,-0.32
7976\C,0,-1.537196,-0.257215,0.091951\H,0,0.781688,2.266961,-0.09973\H
,0,0.190741,1.513472,1.390478\H,0,-1.016404,1.200201,-1.416279\H,0,-1.
818762,1.899536,-0.000757\H,0,-1.918046,-0.274076,1.114291\H,0,-2.2838
93,-0.687436,-0.575405\C,0,1.063154,0.098951,0.02963\C,0,2.347015,0.16
8165,-0.200077\S,0,-0.016629,-1.323303,0.011576\\Version=ES64L-G16RevA
.03\State=1-A\HF=-590.3152526\MP2=-591.2688007\MP3=-591.318669\MP4D=-5
91.3436107\MP4DQ=-591.3152643\MP4SDQ=-591.323937\CCSD=-591.3230511\CCS
D(T)=-591.3730953\RMSD=7.874e-09\PG=C01 [X(C5H6S1)]\\@

T1 Diagnostic = 0.01399882

2-(1-thiocyclopentylidene)carbene (triplet 22)

1\1\GINC-NSCC-N12\SP\UCCSD(T)-FC\CC-pVTZ\C5H6S1(3)\DMTHAMAT\18-Dec-201
8\0\\# uccsd=(t,t1diag)/cc-pvtz geom=connectivity\\gthiolactonecarbene
triplet\\0,3\C,0,0.032576,1.431182,0.201616\C,0,-1.337113,0.943043,-0
.287294\C,0,-1.484619,-0.515669,0.161998\H,0,0.3866,2.31639,-0.32928\H
,0,-0.001669,1.690326,1.268349\H,0,-1.373793,0.991331,-1.379315\H,0,-2
.15675,1.553413,0.10071\H,0,-1.762771,-0.599433,1.215275\H,0,-2.191878
,-1.090009,-0.436087\C,0,1.027842,0.293706,0.026203\C,0,2.412047,0.362
026,-0.09146\S,0,0.199742,-1.246735,-0.031626\\Version=ES64L-G16RevA.0
3\State=3-A\HF=-590.2882822\MP2=-591.2148959\MP3=-591.2681295\MP4D=-59
1.2910146\MP4DQ=-591.2640849\PUHF=-590.2918981\PMP2-0=-591.2171871\PMP
3-0=-591.2694046\MP4SDQ=-591.2734169\CCSD=-591.2738618\CCSD(T)=-591.32
2483\S2=2.022321\S2-1=2.005848\S2A=2.000243\RMSD=6.376e-09\PG=C01 [X(C
5H6S1)]\\@

T1 Diagnostic = 0.02215782

1-thiocyclohex-2-yne (23)

1\1\GINC-NSCC-N13\SP\RCCSD(T)-FC\CC-pVTZ\C5H6S1\DMTHAMAT\18-Dec-2018\0
\\# rccsd=(t,t1diag)/cc-pvtz geom=connectivity\\thiocyclohexyne\\0,1\C
,0,1.855166,-0.371656,0.082828\C,0,1.135271,0.975635,-0.326434\C,0,-0.
232281,1.232663,0.326952\H,0,2.617098,-0.607676,-0.664775\H,0,2.3574,-
0.283064,1.050847\H,0,1.022047,0.985699,-1.413107\H,0,1.798,1.804445,-
0.054159\H,0,-0.146211,1.260312,1.415362\H,0,-0.636593,2.187728,-0.016
626\C,0,0.733687,-1.303649,0.118944\C,0,-0.476955,-1.354778,-0.025667\
S,0,-1.568817,-0.026046,-0.08608\\Version=ES64L-G16RevA.03\State=1-A\H
F=-590.3145895\MP2=-591.3071696\MP3=-591.3405571\MP4D=-591.3673101\MP4
DQ=-591.3357225\MP4SDQ=-591.3459846\CCSD=-591.3446673\CCSD(T)=-591.399
6455\RMSD=3.688e-09\PG=C01 [X(C5H6S1)]\\@

T1 Diagnostic = 0.01599080

TS from singlet 22 to 23 by C shift

1\1\GINC-NSCC-N14\SP\RCCSD(T)-FC\CC-pVTZ\C5H6S1\DMTHAMAT\18-Dec-2018\0
\\# rccsd=(t,t1diag)/cc-pvtz geom=connectivity\\TS to thiacyclohexyne
- C shift\\0,1\C,0,1.555113,0.407518,-0.02305\C,0,0.425133,1.422229,-0
.297628\C,0,-0.872877,1.083305,0.428547\H,0,2.295178,0.44553,-0.816004
\H,0,2.015249,0.593332,0.942758\H,0,0.225983,1.473146,-1.370191\H,0,0.
790115,2.407266,0.017842\H,0,-0.755358,1.072751,1.513209\H,0,-1.701766
,1.737747,0.157324\C,0,0.345555,-1.079808,0.005542\C,0,1.538565,-1.442
225,0.20085\S,0,-1.301146,-0.629743,-0.145657\\Version=ES64L-G16RevA.0
3\State=1-A\HF=-590.2749995\MP2=-591.2555264\MP3=-591.2936248\MP4D=-59
1.3193875\MP4DQ=-591.2883123\MP4SDQ=-591.2974993\CCSD=-591.2959058\CCS
D(T)=-591.3504395\RMSD=4.940e-09\PG=C01 [X(C5H6S1)]\\@

T1 Diagnostic = 0.01397042

TS from singlet 22 to 23 by S shift

1\1\GINC-NSCC-N15\SP\RCCSD(T)-FC\CC-pVTZ\C5H6S1\DMTHAMAT\18-Dec-2018\0
\\# rccsd=(t,t1diag)/cc-pvtz geom=connectivity\\TS to thiocyclohexyne
- S shift\\0,1\C,0,-1.40838446,-0.8069775,-0.39080484\C,0,-1.49223073,
0.57160674,0.32731008\C,0,-0.22532306,1.39945401,0.07188385\H,0,-2.171
2838,-1.49429418,-0.01253094\H,0,-1.54567317,-0.71470903,-1.47259622\H
,0,-1.59448144,0.38314895,1.39736526\H,0,-2.37813438,1.1233123,-0.0015
4521\H,0,-0.30733969,1.98055966,-0.84814801\H,0,-0.03267735,2.07933114
,0.90157957\C,0,-0.07947153,-1.29143832,-0.06303666\C,0,1.00934252,-1.
38250377,0.57855584\S,0,1.32537459,0.35636276,-0.19422338\\Version=ES6
4L-G16RevA.03\State=1-A\HF=-590.290542\MP2=-591.270049\MP3=-591.309393
8\MP4D=-591.3347702\MP4DQ=-591.3035279\MP4SDQ=-591.3137755\CCSD=-591.3
124824\CCSD(T)=-591.3671191\RMSD=4.350e-09\PG=C01 [X(C5H6S1)]\\@

T1 Diagnostic = 0.01738642

2-(1-azacyclohexylidene)carbene (singlet 24)

1\1\GINC-NSCC-N11\SP\RCCSD(T)-FC\CC-pVTZ\C6H9N1\DMTHAMAT\18-Dec-2018\0
\\# rccsd=(t,t1diag)/cc-pvtz geom=connectivity\\delta lactamcarbene si
nglet\\0,1\C,0,1.386189,0.762943,-0.324839\C,0,0.175223,1.515189,0.285
543\C,0,0.143944,-1.474232,-0.192023\C,0,1.446591,-0.715233,0.113989\H
,0,0.348069,1.695993,1.354259\H,0,0.036988,2.486822,-0.194014\H,0,1.30
6902,0.810254,-1.416149\H,0,2.315915,1.268149,-0.04746\H,0,0.167473,-2
.489845,0.211587\H,0,-0.022207,-1.542856,-1.271325\H,0,1.642946,-0.769
358,1.192258\H,0,2.284007,-1.215079,-0.383195\C,0,-1.02163,0.663841,0.
093414\C,0,-2.091772,0.264263,-0.550174\N,0,-0.999998,-0.756068,0.3852
41\H,0,-1.311382,-1.05223,1.301886\\Version=ES64L-G16RevA.03\State=1-A
\HF=-286.8502414\MP2=-288.0366921\MP3=-288.0804117\MP4D=-288.1084276\M
P4DQ=-288.0759149\MP4SDQ=-288.0863077\CCSD=-288.0845306\CCSD(T)=-288.1
432209\RMSD=8.917e-09\PG=C01 [X(C6H9N1)]\\@

T1 Diagnostic = 0.01208762

2-(1-azacyclohexylidene)carbene (triplet 24)

1\1\GINC-NSCC-N13\SP\UCCSD(T)-FC\CC-pVTZ\C6H9N1(3)\DMTHAMAT\18-Dec-201
8\0\\# uccsd=(t,t1diag)/cc-pvtz geom=connectivity\\delta lactamcarbene
triplet\\0,3\C,0,1.035348,1.197627,-0.320291\C,0,-0.428323,1.310263,0
.12683\C,0,0.951899,-1.301688,-0.123882\C,0,1.684293,-0.035814,0.31433
\H,0,-0.472183,1.633198,1.175152\H,0,-0.969193,2.069462,-0.441802\H,0,
1.085845,1.116901,-1.412622\H,0,1.58104,2.10426,-0.045623\H,0,1.249762
,-2.162594,0.48092\H,0,1.197781,-1.536258,-1.168283\H,0,1.649481,0.050
17,1.406094\H,0,2.736659,-0.122482,0.031416\C,0,-1.206696,0.01283,0.02
7461\C,0,-2.615298,-0.045129,-0.003426\N,0,-0.501471,-1.143575,-0.0094
14\H,0,-1.076228,-1.976163,-0.085485\\Version=ES64L-G16RevA.03\State=3
-A\HF=-286.8538023\MP2=-287.9902034\MP3=-288.0455885\MP4D=-288.0707685
\MP4DQ=-288.0413448\PUHF=-286.8581639\PMP2-0=-287.993211\PMP3-0=-288.0
47365\MP4SDQ=-288.0512568\CCSD=-288.0507362\CCSD(T)=-288.1042513\S2=2.
03137\S2-1=2.011714\S2A=2.000435\RMSD=4.612e-09\PG=C01 [X(C6H9N1)]\\@

T1 Diagnostic = 0.01471102

1-azacyclohepty-2-yne (25)

1\1\GINC-NSCC-N11\SP\RCCSD(T)-FC\CC-pVTZ\C6H9N1\DMTHAMAT\18-Dec-2018\0
\\# rccsd=(t,t1diag)/cc-pvtz geom=connectivity\\azacycloheptyne\\0,1\C
,0,1.29702,0.89005,0.265903\C,0,1.820843,-0.547173,-0.051332\C,0,-1.37
6589,0.797924,0.242507\C,0,-0.05285,1.293256,-0.400929\H,0,2.274267,-0
.576069,-1.049409\H,0,2.598295,-0.832366,0.663179\H,0,1.190326,0.98502
4,1.351783\H,0,2.062717,1.611918,-0.040075\H,0,-2.198379,1.433846,-0.0
99617\H,0,-1.297423,0.902626,1.335154\H,0,-0.049936,1.015094,-1.460682
\H,0,-0.103477,2.386743,-0.371722\C,0,0.607041,-1.364519,0.008718\C,0,
-0.603577,-1.366565,-0.018213\N,0,-1.721343,-0.599335,-0.12699\H,0,-2.
578322,-0.949311,0.280392\\Version=ES64L-G16RevA.03\State=1-A\HF=-286.
8724157\MP2=-288.0737046\MP3=-288.1100456\MP4D=-288.1386848\MP4DQ=-288
.1051164\MP4SDQ=-288.1157861\CCSD=-288.1143694\CCSD(T)=-288.1741402\RM
SD=3.576e-09\PG=C01 [X(C6H9N1)]\\@

T1 Diagnostic = 0.01173363

TS from singlet 24 to 25 by C shift

1\1\GINC-NSCC-N14\SP\RCCSD(T)-FC\CC-pVTZ\C6H9N1\DMTHAMAT\18-Dec-2018\0
\\# rccsd=(t,t1diag)/cc-pvtz geom=connectivity\\TS to Azacycloheptyne
- C shift\\0,1\C,0,0.247146,1.464417,-0.237918\C,0,-1.056425,0.955953,
0.384575\C,0,1.371456,-0.826339,-0.285236\C,0,1.494106,0.642215,0.1302
29\H,0,-1.022121,0.891817,1.474431\H,0,-1.877012,1.613562,0.116338\H,0
,0.126837,1.493228,-1.32653\H,0,0.391701,2.500801,0.089025\H,0,2.22657
6,-1.410148,0.061762\H,0,1.333671,-0.911003,-1.375404\H,0,1.661842,0.6
99686,1.213477\H,0,2.37669,1.078504,-0.348742\C,0,-2.13963,-0.427805,-
0.441272\C,0,-1.001145,-0.798575,-0.0217\N,0,0.150453,-1.463327,0.2451
42\H,0,0.2356,-1.772353,1.207576\\Version=ES64L-G16RevA.03\State=1-A\H
F=-286.8323844\MP2=-288.0208816\MP3=-288.0628557\MP4D=-288.090696\MP4D
Q=-288.0574422\MP4SDQ=-288.0676021\CCSD=-288.0659597\CCSD(T)=-288.1257
135\RMSD=6.563e-09\PG=C01 [X(C6H9N1)]\\@

T1 Diagnostic = 0.01153346

TS from singlet 24 to 25 by N shift

1\1\GINC-NSCC-N15\SP\RCCSD(T)-FC\CC-pVTZ\C6H9N1\DMTHAMAT\18-Dec-2018\0
\\# rccsd=(t,t1diag)/cc-pvtz geom=connectivity\\TS to azacycloheptyne
- N shift\\0,1\C,0,-1.616743,0.014073,-0.285603\C,0,-0.996429,-1.30754
1,0.232066\C,0,0.606148,1.355593,-0.23496\C,0,-0.872093,1.262394,0.206
204\H,0,-1.222897,-1.459079,1.294402\H,0,-1.417502,-2.164231,-0.307269
\H,0,-1.616656,-0.003671,-1.379915\H,0,-2.65987,0.054912,0.04087\H,0,1
.000822,2.328351,0.082027\H,0,0.674511,1.323497,-1.326509\H,0,-0.91916
2,1.312965,1.302387\H,0,-1.39236,2.152477,-0.164825\C,0,0.436187,-1.28
8809,-0.003315\C,0,1.631983,-1.100711,-0.37593\N,0,1.547174,0.360789,0
.276905\H,0,1.588588,0.319267,1.289723\\Version=ES64L-G16RevA.03\State
=1-A\HF=-286.8050951\MP2=-287.9945113\MP3=-288.0361022\MP4D=-288.06466
82\MP4DQ=-288.0310456\MP4SDQ=-288.0440291\CCSD=-288.042775\CCSD(T)=-28
8.1040225\RMSD=4.229e-09\PG=C01 [X(C6H9N1)]\\@

T1 Diagnostic = 0.01841911

2-(1-oxacyclohexylidene)carbene (singlet 26)

1\1\GINC-NSCC-N11\SP\RCCSD(T)-FC\CC-pVTZ\C6H8O1\DMTHAMAT\18-Dec-2018\0
\\# rccsd=(t,t1diag)/cc-pvtz geom=connectivity\\delta lactonecarbene s
inglet\\0,1\C,0,-0.816076,1.302404,0.297136\C,0,0.576478,1.205857,-0.3
46791\C,0,-0.94292,-1.213091,0.231038\C,0,-1.664106,0.087618,-0.10469\
H,0,0.509672,1.314928,-1.435715\H,0,1.246592,1.987477,0.020832\H,0,-0.
711535,1.342291,1.386826\H,0,-1.300627,2.231946,-0.01583\H,0,-1.462114
,-2.083319,-0.170118\H,0,-0.838937,-1.333543,1.316334\H,0,-1.871256,0.
118287,-1.180133\H,0,-2.629259,0.098968,0.411387\C,0,1.147467,-0.18335
2,-0.064438\C,0,2.371318,-0.115534,0.411211\O,0,0.378062,-1.272556,-0.
359297\\Version=ES64L-G16RevA.03\State=1-A\HF=-306.6881395\MP2=-307.87
15373\MP3=-307.9189167\MP4D=-307.946481\MP4DQ=-307.9160397\MP4SDQ=-307
.9272522\CCSD=-307.9262086\CCSD(T)=-307.9819391\RMSD=7.916e-09\PG=C01
[X(C6H8O1)]\\@

T1 Diagnostic = 0.01356764

2-(1-oxacyclohexylidene)carbene (triplet 26)

1\1\GINC-NSCC-N2\SP\UCCSD(T)-FC\CC-pVTZ\C6H8O1(3)\DMTHAMAT\18-Dec-2018
\0\\# uccsd=(t,t1diag)/cc-pvtz geom=connectivity\\delta lactonecarbene
triplet\\0,3\C,0,-1.052157,1.195539,0.295422\C,0,0.425268,1.285633,-0
.102636\C,0,-0.911444,-1.283193,0.154753\C,0,-1.664178,-0.058516,-0.32
9719\H,0,0.509271,1.622995,-1.14549\H,0,0.968678,2.027642,0.48657\H,0,
-1.144319,1.144155,1.386239\H,0,-1.58213,2.095569,-0.025693\H,0,-1.177
491,-2.188223,-0.391007\H,0,-1.085326,-1.458261,1.221152\H,0,-1.617911
,0.002455,-1.422686\H,0,-2.717585,-0.172022,-0.057172\C,0,1.192144,-0.
014965,-0.020926\C,0,2.603138,-0.060438,-0.005737\O,0,0.536273,-1.1823
33,0.000143\\Version=ES64L-G16RevA.03\State=3-A\HF=-306.6685271\MP2=-3
07.8226128\MP3=-307.8729733\MP4D=-307.8988051\MP4DQ=-307.8701031\PUHF=
-306.6717125\PMP2-0=-307.8246293\PMP3-0=-307.874095\MP4SDQ=-307.881692
9\CCSD=-307.8806563\CCSD(T)=-307.9340488\S2=2.017552\S2-1=2.004609\S2A
=2.000124\RMSD=9.825e-09\PG=C01 [X(C6H8O1)]\\@

T1 Diagnostic = 0.01634918

1-oxacyclohepty-2-yne (27)

1\1\GINC-NSCC-N12\SP\RCCSD(T)-FC\CC-pVTZ\C6H8O1\DMTHAMAT\18-Dec-2018\0
\\# rccsd=(t,t1diag)/cc-pvtz geom=connectivity\\oxacycloheptyne\\0,1\C
,0,1.254706,0.928118,0.25966\C,0,1.815256,-0.48662,-0.072224\C,0,-1.41
2868,0.724337,0.246477\C,0,-0.117331,1.277527,-0.390804\H,0,2.21063,-0
.511159,-1.0942\H,0,2.64392,-0.735007,0.596198\H,0,1.155136,1.013704,1
.3473\H,0,1.98651,1.683894,-0.047532\H,0,-2.284366,1.263944,-0.124766\
H,0,-1.372052,0.800834,1.337664\H,0,-0.109915,1.025641,-1.457287\H,0,-
0.232055,2.366675,-0.339916\C,0,0.641569,-1.372331,0.056753\C,0,-0.560
614,-1.303704,-0.041541\O,0,-1.715264,-0.68906,-0.070924\\Version=ES64
L-G16RevA.03\State=1-A\HF=-306.6967009\MP2=-307.9199194\MP3=-307.95063
14\MP4D=-307.9795323\MP4DQ=-307.9461456\MP4SDQ=-307.9582471\CCSD=-307.
9563858\CCSD(T)=-308.0165099\RMSD=4.760e-09\PG=C01 [X(C6H8O1)]\\@

T1 Diagnostic = 0.01240841

TS from singlet 26 to 27 by C shift

1\1\GINC-NSCC-N14\SP\RCCSD(T)-FC\CC-pVTZ\C6H8O1\DMTHAMAT\18-Dec-2018\0
\\# rccsd=(t,t1diag)/cc-pvtz geom=connectivity\\TS to Oxacycloheptyne
- C shift\\0,1\C,0,-0.161019,1.466679,0.249315\C,0,1.119092,0.908404,-
0.37649\C,0,-1.380751,-0.780221,0.262822\C,0,-1.44026,0.690425,-0.1159
65\H,0,1.067779,0.818418,-1.463664\H,0,1.963393,1.5438,-0.13212\H,0,-0
.034723,1.49469,1.336785\H,0,-0.275587,2.505093,-0.082362\H,0,-2.23469
3,-1.340988,-0.113428\H,0,-1.287355,-0.926643,1.342599\H,0,-1.624796,0
.769999,-1.193106\H,0,-2.302648,1.137976,0.388715\C,0,0.906818,-0.8832
27,0.011266\C,0,2.034851,-0.517665,0.4514\O,0,-0.217969,-1.413589,-0.3
72189\\Version=ES64L-G16RevA.03\State=1-A\HF=-306.656103\MP2=-307.8685
211\MP3=-307.9036187\MP4D=-307.9318521\MP4DQ=-307.8985816\MP4SDQ=-307.
9103698\CCSD=-307.9081985\CCSD(T)=-307.9685556\RMSD=4.431e-09\PG=C01 [
X(C6H8O1)]\\@

T1 Diagnostic = 0.01249507

TS from singlet 26 to 27 by O shift

1\1\GINC-NSCC-N15\SP\RCCSD(T)-FC\CC-pVTZ\C6H8O1\DMTHAMAT\18-Dec-2018\0
\\# rccsd=(t,t1diag)/cc-pvtz geom=connectivity\\TS to oxacycloheptyne
- O shift\\0,1\C,0,1.573416,0.212258,0.300239\C,0,1.147617,-1.179083,-
0.238604\C,0,-0.811647,1.236542,0.207922\C,0,0.668648,1.359463,-0.1807
91\H,0,1.402897,-1.305202,-1.297111\H,0,1.665054,-1.978109,0.307599\H,
0,1.569008,0.175083,1.393814\H,0,2.602455,0.403956,-0.017727\H,0,-1.35
6091,2.117438,-0.145988\H,0,-0.938085,1.160289,1.293476\H,0,0.730057,1
.447856,-1.271697\H,0,1.047403,2.300941,0.232377\C,0,-0.272663,-1.3475
26,-0.019452\C,0,-1.461,-1.185903,0.424793\O,0,-1.473616,0.137906,-0.4
32423\\Version=ES64L-G16RevA.03\State=1-A\HF=-306.6249929\MP2=-307.832
0899\MP3=-307.8695519\MP4D=-307.8990279\MP4DQ=-307.8658467\MP4SDQ=-307
.8811969\CCSD=-307.8788412\CCSD(T)=-307.9402518\RMSD=4.343e-09\PG=C01
[X(C6H8O1)]\\@

T1 Diagnostic = 0.01987614

2-(1-thiocyclohexylidene)carbene (singlet 28)

1\1\GINC-NSCC-N17\SP\RCCSD(T)-FC\CC-pVTZ\C6H8S1\DMTHAMAT\18-Dec-2018\0
\\# rccsd=(t,t1diag)/cc-pvtz geom=connectivity\\delta thiolactonecarbe
ne singlet\\0,1\C,0,-1.769392,0.42788,0.224105\C,0,-0.798463,1.469154,
-0.384235\C,0,-0.029578,-1.490827,0.406126\C,0,-1.412297,-1.03984,-0.0
85601\H,0,-0.88971,1.490589,-1.475194\H,0,-1.036436,2.469208,-0.011099
\H,0,-1.788076,0.571634,1.309525\H,0,-2.780503,0.629784,-0.142063\H,0,
0.157078,-2.536465,0.149272\H,0,0.086797,-1.368699,1.485606\H,0,-1.476
503,-1.214216,-1.165811\H,0,-2.166594,-1.68571,0.377884\C,0,0.589586,1
.119665,0.001964\C,0,1.586592,1.150055,0.835772\S,0,1.305954,-0.51079,
-0.407307\\Version=ES64L-G16RevA.03\State=1-A\HF=-629.367234\MP2=-630.
5199785\MP3=-630.5695467\MP4D=-630.5985523\MP4DQ=-630.5637012\MP4SDQ=-
630.5742035\CCSD=-630.5721538\CCSD(T)=-630.6335407\RMSD=3.664e-09\PG=C
01 [X(C6H8S1)]\\@

T1 Diagnostic = 0.01332793

2-(1-thiocyclohexylidene)carbene (triplet 28)

1\1\GINC-NSCC-N2\SP\UCCSD(T)-FC\CC-pVTZ\C6H8S1(3)\DMTHAMAT\18-Dec-2018
\0\\# uccsd=(t,t1diag)/cc-pvtz geom=connectivity\\deltathiolactone car
bene triplet\\0,3\C,0,1.195339,1.301881,-0.343148\C,0,-0.243874,1.5589
33,0.118624\C,0,1.19026,-1.227006,-0.121139\C,0,1.819223,0.082233,0.34
3333\H,0,-0.237604,1.878572,1.16999\H,0,-0.690631,2.387135,-0.438645\H
,0,1.21795,1.160461,-1.430283\H,0,1.794033,2.192332,-0.132324\H,0,1.52
4969,-2.077575,0.475833\H,0,1.435797,-1.429959,-1.166841\H,0,1.722895,
0.178061,1.43044\H,0,2.890948,0.033999,0.124632\C,0,-1.208154,0.394152
,0.026125\C,0,-2.596158,0.518765,0.008219\S,0,-0.662386,-1.256048,-0.0
14055\\Version=ES64L-G16RevA.03\State=3-A\HF=-629.3315423\MP2=-630.434
6986\MP3=-630.4975587\MP4D=-630.5241507\MP4DQ=-630.4927804\PUHF=-629.3
35332\PMP2-0=-630.4371093\PMP3-0=-630.4988888\MP4SDQ=-630.5033709\CCSD
=-630.5035718\CCSD(T)=-630.5602061\S2=2.023881\S2-1=2.006365\S2A=2.000
28\RMSD=8.080e-09\PG=C01 [X(C6H8S1)]\\@

T1 Diagnostic = 0.02068639

1-thiocyclohepty-2-yne (29)

1\1\GINC-NSCC-N12\SP\RCCSD(T)-FC\CC-pVTZ\C6H8S1\DMTHAMAT\18-Dec-2018\0
\\# rccsd=(t,t1diag)/cc-pvtz geom=connectivity\\thiocycloheptyne\\0,1\
C,0,1.763557,0.690549,0.242239\C,0,1.996011,-0.823764,-0.050955\C,0,-0
.837616,1.232745,0.290912\C,0,0.524933,1.338072,-0.441019\H,0,2.404056
,-0.954049,-1.060069\H,0,2.726224,-1.237086,0.650572\H,0,1.679852,0.82
5901,1.325381\H,0,2.660692,1.233547,-0.072845\H,0,-1.488535,2.058411,-
0.007843\H,0,-0.693366,1.289414,1.372519\H,0,0.42389,0.975937,-1.46917
3\H,0,0.732084,2.41102,-0.51732\C,0,0.668025,-1.41626,0.04998\C,0,-0.5
41763,-1.355774,-0.00781\S,0,-1.867736,-0.287281,-0.045081\\Version=ES
64L-G16RevA.03\State=1-A\HF=-629.3876734\MP2=-630.5516204\MP3=-630.595
6965\MP4D=-630.6254837\MP4DQ=-630.5904301\MP4SDQ=-630.6009363\CCSD=-63
0.5991374\CCSD(T)=-630.6603951\RMSD=4.307e-09\PG=C01 [X(C6H8S1)]\\@

T1 Diagnostic = 0.01239136

TS from singlet 28 to 29 by C shift

1\1\GINC-NSCC-N14\SP\RCCSD(T)-FC\CC-pVTZ\C6H8S1\DMTHAMAT\18-Dec-2018\0
\\# rccsd=(t,t1diag)/cc-pvtz geom=connectivity\\TS to thiacycloheptyne
- C shift\\0,1\C,0,1.383737,1.068951,0.124604\C,0,1.596655,-0.317693,
-0.473989\C,0,-1.198818,0.995565,0.455132\C,0,0.011328,1.720795,-0.135
084\H,0,1.294113,-0.400469,-1.518787\H,0,2.640325,-0.610036,-0.40916\H
,0,1.57925,1.017435,1.20086\H,0,2.15024,1.73006,-0.297634\H,0,-2.12340
6,1.526172,0.218609\H,0,-1.124979,0.88399,1.538348\H,0,-0.136965,1.835
78,-1.214724\H,0,0.026803,2.732498,0.286562\C,0,0.107876,-1.290364,0.1
57284\C,0,1.180939,-1.775987,0.619141\S,0,-1.42473,-0.695189,-0.267913
\\Version=ES64L-G16RevA.03\State=1-A\HF=-629.3302055\MP2=-630.4862915\
MP3=-630.5339759\MP4D=-630.563381\MP4DQ=-630.5281404\MP4SDQ=-630.53837
21\CCSD=-630.5364791\CCSD(T)=-630.5986391\RMSD=6.204e-09\PG=C01 [X(C6H
8S1)]\\@

T1 Diagnostic = 0.01302430

TS from singlet 28 to 29 by S shift

1\1\GINC-NSCC-N15\SP\RCCSD(T)-FC\CC-pVTZ\C6H8S1\DMTHAMAT\18-Dec-2018\0
\\# rccsd=(t,t1diag)/cc-pvtz geom=connectivity\\TS top thiocycloheptyn
e - S shift\\0,1\C,0,1.889632,0.040272,0.209017\C,0,1.267481,-1.275063
,-0.314362\C,0,-0.312753,1.409535,0.395665\C,0,1.108486,1.305477,-0.17
9412\H,0,1.344315,-1.337614,-1.406403\H,0,1.803843,-2.140642,0.091609\
H,0,1.96873,-0.020027,1.29933\H,0,2.907788,0.116272,-0.183838\H,0,-0.7
31621,2.395536,0.17433\H,0,-0.318856,1.280449,1.481032\H,0,1.058476,1.
391023,-1.270732\H,0,1.674357,2.17479,0.175711\C,0,-0.12585,-1.384328,
0.086241\C,0,-1.283957,-1.420194,0.582095\S,0,-1.560329,0.255377,-0.31
4781\\Version=ES64L-G16RevA.03\State=1-A\HF=-629.3506307\MP2=-630.5049
352\MP3=-630.5535221\MP4D=-630.5825469\MP4DQ=-630.5473684\MP4SDQ=-630.
5587816\CCSD=-630.5571853\CCSD(T)=-630.6191813\RMSD=6.624e-09\PG=C01 [
X(C6H8S1)]\\@

T1 Diagnostic = 0.01671983

Summary output of (R/U)CCSD(T)/cc-pVTZ//CCSD/6-311+G calculations**

2-(1-azacyclobutylidene)carbene (singlet 12)

Charge = 0 Multiplicity = 1
Redundant internal coordinates found in file.
C,0,1.3937124166,0.0406064948,0.2737463325
C,0,0.2927805273,1.0881630052,-0.0628756478
H,0,2.034105936,-0.1620721231,-0.5930240344
H,0,2.0025072284,0.2127454021,1.1649767558
H,0,0.450387434,1.7183976744,-0.937819743
H,0,-0.0143544242,1.6814063804,0.8013211735
C,0,-0.6029117571,-0.155732809,-0.2496112417
C,0,-1.7266047075,-0.21865819,-0.9495513973
N,0,0.3203078509,-0.97895688,0.46333425
H,0,0.4781837856,-1.9086020247,0.0889478827

Zero-point correction= 0.080515 (Hartree/Particle)
Thermal correction to Energy= 0.085330
Thermal correction to Enthalpy= 0.086274
Thermal correction to Gibbs Free Energy= 0.053032
Sum of electronic and zero-point Energies= -209.375261
Sum of electronic and thermal Energies= -209.370446
Sum of electronic and thermal Enthalpies= -209.369502
Sum of electronic and thermal Free Energies= -209.402744

2-(1-azacyclobutylidene)carbene (triplet 12)

Charge = 0 Multiplicity = 3
Redundant internal coordinates found in file.
C,0,1.38854623,-0.15332019,0.
C,0,0.42738827,1.07528184,0.
H,0,2.00005505,-0.27724234,-0.89864571
H,0,2.00005505,-0.27724234,0.89864571
H,0,0.46622202,1.69811961,-0.89651124
H,0,0.46622202,1.69811961,0.89651124
C,0,-0.71925931,0.03954787,0.
C,0,-2.12425373,0.04363259,0.
N,0,0.17407715,-0.98410361,0.
H,0,0.01434652,-1.98386784,0.

Zero-point correction= 0.079454 (Hartree/Particle)
Thermal correction to Energy= 0.084400
Thermal correction to Enthalpy= 0.085345
Thermal correction to Gibbs Free Energy= 0.050785
Sum of electronic and zero-point Energies= -209.349886
Sum of electronic and thermal Energies= -209.344939
Sum of electronic and thermal Enthalpies= -209.343995
Sum of electronic and thermal Free Energies= -209.378555

S**2 before annihilation 2.0290, after 2.0004

1-azacyclopent-2-yne (13)

Charge = 0 Multiplicity = 1
Redundant internal coordinates found in file.
C,0,-0.9447322867,-0.1506947145,0.513951684
C,0,-0.5370065731,1.1440609554,-0.3157189949
H,0,-0.9877722642,0.1139391761,1.5764021693
H,0,-1.8958191614,-0.6134533154,0.2247811341
H,0,-0.9922946824,2.0478126811,0.0990309548
H,0,-0.8154650866,1.0607172342,-1.373384989
C,0,0.9876868244,1.0436719972,-0.0662414985
C,0,1.1605193465,-0.2181241116,-0.1472309173
N,0,0.2179492446,-1.088012823,0.3180309513
H,0,0.0507326389,-1.9024230795,-0.2578994839

Zero-point correction= 0.080758 (Hartree/Particle)
Thermal correction to Energy= 0.085366
Thermal correction to Enthalpy= 0.086310
Thermal correction to Gibbs Free Energy= 0.053763
Sum of electronic and zero-point Energies= -209.366785
Sum of electronic and thermal Energies= -209.362177
Sum of electronic and thermal Enthalpies= -209.361232
Sum of electronic and thermal Free Energies= -209.393780

TS from singlet 12 to 13 by C shift

Charge = 0 Multiplicity = 1
Redundant internal coordinates found in file.
C,0,-0.3784278518,1.0010202073,-0.1227232957
H,0,-0.7261316528,1.4065224957,-1.0757384527
H,0,-0.405081323,1.774560257,0.6473015565
C,0,1.1858946356,-1.2969652371,0.5167879221
C,0,-0.0331893635,-0.9385829448,0.4399307048
C,0,1.0542282008,0.3272021495,-0.2293817271
H,0,1.3849929201,0.1936176478,-1.2591839859
H,0,1.7937115624,0.8559495465,0.3670441104
N,0,-1.1942951461,-0.1782474839,0.3210804377
H,0,-1.7838215216,-0.533196788,-0.4276128502
Zero-point correction= 0.079838 (Hartree/Particle)
Thermal correction to Energy= 0.084360
Thermal correction to Enthalpy= 0.085304
Thermal correction to Gibbs Free Energy= 0.052550
Sum of electronic and zero-point Energies= -209.362790
Sum of electronic and thermal Energies= -209.358268
Sum of electronic and thermal Enthalpies= -209.357324
Sum of electronic and thermal Free Energies= -209.390078

****** 1 imaginary frequencies (negative Signs) ****** -146.5757 cm^{-1}

TS from singlet 12 to 13 by N shift

Charge = 0 Multiplicity = 1
Redundant internal coordinates found in file.
C,0,0.825099146,-0.8461402598,0.182535972
C,0,1.1116689998,0.6602908549,-0.0044425748
H,0,1.3162488159,-1.4451960331,-0.587476017
H,0,1.0885320947,-1.2309702009,1.1702032087
H,0,1.7939994135,0.8922697779,-0.8242361771
H,0,1.4224485083,1.1772597597,0.9064067978
C,0,-1.5078461354,0.4325432952,0.0085650001
C,0,-0.3274485659,0.8681093706,-0.3312921909
N,0,-0.7034235931,-0.9397959602,0.0164751238
H,0,-0.931860304,-1.4404908145,-0.8395982128

Zero-point correction= 0.079296 (Hartree/Particle)
Thermal correction to Energy= 0.083727
Thermal correction to Enthalpy= 0.084671
Thermal correction to Gibbs Free Energy= 0.052314
Sum of electronic and zero-point Energies= -209.344029
Sum of electronic and thermal Energies= -209.339598
Sum of electronic and thermal Enthalpies= -209.338654
Sum of electronic and thermal Free Energies= -209.371011

****** 1 imaginary frequencies (negative Signs) ****** -218.7748 cm^{-1}

2-(1-oxacyclobutylidene)carbene (singlet 14)

Charge = 0 Multiplicity = 1
Redundant internal coordinates found in file.
C,0,1.3283097628,-0.0468871138,0.
C,0,0.2750462649,1.0915832717,0.
H,0,1.9419422524,-0.1239946237,-0.9005181713
H,0,1.9419422524,-0.1239946237,0.9005181713
H,0,0.2499920311,1.7089006226,-0.8989348468
H,0,0.2499920311,1.7089006226,0.8989348468
C,0,-0.6972815838,-0.0938073457,0.
C,0,-2.0305214287,-0.064657345,0.
O,0,0.2818935027,-1.064747908,0.

Zero-point correction= 0.067702 (Hartree/Particle)
Thermal correction to Energy= 0.072508
Thermal correction to Enthalpy= 0.073452
Thermal correction to Gibbs Free Energy= 0.039787
Sum of electronic and zero-point Energies= -229.224965
Sum of electronic and thermal Energies= -229.220159
Sum of electronic and thermal Enthalpies= -229.219215
Sum of electronic and thermal Free Energies= -229.252880

2-(1-oxacyclobutylidene)carbene (triplet 14)

Charge = 0 Multiplicity = 3
Redundant internal coordinates found in file.
C,0,0.1770749634,1.3419847222,0.
C,0,-1.0679631163,0.4339880481,0.
H,0,0.3562640831,1.9293207444,-0.901935317
H,0,0.3562640831,1.9293207444,0.901935317
H,0,-1.6915920427,0.4701742658,-0.8962720562
H,0,-1.6915920427,0.4701742658,0.8962720562
C,0,-0.0313915242,-0.6837659081,0.
C,0,-0.010192172,-2.0893701414,0.
O,0,1.0344443295,0.1494334014,0.

Zero-point correction= 0.067346 (Hartree/Particle)
Thermal correction to Energy= 0.071934
Thermal correction to Enthalpy= 0.072878
Thermal correction to Gibbs Free Energy= 0.039007
Sum of electronic and zero-point Energies= -229.195349
Sum of electronic and thermal Energies= -229.190760
Sum of electronic and thermal Enthalpies= -229.189816
Sum of electronic and thermal Free Energies= -229.223688

S**2 before annihilation 2.0175, after 2.0001

1-oxacyclopent-2-yne (15)

Charge = 0 Multiplicity = 1
Redundant internal coordinates found in file.
C,0,0.9907719822,-0.2197163,-0.1706516289
C,0,0.3907691947,1.2169820125,0.0981057073
H,0,1.0702923596,-0.3882247857,-1.250165951
H,0,1.9387027147,-0.4468882202,0.3243382722
H,0,0.7837301917,1.9485195818,-0.6130110082
H,0,0.5887263074,1.5634824668,1.1181523001
C,0,-1.0836824791,0.8330462212,-0.129976167
C,0,-1.1031455041,-0.3570820151,0.278279497
O,0,-0.0352267671,-1.1585329715,0.3447949785

Zero-point correction= 0.068257 (Hartree/Particle)
Thermal correction to Energy= 0.072693
Thermal correction to Enthalpy= 0.073637
Thermal correction to Gibbs Free Energy= 0.041359
Sum of electronic and zero-point Energies= -229.205347
Sum of electronic and thermal Energies= -229.200912
Sum of electronic and thermal Enthalpies= -229.199967
Sum of electronic and thermal Free Energies= -229.232246

TS from singlet 14 to 15 by C shift

Charge = 0 Multiplicity = 1
Redundant internal coordinates found in file.
C,0,0.9398406871,0.6232635029,-0.0650607193
C,0,-0.5836017453,1.0264361151,0.0526490858
H,0,1.5291244717,1.175942762,-0.7995144154
H,0,1.4224205782,0.6273726546,0.9165391661
H,0,-0.9836996315,1.4210372786,-0.8851800368
H,0,-0.731009857,1.7596148124,0.8492195696
C,0,-0.3685108587,-0.9629793107,-0.118179429
C,0,-1.3026017135,-0.3535558238,0.4683745102
O,0,0.879262069,-0.8006819913,-0.5135747311

Zero-point correction= 0.066897 (Hartree/Particle)
Thermal correction to Energy= 0.071136
Thermal correction to Enthalpy= 0.072080
Thermal correction to Gibbs Free Energy= 0.040053
Sum of electronic and zero-point Energies= -229.204788
Sum of electronic and thermal Energies= -229.200549
Sum of electronic and thermal Enthalpies= -229.199605
Sum of electronic and thermal Free Energies= -229.231632

****** 1 imaginary frequencies (negative Signs) ****** -328.9302 cm^{-1}

TS from singlet 14 to 15 by O shift

Charge = 0 Multiplicity = 1
Redundant internal coordinates found in file.
C,0,0.9691446982,0.5944855923,-0.0184395849
C,0,0.9559735357,-0.9509303426,0.0621235806
H,0,1.5832177374,1.0721015295,0.7455947962
H,0,1.2328557496,0.9585647171,-1.0159430851
H,0,1.4272157068,-1.3566120159,0.9593024202
H,0,1.3336877248,-1.4539919543,-0.8315290932
C,0,-1.4055571701,-0.0499503824,-0.3613930736
C,0,-0.5320157554,-0.9093200942,0.1386605726
O,0,-0.4380267871,0.9722371004,0.258375547

Zero-point correction= 0.065625 (Hartree/Particle)
Thermal correction to Energy= 0.070067
Thermal correction to Enthalpy= 0.071012
Thermal correction to Gibbs Free Energy= 0.038581
Sum of electronic and zero-point Energies= -229.175866
Sum of electronic and thermal Energies= -229.171423
Sum of electronic and thermal Enthalpies= -229.170479
Sum of electronic and thermal Free Energies= -229.202909

****** 1 imaginary frequencies (negative Signs) ****** -316.2697 cm^{-1}

2-(1-thiocyclobutylidene)carbene (singlet 16)

Charge = 0 Multiplicity = 1
Redundant internal coordinates found in file.
C,0,0.9595252334,2.7880362551,0.0648817568
C,0,-0.5837070488,2.7934566287,-0.081405909
H,0,1.5020252576,3.397159566,-0.6590884701
H,0,1.2888013291,3.0128816887,1.0808566085
H,0,-0.8976817404,3.1591899821,-1.0629672678
H,0,-1.1287129737,3.3210161753,0.7047112396
C,0,-0.7025983106,1.2635187042,-0.0213233754
C,0,-1.7349352316,0.4899125648,0.25095016
S,0,1.046071255,0.9712876751,-0.2741208426

Zero-point correction= 0.065111 (Hartree/Particle)
Thermal correction to Energy= 0.070271
Thermal correction to Enthalpy= 0.071215
Thermal correction to Gibbs Free Energy= 0.036364
Sum of electronic and zero-point Energies= -551.858042
Sum of electronic and thermal Energies= -551.852882
Sum of electronic and thermal Enthalpies= -551.851937
Sum of electronic and thermal Free Energies= -551.886789

2-(1-thiocyclobutylidene)carbene (triplet 16)

Charge = 0 Multiplicity = 3
Redundant internal coordinates found in file.
C,0,1.3386360556,0.5672826178,0.
C,0,0.0035385106,1.3546411344,0.
H,0,1.9481571912,0.6941890035,-0.8959817532
H,0,1.9481571912,0.6941890035,0.8959817532
H,0,-0.1518905683,1.973495116,-0.8898900497
H,0,-0.1518905683,1.973495116,0.8898900497
C,0,-0.8794451021,0.1075815319,0.
C,0,-2.2543676151,-0.1211913945,0.
S,0,0.440197026,-1.0423992815,0.

Zero-point correction= 0.064239 (Hartree/Particle)
Thermal correction to Energy= 0.069288
Thermal correction to Enthalpy= 0.070232
Thermal correction to Gibbs Free Energy= 0.034622
Sum of electronic and zero-point Energies= -551.816672
Sum of electronic and thermal Energies= -551.811623
Sum of electronic and thermal Enthalpies= -551.810679
Sum of electronic and thermal Free Energies= -551.846289

S**2 before annihilation 2.0215, after 2.0002

1-thiocyclopent-2-yne (17)

Charge = 0 Multiplicity = 1
Redundant internal coordinates found in file.
C,0,0.980166349,0.3538762475,0.1089205317
C,0,0.0257257284,1.5748177276,-0.1380438279
H,0,1.8784630989,0.3632642921,-0.5164640743
H,0,1.2742859015,0.3431434615,1.1628371333
H,0,-0.0132501712,1.8515230742,-1.1979564886

H,0,0.342650932,2.4382161866,0.451905657
C,0,-1.2601090602,0.9296784626,0.2929669461
C,0,-1.4883474131,-0.2778868422,0.1129120309
S,0,-0.0364273553,-1.1977216097,-0.2089189183

Zero-point correction= 0.066046 (Hartree/Particle)
Thermal correction to Energy= 0.070949
Thermal correction to Enthalpy= 0.071893
Thermal correction to Gibbs Free Energy= 0.037994
Sum of electronic and zero-point Energies= -551.858937
Sum of electronic and thermal Energies= -551.854034
Sum of electronic and thermal Enthalpies= -551.853090
Sum of electronic and thermal Free Energies= -551.886989

TS from singlet 16 to 17 by C shift

Charge = 0 Multiplicity = 1
Redundant internal coordinates found in file.
C,0,0.2358255915,1.1987191627,0.1559328055
C,0,-1.2127201294,0.6887642353,-0.1756889493
H,0,0.5487820655,2.0136888347,-0.4996494396
H,0,0.3084327202,1.4992874479,1.2030612437
H,0,-1.4524911687,0.8323168261,-1.2299475975
H,0,-1.9366477723,1.1872581863,0.4675216991
C,0,-0.3399994159,-0.9618544725,0.087775201
C,0,-1.6067212447,-0.9639109401,0.1464388004
S,0,1.2607572637,-0.3163764404,-0.1058588834

Zero-point correction= 0.064227 (Hartree/Particle)
Thermal correction to Energy= 0.068988
Thermal correction to Enthalpy= 0.069932
Thermal correction to Gibbs Free Energy= 0.036073
Sum of electronic and zero-point Energies= -551.837365
Sum of electronic and thermal Energies= -551.832605
Sum of electronic and thermal Enthalpies= -551.831661
Sum of electronic and thermal Free Energies= -551.865519

****** 1 imaginary frequencies (negative Signs) ****** -313.1815 cm^{-1}

TS from singlet 16 to 17 by S shift

Charge = 0 Multiplicity = 1
Redundant internal coordinates found in file.
C,0,1.0487358686,-0.7083242239,0.3330797552
C,0,1.3166898037,0.7117182621,-0.2107393658
H,0,1.6208640362,-1.5036036973,-0.1482847516
H,0,1.1517001371,-0.7512081862,1.4196069082
H,0,1.6761129817,0.6904639671,-1.2440852899
H,0,1.9666544907,1.3400741274,0.4032436428
C,0,-0.119265212,1.1133959028,-0.1410529492
C,0,-1.3104350431,1.1019933282,0.3401104611
S,0,-0.7591381729,-0.8205566002,-0.1401780207

Zero-point correction= 0.064455 (Hartree/Particle)
Thermal correction to Energy= 0.069172
Thermal correction to Enthalpy= 0.070116
Thermal correction to Gibbs Free Energy= 0.036435

Sum of electronic and zero-point Energies= -551.849306
Sum of electronic and thermal Energies= -551.844588
Sum of electronic and thermal Enthalpies= -551.843644
Sum of electronic and thermal Free Energies= -551.877325

****** 1 imaginary frequencies (negative Signs) ****** -200.3235 cm^{-1}

2-(1-azacyclopentylidene)carbene (singlet 18)

Charge = 0 Multiplicity = 1
Redundant internal coordinates found in file. (old form).
C,0,0.1967777493,1.1958873496,0.1675939333
C,0,-1.2340278228,0.7815512268,-0.2396375712
C,0,-1.2966173143,-0.6852440188,0.1920792247
H,0,0.5986610366,2.0171294618,-0.429727876
H,0,0.2250329276,1.4819539991,1.2247678127
H,0,-1.3540587853,0.8494979964,-1.3260477498
H,0,-1.995716521,1.4006064338,0.2437662935
H,0,-1.4494616398,-0.7576645239,1.2808464595
H,0,-2.0816991152,-1.2540797545,-0.3152466072
C,0,0.9622853315,-0.1277866529,-0.0282179563
C,0,2.29003941,-0.0916907322,-0.018017654
N,0,0.0287905944,-1.1743665706,-0.2224972079
H,0,0.3074696591,-2.0619925246,0.1754474687

Zero-point correction= 0.110763 (Hartree/Particle)
Thermal correction to Energy= 0.116412
Thermal correction to Enthalpy= 0.117356
Thermal correction to Gibbs Free Energy= 0.081664
Sum of electronic and zero-point Energies= -248.589628
Sum of electronic and thermal Energies= -248.583978
Sum of electronic and thermal Enthalpies= -248.583034
Sum of electronic and thermal Free Energies= -248.618727

2-(1-azacyclopentylidene)carbene (triplet 18)

Charge = 0 Multiplicity = 3
Redundant internal coordinates found in file. (old form).
C,0,0.1815577063,1.2138967636,0.1464261192
C,0,-1.2622284332,0.7996068819,-0.2069038225
C,0,-1.3224462115,-0.68312123,0.207483138
H,0,0.5900101004,1.9924638167,-0.5017007572
H,0,0.2567477935,1.5648517189,1.184445861
H,0,-1.4163141606,0.8800487671,-1.2887043448
H,0,-2.0230407341,1.4008576345,0.2983304614
H,0,-1.6305400998,-0.803543948,1.2551449114
H,0,-1.9802435792,-1.2868311848,-0.4248803648
C,0,0.971689692,-0.0843794994,0.0221948713
C,0,2.3725389973,-0.2732781844,-0.0812526464
N,0,0.0738459257,-1.0922908317,0.0441177471
H,0,0.3858985132,-2.0544790143,0.0504073965

Zero-point correction= 0.110700 (Hartree/Particle)
Thermal correction to Energy= 0.116226
Thermal correction to Enthalpy= 0.117170
Thermal correction to Gibbs Free Energy= 0.080900

Sum of electronic and zero-point Energies= -248.564331
Sum of electronic and thermal Energies= -248.558806
Sum of electronic and thermal Enthalpies= -248.557862
Sum of electronic and thermal Free Energies= -248.594131

S**2 before annihilation 2.0223, after 2.0002

1-azacyclohex-2-yne (19)

Charge = 0 Multiplicity = 1
Redundant internal coordinates found in file. (old form).
C,0,1.5807642018,-0.1525843815,0.1665430373
C,0,0.6753788903,1.0497134881,-0.284253037
C,0,-0.7739054103,1.0038193204,0.2521079503
H,0,2.4619641723,-0.2231632982,-0.4794536644
H,0,1.9257744115,-0.0346853051,1.2002752249
H,0,0.6365099551,1.0587395008,-1.380194153
H,0,1.1280849596,1.9949215386,0.0442243334
H,0,-0.7476145249,1.0166789081,1.3528866155
H,0,-1.3180574776,1.8881657319,-0.0978769062
C,0,0.6146198895,-1.2816617917,0.0289764144
C,0,-0.6114005751,-1.2437652925,-0.0428738165
N,0,-1.5177185329,-0.2077105596,-0.2361989544
H,0,-2.4034452192,-0.3200990591,0.2439330857

Zero-point correction= 0.111691 (Hartree/Particle)
Thermal correction to Energy= 0.117074
Thermal correction to Enthalpy= 0.118018
Thermal correction to Gibbs Free Energy= 0.083273
Sum of electronic and zero-point Energies= -248.597129
Sum of electronic and thermal Energies= -248.591747
Sum of electronic and thermal Enthalpies= -248.590803
Sum of electronic and thermal Free Energies= -248.625548

TS from singlet 18 to 19 by C shift

Charge = 0 Multiplicity = 1
Redundant internal coordinates found in file. (old form).
C,0,0.7475615713,0.9407619866,0.4133383559
C,0,-0.6398190442,1.1550734613,-0.2109149029
C,0,-1.4626410526,-0.1544622649,-0.1091864104
H,0,1.4265100398,1.7473917409,0.1437980208
H,0,0.727175894,0.8420267834,1.5031172111
H,0,-0.5007463074,1.4277801821,-1.2621758882
H,0,-1.1563357816,1.9847742494,0.2876385829
H,0,-2.1636552378,-0.1319205535,0.7317887418
H,0,-2.0313308586,-0.3350225854,-1.025842368
C,0,0.7567378128,-0.8048664529,-0.0774896295
C,0,1.8713086518,-0.3011715813,-0.4350339399
N,0,-0.5038102302,-1.2759888792,0.1172963582
H,0,-0.6204874572,-1.7096300865,1.0258858682

Zero-point correction= 0.109436 (Hartree/Particle)
Thermal correction to Energy= 0.114865
Thermal correction to Enthalpy= 0.115810
Thermal correction to Gibbs Free Energy= 0.080553
Sum of electronic and zero-point Energies= -248.571743

Sum of electronic and thermal Energies= -248.566314
Sum of electronic and thermal Enthalpies= -248.565369
Sum of electronic and thermal Free Energies= -248.600626

****** 1 imaginary frequencies (negative Signs) ****** -392.7320 cm^{-1}

TS from singlet 18 to 19 by N shift

Charge = 0 Multiplicity = 1
Redundant internal coordinates found in file. (old form).
C,0,-0.1570866268,1.222505905,-0.3287717371
C,0,-1.3490880686,0.4005676793,0.1747223141
H,0,-0.0747559487,1.1468820072,-1.4191543022
H,0,-0.2671591195,2.2822838366,-0.0664369304
H,0,-1.4767832239,0.5724566738,1.2506620689
H,0,-2.2891990315,0.6588637459,-0.3278199122
C,0,0.4654819385,-1.0863200617,0.0582355537
C,0,1.6765962789,-0.7146872079,-0.1248855781
N,0,1.1295130309,0.7758437762,0.2340604547
H,0,1.1469880512,0.8866332845,1.2453506638
C,0,-0.995892481,-1.1112410629,-0.0486711324
H,0,-1.3014274613,-1.4371415745,-1.0492372942
H,0,-1.4792263381,-1.7438970015,0.7016758314

Zero-point correction= 0.109206 (Hartree/Particle)
Thermal correction to Energy= 0.114449
Thermal correction to Enthalpy= 0.115393
Thermal correction to Gibbs Free Energy= 0.080790
Sum of electronic and zero-point Energies= -248.551632
Sum of electronic and thermal Energies= -248.546389
Sum of electronic and thermal Enthalpies= -248.545445
Sum of electronic and thermal Free Energies= -248.580048

****** 1 imaginary frequencies (negative Signs) ****** -485.4867 cm^{-1}

2-(1-oxacyclopentylidene)carbene (singlet 20)

Charge = 0 Multiplicity = 1
Redundant internal coordinates found in file. (old form).
C,0,0.3329615883,0.5437173261,0.19456463
C,0,1.7808518664,0.557218995,-0.3329133005
C,0,2.2399969076,1.9559194264,0.0742365761
H,0,-0.3214867767,-0.1596860708,-0.3241154026
H,0,0.3125403137,0.3271690024,1.2689842005
H,0,1.7887557641,0.4597088102,-1.4236877223
H,0,2.3970887449,-0.2337453831,0.1048374675
H,0,2.5372414812,1.9896515815,1.1303657549
H,0,3.0397042667,2.3680067268,-0.5448725243
O,0,1.0812766535,2.796174662,-0.1110300066
C,0,-0.0336628623,2.0087565762,-0.0482362623
C,0,-1.3169516674,2.3348830172,-0.2058335105

Zero-point correction= 0.098078 (Hartree/Particle)
Thermal correction to Energy= 0.103558
Thermal correction to Enthalpy= 0.104502
Thermal correction to Gibbs Free Energy= 0.069117
Sum of electronic and zero-point Energies= -268.438383

Sum of electronic and thermal Energies= -268.432904
Sum of electronic and thermal Enthalpies= -268.431960
Sum of electronic and thermal Free Energies= -268.467345

2-(1-oxacyclopentylidene)carbene (triplet 20)

Charge = 0 Multiplicity = 3
Redundant internal coordinates found in file. (old form).
C,0,0.0357740571,1.2025848759,0.1843072352
C,0,-1.3392248822,0.660534694,-0.2356722095
C,0,-1.2035209625,-0.8176618858,0.1318524542
H,0,0.3878540201,2.0610101251,-0.3914075842
H,0,0.0538047806,1.4767864906,1.2484646986
H,0,-1.4770022781,0.7685166806,-1.3169373591
H,0,-2.1771251143,1.1396742791,0.2776743772
H,0,-1.4347077152,-1.0066054596,1.1864241462
H,0,-1.76812566,-1.5061904894,-0.4987249489
O,0,0.2035960859,-1.1173290924,-0.0634890789
C,0,0.9377119811,-0.0015105131,-0.0155858756
C,0,2.3501949676,-0.033998385,-0.1390237955

Zero-point correction= 0.097807 (Hartree/Particle)
Thermal correction to Energy= 0.103147
Thermal correction to Enthalpy= 0.104092
Thermal correction to Gibbs Free Energy= 0.068106
Sum of electronic and zero-point Energies= -268.403409
Sum of electronic and thermal Energies= -268.398069
Sum of electronic and thermal Enthalpies= -268.397125
Sum of electronic and thermal Free Energies= -268.433111

S**2 before annihilation 2.0164, after 2.0001

1-oxacyclohex-2-yne (21)

Charge = 0 Multiplicity = 1
Redundant internal coordinates found in file. (old form).
C,0,1.568883899,-0.0549517774,0.0854787033
C,0,0.5670927751,1.0932306282,-0.2789031032
C,0,-0.8524153398,0.9159834992,0.3052737915
H,0,2.373849847,-0.1020262648,-0.6545533152
H,0,2.0281388714,0.0961202616,1.0688772168
H,0,0.4812754646,1.1530124859,-1.3706807255
H,0,0.9441521244,2.0604379678,0.0801831319
H,0,-0.8182375043,0.8619064891,1.3999129494
H,0,-1.5103900866,1.7282983277,-0.0115481925
O,0,-1.521293017,-0.3140817488,-0.166183752
C,0,0.67035251,-1.2620563168,0.0941907785
C,0,-0.5372072839,-1.2007017919,-0.1255344831

Zero-point correction= 0.098654 (Hartree/Particle)
Thermal correction to Energy= 0.103937
Thermal correction to Enthalpy= 0.104881
Thermal correction to Gibbs Free Energy= 0.070298
Sum of electronic and zero-point Energies= -268.439469
Sum of electronic and thermal Energies= -268.434186
Sum of electronic and thermal Enthalpies= -268.433242
Sum of electronic and thermal Free Energies= -268.467825

TS from singlet 20 to 21 by C shift

Charge = 0 Multiplicity = 1
Redundant internal coordinates found in file. (old form).
C,0,0.747136978,1.0781371975,0.0542272899
C,0,-0.7831255182,1.0706085245,-0.21100825
C,0,-1.3656029176,-0.242363134,0.3025660623
H,0,1.2592789498,1.6598877856,-0.7097105997
H,0,0.9568519387,1.4736743227,1.0472442276
H,0,-0.975889672,1.1347535043,-1.2867978983
H,0,-1.2439906221,1.9349899234,0.2803090441
H,0,-1.3157224307,-0.3214492472,1.3948428018
H,0,-2.3734897342,-0.4629687862,-0.0515500587
O,0,-0.4873458636,-1.2446568863,-0.2795773106
C,0,0.7256640851,-0.780392982,-0.0885533612
C,0,1.8947017568,-0.3587653823,0.1739694629

Zero-point correction= 0.097191 (Hartree/Particle)
Thermal correction to Energy= 0.102295
Thermal correction to Enthalpy= 0.103239
Thermal correction to Gibbs Free Energy= 0.068714
Sum of electronic and zero-point Energies= -268.416850
Sum of electronic and thermal Energies= -268.411747
Sum of electronic and thermal Enthalpies= -268.410803
Sum of electronic and thermal Free Energies= -268.445328

****** 1 imaginary frequencies (negative Signs) ****** -349.8254 cm^{-1}

TS from singlet 20 to 21 by O shift

Charge = 0 Multiplicity = 1
Redundant internal coordinates found in file. (old form).
C,0,-1.1712988171,-0.9259787525,0.0338378576
C,0,-1.2202278575,0.6456378073,-0.1023194548
C,0,0.1409412872,1.2144946345,0.2956510517
H,0,-1.5016273201,-1.2321675857,1.03311215
H,0,-1.7937426964,-1.4086618001,-0.7238162325
H,0,-2.0356009754,1.0493063846,0.5086827899
H,0,-1.4111996944,0.8905178292,-1.1518801931
H,0,0.2372105635,2.2630921511,-0.005339123
H,0,0.3148892977,1.1255092623,1.3758763257
O,0,1.1642768044,0.5173755886,-0.4227792738
C,0,0.262776176,-1.1806535403,-0.1078859241
C,0,1.4735786722,-0.8777577189,0.2485331563

Zero-point correction= 0.096173 (Hartree/Particle)
Thermal correction to Energy= 0.101244
Thermal correction to Enthalpy= 0.102188
Thermal correction to Gibbs Free Energy= 0.067928
Sum of electronic and zero-point Energies= -268.387174
Sum of electronic and thermal Energies= -268.382102
Sum of electronic and thermal Enthalpies= -268.381158
Sum of electronic and thermal Free Energies= -268.415419

****** 1 imaginary frequencies (negative Signs) ****** -503.6318 cm^{-1}

2-(1-thiocyclopentylidene)carbene (singlet 22)

Charge = 0 Multiplicity = 1
Redundant internal coordinates found in file. (old form).
C,0,0.3132738483,0.6005809207,0.2449659416
C,0,1.7881021786,0.568955982,-0.181241129
C,0,2.3513268435,1.9563286682,0.1337808661
H,0,-0.2503688975,-0.2630401308,-0.1193423927
H,0,0.2308893929,0.6404697841,1.3382214279
H,0,1.8532469965,0.3767648273,-1.2580149599
H,0,2.3405997367,-0.2151520518,0.3499674417
H,0,2.5432922669,2.068628359,1.2050369623
H,0,3.2676135656,2.1716588279,-0.4215011266
C,0,-0.2096049537,1.917361416,-0.3390381743
C,0,-1.4506951678,1.9656805233,-0.784174496
S,0,1.0606404598,3.1595375542,-0.3863604512

Zero-point correction= 0.094963 (Hartree/Particle)
Thermal correction to Energy= 0.100934
Thermal correction to Enthalpy= 0.101878
Thermal correction to Gibbs Free Energy= 0.064873
Sum of electronic and zero-point Energies= -591.067464
Sum of electronic and thermal Energies= -591.061493
Sum of electronic and thermal Enthalpies= -591.060549
Sum of electronic and thermal Free Energies= -591.097554

2-(1-thiocyclopentylidene)carbene (triplet 22)

Charge = 0 Multiplicity = 3
Redundant internal coordinates found in file. (old form).
C,0,0.3334858882,0.5719306863,0.2074421036
C,0,1.824104436,0.5457614504,-0.1531510183
C,0,2.3553701713,1.9599906859,0.1139524315
H,0,-0.228774317,-0.2619858756,-0.2218231407
H,0,0.1981427506,0.5416460494,1.2988157446
H,0,1.9376853003,0.3119331897,-1.2183710456
H,0,2.3809306012,-0.1991493858,0.4258412012
H,0,2.5111558381,2.1360132476,1.1836126971
H,0,3.2794752037,2.1841025221,-0.4241518008
C,0,-0.2231963776,1.8933562677,-0.3078464107
C,0,-1.5569711257,2.1881575705,-0.6501609688
S,0,1.0269079009,3.0760182717,-0.4718598831

Zero-point correction= 0.094459 (Hartree/Particle)
Thermal correction to Energy= 0.100215
Thermal correction to Enthalpy= 0.101160
Thermal correction to Gibbs Free Energy= 0.063853
Sum of electronic and zero-point Energies= -591.021617
Sum of electronic and thermal Energies= -591.015860
Sum of electronic and thermal Enthalpies= -591.014916
Sum of electronic and thermal Free Energies= -591.052223

S**2 before annihilation 2.0186, after 2.0002

1-thiocyclohex-2-yne (23)

Charge = 0 Multiplicity = 1
Redundant internal coordinates found in file. (old form).
C,0,1.8594064165,-0.3541182757,0.0909340401
C,0,1.1311964918,0.9653220504,-0.3442837974
C,0,-0.2310601008,1.2141540142,0.334832169
H,0,2.6449443469,-0.5979692694,-0.6315477099
H,0,2.3234012242,-0.253017585,1.0786436521
H,0,0.9880432835,0.9395565664,-1.4307811845
H,0,1.7838788149,1.8170880584,-0.1104666943
H,0,-0.1183564197,1.1965058242,1.4243442207
H,0,-0.6122304542,2.1970417717,0.0363856934
C,0,0.739219274,-1.3164827438,0.1098570547
C,0,-0.4802624113,-1.3205316649,-0.0331618782
S,0,-1.5703675759,0.0120639335,-0.1166704459

Zero-point correction= 0.095999 (Hartree/Particle)
Thermal correction to Energy= 0.101692
Thermal correction to Enthalpy= 0.102636
Thermal correction to Gibbs Free Energy= 0.066732
Sum of electronic and zero-point Energies= -591.085887
Sum of electronic and thermal Energies= -591.080195
Sum of electronic and thermal Enthalpies= -591.079250
Sum of electronic and thermal Free Energies= -591.115155

TS from singlet 22 to 23 by C shift

Charge = 0 Multiplicity = 1
Redundant internal coordinates found in file. (old form).
C,0,1.5601676551,0.3992718892,-0.0164985656
C,0,0.4243569452,1.4095146186,-0.3041910491
C,0,-0.8696868496,1.066222302,0.4327295556
H,0,2.3068938842,0.4540710555,-0.8073033848
H,0,2.0094313453,0.6142436023,0.9528523124
H,0,0.2178071003,1.4391920135,-1.3790720315
H,0,0.788609276,2.4004875659,-0.0006548627
H,0,-0.726829527,1.0383396991,1.5170031162
H,0,-1.6875720269,1.748275409,0.1851442505
C,0,0.325825563,-1.0716508752,-0.0132914445
C,0,1.5282052469,-1.3999470638,0.2224482994
S,0,-1.3174646225,-0.6069717062,-0.1756227158

Zero-point correction= 0.094012 (Hartree/Particle)
Thermal correction to Energy= 0.099574
Thermal correction to Enthalpy= 0.100518
Thermal correction to Gibbs Free Energy= 0.064545
Sum of electronic and zero-point Energies= -591.039678
Sum of electronic and thermal Energies= -591.034116
Sum of electronic and thermal Enthalpies= -591.033172
Sum of electronic and thermal Free Energies= -591.069145

****** 1 imaginary frequencies (negative Signs) ****** -414.6751 cm^{-1}

TS from singlet 22 to 23 by S shift

Charge = 0 Multiplicity = 1
Redundant internal coordinates found in file. (old form).
C,0,-1.4186615832,-0.7863679554,-0.4119410808
C,0,-1.4801393075,0.5594041193,0.3541393512
C,0,-0.2229671094,1.3845032048,0.0528402585
H,0,-2.1813737317,-1.487603439,-0.0551778683
H,0,-1.5422839993,-0.651949201,-1.4926664413
H,0,-1.528624382,0.3353144624,1.4243673077
H,0,-2.376531222,1.1278498328,0.0780754888
H,0,-0.3272035101,1.8916129902,-0.9115060527
H,0,-0.0654080101,2.1345824468,0.8322631296
C,0,-0.0808305072,-1.2871990338,-0.0851414898
C,0,0.9881651688,-1.3859726595,0.602264655
S,0,1.3355727837,0.3696056423,-0.0937501077

Zero-point correction= 0.094156 (Hartree/Particle)
Thermal correction to Energy= 0.099730
Thermal correction to Enthalpy= 0.100674
Thermal correction to Gibbs Free Energy= 0.064687
Sum of electronic and zero-point Energies= -591.053041
Sum of electronic and thermal Energies= -591.047467
Sum of electronic and thermal Enthalpies= -591.046522
Sum of electronic and thermal Free Energies= -591.082510

****** 1 imaginary frequencies (negative Signs) ****** -335.0619 cm^{-1}

2-(1-azacyclohexylidene)carbene (singlet 24)

Charge = 0 Multiplicity = 1
Redundant internal coordinates found in file. (old form).
C,0,1.4033315091,0.7224488409,-0.3620409507
C,0,0.2271374668,1.5264255917,0.24305742
C,0,0.1035787345,-1.4700642104,-0.1530756878
C,0,1.423878464,-0.7368573755,0.1391232343
H,0,0.412235155,1.7189706955,1.3096801094
H,0,0.1095499459,2.4918113999,-0.2599355428
H,0,1.2987390155,0.7242497984,-1.4554195014
H,0,2.3555003527,1.2107718693,-0.1210033465
H,0,0.1017943882,-2.4802448204,0.2716692917
H,0,-0.0657829804,-1.5477390593,-1.2340919924
H,0,1.5997441468,-0.747221044,1.2250803018
H,0,2.2548221991,-1.280191256,-0.3286533102
C,0,-0.9980511346,0.6992560375,0.0772394473
C,0,-2.0880646558,0.2954512898,-0.5505116033
N,0,-1.017358115,-0.7113595024,0.4117419719
H,0,-1.3136434818,-0.9633182649,1.3459771686

Zero-point correction= 0.140811 (Hartree/Particle)
Thermal correction to Energy= 0.147096
Thermal correction to Enthalpy= 0.148041
Thermal correction to Gibbs Free Energy= 0.111131
Sum of electronic and zero-point Energies= -287.777400
Sum of electronic and thermal Energies= -287.771115
Sum of electronic and thermal Enthalpies= -287.770171
Sum of electronic and thermal Free Energies= -287.807080

2-(1-azacyclohexylidene)carbene (triplet 24)

Charge = 0 Multiplicity = 3
Redundant internal coordinates found in file. (old form).
C,0,1.3969810609,0.6687589752,-0.4521531558
C,0,0.2181198092,1.4288477969,0.1680522845
C,0,0.2710154114,-1.5260943241,-0.0483388613
C,0,1.5273922774,-0.700940024,0.2205178969
H,0,0.47325331,1.7329092347,1.1936604754
H,0,-0.0104744338,2.3466054955,-0.3825973475
H,0,1.2282141775,0.5323333449,-1.529866837
H,0,2.3199394045,1.2485411985,-0.3376240813
H,0,0.2387991778,-2.419423891,0.5852740278
H,0,0.2570021929,-1.8552042094,-1.097675618
H,0,1.6566817721,-0.5683078781,1.3032230873
H,0,2.3977252139,-1.2522885209,-0.1534305871
C,0,-1.0561083751,0.6059037208,0.2462981116
C,0,-2.3498233524,1.1822975366,0.3752665253
N,0,-0.9378371296,-0.739398095,0.2142939877
H,0,-1.8234695065,-1.2321503706,0.2539371015

Zero-point correction= 0.140437 (Hartree/Particle)
Thermal correction to Energy= 0.146897
Thermal correction to Enthalpy= 0.147841
Thermal correction to Gibbs Free Energy= 0.109179
Sum of electronic and zero-point Energies= -287.745860
Sum of electronic and thermal Energies= -287.739400
Sum of electronic and thermal Enthalpies= -287.738456
Sum of electronic and thermal Free Energies= -287.777118

S**2 before annihilation 2.0306, after 2.0004

1-azacyclohepty-2-yne (25)

Charge = 0 Multiplicity = 1
Redundant internal coordinates found in file. (old form).
C,0,1.2915230993,0.8907335546,0.2763404033
C,0,1.8255145793,-0.53400888,-0.0521534529
C,0,-1.3725925739,0.7972683642,0.2390396035
C,0,-0.0494996458,1.2804340647,-0.414105542
H,0,2.2719128181,-0.5546512655,-1.0548641065
H,0,2.5997137661,-0.8302398608,0.6638401558
H,0,1.1566369174,0.9671733547,1.3638817487
H,0,2.0562666864,1.6278461422,-0.0049324311
H,0,-2.1906011652,1.4544257695,-0.0791041171
H,0,-1.2714782622,0.8753699723,1.3341801092
H,0,-0.0411685355,0.9687804539,-1.4675220576
H,0,-0.1000292096,2.3773139294,-0.4107362631
C,0,0.6077317975,-1.3685592861,-0.0047776044
C,0,-0.6102008925,-1.3541203873,-0.027771979
N,0,-1.7436313166,-0.5855036982,-0.1594866952
H,0,-2.5606096927,-0.9321017077,0.3283663682

Zero-point correction= 0.141086 (Hartree/Particle)
Thermal correction to Energy= 0.147560
Thermal correction to Enthalpy= 0.148504
Thermal correction to Gibbs Free Energy= 0.111118

Sum of electronic and zero-point Energies= -287.804961
Sum of electronic and thermal Energies= -287.798487
Sum of electronic and thermal Enthalpies= -287.797543
Sum of electronic and thermal Free Energies= -287.834929

TS from singlet 24 to 25 by C shift

Charge = 0 Multiplicity = 1
Redundant internal coordinates found in file. (old form).
C,0,-0.2388214553,1.4572464174,0.2444258257
C,0,1.0660108041,0.9482295363,-0.3814434883
C,0,-1.3702276091,-0.8309993017,0.2891534286
C,0,-1.4840461792,0.6364686184,-0.1340452615
H,0,1.0076905253,0.8554684779,-1.4720967845
H,0,1.8752403552,1.6394889986,-0.1520219214
H,0,-0.1179969704,1.4707968237,1.3362098002
H,0,-0.3850976549,2.4975527935,-0.0771269184
H,0,-2.2379015619,-1.4088954151,-0.0456765647
H,0,-1.3114507916,-0.9086149643,1.3812768406
H,0,-1.633387837,0.6856820148,-1.2232397826
H,0,-2.3730921804,1.0779645658,0.3346580723
C,0,2.1036176185,-0.3821191179,0.477490873
C,0,0.9900611396,-0.8153701056,0.0349607349
N,0,-0.1636467631,-1.47797329,-0.2571402905
H,0,-0.2461593698,-1.7133100619,-1.2406826135

Zero-point correction= 0.138755 (Hartree/Particle)
Thermal correction to Energy= 0.145017
Thermal correction to Enthalpy= 0.145961
Thermal correction to Gibbs Free Energy= 0.108956
Sum of electronic and zero-point Energies= -287.759661
Sum of electronic and thermal Energies= -287.753399
Sum of electronic and thermal Enthalpies= -287.752455
Sum of electronic and thermal Free Energies= -287.789461

****** 1 imaginary frequencies (negative Signs) ****** -454.1150 cm^{-1}

TS from singlet 24 to 25 by N shift

Charge = 0 Multiplicity = 1
Redundant internal coordinates found in file. (old form).
C,0,1.6158065382,-0.0123227889,-0.2924381996
C,0,1.0093334646,1.3046378092,0.2377371772
C,0,-0.6097023263,-1.3547060546,-0.2411660292
C,0,0.8617423206,-1.2507724782,0.2141710997
H,0,1.2227363664,1.4381206338,1.3063526069
H,0,1.4253111123,2.1701397568,-0.2941532463
H,0,1.5940043356,0.0050482997,-1.3895916712
H,0,2.6656623179,-0.0663491285,0.0203174723
H,0,-0.993495069,-2.3386144848,0.0619938465
H,0,-0.6690548182,-1.3011966651,-1.3348757364
H,0,0.893918063,-1.2754895642,1.3146891627
H,0,1.3859270064,-2.1500263953,-0.1366780721
C,0,-0.4356086409,1.2785003697,-0.0074237808
C,0,-1.6331058299,1.0773801456,-0.3935670453
N,0,-1.5685127871,-0.3764793255,0.2835774664
H,0,-1.5378165536,-0.3095537697,1.2974927292

Zero-point correction= 0.138158 (Hartree/Particle)
Thermal correction to Energy= 0.144470
Thermal correction to Enthalpy= 0.145414
Thermal correction to Gibbs Free Energy= 0.108282
Sum of electronic and zero-point Energies= -287.737442
Sum of electronic and thermal Energies= -287.731130
Sum of electronic and thermal Enthalpies= -287.730186
Sum of electronic and thermal Free Energies= -287.767318

****** 1 imaginary frequencies (negative Signs) ****** -573.1773 cm^{-1}

2-(1-oxacyclohexylidene)carbene (singlet 26)

Charge = 0 Multiplicity = 1
Redundant internal coordinates found in file. (old form).
C,0,-3.9123342998,-0.7139185043,-0.0153240386
C,0,-2.3748050775,-0.6870900375,-0.0139279882
C,0,-3.8555109137,1.5305137487,1.1242574852
C,0,-4.4380911397,0.1193006546,1.1625634113
H,0,-1.9843511581,-1.2295375097,0.8580462817
H,0,-1.9600938644,-1.1524177427,-0.9158289114
H,0,-4.280985151,-0.2925069412,-0.9604791232
H,0,-4.2708972189,-1.7480067578,0.0489655395
H,0,-4.13616947,2.1077240861,2.0086874868
H,0,-4.1963509407,2.0650148546,0.225531118
H,0,-4.1556675718,-0.3570442229,2.1108921103
H,0,-5.5329363514,0.1870445352,1.1362593549
C,0,-1.9190618604,0.7558778795,0.098079502
C,0,-1.046150861,1.2002781944,-0.8045553272
O,0,-2.4170856316,1.5117722628,1.1321447389

Zero-point correction= 0.127320 (Hartree/Particle)
Thermal correction to Energy= 0.133724
Thermal correction to Enthalpy= 0.134668
Thermal correction to Gibbs Free Energy= 0.097247
Sum of electronic and zero-point Energies= -307.622658
Sum of electronic and thermal Energies= -307.616254
Sum of electronic and thermal Enthalpies= -307.615310
Sum of electronic and thermal Free Energies= -307.652731

2-(1-oxacyclohexylidene)carbene (triplet 26)

Charge = 0 Multiplicity = 3
Redundant internal coordinates found in file. (old form).
C,0,-1.0545136538,1.1933411425,0.3077487989
C,0,0.4166254552,1.2857298794,-0.1086095515
C,0,-0.9042452151,-1.2758904683,0.1570177724
C,0,-1.657284928,-0.0535251367,-0.3395285965
H,0,0.4814001027,1.6042438827,-1.1605418244
H,0,0.9693788014,2.0308276452,0.4717626741
H,0,-1.1347292317,1.11453172,1.400694844
H,0,-1.5885794886,2.1003431323,0.0043507922
H,0,-1.1895254999,-2.1877255846,-0.3723176295
H,0,-1.0716348089,-1.4231370548,1.2312825598
H,0,-1.5822567281,0.0140676098,-1.4331281832

H,0,-2.7166549778,-0.1754002229,-0.0840565574
C,0,1.180860647,-0.0223130928,-0.0203343651
C,0,2.6026626573,-0.0617152936,0.0165514673
O,0,0.5308736983,-1.1870971481,-0.0254241412

Zero-point correction= 0.127030 (Hartree/Particle)
Thermal correction to Energy= 0.133457
Thermal correction to Enthalpy= 0.134402
Thermal correction to Gibbs Free Energy= 0.095603
Sum of electronic and zero-point Energies= -307.578486
Sum of electronic and thermal Energies= -307.572058
Sum of electronic and thermal Enthalpies= -307.571114
Sum of electronic and thermal Free Energies= -307.609913

S**2 before annihilation 2.0168, after 2.0001

1-oxacyclohepty-2-yne (27)

Charge = 0 Multiplicity = 1
Redundant internal coordinates found in file. (old form).
C,0,1.2514793087,0.9308265301,0.2648978701
C,0,1.8194000297,-0.4762397014,-0.0712300468
C,0,-1.4081593931,0.7164126903,0.2501527597
C,0,-0.115057715,1.2669397051,-0.4006260717
H,0,2.203927882,-0.4992098764,-1.0989735193
H,0,2.6472157523,-0.7292784556,0.5993637044
H,0,1.1339210243,1.0061862715,1.35472127
H,0,1.9821117433,1.6958912115,-0.031509393
H,0,-2.2763760997,1.2825759431,-0.0970163655
H,0,-1.3392030656,0.7808128001,1.3439554214
H,0,-0.103444942,0.9859259031,-1.4627732139
H,0,-0.2323570223,2.359196926,-0.3690003721
C,0,0.638421292,-1.3657905046,0.0615805827
C,0,-0.5710509146,-1.2966753006,-0.0510056153
O,0,-1.72878353,-0.6707434821,-0.0875918306

Zero-point correction= 0.128342 (Hartree/Particle)
Thermal correction to Energy= 0.134619
Thermal correction to Enthalpy= 0.135563
Thermal correction to Gibbs Free Energy= 0.098544
Sum of electronic and zero-point Energies= -307.649062
Sum of electronic and thermal Energies= -307.642785
Sum of electronic and thermal Enthalpies= -307.641841
Sum of electronic and thermal Free Energies= -307.678860

TS from singlet 26 to 27 by C shift

Charge = 0 Multiplicity = 1
Redundant internal coordinates found in file. (old form).
C,0,-0.1547497352,1.4679648902,0.258389979
C,0,1.1211475811,0.9004464777,-0.37344116
C,0,-1.3695605488,-0.7838581022,0.252294464
C,0,-1.4312757245,0.6927815757,-0.1166626701
H,0,1.0429365278,0.7860953146,-1.4607493008
H,0,1.965945402,1.5546962557,-0.1674310413

H,0,-0.0264786074,1.4800772253,1.3487925067
H,0,-0.2683123433,2.5104252227,-0.0684015677
H,0,-2.2384664563,-1.3332922768,-0.1149138092
H,0,-1.2618049724,-0.9321857223,1.3332689171
H,0,-1.6037956968,0.7733572918,-1.198380443
H,0,-2.2994615054,1.1354993617,0.3880466364
C,0,0.8918039765,-0.8841382145,0.0126890611
C,0,1.9934767891,-0.4806908747,0.5026772045
O,0,-0.2292102066,-1.4085328749,-0.4035534067

Zero-point correction= 0.126183 (Hartree/Particle)
Thermal correction to Energy= 0.132234
Thermal correction to Enthalpy= 0.133179
Thermal correction to Gibbs Free Energy= 0.096537
Sum of electronic and zero-point Energies= -307.603815
Sum of electronic and thermal Energies= -307.597764
Sum of electronic and thermal Enthalpies= -307.596820
Sum of electronic and thermal Free Energies= -307.633461

****** 1 imaginary frequencies (negative Signs) ****** -447.9128 cm^{-1}

TS from singlet 26 to 27 by O shift

Charge = 0 Multiplicity = 1
Redundant internal coordinates found in file. (old form).
C,0,-1.574050062,0.2088997552,-0.3085158988
C,0,-1.1653070554,-1.1771377065,0.2421633359
C,0,0.8143971348,1.2327377434,-0.2118017339
C,0,-0.6640706395,1.3451362273,0.1913086256
H,0,-1.4140642257,-1.2899392424,1.3046948633
H,0,-1.6735693018,-1.9838889343,-0.3038732433
H,0,-1.5431996969,0.1704697916,-1.4048441244
H,0,-2.6094817166,0.4132720019,-0.009588398
H,0,1.3474478482,2.130356948,0.1232556806
H,0,0.9216534436,1.141645206,-1.3009576647
H,0,-0.7151179669,1.4056806738,1.2874114367
H,0,-1.0454895806,2.2967517404,-0.2030278976
C,0,0.2711747057,-1.341458039,0.0282133931
C,0,1.4618393614,-1.152588429,-0.4238710356
O,0,1.4933116617,0.1522823937,0.4328397812

Zero-point correction= 0.125273 (Hartree/Particle)
Thermal correction to Energy= 0.131349
Thermal correction to Enthalpy= 0.132293
Thermal correction to Gibbs Free Energy= 0.095652
Sum of electronic and zero-point Energies= -307.575802
Sum of electronic and thermal Energies= -307.569726
Sum of electronic and thermal Enthalpies= -307.568782
Sum of electronic and thermal Free Energies= -307.605424

****** 1 imaginary frequencies (negative Signs) ****** -556.8859 cm^{-1}

2-(1-thiocyclohexylidene)carbene (singlet 28)

Charge = 0 Multiplicity = 1
Redundant internal coordinates found in file. (old form).
C,0,-3.8746723134,-0.7286634714,-0.0061654737
C,0,-2.3344843595,-0.8534997017,-0.0269971336
C,0,-3.9253799745,1.6100835631,1.1096337731
C,0,-4.4131680852,0.1524782503,1.138481254
H,0,-1.9788590952,-1.4134823264,0.8470627192
H,0,-2.0054505711,-1.3806317208,-0.9295462891
H,0,-4.1983036546,-0.3074362204,-0.9673844183
H,0,-4.3165879582,-1.7294298377,0.0774445989
H,0,-4.3711349242,2.1797125575,1.9325243624
H,0,-4.1740899466,2.0996444103,0.1615932396
H,0,-4.1361430914,-0.2937327455,2.1037299328
H,0,-5.5103109792,0.1551424854,1.089843291
C,0,-1.756387077,0.5199510838,-0.0157643545
C,0,-1.3721111289,1.6034657499,-0.6427602977
S,0,-2.113408371,1.6834024236,1.323616466

Zero-point correction= 0.124554 (Hartree/Particle)
Thermal correction to Energy= 0.131258
Thermal correction to Enthalpy= 0.132202
Thermal correction to Gibbs Free Energy= 0.094023
Sum of electronic and zero-point Energies= -630.261177
Sum of electronic and thermal Energies= -630.254473
Sum of electronic and thermal Enthalpies= -630.253529
Sum of electronic and thermal Free Energies= -630.291708

2-(1-thiocyclohexylidene)carbene (triplet 28)

Charge = 0 Multiplicity = 3
Redundant internal coordinates found in file. (old form).
C,0,-1.209625,1.289597,0.347434
C,0,0.22523,1.561212,-0.114829
C,0,-1.162581,-1.226507,0.136227
C,0,-1.800903,0.067724,-0.362055
H,0,0.211539,1.881491,-1.1677
H,0,0.670812,2.385467,0.453539
H,0,-1.22963,1.125551,1.434383
H,0,-1.820589,2.177027,0.144553
H,0,-1.507707,-2.090606,-0.440128
H,0,-1.417417,-1.393511,1.188751
H,0,-1.65845,0.163134,-1.447069
H,0,-2.880688,0.00371,-0.178297
C,0,1.186655,0.388647,-0.023621
C,0,2.588819,0.547967,-0.009949
S,0,0.66666,-1.251506,0.010796

Zero-point correction= 0.123678 (Hartree/Particle)
Thermal correction to Energy= 0.130586
Thermal correction to Enthalpy= 0.131530
Thermal correction to Gibbs Free Energy= 0.091357
Sum of electronic and zero-point Energies= -630.197316
Sum of electronic and thermal Energies= -630.190409

Sum of electronic and thermal Enthalpies= -630.189465
Sum of electronic and thermal Free Energies= -630.229637

S**2 before annihilation 2.0195, after 2.0002

1-thiocyclohepty-2-yne (29)

Charge = 0 Multiplicity = 1
Redundant internal coordinates found in file. (old form).
C,0,1.7632225548,0.6960860197,0.2470195805
C,0,2.0029249253,-0.8112217125,-0.0484292338
C,0,-0.8356599722,1.2181890942,0.296240628
C,0,0.5221448172,1.3219840249,-0.4506014762
H,0,2.400537015,-0.9405172664,-1.0632141143
H,0,2.7301481095,-1.2309461237,0.654736042
H,0,1.6622378742,0.8277758135,1.3325107207
H,0,2.6592822745,1.251489706,-0.0600672258
H,0,-1.4664453153,2.0727327393,0.0253230495
H,0,-0.6660325369,1.2489862191,1.3782630515
H,0,0.4203763183,0.9274573768,-1.4699848362
H,0,0.7241719364,2.3965341871,-0.5512755553
C,0,0.6643191969,-1.4112657453,0.054735886
C,0,-0.5510502459,-1.3306399853,-0.0129710772
S,0,-1.8785231218,-0.2562558073,-0.0720855194
Zero-point correction= 0.125253 (Hartree/Particle)
Thermal correction to Energy= 0.132092
Thermal correction to Enthalpy= 0.133037
Thermal correction to Gibbs Free Energy= 0.094365
Sum of electronic and zero-point Energies= -630.286216
Sum of electronic and thermal Energies= -630.279377
Sum of electronic and thermal Enthalpies= -630.278433
Sum of electronic and thermal Free Energies= -630.317104

TS from singlet 28 to 29 by C shift

Charge = 0 Multiplicity = 1
Redundant internal coordinates found in file. (old form).
C,0,1.3888210046,1.0664397735,0.1324989131
C,0,1.5955568594,-0.3242331872,-0.4674343128
C,0,-1.1965602526,0.9763154322,0.4503976839
C,0,0.0144634843,1.7097650526,-0.1363913244
H,0,1.2624412206,-0.3961750835,-1.5081734906
H,0,2.6470243328,-0.6079804723,-0.4421979591
H,0,1.5747318402,1.0103701058,1.2129813723
H,0,2.1545793635,1.731155628,-0.2913351536
H,0,-2.1150794806,1.5312533095,0.2318113218
H,0,-1.1073602965,0.8523018484,1.5341083032
H,0,-0.1278496708,1.8113574259,-1.2210786825
H,0,0.0232138025,2.7251008225,0.2830031031
C,0,0.0950580296,-1.2777526193,0.1513241095
C,0,1.1798912769,-1.7080989176,0.6561177309
S,0,-1.429439234,-0.6772160184,-0.3024156948

Zero-point correction= 0.122836 (Hartree/Particle)
Thermal correction to Energy= 0.129413
Thermal correction to Enthalpy= 0.130357
Thermal correction to Gibbs Free Energy= 0.092222

Sum of electronic and zero-point Energies= -630.227525
Sum of electronic and thermal Energies= -630.220947
Sum of electronic and thermal Enthalpies= -630.220003
Sum of electronic and thermal Free Energies= -630.258138

****** 1 imaginary frequencies (negative Signs) ****** -497.3868

TS from singlet 28 to 29 by S shift

Charge = 0 Multiplicity = 1
Redundant internal coordinates found in file. (old form).
C,0,1.8936792236,0.0403704014,0.214651533
C,0,1.2842460456,-1.2733704699,-0.3149446457
C,0,-0.3193728117,1.3925349494,0.3958433084
C,0,1.1000242022,1.292993515,-0.1905618045
H,0,1.3531098021,-1.3267838123,-1.4093935093
H,0,1.8128494848,-2.1437357176,0.0943767641
H,0,1.9538151379,-0.0157674463,1.309439752
H,0,2.9182246825,0.1272807319,-0.1676144115
H,0,-0.7181070803,2.3959907543,0.2031515402
H,0,-0.306493651,1.237112921,1.4806844965
H,0,1.0400479521,1.3536251424,-1.285872024
H,0,1.6626483709,2.1734472472,0.1493381091
C,0,-0.1219828276,-1.3682255373,0.0902783441
C,0,-1.2887307407,-1.3747585269,0.5814977964
S,0,-1.5683882003,0.2772397176,-0.3267935687

Zero-point correction= 0.123613 (Hartree/Particle)
Thermal correction to Energy= 0.130054
Thermal correction to Enthalpy= 0.130998
Thermal correction to Gibbs Free Energy= 0.093144
Sum of electronic and zero-point Energies= -630.245761
Sum of electronic and thermal Energies= -630.239320
Sum of electronic and thermal Enthalpies= -630.238376
Sum of electronic and thermal Free Energies= -630.276230

****** 1 imaginary frequencies (negative Signs) ****** -380.7608 cm^{-1}

Summary output of (R/U)CCSD(T)/cc-pVTZ//CCSD/6-311+G calculations**

2-(1-azacyclobutylidene)carbene (singlet 12)

1\1\GINC-NODE36\SP\RCCSD(T)-FC\CC-pVTZ\C4H5N1\RABLENP\01-Dec-2018\0\\#
n CCSD=(T,T1Diag)/cc-pVTZ geom=check guess=read\\Betalactame carbene s
inglet\\0,1\C,0,1.3937124166,0.0406064948,0.2737463325\C,0,0.292780527
3,1.0881630052,-0.0628756478\H,0,2.034105936,-0.1620721231,-0.59302403
44\H,0,2.0025072284,0.2127454021,1.1649767558\H,0,0.450387434,1.718397
6744,-0.937819743\H,0,-0.0143544242,1.6814063804,0.8013211735\C,0,-0.6
029117571,-0.155732809,-0.2496112417\C,0,-1.7266047075,-0.21865819,-0.
9495513973\N,0,0.3203078509,-0.97895688,0.46333425\H,0,0.4781837856,-1
.9086020247,0.0889478827\\Version=AM64L-G09RevD.01\State=1-A\HF=-208.7
207585\MP2=-209.5328859\MP3=-209.567432\MP4D=-209.5877294\MP4DQ=-209.5
653038\MP4SDQ=-209.5732405\CCSD=-209.573151\CCSD(T)=-209.6137782\RMSD=
6.080e-09\PG=C01 [X(C4H5N1)]\\@

T1 Diagnostic = 0.01522158

2-(1-azacyclobutylidene)carbene (triplet 12)

1\1\GINC-NODE33\SP\UCCSD(T)-FC\CC-pVTZ\C4H5N1(3)\RABLENP\01-Dec-2018\0
\\#n CCSD=(T,T1Diag)/cc-pVTZ geom=check guess=read\\Betalactame carben
e triplet\\0,3\C,0,1.38854623,-0.15332019,0.\C,0,0.42738827,1.07528184
,0.\H,0,2.00005505,-0.27724234,-0.89864571\H,0,2.00005505,-0.27724234,
0.89864571\H,0,0.46622202,1.69811961,-0.89651124\H,0,0.46622202,1.6981
1961,0.89651124\C,0,-0.71925931,0.03954787,0.\C,0,-2.12425373,0.043632
59,0.\N,0,0.17407715,-0.98410361,0.\H,0,0.01434652,-1.98386784,0.\\Ver
sion=AM64L-G09RevD.01\State=3-A"\HF=-208.7182236\MP2=-209.5043417\MP3=
-209.5408934\MP4D=-209.5589596\MP4DQ=-209.5379799\PUHF=-208.7224653\PM
P2-0=-209.507248\PMP3-0=-209.542599\MP4SDQ=-209.5457588\CCSD=-209.5456
819\CCSD(T)=-209.5841242\S2=2.030018\S2-1=2.010945\S2A=2.000412\RMSD=5
.411e-09\PG=CS [SG(C4H1N1),X(H4)]\\@

T1 Diagnostic = 0.01719886

1-azacyclopent-2-yne (13)

1\1\GINC-NODE07\SP\RCCSD(T)-FC\CC-pVTZ\C4H5N1\RABLENP\01-Dec-2018\0\\#
n CCSD=(T,T1Diag)/cc-pVTZ geom=check guess=read\\N cyclopentyne single
t\\0,1\C,0,-0.9447322867,-0.1506947145,0.513951684\C,0,-0.5370065731,1
.1440609554,-0.3157189949\H,0,-0.9877722642,0.1139391761,1.5764021693\
H,0,-1.8958191614,-0.6134533154,0.2247811341\H,0,-0.9922946824,2.04781
26811,0.0990309548\H,0,-0.8154650866,1.0607172342,-1.373384989\C,0,0.9
876868244,1.0436719972,-0.0662414985\C,0,1.1605193465,-0.2181241116,-0
.1472309173\N,0,0.2179492446,-1.088012823,0.3180309513\H,0,0.050732638
9,-1.9024230795,-0.2578994839\\Version=AM64L-G09RevD.01\State=1-A\HF=-
208.6934329\MP2=-209.527362\MP3=-209.5535344\MP4D=-209.573862\MP4DQ=-2
09.5493796\MP4SDQ=-209.5615047\CCSD=-209.5649732\CCSD(T)=-209.6128616\
RMSD=6.421e-09\PG=C01 [X(C4H5N1)]\\@

T1 Diagnostic = 0.04058311

TS from singlet 12 to 13 by C shift

1\1\GINC-NODE36\SP\RCCSD(T)-FC\CC-pVTZ\C4H5N1\RABLENP\03-Dec-2018\0\\#
n CCSD=(T,T1Diag)/cc-pVTZ geom=check guess=read\\TS to azacyclopentyne
- C shift\\0,1\C,0,-0.3784278518,1.0010202073,-0.1227232957\H,0,-0.72
61316528,1.4065224957,-1.0757384527\H,0,-0.405081323,1.774560257,0.647
3015565\C,0,1.1858946356,-1.2969652371,0.5167879221\C,0,-0.0331893635,
-0.9385829448,0.4399307048\C,0,1.0542282008,0.3272021495,-0.2293817271
\H,0,1.3849929201,0.1936176478,-1.2591839859\H,0,1.7937115624,0.855949
5465,0.3670441104\N,0,-1.1942951461,-0.1782474839,0.3210804377\H,0,-1.
7838215216,-0.533196788,-0.4276128502\\Version=AM64L-G09RevD.01\State=
1-A\HF=-208.6964659\MP2=-209.5358486\MP3=-209.5590118\MP4D=-209.579386
9\MP4DQ=-209.5545278\MP4SDQ=-209.5627502\CCSD=-209.5617014\CCSD(T)=-20
9.6063298\RMSD=4.011e-09\PG=C01 [X(C4H5N1)]\\@

T1 Diagnostic = 0.01355023

TS from singlet 12 to 13 by N shift

1\1\GINC-NODE34\SP\RCCSD(T)-FC\CC-pVTZ\C4H5N1\RABLENP\02-Dec-2018\0\\#
n CCSD=(T,T1Diag)/cc-pVTZ geom=check guess=read\\TS to azacyclopentyne
- N shift\\0,1\C,0,0.825099146,-0.8461402598,0.182535972\C,0,1.111668
9998,0.6602908549,-0.0044425748\H,0,1.3162488159,-1.4451960331,-0.5874
76017\H,0,1.0885320947,-1.2309702009,1.1702032087\H,0,1.7939994135,0.8
922697779,-0.8242361771\H,0,1.4224485083,1.1772597597,0.9064067978\C,0
,-1.5078461354,0.4325432952,0.0085650001\C,0,-0.3274485659,0.868109370
6,-0.3312921909\N,0,-0.7034235931,-0.9397959602,0.0164751238\H,0,-0.93
1860304,-1.4404908145,-0.8395982128\\Version=AM64L-G09RevD.01\State=1-
A\HF=-208.6750577\MP2=-209.5107471\MP3=-209.5355906\MP4D=-209.5568774\
MP4DQ=-209.531873\MP4SDQ=-209.5427651\CCSD=-209.5422021\CCSD(T)=-209.5
884597\RMSD=5.725e-09\PG=C01 [X(C4H5N1)]\\@

T1 Diagnostic = 0.02213288

2-(1-oxacyclobutylidene)carbene (singlet 14)

1\1\GINC-NODE32\SP\RCCSD(T)-FC\CC-pVTZ\C4H4O1\RABLENP\02-Dec-2018\0\\#
n CCSD=(T,T1Diag)/cc-pVTZ geom=check guess=read\\Betalactone carbene s
inglet\\0,1\C,0,1.3283097628,-0.0468871138,0.\C,0,0.2750462649,1.09158
32717,0.\H,0,1.9419422524,-0.1239946237,-0.9005181713\H,0,1.9419422524
,-0.1239946237,0.9005181713\H,0,0.2499920311,1.7089006226,-0.898934846
8\H,0,0.2499920311,1.7089006226,0.8989348468\C,0,-0.6972815838,-0.0938
073457,0.\C,0,-2.0305214287,-0.064657345,0.\O,0,0.2818935027,-1.064747
908,0.\\Version=AM64L-G09RevD.01\State=1-A'\HF=-228.5510005\MP2=-229.3
837357\MP3=-229.4133888\MP4D=-229.4340985\MP4DQ=-229.4119337\MP4SDQ=-2
29.4212221\CCSD=-229.4206681\CCSD(T)=-229.4612424\RMSD=8.732e-09\PG=CS
[SG(C4O1),X(H4)]\\@

T1 Diagnostic = 0.01558942

2-(1-oxacyclobutylidene)carbene (triplet 14)

1\1\GINC-NODE04\SP\UCCSD(T)-FC\CC-pVTZ\C4H4O1(3)\RABLENP\01-Dec-2018\0
\\#n CCSD=(T,T1Diag)/cc-pVTZ geom=check guess=read\\Betalactone carben
e triplet\\0,3\C,0,0.1770749634,1.3419847222,0.\C,0,-1.0679631163,0.43
39880481,0.\H,0,0.3562640831,1.9293207444,-0.901935317\H,0,0.356264083
1,1.9293207444,0.901935317\H,0,-1.6915920427,0.4701742658,-0.896272056
2\H,0,-1.6915920427,0.4701742658,0.8962720562\C,0,-0.0313915242,-0.683
7659081,0.\C,0,-0.010192172,-2.0893701414,0.\O,0,1.0344443295,0.149433
4014,0.\\Version=AM64L-G09RevD.01\State=3-A"\HF=-228.544079\MP2=-229.3
505711\MP3=-229.3813775\MP4D=-229.4003432\MP4DQ=-229.3799459\PUHF=-228
.5472631\PMP2-0=-229.3525807\PMP3-0=-229.3824852\MP4SDQ=-229.3896738\C
CSD=-229.3890512\CCSD(T)=-229.4276063\S2=2.017495\S2-1=2.004524\S2A=2.
000131\RMSD=4.069e-09\PG=CS [SG(C4O1),X(H4)]\\@

T1 Diagnostic = 0.01995477

1-oxacyclopent-2-yne (15)

1\1\GINC-NODE04\SP\RCCSD(T)-FC\CC-pVTZ\C4H4O1\RABLENP\01-Dec-2018\0\\#
n CCSD=(T,T1Diag)/cc-pVTZ geom=check guess=read\\Oxo cyclopentyne sing
let\\0,1\C,0,0.9907719822,-0.2197163,-0.1706516289\C,0,0.3907691947,1.
2169820125,0.0981057073\H,0,1.0702923596,-0.3882247857,-1.250165951\H,
0,1.9387027147,-0.4468882202,0.3243382722\H,0,0.7837301917,1.948519581
8,-0.6130110082\H,0,0.5887263074,1.5634824668,1.1181523001\C,0,-1.0836
824791,0.8330462212,-0.129976167\C,0,-1.1031455041,-0.3570820151,0.278
279497\O,0,-0.0352267671,-1.1585329715,0.3447949785\\Version=AM64L-G09
RevD.01\State=1-A\HF=-228.5014783\MP2=-229.370139\MP3=-229.38636\MP4D=
-229.4088019\MP4DQ=-229.3832196\MP4SDQ=-229.3975008\CCSD=-229.4010843\
CCSD(T)=-229.4523007\RMSD=1.611e-09\PG=C01 [X(C4H4O1)]\\@

T1 Diagnostic = 0.04638607

TS from singlet 14 to 15 by C shift

1\1\GINC-NODE33\SP\RCCSD(T)-FC\CC-pVTZ\C4H4O1\RABLENP\05-Dec-2018\0\\#
n CCSD=(T,T1Diag)/cc-pVTZ geom=check guess=read\\TS to oxacyclopentyne
- C shift\\0,1\C,0,0.9398406871,0.6232635029,-0.0650607193\C,0,-0.583
6017453,1.0264361151,0.0526490858\H,0,1.5291244717,1.175942762,-0.7995
144154\H,0,1.4224205782,0.6273726546,0.9165391661\H,0,-0.9836996315,1.
4210372786,-0.8851800368\H,0,-0.731009857,1.7596148124,0.8492195696\C,
0,-0.3685108587,-0.9629793107,-0.118179429\C,0,-1.3026017135,-0.353555
8238,0.4683745102\O,0,0.879262069,-0.8006819913,-0.5135747311\\Version
=AM64L-G09RevD.01\State=1-A\HF=-228.5131555\MP2=-229.3753546\MP3=-229.
3917733\MP4D=-229.4127576\MP4DQ=-229.3882734\MP4SDQ=-229.4001234\CCSD=
-229.400336\CCSD(T)=-229.4466849\RMSD=3.191e-09\PG=C01 [X(C4H4O1)]\\@

T1 Diagnostic = 0.02974928

TS from singlet 14 to 15 by O shift

1\1\GINC-NODE32\SP\RCCSD(T)-FC\CC-pVTZ\C4H4O1\RABLENP\02-Dec-2018\0\\#
n CCSD=(T,T1Diag)/cc-pVTZ geom=check guess=read\\TS to oxacyclopentyne
- O shift\\0,1\C,0,0.9691446982,0.5944855923,-0.0184395849\C,0,0.9559
735357,-0.9509303426,0.0621235806\H,0,1.5832177374,1.0721015295,0.7455
947962\H,0,1.2328557496,0.9585647171,-1.0159430851\H,0,1.4272157068,-1
.3566120159,0.9593024202\H,0,1.3336877248,-1.4539919543,-0.8315290932\
C,0,-1.4055571701,-0.0499503824,-0.3613930736\C,0,-0.5320157554,-0.909
3200942,0.1386605726\O,0,-0.4380267871,0.9722371004,0.258375547\\Versi
on=AM64L-G09RevD.01\State=1-A\HF=-228.489031\MP2=-229.3408599\MP3=-229
.3613638\MP4D=-229.383646\MP4DQ=-229.359393\MP4SDQ=-229.3731377\CCSD=-
229.3713595\CCSD(T)=-229.4178277\RMSD=8.315e-09\PG=C01 [X(C4H4O1)]\\@

T1 Diagnostic = 0.02495660

2-(1-thiocyclobutylidene)carbene (singlet 16)

1\1\GINC-NODE32\SP\RCCSD(T)-FC\CC-pVTZ\C4H4S1\RABLENP\01-Dec-2018\0\\#
n CCSD=(T,T1Diag)/cc-pVTZ geom=check guess=read\\Betalactone sulfur ca
rbene singlet\\0,1\C,0,0.9595252334,2.7880362551,0.0648817568\C,0,-0.5
837070488,2.7934566287,-0.081405909\H,0,1.5020252576,3.397159566,-0.65
90884701\H,0,1.2888013291,3.0128816887,1.0808566085\H,0,-0.8976817404,
3.1591899821,-1.0629672678\H,0,-1.1287129737,3.3210161753,0.7047112396
\C,0,-0.7025983106,1.2635187042,-0.0213233754\C,0,-1.7349352316,0.4899
125648,0.25095016\S,0,1.046071255,0.9712876751,-0.2741208426\\Version=
AM64L-G09RevD.01\State=1-A\HF=-551.2362306\MP2=-552.0129835\MP3=-552.0
551843\MP4D=-552.0766765\MP4DQ=-552.0525517\MP4SDQ=-552.0601951\CCSD=-
552.0596985\CCSD(T)=-552.1020364\RMSD=5.861e-09\PG=C01 [X(C4H4S1)]\\@

T1 Diagnostic = 0.01554885

2-(1-thiocyclobutylidene)carbene (triplet 16)

1\1\GINC-NODE34\SP\UCCSD(T)-FC\CC-pVTZ\C4H4S1(3)\RABLENP\01-Dec-2018\0
\\#n CCSD=(T,T1Diag)/cc-pVTZ geom=check guess=read\\Beta thiolactone c
arbene triplet\\0,3\C,0,1.3386360556,0.5672826178,0.\C,0,0.0035385106,
1.3546411344,0.\H,0,1.9481571912,0.6941890035,-0.8959817532\H,0,1.9481
571912,0.6941890035,0.8959817532\H,0,-0.1518905683,1.973495116,-0.8898
900497\H,0,-0.1518905683,1.973495116,0.8898900497\C,0,-0.8794451021,0.
1075815319,0.\C,0,-2.2543676151,-0.1211913945,0.\S,0,0.440197026,-1.04
23992815,0.\\Version=AM64L-G09RevD.01\State=3-A"\HF=-551.2137483\MP2=-
551.9646636\MP3=-552.0094059\MP4D=-552.0287791\MP4DQ=-552.0060668\PUHF
=-551.2172622\PMP2-0=-551.9668787\PMP3-0=-552.0106356\MP4SDQ=-552.0144
06\CCSD=-552.0152369\CCSD(T)=-552.0563242\S2=2.021509\S2-1=2.005495\S2
A=2.000231\RMSD=4.083e-09\PG=CS [SG(C4S1),X(H4)]\\@

T1 Diagnostic = 0.02517095

1-thiocyclopent-2-yne (17)

1\1\GINC-NODE33\SP\RCCSD(T)-FC\CC-pVTZ\C4H4S1\RABLENP\02-Dec-2018\0\\#
n CCSD=(T,T1Diag)/cc-pVTZ geom=check guess=read\\sulfur cyclopentyne\\
0,1\C,0,0.980166349,0.3538762475,0.1089205317\C,0,0.0257257284,1.57481
77276,-0.1380438279\H,0,1.8784630989,0.3632642921,-0.5164640743\H,0,1.
2742859015,0.3431434615,1.1628371333\H,0,-0.0132501712,1.8515230742,-1
.1979564886\H,0,0.342650932,2.4382161866,0.451905657\C,0,-1.2601090602
,0.9296784626,0.2929669461\C,0,-1.4883474131,-0.2778868422,0.112912030
9\S,0,-0.0364273553,-1.1977216097,-0.2089189183\\Version=AM64L-G09RevD
.01\State=1-A\HF=-551.2123594\MP2=-552.0302551\MP3=-552.0554605\MP4D=-
552.0792623\MP4DQ=-552.0514011\MP4SDQ=-552.0628577\CCSD=-552.0635094\C
CSD(T)=-552.1135715\RMSD=3.764e-09\PG=C01 [X(C4H4S1)]\\@

T1 Diagnostic = 0.03103232

TS from singlet 16 to 17 by C shift

1\1\GINC-NODE07\SP\RCCSD(T)-FC\CC-pVTZ\C4H4S1\RABLENP\02-Dec-2018\0\\#
n CCSD=(T,T1Diag)/cc-pVTZ geom=check guess=read\\TS to thiocyclopentyn
e - C shift\\0,1\C,0,0.2358255915,1.1987191627,0.1559328055\C,0,-1.212
7201294,0.6887642353,-0.1756889493\H,0,0.5487820655,2.0136888347,-0.49
96494396\H,0,0.3084327202,1.4992874479,1.2030612437\H,0,-1.4524911687,
0.8323168261,-1.2299475975\H,0,-1.9366477723,1.1872581863,0.4675216991
\C,0,-0.3399994159,-0.9618544725,0.087775201\C,0,-1.6067212447,-0.9639
109401,0.1464388004\S,0,1.2607572637,-0.3163764404,-0.1058588834\\Vers
ion=AM64L-G09RevD.01\State=1-A\HF=-551.2019852\MP2=-552.007495\MP3=-55
2.0367188\MP4D=-552.0587304\MP4DQ=-552.0322776\MP4SDQ=-552.0411092\CCS
D=-552.0399975\CCSD(T)=-552.0868824\RMSD=7.169e-09\PG=C01 [X(C4H4S1)]\
\@

T1 Diagnostic = 0.01822438

TS from singlet 16 to 17 by S shift

1\1\GINC-NODE04\SP\RCCSD(T)-FC\CC-pVTZ\C4H4S1\RABLENP\02-Dec-2018\0\\#
n CCSD=(T,T1Diag)/cc-pVTZ geom=check guess=read\\TS to thiocyclopentyn
e - S shift\\0,1\C,0,1.0487358686,-0.7083242239,0.3330797552\C,0,1.316
6898037,0.7117182621,-0.2107393658\H,0,1.6208640362,-1.5036036973,-0.1
482847516\H,0,1.1517001371,-0.7512081862,1.4196069082\H,0,1.6761129817
,0.6904639671,-1.2440852899\H,0,1.9666544907,1.3400741274,0.4032436428
\C,0,-0.119265212,1.1133959028,-0.1410529492\C,0,-1.3104350431,1.10199
33282,0.3401104611\S,0,-0.7591381729,-0.8205566002,-0.1401780207\\Vers
ion=AM64L-G09RevD.01\State=1-A\HF=-551.2182481\MP2=-552.0213944\MP3=-5
52.0525527\MP4D=-552.074631\MP4DQ=-552.0476864\MP4SDQ=-552.0562721\CCS
D=-552.0550178\CCSD(T)=-552.1018299\RMSD=7.735e-09\PG=C01 [X(C4H4S1)]\
\@

T1 Diagnostic = 0.01629645

2-(1-azacyclopentylidene)carbene (singlet 18)

1\1\GINC-N18\SP\RCCSD(T)-FC\CC-pVTZ\C5H7N1\DMTHAMAT\03-Dec-2018\0\\# r
ccsd=(t,t1diag)/cc-pvtz geom=connectivity\\gammalactamcarbene singlet\
\0,1\C,0,0.173096,1.204091,0.159495\C,0,-1.250002,0.755473,-0.23831\C,
0,-1.280601,-0.707376,0.209934\H,0,0.555881,2.026784,-0.448282\H,0,0.1
98657,1.502519,1.213315\H,0,-1.374803,0.808717,-1.325002\H,0,-2.022948
,1.363893,0.240708\H,0,-1.428514,-0.770755,1.299946\H,0,-2.055204,-1.2
98109,-0.288394\C,0,0.965471,-0.105447,-0.024109\C,0,2.292207,-0.04156
4,-0.018749\N,0,0.053439,-1.173375,-0.203592\H,0,0.351832,-2.05048,0.2
03287\\Version=ES64L-G16RevA.03\State=1-A\HF=-247.8061211\MP2=-248.793
659\MP3=-248.8366752\MP4D=-248.8604406\MP4DQ=-248.8339158\MP4SDQ=-248.
8428884\CCSD=-248.8424935\CCSD(T)=-248.8906003\RMSD=6.273e-09\PG=C01 [
X(C5H7N1)]\\@

T1 Diagnostic = 0.01402090

2-(1-azacyclopentylidene)carbene (triplet 18)

1\1\GINC-NSCC-N12\SP\UCCSD(T)-FC\CC-pVTZ\C5H7N1(3)\DMTHAMAT\05-Dec-201
8\0\\# uccsd=(t,t1diag)/cc-pvtz geom=connectivity\\gammalactamcarbene
triplet\\0,3\C,0,0.038086,1.221934,0.169264\C,0,-1.3477,0.668843,-0.22
4036\C,0,-1.249551,-0.82855,0.125256\H,0,0.365746,2.068405,-0.43835\H,
0,0.063133,1.532532,1.222454\H,0,-1.497963,0.779989,-1.303654\H,0,-2.1
75237,1.160538,0.294633\H,0,-1.553906,-1.028262,1.161838\H,0,-1.830352
,-1.471673,-0.542659\C,0,0.967026,0.02433,0.001701\C,0,2.381175,-0.005
962,-0.087891\N,0,0.184855,-1.075417,-0.033729\H,0,0.600382,-1.997181,
-0.063926\\Version=ES64L-G16RevA.03\State=3-A\HF=-247.8041181\MP2=-248
.7645129\MP3=-248.8102394\MP4D=-248.8317717\MP4DQ=-248.8066845\PUHF=-2
47.8082789\PMP2-0=-248.7673608\PMP3-0=-248.8119144\MP4SDQ=-248.8153865
\CCSD=-248.8150407\CCSD(T)=-248.8607706\S2=2.029305\S2-1=2.010649\S2A=
2.000387\RMSD=3.893e-09\PG=C01 [X(C5H7N1)]\\@

T1 Diagnostic = 0.01531155

1-azacyclohex-2-yne (19)

1\1\GINC-NSCC-N2\SP\RCCSD(T)-FC\CC-pVTZ\C5H7N1\DMTHAMAT\04-Dec-2018\0\
\# rccsd=(t,t1diag)/cc-pvtz geom=connectivity\\azacyclohexyne\\0,1\C,0
,1.583742,-0.158556,0.141739\C,0,0.672217,1.046617,-0.288542\C,0,-0.76
8427,0.998937,0.270462\H,0,2.454552,-0.226417,-0.518482\H,0,1.945162,-
0.046153,1.170464\H,0,0.616008,1.061254,-1.38367\H,0,1.130761,1.989842
,0.037529\H,0,-0.724702,1.006168,1.370738\H,0,-1.317402,1.885412,-0.06
6359\C,0,0.614711,-1.286285,0.013748\C,0,-0.612265,-1.24722,-0.038484\
N,0,-1.520768,-0.209601,-0.21216\H,0,-2.398865,-0.323856,0.28136\\Vers
ion=ES64L-G16RevA.03\State=1-A\HF=-247.7861981\MP2=-248.8226992\MP3=-2
48.8463158\MP4D=-248.873179\MP4DQ=-248.8420903\MP4SDQ=-248.8526251\CCS
D=-248.8517617\CCSD(T)=-248.9065383\RMSD=6.317e-09\PG=C01 [X(C5H7N1)]\
\@

T1 Diagnostic = 0.01496655

TS from singlet 18 to 19 by C shift

1\1\GINC-NSCC-N12\SP\RCCSD(T)-FC\CC-pVTZ\C5H7N1\DMTHAMAT\04-Dec-2018\0
\\# rccsd=(t,t1diag)/cc-pvtz geom=connectivity\\TS to azacyclohexyne-C
shift\\0,1\C,0,0.788928,0.906679,0.432259\C,0,-0.580017,1.185314,-0.2
07116\C,0,-1.462018,-0.086201,-0.119988\H,0,1.506563,1.682841,0.173712
\H,0,0.751377,0.805285,1.521337\H,0,-0.416535,1.455031,-1.255632\H,0,-
1.064503,2.035733,0.288675\H,0,-2.1711,-0.034971,0.712927\H,0,-2.02748
9,-0.237892,-1.043839\C,0,0.725351,-0.835929,-0.065228\C,0,1.865547,-0
.3817,-0.407928\N,0,-0.557297,-1.250499,0.113168\H,0,-0.703974,-1.6815
13,1.018655\\Version=ES64L-G16RevA.03\State=1-A\HF=-247.7746128\MP2=-2
48.7907949\MP3=-248.8221423\MP4D=-248.8463955\MP4DQ=-248.8171262\MP4SD
Q=-248.8262564\CCSD=-248.8248819\CCSD(T)=-248.8773486\RMSD=8.227e-09\P
G=C01 [X(C5H7N1)]\\@

T1 Diagnostic = 0.01203218

TS from singlet 18 to 19 by N shift

1\1\GINC-NSCC-N2\SP\RCCSD(T)-FC\CC-pVTZ\C5H7N1\DMTHAMAT\04-Dec-2018\0\
\# rccsd=(t,t1diag)/cc-pvtz geom=connectivity\\TS to azacyclohexyne-N
shift\\0,1\C,0,-0.103712,1.228714,-0.329841\C,0,-1.326008,0.462252,0.1
88315\H,0,-0.04121,1.154183,-1.421615\H,0,-0.159195,2.291336,-0.06185\
H,0,-1.429437,0.635165,1.266695\H,0,-2.260046,0.76733,-0.2991\C,0,0.41
3617,-1.108886,0.038993\C,0,1.638248,-0.794573,-0.160831\N,0,1.168284,
0.718699,0.211948\H,0,1.205972,0.823956,1.223278\C,0,-1.048702,-1.0636
46,-0.046104\H,0,-1.384197,-1.370087,-1.043216\H,0,-1.550533,-1.675934
,0.708982\\Version=ES64L-G16RevA.03\State=1-A\HF=-247.7510484\MP2=-248
.7657994\MP3=-248.7978243\MP4D=-248.8228529\MP4DQ=-248.7932898\MP4SDQ=
-248.8050492\CCSD=-248.8040477\CCSD(T)=-248.857917\RMSD=8.940e-09\PG=C
01 [X(C5H7N1)]\\@

T1 Diagnostic = 0.01928363

2-(1-oxacyclopentylidene)carbene (singlet 20)

1\1\GINC-N19\SP\RCCSD(T)-FC\CC-pVTZ\C5H6O1\DMTHAMAT\03-Dec-2018\0\\# r
ccsd=(t,t1diag)/cc-pvtz geom=connectivity\\gammalactonecarbene singlet
\\0,1\C,0,0.168268,1.18194,0.232536\C,0,-1.214854,0.750549,-0.292513\C
,0,-1.261948,-0.713239,0.141139\H,0,0.599468,2.03197,-0.300029\H,0,0.1
22581,1.414936,1.302729\H,0,-1.245772,0.822014,-1.384894\H,0,-2.031029
,1.343133,0.131399\H,0,-1.541634,-0.81033,1.19814\H,0,-1.910371,-1.345
559,-0.469076\O,0,0.087579,-1.195324,-0.030201\C,0,0.934569,-0.124111,
0.016387\C,0,2.25832,-0.077401,-0.136992\\Version=ES64L-G16RevA.03\Sta
te=1-A\HF=-267.6359562\MP2=-268.6438104\MP3=-268.6818841\MP4D=-268.705
9356\MP4DQ=-268.6796716\MP4SDQ=-268.6898956\CCSD=-268.6890936\CCSD(T)=
-268.7371961\RMSD=5.459e-09\PG=C01 [X(C5H6O1)]\\@

T1 Diagnostic = 0.01422591

2-(1-oxacyclopentylidene)carbene (triplet 20)

1\1\GINC-NSCC-N13\SP\UCCSD(T)-FC\CC-pVTZ\C5H6O1(3)\DMTHAMAT\04-Dec-201
8\0\\# uccsd=(t,t1diag)/cc-pvtz geom=connectivity\\deltalactonecarbene
triplet\\0,3\C,0,0.03278,1.202932,0.185456\C,0,-1.335948,0.653985,-0.
245896\C,0,-1.200677,-0.820929,0.134741\H,0,0.389217,2.057756,-0.39293
\H,0,0.037963,1.485493,1.247565\H,0,-1.461649,0.753145,-1.329476\H,0,-
2.180873,1.13491,0.254093\H,0,-1.443403,-1.002231,1.188061\H,0,-1.7562
88,-1.515856,-0.496805\O,0,0.209338,-1.118397,-0.042196\C,0,0.94001,-0
.000303,0.00531\C,0,2.353889,-0.030025,-0.101767\\Version=ES64L-G16Rev
A.03\State=3-A\HF=-267.6248754\MP2=-268.604052\MP3=-268.6445812\MP4D=-
268.6667765\MP4DQ=-268.6423345\PUHF=-267.6280224\PMP2-0=-268.6060358\P
MP3-0=-268.6456782\MP4SDQ=-268.652725\CCSD=-268.6518537\CCSD(T)=-268.6
975285\S2=2.017209\S2-1=2.004428\S2A=2.000119\RMSD=3.945e-09\PG=C01 [X
(C5H6O1)]\\@

T1 Diagnostic = 0.01699090

1-oxacyclohex-2-yne (21)

1\1\GINC-NSCC-N14\SP\RCCSD(T)-FC\CC-pVTZ\C5H6O1\DMTHAMAT\04-Dec-2018\0
\\# rccsd=(t,t1diag)/cc-pvtz geom=connectivity\\oxacyclohexyne\\0,1\C,
0,1.571644,-0.084035,0.093794\C,0,0.588032,1.075713,-0.283265\C,0,-0.8
34015,0.927324,0.302777\H,0,2.375667,-0.151954,-0.645643\H,0,2.033357,
0.070611,1.075485\H,0,0.50302,1.124799,-1.375639\H,0,0.980344,2.040755
,0.065148\H,0,-0.800545,0.884788,1.397947\H,0,-1.479142,1.74637,-0.023
009\O,0,-1.522269,-0.297165,-0.155114\C,0,0.654192,-1.276652,0.115783\
C,0,-0.55228,-1.19869,-0.104652\\Version=ES64L-G16RevA.03\State=1-A\HF
=-267.6080603\MP2=-268.6666572\MP3=-268.6845522\MP4D=-268.7117029\MP4D
Q=-268.6807904\MP4SDQ=-268.6930873\CCSD=-268.6918002\CCSD(T)=-268.7471
603\RMSD=7.748e-09\PG=C01 [X(C5H6O1)]\\@

T1 Diagnostic = 0.01669211

TS from singlet 20 to 21 by C shift

1\1\GINC-NSCC-N15\SP\RCCSD(T)-FC\CC-pVTZ\C5H6O1\DMTHAMAT\04-Dec-2018\0
\\# rccsd=(t,t1diag)/cc-pvtz geom=connectivity\\TS to oxacyclohexyne-C
shift\\0,1\C,0,0.79944281,1.0440257,0.03491674\C,0,-0.73356614,1.0990
679,-0.20790863\C,0,-1.36365021,-0.18217445,0.32906949\H,0,1.32435538,
1.59506127,-0.74299778\H,0,1.04071882,1.44093755,1.02018811\H,0,-0.939
5743,1.15977214,-1.28144101\H,0,-1.14975919,1.98762038,0.28015135\H,0,
-1.30076842,-0.25151801,1.42133717\H,0,-2.38522806,-0.3632969,-0.00779
018\O,0,-0.53773051,-1.22733236,-0.2543009\C,0,0.69668716,-0.81332067,
-0.08627762\C,0,1.88643635,-0.43925141,0.15435994\\Version=ES64L-G16Re
vA.03\State=1-A\HF=-267.6003303\MP2=-268.639\MP3=-268.6638234\MP4D=-26
8.6884374\MP4DQ=-268.6593022\MP4SDQ=-268.670117\CCSD=-268.6681768\CCSD
(T)=-268.7210876\RMSD=3.356e-09\PG=C01 [X(C5H6O1)]\\@

T1 Diagnostic = 0.01335026

TS from singlet 20 to 21 by O shift

1\1\GINC-N18\SP\RCCSD(T)-FC\CC-pVTZ\C5H6O1\DMTHAMAT\04-Dec-2018\0\\# r
ccsd=(t,t1diag)/cc-pvtz geom=connectivity\\TS to oxacyclohexyne-O shif
t\\0,1\C,0,-1.161098,-0.941933,0.028972\C,0,-1.227967,0.628735,-0.1104
72\C,0,0.122841,1.214782,0.297728\H,0,-1.495983,-1.250379,1.026033\H,0
,-1.771316,-1.433459,-0.732931\H,0,-2.053232,1.023523,0.493006\H,0,-1.
413164,0.869454,-1.162028\H,0,0.208874,2.263948,-0.004378\H,0,0.288869
,1.129791,1.37952\O,0,1.160501,0.528906,-0.410911\C,0,0.277091,-1.1794
05,-0.100387\C,0,1.481123,-0.861199,0.265504\\Version=ES64L-G16RevA.03
\State=1-A\HF=-267.5677289\MP2=-268.5998651\MP3=-268.6279097\MP4D=-268
.6537461\MP4DQ=-268.6246536\MP4SDQ=-268.639045\CCSD=-268.6370383\CCSD(
T)=-268.6912256\RMSD=8.132e-09\PG=C01 [X(C5H6O1)]\\@

T1 Diagnostic = 0.02161052

2-(1-thiocyclopentylidene)carbene (singlet 22)

1\1\GINC-N19\SP\RCCSD(T)-FC\CC-pVTZ\C5H6S1\DMTHAMAT\04-Dec-2018\0\\# r
ccsd=(t,t1diag)/cc-pvtz geom=connectivity\\gamma thiolactonecarbene si
nglet\\0,1\C,0,0.298446,1.384122,0.306377\C,0,-1.07375,1.152408,-0.342
592\C,0,-1.528325,-0.237021,0.109196\H,0,0.819267,2.253195,-0.105585\H
,0,0.19246,1.516915,1.390221\H,0,-0.970414,1.173601,-1.433232\H,0,-1.7
92856,1.923814,-0.042457\H,0,-1.89102,-0.215622,1.141207\H,0,-2.304433
,-0.65391,-0.537447\C,0,1.055993,0.07903,0.039487\C,0,2.353489,0.1346,
-0.193329\S,0,-0.043007,-1.317301,0.004528\\Version=ES64L-G16RevA.03\S
tate=1-A\HF=-590.3145482\MP2=-591.2690799\MP3=-591.3186029\MP4D=-591.3
436741\MP4DQ=-591.3153051\MP4SDQ=-591.3240305\CCSD=-591.3231247\CCSD(T
)=-591.3732852\RMSD=3.045e-09\PG=C01 [X(C5H6S1)]\\@

T1 Diagnostic = 0.01405002

2-(1-thiocyclopentylidene)carbene (triplet 22)

1\1\GINC-N19\SP\UCCSD(T)-FC\CC-pVTZ\C5H6S1(3)\DMTHAMAT\04-Dec-2018\0\\
uccsd=(t,t1diag)/cc-pvtz geom=connectivity\\gamma thiolactonecarbene
triplet\\0,3\C,0,0.028343,1.429002,0.213569\C,0,-1.32816,0.937634,-0.
307115\C,0,-1.472312,-0.512591,0.172153\H,0,0.393141,2.319157,-0.30640
1\H,0,-0.031377,1.667427,1.285911\H,0,-1.32716,0.957843,-1.403409\H,0,
-2.160661,1.554035,0.049498\H,0,-1.744928,-0.561052,1.231756\H,0,-2.19
3447,-1.091259,-0.409964\C,0,1.013953,0.281289,0.03215\C,0,2.413833,0.
363762,-0.096601\S,0,0.195656,-1.240046,-0.033271\\Version=ES64L-G16Re
vA.03\State=3-A\HF=-590.2885373\MP2=-591.2155998\MP3=-591.2685506\MP4D
=-591.2914947\MP4DQ=-591.2646343\PUHF=-590.2921752\PMP2-0=-591.2179142
\PMP3-0=-591.2698398\MP4SDQ=-591.2738593\CCSD=-591.2741498\CCSD(T)=-59
1.3226523\S2=2.022641\S2-1=2.006048\S2A=2.000247\RMSD=9.972e-09\PG=C01
[X(C5H6S1)]\\@

T1 Diagnostic = 0.02129363

1-thiocyclohex-2-yne (23)

1\1\GINC-NSCC-N2\SP\RCCSD(T)-FC\CC-pVTZ\C5H6S1\DMTHAMAT\04-Dec-2018\0\
\# rccsd=(t,t1diag)/cc-pvtz geom=connectivity\\thiocyclohexyne\\0,1\C,
0,1.867674,-0.326796,0.084195\C,0,1.107801,0.977087,-0.343719\C,0,-0.2
53065,1.196968,0.348057\H,0,2.651469,-0.553854,-0.645616\H,0,2.338544,
-0.216154,1.067619\H,0,0.955185,0.948555,-1.428858\H,0,1.744504,1.8423
8,-0.115581\H,0,-0.129947,1.181453,1.436474\H,0,-0.657627,2.171663,0.0
53561\C,0,0.76826,-1.312584,0.113058\C,0,-0.452135,-1.342327,-0.018691
\S,0,-1.570834,-0.033008,-0.091563\\Version=ES64L-G16RevA.03\State=1-A
\HF=-590.312795\MP2=-591.30855\MP3=-591.3406714\MP4D=-591.3679615\MP4D
Q=-591.3361033\MP4SDQ=-591.3463082\CCSD=-591.3449116\CCSD(T)=-591.4002
663\RMSD=4.783e-09\PG=C01 [X(C5H6S1)]\\@

T1 Diagnostic = 0.01519235

TS from singlet 22 to 23 by C shift

1\1\GINC-NSCC-N12\SP\RCCSD(T)-FC\CC-pVTZ\C5H6S1\DMTHAMAT\04-Dec-2018\0
\\# rccsd=(t,t1diag)/cc-pvtz geom=connectivity\\TS to thiocyclohexyne
- C shift\\0,1\C,0,1.57487,0.357603,-0.030087\C,0,0.457462,1.390933,-0
.307667\C,0,-0.83414,1.078575,0.447083\H,0,2.312433,0.39312,-0.830533\
H,0,2.041005,0.566725,0.932554\H,0,0.237876,1.420745,-1.379957\H,0,0.8
4679,2.375033,-0.013027\H,0,-0.678069,1.051967,1.529565\H,0,-1.640267,
1.777102,0.206978\C,0,0.309324,-1.086345,-0.004912\C,0,1.507281,-1.439
536,0.216953\S,0,-1.325534,-0.587004,-0.148363\\Version=ES64L-G16RevA.
03\State=1-A\HF=-590.2735755\MP2=-591.256101\MP3=-591.2933173\MP4D=-59
1.3192243\MP4DQ=-591.2881871\MP4SDQ=-591.2976076\CCSD=-591.2959963\CCS
D(T)=-591.3507265\RMSD=6.246e-09\PG=C01 [X(C5H6S1)]\\@

T1 Diagnostic = 0.01466933

TS from singlet 22 to 23 by S shift

1\1\GINC-NSCC-N14\SP\RCCSD(T)-FC\CC-pVTZ\C5H6S1\DMTHAMAT\04-Dec-2018\0
\\# rccsd=(t,t1diag)/cc-pvtz geom=connectivity\\TS to thiocyclohexyne-
S shift\\0,1\C,0,-1.450759,-0.76104,-0.385994\C,0,-1.456974,0.590825,0
.371755\C,0,-0.192989,1.385982,0.022298\H,0,-2.216109,-1.442899,0.0013
65\H,0,-1.607827,-0.631117,-1.462919\H,0,-1.474185,0.374981,1.444634\H
,0,-2.34956,1.177049,0.121772\H,0,-0.318629,1.888843,-0.941722\H,0,0.0
07112,2.137571,0.790424\C,0,-0.113903,-1.289063,-0.100637\C,0,0.975277
,-1.406811,0.551175\S,0,1.337206,0.336013,-0.169071\\Version=ES64L-G16
RevA.03\State=1-A\HF=-590.289954\MP2=-591.2702217\MP3=-591.3092386\MP4
D=-591.3347419\MP4DQ=-591.3035017\MP4SDQ=-591.3139408\CCSD=-591.312657
5\CCSD(T)=-591.3674746\RMSD=4.418e-09\PG=C01 [X(C5H6S1)]\\@

T1 Diagnostic = 0.01783429

2-(1-azacyclohexylidene)carbene (singlet 24)

1\1\GINC-N18\SP\RCCSD(T)-FC\CC-pVTZ\C6H9N1\DMTHAMAT\03-Dec-2018\0\\# r
ccsd=(t,t1diag)/cc-pvtz geom=connectivity\\deltalactamcarbene singlet\
\0,1\C,0,1.40096,0.73016,-0.336117\C,0,0.220148,1.512281,0.288322\C,0,
0.107733,-1.471671,-0.195962\C,0,1.42465,-0.743153,0.122088\H,0,0.4011
02,1.67403,1.360755\H,0,0.101046,2.491633,-0.186529\H,0,1.29996,0.7637
14,-1.42932\H,0,2.350722,1.21423,-0.077636\H,0,0.107869,-2.493882,0.19
8957\H,0,-0.057815,-1.518127,-1.279359\H,0,1.596975,-0.784821,1.207863
\H,0,2.258908,-1.269875,-0.358611\C,0,-1.001766,0.686435,0.094163\C,0,
-2.088376,0.297757,-0.54885\N,0,-1.017541,-0.733444,0.387072\H,0,-1.31
607,-1.013654,1.312506\\Version=ES64L-G16RevA.03\State=1-A\HF=-286.849
0817\MP2=-288.0370378\MP3=-288.0803397\MP4D=-288.1085186\MP4DQ=-288.07
58565\MP4SDQ=-288.0863193\CCSD=-288.0845219\CCSD(T)=-288.1434854\RMSD=
9.808e-09\PG=C01 [X(C6H9N1)]\\@

T1 Diagnostic = 0.01207640

2-(1-azacyclohexylidene)carbene (triplet 24)

1\1\GINC-NSCC-N17\SP\UCCSD(T)-FC\CC-pVTZ\C6H9N1(3)\DMTHAMAT\07-Dec-201
8\0\\# uccsd=(t,t1diag)/cc-pvtz geom=connectivity\\deltalactamcarbene
triplet\\0,3\C,0,1.031182,1.190691,-0.335993\C,0,-0.423268,1.311118,0.
135363\C,0,0.955887,-1.298834,-0.124234\C,0,1.678538,-0.030474,0.32374
6\H,0,-0.445145,1.618676,1.19099\H,0,-0.971532,2.075136,-0.424487\H,0,
1.061509,1.075021,-1.428816\H,0,1.582984,2.104977,-0.089594\H,0,1.2520
37,-2.161026,0.483599\H,0,1.206032,-1.521788,-1.171772\H,0,1.614209,0.
064184,1.416201\H,0,2.738636,-0.116844,0.059106\C,0,-1.201461,0.011071
,0.029019\C,0,-2.622048,-0.046725,-0.004935\N,0,-0.497178,-1.140995,-0
.010738\H,0,-1.071473,-1.972449,-0.097859\\Version=ES64L-G16RevA.03\St
ate=3-A\HF=-286.8534328\MP2=-287.9902447\MP3=-288.0455425\MP4D=-288.07
0797\MP4DQ=-288.0413241\PUHF=-286.857732\PMP2-0=-287.9932114\PMP3-0=-2
88.0472975\MP4SDQ=-288.0512623\CCSD=-288.050733\CCSD(T)=-288.1043475\S
2=2.030974\S2-1=2.0116\S2A=2.000425\RMSD=9.768e-09\PG=C01 [X(C6H9N1)]\
\@

T1 Diagnostic = 0.01470484

1-azacyclohepty-2-yne (25)

1\1\GINC-NSCC-N2\SP\RCCSD(T)-FC\CC-pVTZ\C6H9N1\DMTHAMAT\03-Dec-2018\0\
\# rccsd=(t,t1diag)/cc-pvtz geom=connectivity\\azacycloheptyne\\0,1\C,
0,1.297127,0.88679,0.273345\C,0,1.826661,-0.540502,-0.051274\C,0,-1.36
7318,0.798818,0.248229\C,0,-0.046118,1.276321,-0.412864\H,0,2.26857,-0
.566465,-1.055848\H,0,2.603399,-0.835249,0.662578\H,0,1.16722,0.968259
,1.361128\H,0,2.062166,1.621044,-0.014524\H,0,-2.185344,1.456316,-0.06
9166\H,0,-1.26119,0.881481,1.34256\H,0,-0.043108,0.960058,-1.464934\H,
0,-0.094325,2.37331,-0.414066\C,0,0.607348,-1.372251,0.005131\C,0,-0.6
10642,-1.355328,-0.012543\N,0,-1.743026,-0.584889,-0.142605\H,0,-2.558
562,-0.927621,0.350364\\Version=ES64L-G16RevA.03\State=1-A\HF=-286.870
8945\MP2=-288.0744823\MP3=-288.1101441\MP4D=-288.1391567\MP4DQ=-288.10
53217\MP4SDQ=-288.1160954\CCSD=-288.1146625\CCSD(T)=-288.1748796\RMSD=
7.869e-09\PG=C01 [X(C6H9N1)]\\@

T1 Diagnostic = 0.01161414

TS from singlet 24 to 25 by C shift

1\1\GINC-NSCC-N12\SP\RCCSD(T)-FC\CC-pVTZ\C6H9N1\DMTHAMAT\03-Dec-2018\0
\\# rccsd=(t,t1diag)/cc-pvtz geom=connectivity\\TS to azacycloheptyne-
C shift\\0,1\C,0,0.156784,1.469455,-0.238132\C,0,-1.115283,0.891051,0.
39487\C,0,1.406951,-0.755839,-0.295567\C,0,1.445996,0.714722,0.130393\
H,0,-1.045038,0.799266,1.484903\H,0,-1.961314,1.539136,0.172288\H,0,0.
028292,1.478846,-1.329084\H,0,0.2501,2.515369,0.084957\H,0,2.306055,-1
.287852,0.032317\H,0,1.345224,-0.834214,-1.387473\H,0,1.599639,0.76951
7,1.218722\H,0,2.30745,1.203449,-0.342944\C,0,-2.086885,-0.490412,-0.4
60568\C,0,-0.94917,-0.865234,-0.026218\N,0,0.239751,-1.466663,0.256903
\H,0,0.340974,-1.69933,1.239332\\Version=ES64L-G16RevA.03\State=1-A\HF
=-286.8307021\MP2=-288.0218438\MP3=-288.0629111\MP4D=-288.0909184\MP4D
Q=-288.0574872\MP4SDQ=-288.0677518\CCSD=-288.0661004\CCSD(T)=-288.1262
064\RMSD=8.720e-09\PG=C01 [X(C6H9N1)]\\@

T1 Diagnostic = 0.01159764

TS from singlet 24 to 25 by N shift

1\1\GINC-NSCC-N2\SP\RCCSD(T)-FC\CC-pVTZ\C6H9N1\DMTHAMAT\04-Dec-2018\0\
\# rccsd=(t,t1diag)/cc-pvtz geom=connectivity\\TS to azacycloheptyne-N
shift\\0,1\C,0,-1.615018,0.093672,-0.295691\C,0,-1.080632,-1.25279,0.
23793\C,0,0.67836,1.316304,-0.240021\C,0,-0.797753,1.29117,0.211358\H,
0,-1.303702,-1.373192,1.306125\H,0,-1.540454,-2.095819,-0.293959\H,0,-
1.591197,0.073544,-1.392756\H,0,-2.661379,0.203723,0.014069\H,0,1.1129
36,2.278939,0.06289\H,0,0.737754,1.258103,-1.333489\H,0,-0.831553,1.31
9189,1.311748\H,0,-1.27259,2.216418,-0.14215\C,0,0.364323,-1.303627,-0
.003245\C,0,1.571837,-1.166822,-0.386323\N,0,1.582554,0.289419,0.28869
5\H,0,1.545608,0.225718,1.30261\\Version=ES64L-G16RevA.03\State=1-A\HF
=-286.8038041\MP2=-287.9944475\MP3=-288.0357662\MP4D=-288.0645597\MP4D
Q=-288.0307754\MP4SDQ=-288.0440503\CCSD=-288.0428782\CCSD(T)=-288.1045
484\RMSD=8.180e-09\PG=C01 [X(C6H9N1)]\\@

T1 Diagnostic = 0.01914159

2-(1-oxacyclohexylidene)carbene (singlet 26)

1\1\GINC-N18\SP\RCCSD(T)-FC\CC-pVTZ\C6H8O1\DMTHAMAT\04-Dec-2018\0\\# r
ccsd=(t,t1diag)/cc-pvtz geom=connectivity\\deltalactonecarbene singlet
\\0,1\C,0,-0.90576,1.25647,0.306884\C,0,0.482336,1.255876,-0.354863\C,
0,-0.831975,-1.259326,0.23849\C,0,-1.661131,-0.019882,-0.091156\H,0,0.
386013,1.3628,-1.444051\H,0,1.107163,2.078524,0.012263\H,0,-0.786529,1
.29079,1.398404\H,0,-1.465823,2.149744,0.006169\H,0,-1.306464,-2.17247
9,-0.129163\H,0,-0.682349,-1.346517,1.324576\H,0,-1.870454,-0.007695,-
1.169131\H,0,-2.621949,-0.086147,0.434587\C,0,1.146011,-0.080509,-0.07
9152\C,0,2.37046,-0.066161,0.444999\O,0,0.455094,-1.223478,-0.403109\\
Version=ES64L-G16RevA.03\State=1-A\HF=-306.6881558\MP2=-307.8709157\MP
3=-307.9187568\MP4D=-307.9465041\MP4DQ=-307.9160932\MP4SDQ=-307.927246
8\CCSD=-307.9263035\CCSD(T)=-307.9819065\RMSD=5.219e-09\PG=C01 [X(C6H8
O1)]\\@

T1 Diagnostic = 0.01332774

2-(1-oxacyclohexylidene)carbene (triplet 26)

1\1\GINC-N18\SP\UCCSD(T)-FC\CC-pVTZ\C6H8O1(3)\DMTHAMAT\07-Dec-2018\0\\
 # uccsd=(t,t1diag)/cc-pvtz geom=connectivity\\deltalactonecarbene trip
 let\\0,3\C,0,-1.061996,1.186528,0.30623\C,0,0.407241,1.289108,-0.11444
 \C,0,-0.891684,-1.282061,0.166702\C,0,-1.656106,-0.068319,-0.333591\H,
 0,0.46664,1.603179,-1.168024\H,0,0.955314,2.041479,0.460965\H,0,-1.138
 713,1.112216,1.399742\H,0,-1.604339,2.087642,-0.000014\H,0,-1.170782,-
 2.198711,-0.35759\H,0,-1.055054,-1.425616,1.242084\H,0,-1.584484,-0.00
 5269,-1.42769\H,0,-2.713763,-0.197739,-0.074754\C,0,1.182505,-0.012142
 ,-0.02204\C,0,2.604675,-0.039616,0.011277\O,0,0.542171,-1.18227,-0.019
 943\\Version=ES64L-G16RevA.03\State=3-A\HF=-306.6685644\MP2=-307.82270
 08\MP3=-307.8731712\MP4D=-307.8990119\MP4DQ=-307.8703037\PUHF=-306.671
 7282\PMP2-0=-307.8247094\PMP3-0=-307.8742943\MP4SDQ=-307.881821\CCSD=-
 307.8808053\CCSD(T)=-307.9341899\S2=2.017524\S2-1=2.004669\S2A=2.00012
 1\RMSD=8.937e-09\PG=C01 [X(C6H8O1)]\\@

T1 Diagnostic = 0.01622739

1-oxacyclohepty-2-yne (27)

1\1\GINC-N19\SP\RCCSD(T)-FC\CC-pVTZ\C6H8O1\DMTHAMAT\03-Dec-2018\0\\# r
 ccsd=(t,t1diag)/cc-pvtz geom=connectivity\\oxacycloheptyne\\0,1\C,0,1.
 260114,0.920923,0.261148\C,0,1.818749,-0.490139,-0.073789\C,0,-1.40075
 1,0.721818,0.256104\C,0,-0.106772,1.263431,-0.400387\H,0,2.19956,-0.51
 7611,-1.102806\H,0,2.647422,-0.746456,0.594497\H,0,1.146784,0.999382,1
 .351201\H,0,1.994105,1.6811,-0.039505\H,0,-2.266895,1.292206,-0.089323
 \H,0,-1.327619,0.788251,1.349514\H,0,-0.100473,0.979994,-1.461935\H,0,
 -0.217668,2.356414,-0.370805\C,0,0.633136,-1.372567,0.065109\C,0,-0.57
 6303,-1.296728,-0.043438\O,0,-1.730532,-0.664213,-0.077415\\Version=ES
 64L-G16RevA.03\State=1-A\HF=-306.6952796\MP2=-307.9203961\MP3=-307.950
 5484\MP4D=-307.9797181\MP4DQ=-307.9461454\MP4SDQ=-307.9583815\CCSD=-30
 7.9565105\CCSD(T)=-308.0169768\RMSD=5.762e-09\PG=C01 [X(C6H8O1)]\\@

T1 Diagnostic = 0.01252777

TS from singlet 26 to 27 by C shift

1\1\GINC-NSCC-N12\SP\RCCSD(T)-FC\CC-pVTZ\C6H8O1\DMTHAMAT\03-Dec-2018\0
 \\# rccsd=(t,t1diag)/cc-pvtz geom=connectivity\\TS to oxacycloheptyne-
 C shift\\0,1\C,0,-0.083133,1.471802,0.246043\C,0,1.159498,0.843297,-0.
 394309\C,0,-1.395816,-0.724251,0.272936\C,0,-1.396593,0.750519,-0.1084
 51\H,0,1.064312,0.723365,-1.47966\H,0,2.034485,1.46138,-0.203155\H,0,0
 .057599,1.487418,1.334862\H,0,-0.154337,2.51547,-0.088771\H,0,-2.29205
 8,-1.237916,-0.080074\H,0,-1.28274,-0.868089,1.353974\H,0,-1.577355,0.
 829521,-1.188939\H,0,-2.238828,1.235307,0.401546\C,0,0.85616,-0.926132
 ,0.010096\C,0,1.979866,-0.567535,0.484686\O,0,-0.291371,-1.404082,-0.3
 89474\\Version=ES64L-G16RevA.03\State=1-A\HF=-306.6547804\MP2=-307.869
 3248\MP3=-307.9037526\MP4D=-307.9321397\MP4DQ=-307.8988092\MP4SDQ=-307
 .9106556\CCSD=-307.9084682\CCSD(T)=-307.9690201\RMSD=4.536e-09\PG=C01
 [X(C6H8O1)]\\@

T1 Diagnostic = 0.01254699

TS from singlet 26 to 27 by O shift

1\1\GINC-N19\SP\RCCSD(T)-FC\CC-pVTZ\C6H8O1\DMTHAMAT\04-Dec-2018\0\\# r
ccsd=(t,t1diag)/cc-pvtz geom=connectivity\\TS to oxacycloheptyne-O shi
ft\\0,1\C,0,1.5604,0.300648,0.308442\C,0,1.236049,-1.108519,-0.239704\
C,0,-0.885689,1.177864,0.211397\C,0,0.583074,1.378871,-0.192745\H,0,1.
490678,-1.207851,-1.302189\H,0,1.792472,-1.8821,0.307379\H,0,1.532449,
0.262274,1.40485\H,0,2.581421,0.566817,0.008583\H,0,-1.472256,2.041003
,-0.124843\H,0,-0.986721,1.082287,1.300764\H,0,0.629846,1.440541,-1.28
8976\H,0,0.906374,2.352495,0.199794\C,0,-0.18775,-1.35913,-0.024659\C,
0,-1.387451,-1.241918,0.427793\O,0,-1.498258,0.057205,-0.431063\\Versi
on=ES64L-G16RevA.03\State=1-A\HF=-306.6247391\MP2=-307.8312091\MP3=-30
7.8691142\MP4D=-307.8984885\MP4DQ=-307.8654149\MP4SDQ=-307.8808943\CCS
D=-307.8788404\CCSD(T)=-307.940332\RMSD=8.994e-09\PG=C01 [X(C6H8O1)]\\
@

T1 Diagnostic = 0.02084432

2-(1-thiocyclohexylidene)carbene (singlet 28)

1\1\GINC-N18\SP\RCCSD(T)-FC\CC-pVTZ\C6H8S1\DMTHAMAT\03-Dec-2018\0\\# r
ccsd=(t,t1diag)/cc-pvtz geom=connectivity\\delta thiolactonecarbene si
nglet\\0,1\C,0,-1.773435,0.400482,0.231875\C,0,-0.827374,1.44964,-0.39
4567\C,0,0.008556,-1.472683,0.413742\C,0,-1.38494,-1.057291,-0.085259\
H,0,-0.915692,1.44757,-1.488289\H,0,-1.075474,2.453502,-0.032027\H,0,-
1.773237,0.544269,1.320653\H,0,-2.796442,0.580714,-0.121376\H,0,0.2015
6,-2.52531,0.179114\H,0,0.110567,-1.323968,1.494383\H,0,-1.434502,-1.2
20201,-1.170872\H,0,-2.127933,-1.723685,0.372971\C,0,0.569206,1.110641
,-0.000442\C,0,1.563152,1.169847,0.850065\S,0,1.30501,-0.489794,-0.415
44\\Version=ES64L-G16RevA.03\State=1-A\HF=-629.3666203\MP2=-630.520224
\MP3=-630.5694455\MP4D=-630.598604\MP4DQ=-630.5637735\MP4SDQ=-630.5743
709\CCSD=-630.572279\CCSD(T)=-630.6337831\RMSD=3.996e-09\PG=C01 [X(C6H
8S1)]\\@

T1 Diagnostic = 0.01348022

2-(1-thiocyclohexylidene)carbene (triplet 28)

1\1\GINC-NSCC-N2\SP\UCCSD(T)-FC\CC-pVTZ\C6H8S1(3)\DMTHAMAT\06-Dec-2018
\0\\# uccsd=(t,t1diag)/cc-pvtz geom=connectivity\\delta thiolactonecar
bene triplet\\0,3\C,0,-1.209625,1.289597,0.347434\C,0,0.22523,1.561212
,-0.114829\C,0,-1.162581,-1.226507,0.136227\C,0,-1.800903,0.067724,-0.
362055\H,0,0.211539,1.881491,-1.1677\H,0,0.670812,2.385467,0.453539\H,
0,-1.22963,1.125551,1.434383\H,0,-1.820589,2.177027,0.144553\H,0,-1.50
7707,-2.090606,-0.440128\H,0,-1.417417,-1.393511,1.188751\H,0,-1.65845
,0.163134,-1.447069\H,0,-2.880688,0.00371,-0.178297\C,0,1.186655,0.388
647,-0.023621\C,0,2.588819,0.547967,-0.009949\S,0,0.66666,-1.251506,0.
010796\\Version=ES64L-G16RevA.03\State=3-A\HF=-629.331873\MP2=-630.435
3317\MP3=-630.4979985\MP4D=-630.5246488\MP4DQ=-630.4933695\PUHF=-629.3
356606\PMP2-0=-630.4377439\PMP3-0=-630.4993272\MP4SDQ=-630.5038404\CCS
D=-630.5039043\CCSD(T)=-630.5603831\S2=2.023889\S2-1=2.006404\S2A=2.00
0278\RMSD=8.144e-09\PG=C01 [X(C6H8S1)]\\@

T1 Diagnostic = 0.01995962

1-thiocyclohepty-2-yne (29)

1\1\GINC-NSCC-N15\SP\RCCSD(T)-FC\CC-pVTZ\C6H8S1\DMTHAMAT\04-Dec-2018\0
\\# rccsd=(t,t1diag)/cc-pvtz geom=connectivity\\thiocycloheptyne\\0,1\
C,0,1.762861,0.701011,0.239828\C,0,2.012146,-0.804998,-0.054308\C,0,-0
.839575,1.203137,0.306178\C,0,0.512146,1.315893,-0.450338\H,0,2.40366,
-0.933281,-1.07159\H,0,2.747479,-1.217668,0.644586\H,0,1.668433,0.8341
22,1.325736\H,0,2.652436,1.26269,-0.074611\H,0,-1.478823,2.052234,0.03
7987\H,0,-0.662649,1.23744,1.386926\H,0,0.406326,0.918523,-1.468204\H,
0,0.705151,2.391764,-0.554546\C,0,0.678973,-1.415146,0.059395\C,0,-0.5
37426,-1.344042,0.000025\S,0,-1.873548,-0.280059,-0.051935\\Version=ES
64L-G16RevA.03\State=1-A\HF=-629.3864245\MP2=-630.5523809\MP3=-630.595
6746\MP4D=-630.6257569\MP4DQ=-630.5905749\MP4SDQ=-630.6011621\CCSD=-63
0.5993336\CCSD(T)=-630.6608858\RMSD=4.880e-09\PG=C01 [X(C6H8S1)]\\@

T1 Diagnostic = 0.01236696

TS from singlet 28 to 29 by C shift

1\1\GINC-N19\SP\RCCSD(T)-FC\CC-pVTZ\C6H8S1\DMTHAMAT\03-Dec-2018\0\\# r
ccsd=(t,t1diag)/cc-pvtz geom=connectivity\\TS to thiocyckloheptyne-Csh
ift\\0,1\C,0,1.425062,1.014277,0.114563\C,0,1.578908,-0.383655,-0.4843
92\C,0,-1.156937,1.006786,0.470377\C,0,0.067936,1.700147,-0.136076\H,0
,1.228461,-0.447474,-1.519956\H,0,2.621136,-0.700483,-0.473688\H,0,1.6
2493,0.954902,1.192374\H,0,2.205156,1.653418,-0.322267\H,0,-2.060558,1
.590003,0.26364\H,0,-1.055822,0.88257,1.553018\H,0,-0.086983,1.803632,
-1.218855\H,0,0.114931,2.715685,0.280278\C,0,0.058247,-1.287737,0.1588
69\C,0,1.136217,-1.751013,0.649005\S,0,-1.452991,-0.640318,-0.274289\\
Version=ES64L-G16RevA.03\State=1-A\HF=-629.3288964\MP2=-630.48696\MP3=
-630.5339007\MP4D=-630.5634499\MP4DQ=-630.5282333\MP4SDQ=-630.53862\CC
SD=-630.5367141\CCSD(T)=-630.5990498\RMSD=7.194e-09\PG=C01 [X(C6H8S1)]
\\@

T1 Diagnostic = 0.01340585

TS from singlet 28 to 29 by S shift

1\1\GINC-N18\SP\RCCSD(T)-FC\CC-pVTZ\C6H8S1\DMTHAMAT\03-Dec-2018\0\\# r
ccsd=(t,t1diag)/cc-pvtz geom=connectivity\\TS to thiocyckloheptyne-S s
hift\\0,1\C,0,1.893544,0.09778,0.212606\C,0,1.328839,-1.235079,-0.3187
98\C,0,-0.364862,1.371304,0.4037\C,0,1.055426,1.322948,-0.186986\H,0,1
.396328,-1.283217,-1.413577\H,0,1.888724,-2.087498,0.086681\H,0,1.9588
03,0.040941,1.307065\H,0,2.913305,0.221433,-0.172322\H,0,-0.798975,2.3
60708,0.21484\H,0,-0.34339,1.213616,1.488078\H,0,0.990167,1.384285,-1.
281954\H,0,1.587935,2.221642,0.15369\C,0,-0.072027,-1.38007,0.090146\C
,0,-1.236393,-1.428647,0.584646\S,0,-1.576254,0.214917,-0.318399\\Vers
ion=ES64L-G16RevA.03\State=1-A\HF=-629.3496426\MP2=-630.5049925\MP3=-6
30.5531255\MP4D=-630.5823081\MP4DQ=-630.5471543\MP4SDQ=-630.5587954\CC
SD=-630.5572349\CCSD(T)=-630.6194154\RMSD=7.105e-09\PG=C01 [X(C6H8S1)]
\\@

T1 Diagnostic = 0.01736777

Summary of Natural Population Analysis at CCSD/6-311+G**

2-(1-azacyclobutylidene)carbene (singlet 12)

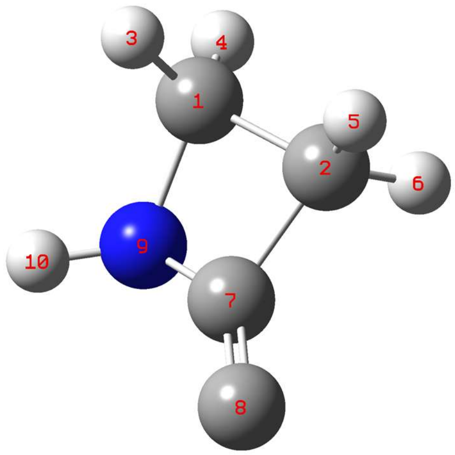

Summary of Natural Population Analysis:

Atom	No	Natural Charge	Natural Population Core	Valence	Rydberg	Total
C	1	-0.09575	1.99947	4.07623	0.02005	6.09575
C	2	-0.34013	1.99933	4.32041	0.02040	6.34013
H	3	0.15821	0.00000	0.83957	0.00222	0.84179
H	4	0.16801	0.00000	0.83034	0.00166	0.83199
H	5	0.20031	0.00000	0.79856	0.00113	0.79969
H	6	0.20019	0.00000	0.79832	0.00149	0.79981
C	7	-0.12305	1.99938	4.08428	0.03939	6.12305
C	8	0.14217	1.99953	3.83984	0.01846	5.85783
N	9	-0.67777	1.99950	5.65586	0.02240	7.67777
H	10	0.36781	0.00000	0.62966	0.00253	0.63219
* Total *		-0.00000	9.99721	25.87307	0.12972	36.00000

1-azacyclopent-2-yne (13)

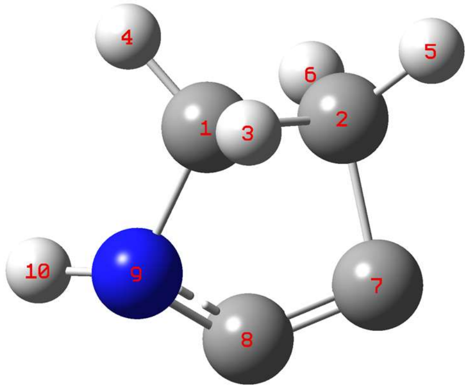

Summary of Natural Population Analysis:

Atom	No	Natural Charge	Natural Population Core	Valence	Rydberg	Total
C	1	-0.10398	1.99944	4.08147	0.02308	6.10398
C	2	-0.37685	1.99937	4.35528	0.02219	6.37685
H	3	0.17276	0.00000	0.82524	0.00199	0.82724
H	4	0.17051	0.00000	0.82761	0.00188	0.82949
H	5	0.19032	0.00000	0.80786	0.00183	0.80968
H	6	0.18955	0.00000	0.80734	0.00312	0.81045
C	7	-0.12367	1.99853	4.08263	0.04250	6.12367
C	8	0.20771	1.99836	3.74754	0.04638	5.79229
N	9	-0.70467	1.99941	5.68040	0.02487	7.70467
H	10	0.37831	0.00000	0.61925	0.00244	0.62169
* Total *		-0.00000	9.99511	25.83461	0.17028	36.00000

TS from singlet 12 to 13 by C shift

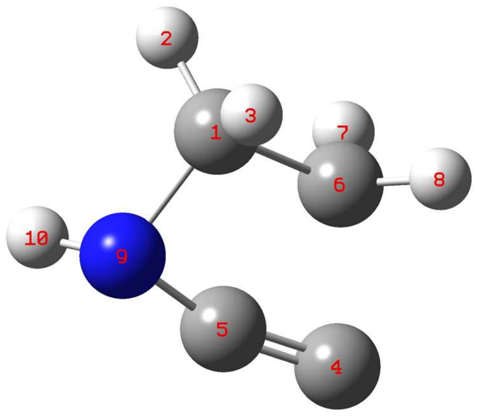

Summary of Natural Population Analysis:

Atom	No	Natural Charge	Natural Population Core	Valence	Rydberg	Total
C	1	-0.10066	1.99940	4.07556	0.02570	6.10066
H	2	0.17698	0.00000	0.82162	0.00139	0.82302
H	3	0.18134	0.00000	0.81725	0.00141	0.81866
C	4	-0.16690	1.99902	4.13773	0.03016	6.16690
C	5	0.12667	1.99881	3.83899	0.03553	5.87333
C	6	-0.28224	1.99935	4.25821	0.02468	6.28224
H	7	0.18653	0.00000	0.81122	0.00225	0.81347
H	8	0.20469	0.00000	0.79341	0.00190	0.79531
N	9	-0.69255	1.99943	5.67024	0.02288	7.69255
H	10	0.36615	0.00000	0.63068	0.00317	0.63385
* Total *		0.00000	9.99602	25.85491	0.14907	36.00000

TS from singlet 12 to 13 by C shift

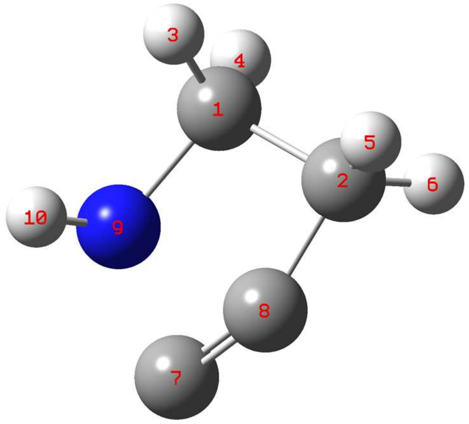

Summary of Natural Population Analysis:

Atom	No	Natural Charge	Natural Population Core	Valence	Rydberg	Total
C	1	-0.09385	1.99942	4.07024	0.02418	6.09385
C	2	-0.42249	1.99921	4.40064	0.02264	6.42249
H	3	0.17677	0.00000	0.82183	0.00141	0.82323
H	4	0.18189	0.00000	0.81695	0.00116	0.81811
H	5	0.19629	0.00000	0.80225	0.00146	0.80371
H	6	0.20481	0.00000	0.79345	0.00174	0.79519
C	7	-0.01495	1.99896	3.97353	0.04245	6.01495
C	8	0.07640	1.99908	3.89384	0.03068	5.92360
N	9	-0.66347	1.99963	5.64105	0.02279	7.66347
H	10	0.35861	0.00000	0.63837	0.00302	0.64139
* Total *		-0.00000	9.99631	25.85215	0.15153	36.00000

2-(1-oxacyclobutylidene)carbene (singlet 14)

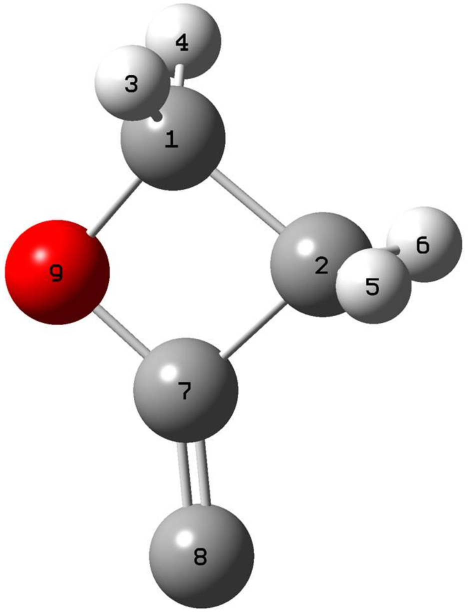

Summary of Natural Population Analysis:

Atom	No	Natural Charge	Natural Population Core	Valence	Rydberg	Total
C	1	0.06787	1.99941	3.91137	0.02135	5.93213
C	2	-0.38817	1.99934	4.37187	0.01696	6.38817
H	3	0.16147	0.00000	0.83665	0.00188	0.83853
H	4	0.16147	0.00000	0.83665	0.00188	0.83853
H	5	0.20460	0.00000	0.79418	0.00123	0.79540
H	6	0.20460	0.00000	0.79418	0.00123	0.79540
C	7	0.04808	1.99930	3.91236	0.04026	5.95192
C	8	0.14970	1.99954	3.83264	0.01811	5.85030
O	9	-0.60963	1.99978	6.58809	0.02176	8.60963
* Total *		-0.00000	9.99739	25.87798	0.12463	36.00000

1-oxacyclopent-2-yne (15)

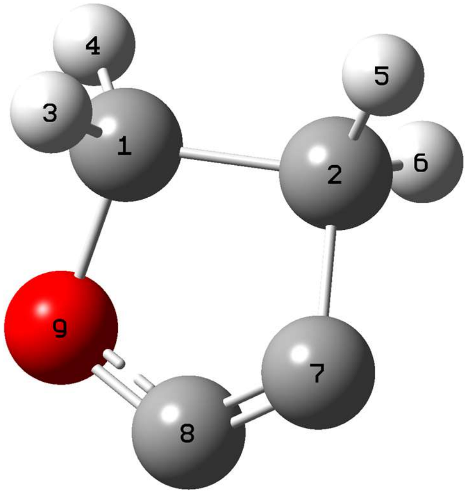

Summary of Natural Population Analysis:

Atom	No	Natural Charge	Natural Population Core	Valence	Rydberg	Total
C	1	0.05958	1.99932	3.91681	0.02429	5.94042
C	2	-0.36548	1.99935	4.34485	0.02128	6.36548
H	3	0.16156	0.00000	0.83647	0.00198	0.83844
H	4	0.16872	0.00000	0.82951	0.00176	0.83128
H	5	0.19611	0.00000	0.80198	0.00190	0.80389
H	6	0.19556	0.00000	0.80142	0.00302	0.80444
C	7	-0.20376	1.99821	4.16377	0.04178	6.20376
C	8	0.39654	1.99788	3.55611	0.04946	5.60346
O	9	-0.60884	1.99976	6.58814	0.02094	8.60884
* Total *		0.00000	9.99452	25.83908	0.16640	36.00000

TS from singlet 14 to 15 by C shift

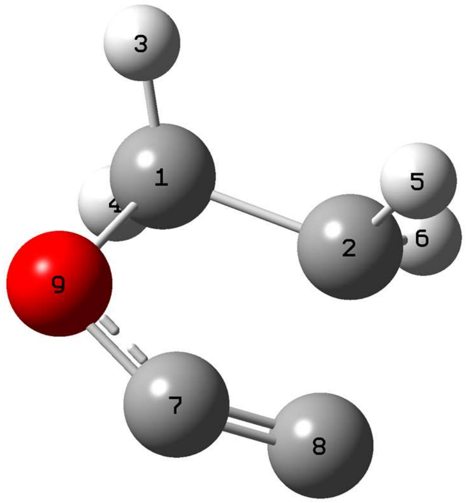

Summary of Natural Population Analysis:

Atom	No	Natural Charge	Natural Population Core	Valence	Rydberg	Total
C	1	0.05729	1.99930	3.92096	0.02245	5.94271
C	2	-0.31670	1.99935	4.29114	0.02622	6.31670
H	3	0.17370	0.00000	0.82447	0.00183	0.82630
H	4	0.16826	0.00000	0.82984	0.00190	0.83174
H	5	0.19116	0.00000	0.80630	0.00254	0.80884
H	6	0.20153	0.00000	0.79669	0.00178	0.79847
C	7	0.49604	1.99820	3.45944	0.04632	5.50396
C	8	-0.34730	1.99849	4.31203	0.03678	6.34730
O	9	-0.62397	1.99973	6.60284	0.02141	8.62397
* Total *		0.00000	9.99506	25.84371	0.16123	36.00000

TS from singlet 14 to 15 by O shift

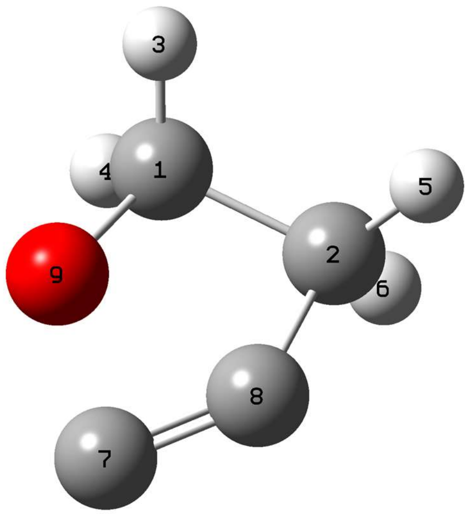

Summary of Natural Population Analysis:

Atom	No	Natural Charge	Natural Population Core	Valence	Rydberg	Total
C	1	0.06853	1.99938	3.90929	0.02280	5.93147
C	2	-0.45450	1.99924	4.43298	0.02228	6.45450
H	3	0.17535	0.00000	0.82273	0.00192	0.82465
H	4	0.16522	0.00000	0.83289	0.00189	0.83478
H	5	0.19952	0.00000	0.79883	0.00165	0.80048
H	6	0.21249	0.00000	0.78569	0.00182	0.78751
C	7	0.13188	1.99888	3.82868	0.04056	5.86812
C	8	0.13931	1.99915	3.82738	0.03416	5.86069
O	9	-0.63782	1.99986	6.62115	0.01681	8.63782
* Total *		0.00000	9.99651	25.85962	0.14387	36.00000

2-(1-thiocyclobutylidene)carbene (singlet 16)

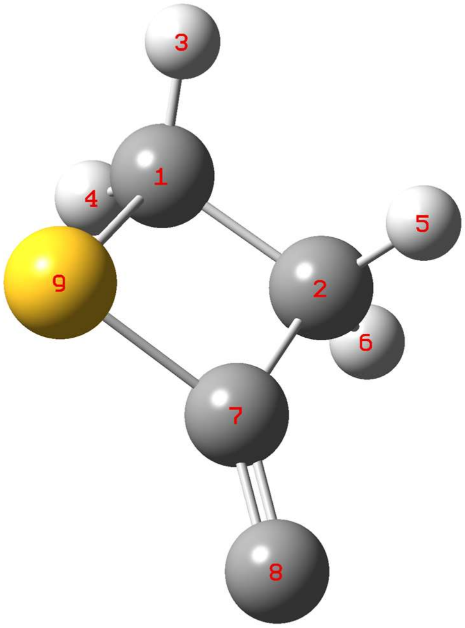

Summary of Natural Population Analysis:

Atom	No	Natural Charge	Natural Population Core	Valence	Rydberg	Total
C	1	-0.42067	1.99938	4.40135	0.01994	6.42067
C	2	-0.33528	1.99933	4.31318	0.02277	6.33528
H	3	0.19087	0.00000	0.80704	0.00209	0.80913
H	4	0.18976	0.00000	0.80776	0.00248	0.81024
H	5	0.20511	0.00000	0.79357	0.00133	0.79489
H	6	0.20284	0.00000	0.79580	0.00136	0.79716
C	7	-0.51263	1.99927	4.46734	0.04603	6.51263
C	8	0.22115	1.99950	3.75649	0.02287	5.77885
S	9	0.25885	9.99919	5.70034	0.04161	15.74115
* Total *		0.00000	17.99666	25.84286	0.16048	44.00000

1-thiocyclopent-2-yne (17)

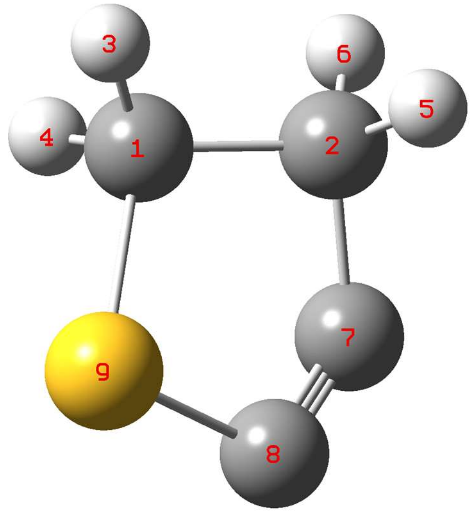

Summary of Natural Population Analysis:

Atom	No	Natural Charge	Natural Population Core	Valence	Rydberg	Total
C	1	-0.43016	1.99934	4.40908	0.02175	6.43016
C	2	-0.38606	1.99925	4.36060	0.02621	6.38606
H	3	0.19250	0.00000	0.80539	0.00210	0.80750
H	4	0.18936	0.00000	0.80807	0.00257	0.81064
H	5	0.19832	0.00000	0.79913	0.00256	0.80168
H	6	0.20095	0.00000	0.79768	0.00137	0.79905
C	7	0.03063	1.99792	3.93676	0.03469	5.96937
C	8	-0.29128	1.99775	4.25126	0.04227	6.29128
S	9	0.29574	9.99911	5.65533	0.04981	15.70426
* Total *		-0.00000	17.99337	25.82330	0.18333	44.00000

TS from singlet 16 to 17 by C shift

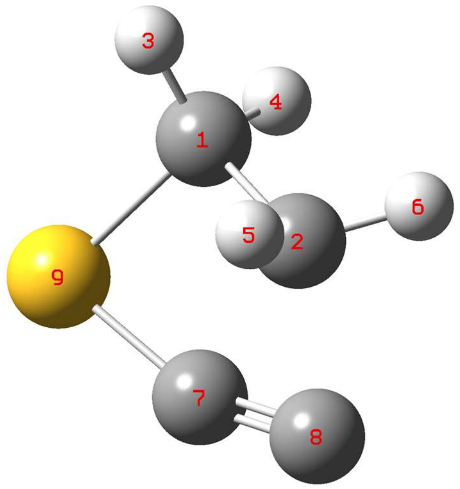

Summary of Natural Population Analysis:

Atom	No	Natural Charge	Natural Population Core	Valence	Rydberg	Total
C	1	-0.42278	1.99929	4.39609	0.02740	6.42278
C	2	-0.27629	1.99931	4.24878	0.02820	6.27629
H	3	0.20167	0.00000	0.79665	0.00168	0.79833
H	4	0.19763	0.00000	0.80004	0.00233	0.80237
H	5	0.19291	0.00000	0.80472	0.00237	0.80709
H	6	0.21469	0.00000	0.78362	0.00169	0.78531
C	7	-0.20461	1.99856	4.16847	0.03759	6.20461
C	8	-0.17745	1.99893	4.14234	0.03619	6.17745
S	9	0.27423	9.99890	5.68170	0.04517	15.72577
* Total *		-0.00000	17.99498	25.82240	0.18261	44.00000

TS from singlet 16 to 17 by S shift

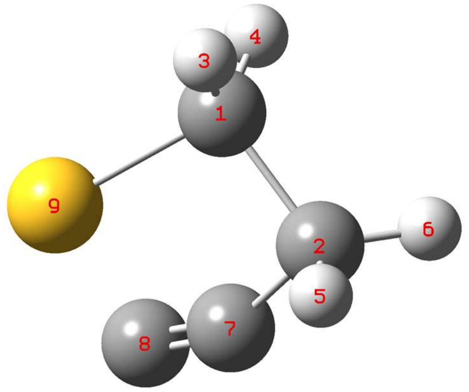

Summary of Natural Population Analysis:

Atom	No	Natural Charge	Natural Population Core	Valence	Rydberg	Total
C	1	-0.41344	1.99936	4.39304	0.02104	6.41344
C	2	-0.39202	1.99920	4.36655	0.02627	6.39202
H	3	0.19842	0.00000	0.79928	0.00230	0.80158
H	4	0.19551	0.00000	0.80183	0.00267	0.80449
H	5	0.20854	0.00000	0.78946	0.00200	0.79146
H	6	0.21324	0.00000	0.78523	0.00153	0.78676
C	7	-0.15535	1.99904	4.11607	0.04025	6.15535
C	8	-0.12017	1.99902	4.08707	0.03408	6.12017
S	9	0.26528	9.99941	5.69244	0.04288	15.73472
* Total *		0.00000	17.99603	25.83096	0.17301	44.00000

2-(1-azacyclopentylidene)carbene (singlet 18)

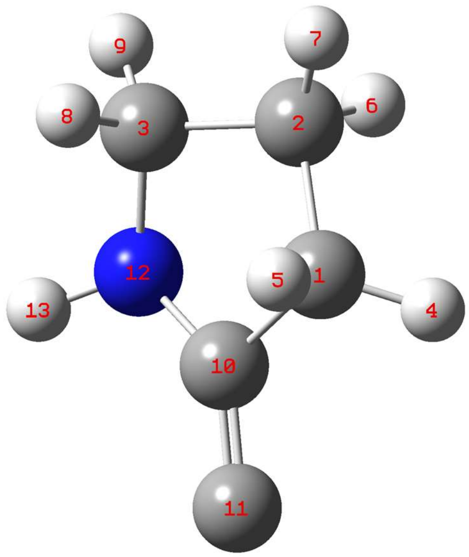

Summary of Natural Population Analysis:

Atom	No	Natural Charge	Core	Valence	Rydberg	Total
			Natural Population			
C	1	-0.35248	1.99934	4.33545	0.01769	6.35248
C	2	-0.34450	1.99941	4.32742	0.01767	6.34450
C	3	-0.10544	1.99942	4.08546	0.02056	6.10544
H	4	0.20579	0.00000	0.79268	0.00152	0.79421
H	5	0.19244	0.00000	0.80570	0.00186	0.80756
H	6	0.18404	0.00000	0.81376	0.00220	0.81596
H	7	0.18783	0.00000	0.81055	0.00162	0.81217
H	8	0.14659	0.00000	0.85123	0.00219	0.85341
H	9	0.17607	0.00000	0.82250	0.00143	0.82393
C	10	-0.09843	1.99932	4.05955	0.03956	6.09843
C	11	0.12833	1.99956	3.85607	0.01604	5.87167
N	12	-0.69281	1.99947	5.67165	0.02169	7.69281
H	13	0.37255	0.00000	0.62343	0.00402	0.62745

* Total *		0.00000	11.99651	31.85544	0.14805	44.00000

1-azacyclohex-2-yne (19)

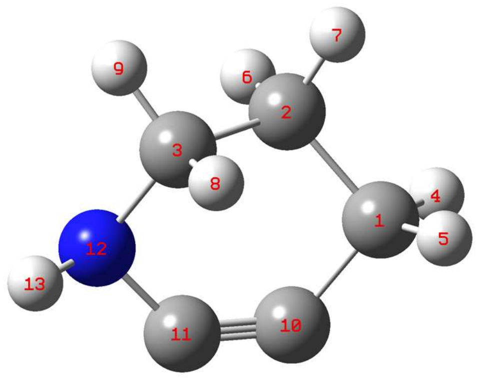

Summary of Natural Population Analysis:

Atom	No	Natural Charge	Natural Population Core	Valence	Rydberg	Total
C	1	-0.36082	1.99929	4.34020	0.02133	6.36082
C	2	-0.33950	1.99939	4.32210	0.01802	6.33950
C	3	-0.10498	1.99937	4.08397	0.02164	6.10498
H	4	0.19081	0.00000	0.80715	0.00203	0.80919
H	5	0.18724	0.00000	0.81042	0.00233	0.81276
H	6	0.18513	0.00000	0.81262	0.00224	0.81487
H	7	0.18718	0.00000	0.81122	0.00160	0.81282
H	8	0.15111	0.00000	0.84669	0.00220	0.84889
H	9	0.17796	0.00000	0.82050	0.00155	0.82204
C	10	-0.09659	1.99791	4.06863	0.03005	6.09659
C	11	0.16100	1.99786	3.80691	0.03423	5.83900
N	12	-0.70416	1.99947	5.67800	0.02669	7.70416
H	13	0.36562	0.00000	0.63164	0.00274	0.63438
* Total *		-0.00000	11.99328	31.84006	0.16665	44.00000

TS from singlet 18 to 19 by C shift

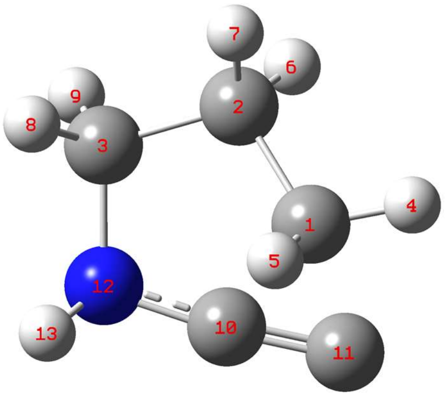

Summary of Natural Population Analysis:

Atom	No	Natural Charge	Natural Population Core	Valence	Rydberg	Total
C	1	-0.30526	1.99930	4.27856	0.02740	6.30526
C	2	-0.37486	1.99937	4.35403	0.02146	6.37486
C	3	-0.10526	1.99940	4.08512	0.02074	6.10526
H	4	0.23890	0.00000	0.75968	0.00142	0.76110
H	5	0.17802	0.00000	0.81975	0.00223	0.82198
H	6	0.19108	0.00000	0.80718	0.00174	0.80892
H	7	0.19352	0.00000	0.80506	0.00142	0.80648
H	8	0.16641	0.00000	0.83171	0.00188	0.83359
H	9	0.17578	0.00000	0.82252	0.00170	0.82422
C	10	0.06713	1.99892	3.89703	0.03692	5.93287
C	11	-0.10788	1.99916	4.08122	0.02749	6.10788
N	12	-0.69384	1.99938	5.67111	0.02335	7.69384
H	13	0.37625	0.00000	0.62085	0.00289	0.62375
* Total *		0.00000	11.99553	31.83382	0.17065	44.00000

TS from singlet 18 to 19 by N shift

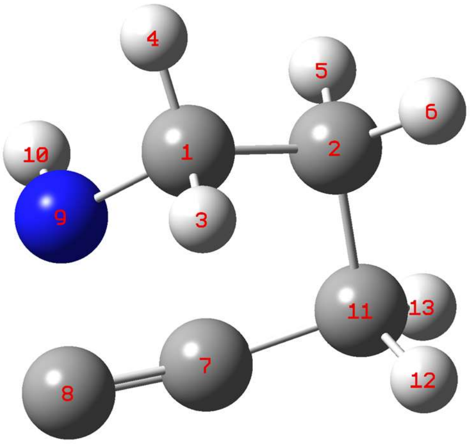

Summary of Natural Population Analysis:

Atom	No	Natural Charge	Natural Population Core	Valence	Rydberg	Total
C	1	-0.13071	1.99939	4.10157	0.02976	6.13071
C	2	-0.32809	1.99940	4.31271	0.01598	6.32809
H	3	0.17723	0.00000	0.82117	0.00160	0.82277
H	4	0.18506	0.00000	0.81375	0.00119	0.81494
H	5	0.17478	0.00000	0.82268	0.00254	0.82522
H	6	0.18995	0.00000	0.80805	0.00200	0.81005
C	7	0.11276	1.99909	3.86644	0.02171	5.88724
C	8	-0.06945	1.99880	4.02555	0.04511	6.06945
N	9	-0.64544	1.99960	5.62069	0.02515	7.64544
H	10	0.35302	0.00000	0.64391	0.00307	0.64698
C	11	-0.43249	1.99922	4.40827	0.02500	6.43249
H	12	0.20673	0.00000	0.79126	0.00201	0.79327
H	13	0.20665	0.00000	0.79185	0.00151	0.79335
* Total *		0.00000	11.99549	31.82790	0.17661	44.00000

2-(1-oxacyclopentylidene)carbene (singlet 20)

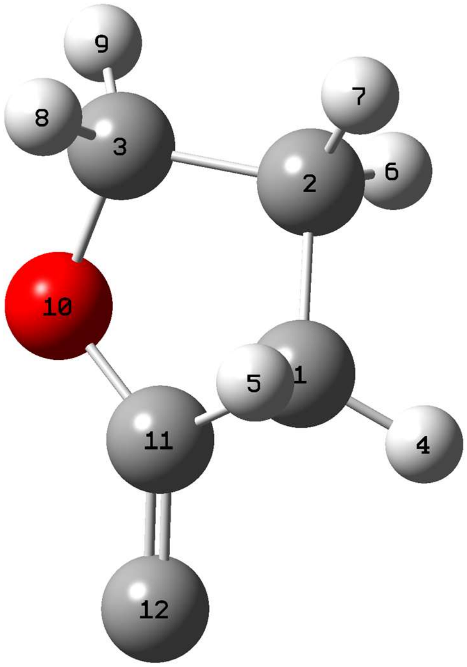

Summary of Natural Population Analysis:

Atom	No	Natural Charge	Natural Population Core	Valence	Rydberg	Total
C	1	-0.37788	1.99935	4.36165	0.01688	6.37788
C	2	-0.36625	1.99941	4.35111	0.01573	6.36625
C	3	0.05505	1.99931	3.92312	0.02252	5.94495
H	4	0.21180	0.00000	0.78671	0.00149	0.78820
H	5	0.19363	0.00000	0.80453	0.00183	0.80637
H	6	0.18585	0.00000	0.81228	0.00187	0.81415
H	7	0.19386	0.00000	0.80444	0.00170	0.80614

H	8	0.14917	0.00000	0.84885	0.00198	0.85083
H	9	0.17096	0.00000	0.82742	0.00162	0.82904
O	10	-0.61973	1.99975	6.59634	0.02364	8.61973
C	11	0.05911	1.99916	3.89858	0.04316	5.94089
C	12	0.14443	1.99956	3.84070	0.01530	5.85557
* Total *		-0.00000	11.99654	31.85574	0.14771	44.00000

1-oxacyclohex-2-yne (21)

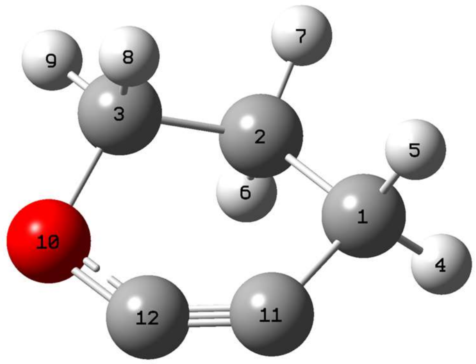

Summary of Natural Population Analysis:

Atom	No	Natural Charge	Natural Population Core	Valence	Rydberg	Total
C	1	-0.34883	1.99930	4.32801	0.02152	6.34883
C	2	-0.37076	1.99939	4.35485	0.01652	6.37076
C	3	0.05583	1.99924	3.92155	0.02338	5.94417
H	4	0.19841	0.00000	0.79951	0.00208	0.80159
H	5	0.18901	0.00000	0.80875	0.00224	0.81099
H	6	0.18523	0.00000	0.81261	0.00216	0.81477
H	7	0.19168	0.00000	0.80662	0.00171	0.80832
H	8	0.15909	0.00000	0.83894	0.00197	0.84091
H	9	0.17528	0.00000	0.82311	0.00161	0.82472
O	10	-0.60875	1.99975	6.58510	0.02390	8.60875
C	11	-0.21336	1.99789	4.18181	0.03366	6.21336
C	12	0.38717	1.99761	3.57961	0.03561	5.61283
* Total *		0.00000	11.99318	31.84047	0.16635	44.00000

TS from singlet 20 to 21 by C shift

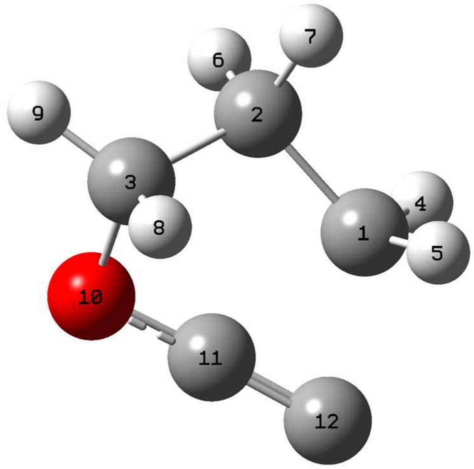

Summary of Natural Population Analysis:

Atom	No	Natural Charge	Natural Population Core	Valence	Rydberg	Total
C	1	-0.29500	1.99928	4.26960	0.02612	6.29500
C	2	-0.38305	1.99937	4.36298	0.02070	6.38305
C	3	0.05363	1.99927	3.92044	0.02666	5.94637
H	4	0.20565	0.00000	0.79265	0.00170	0.79435
H	5	0.19748	0.00000	0.80063	0.00189	0.80252
H	6	0.19156	0.00000	0.80670	0.00173	0.80844
H	7	0.20224	0.00000	0.79650	0.00127	0.79776
H	8	0.16187	0.00000	0.83648	0.00165	0.83813
H	9	0.17832	0.00000	0.82062	0.00106	0.82168
O	10	-0.59710	1.99970	6.57619	0.02121	8.59710
C	11	0.26309	1.99862	3.70269	0.03560	5.73691
C	12	-0.17869	1.99906	4.14851	0.03112	6.17869

* Total *	0.00000	11.99530	31.83399	0.17070	44.00000

TS from singlet 20 to 21 by O shift

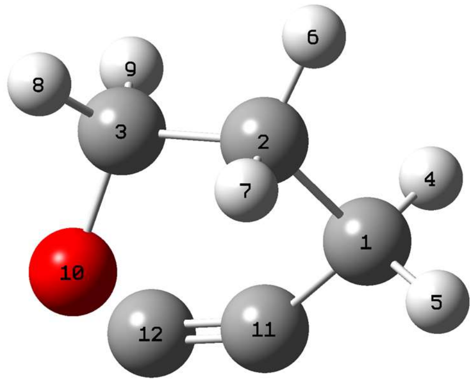

Summary of Natural Population Analysis:

Atom	No	Natural Charge	Natural Population Core	Valence	Rydberg	Total
C	1	-0.44473	1.99923	4.42103	0.02447	6.44473
C	2	-0.33836	1.99940	4.32346	0.01550	6.33836
C	3	0.03688	1.99933	3.93429	0.02950	5.96312
H	4	0.21477	0.00000	0.78324	0.00199	0.78523
H	5	0.20351	0.00000	0.79493	0.00155	0.79649
H	6	0.19200	0.00000	0.80616	0.00184	0.80800
H	7	0.18860	0.00000	0.80923	0.00217	0.81140
H	8	0.17932	0.00000	0.81960	0.00109	0.82068
H	9	0.15574	0.00000	0.84224	0.00203	0.84426
O	10	-0.63149	1.99984	6.61009	0.02156	8.63149
C	11	0.17628	1.99914	3.79979	0.02480	5.82372
C	12	0.06748	1.99871	3.89456	0.03925	5.93252
* Total *		0.00000	11.99563	31.83861	0.16575	44.00000

2-(1-thiocyclopentylidene)carbene (singlet 22)

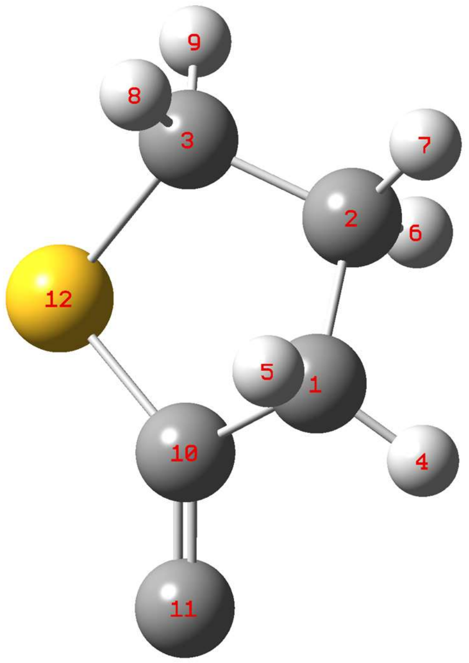

Summary of Natural Population Analysis:

Atom	No	Natural Charge	Natural Population Core	Valence	Rydberg	Total
C	1	-0.33964	1.99931	4.31667	0.02366	6.33964
C	2	-0.34383	1.99939	4.32216	0.02228	6.34383
C	3	-0.44538	1.99933	4.42570	0.02036	6.44538
H	4	0.20815	0.00000	0.79040	0.00145	0.79185
H	5	0.19323	0.00000	0.80523	0.00154	0.80677

H	6	0.18787	0.00000	0.81052	0.00162	0.81213
H	7	0.19260	0.00000	0.80601	0.00139	0.80740
H	8	0.18489	0.00000	0.81258	0.00253	0.81511
H	9	0.20153	0.00000	0.79661	0.00186	0.79847
C	10	-0.51341	1.99916	4.47080	0.04345	6.51341
C	11	0.21621	1.99953	3.76222	0.02204	5.78379
S	12	0.25780	9.99916	5.69968	0.04336	15.74220
* Total *		-0.00000	19.99588	31.81857	0.18555	52.00000

1-thiocyclohex-2-yne (23)

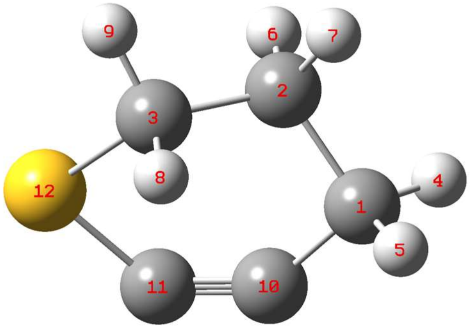

Summary of Natural Population Analysis:

Atom	No	Natural Charge	Natural Population Core	Valence	Rydberg	Total
C	1	-0.38775	1.99927	4.36358	0.02490	6.38775
C	2	-0.33230	1.99937	4.31493	0.01799	6.33230
C	3	-0.45426	1.99928	4.43270	0.02228	6.45426
H	4	0.20638	0.00000	0.79203	0.00159	0.79362
H	5	0.19414	0.00000	0.80404	0.00183	0.80586
H	6	0.18726	0.00000	0.81062	0.00212	0.81274
H	7	0.19018	0.00000	0.80810	0.00172	0.80982
H	8	0.18730	0.00000	0.81016	0.00255	0.81270
H	9	0.19934	0.00000	0.79887	0.00179	0.80066
C	10	0.00983	1.99808	3.96519	0.02689	5.99017
C	11	-0.28796	1.99777	4.25648	0.03371	6.28796
S	12	0.28785	9.99902	5.66120	0.05193	15.71215

* Total *		-0.00000	19.99280	31.81790	0.18930	52.00000

TS from singlet 22 to 23 by C shift

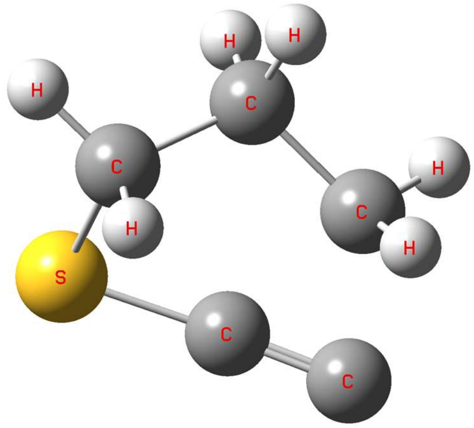

Summary of Natural Population Analysis:

Atom	No	Natural Charge	Natural Population Core	Valence	Rydberg	Total
C	1	-0.27514	1.99925	4.24850	0.02738	6.27514
C	2	-0.36247	1.99935	4.33940	0.02372	6.36247
C	3	-0.43627	1.99931	4.41479	0.02217	6.43627
H	4	0.20752	0.00000	0.79091	0.00157	0.79248
H	5	0.19926	0.00000	0.79893	0.00181	0.80074
H	6	0.19216	0.00000	0.80597	0.00186	0.80784
H	7	0.19852	0.00000	0.80008	0.00140	0.80148

H	8	0.19453	0.00000	0.80274	0.00272	0.80547
H	9	0.20332	0.00000	0.79501	0.00167	0.79668
C	10	-0.33682	1.99868	4.30061	0.03752	6.33682
C	11	-0.09309	1.99912	4.06144	0.03252	6.09309
S	12	0.30846	9.99889	5.64602	0.04663	15.69154
* Total *		-0.00000	19.99460	31.80441	0.20099	52.00000

TS from singlet 22 to 23 by S shift

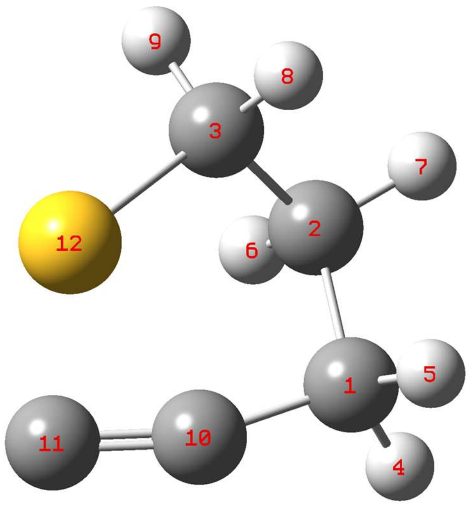

Summary of Natural Population Analysis:

		Natural	Natural Population			
Atom	No	Charge	Core	Valence	Rydberg	Total
C	1	-0.42710	1.99918	4.40268	0.02523	6.42710
C	2	-0.33231	1.99938	4.31534	0.01758	6.33231
C	3	-0.45789	1.99929	4.43770	0.02090	6.45789
H	4	0.23355	0.00000	0.76504	0.00140	0.76645

H	5	0.20887	0.00000	0.78934	0.00179	0.79113
H	6	0.18897	0.00000	0.80919	0.00185	0.81103
H	7	0.18973	0.00000	0.80840	0.00187	0.81027
H	8	0.18937	0.00000	0.80834	0.00229	0.81063
H	9	0.19938	0.00000	0.79874	0.00188	0.80062
C	10	-0.00565	1.99901	3.97209	0.03455	6.00565
C	11	-0.24785	1.99875	4.20854	0.04055	6.24785
S	12	0.26091	9.99936	5.69456	0.04517	15.73909
* Total *		0.00000	19.99498	31.80996	0.19506	52.00000

2-(1-azacyclohexylidene)carbene (singlet 24)

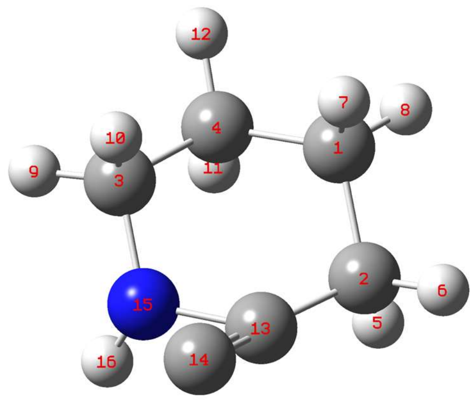

Summary of Natural Population Analysis:

Atom	No	Natural Charge	Natural Population: Core	Valence	Rydberg	Total
C	1	-0.32565	1.99940	4.30860	0.01766	6.32565
C	2	-0.37010	1.99932	4.35178	0.01900	6.37010
C	3	-0.10636	1.99939	4.07869	0.02829	6.10636
C	4	-0.35070	1.99940	4.33437	0.01694	6.35070
H	5	0.18380	0.00000	0.81364	0.00256	0.81620
H	6	0.20227	0.00000	0.79600	0.00173	0.79773
H	7	0.17534	0.00000	0.82240	0.00226	0.82466

H	8	0.17812	0.00000	0.82030	0.00158	0.82188
H	9	0.17418	0.00000	0.82452	0.00131	0.82582
H	10	0.17855	0.00000	0.81966	0.00179	0.82145
H	11	0.16522	0.00000	0.83206	0.00272	0.83478
H	12	0.18206	0.00000	0.81625	0.00169	0.81794
C	13	-0.09567	1.99912	4.05392	0.04264	6.09567
C	14	0.07108	1.99936	3.91118	0.01838	5.92892
N	15	-0.63637	1.99949	5.61288	0.02400	7.63637
H	16	0.37422	0.00000	0.62335	0.00243	0.62578
* Total *		-0.00000	13.99547	37.81957	0.18497	52.00000

1-azacyclohepty-2-yne (25)

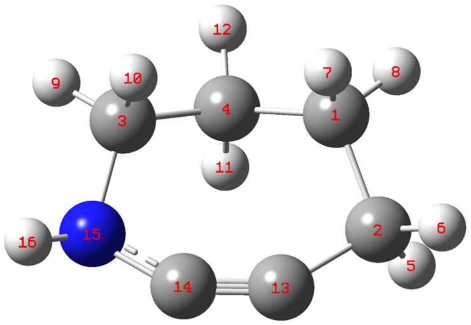

Summary of Natural Population Analysis:

		Natural	Natural Population			
Atom	No	Charge	Core	Valence	Rydberg	Total
C	1	-0.31385	1.99939	4.29590	0.01856	6.31385
C	2	-0.37931	1.99928	4.35882	0.02120	6.37931
C	3	-0.09058	1.99936	4.06909	0.02213	6.09058
C	4	-0.35741	1.99938	4.33912	0.01891	6.35741
H	5	0.18722	0.00000	0.81023	0.00255	0.81278
H	6	0.19377	0.00000	0.80431	0.00192	0.80623
H	7	0.17103	0.00000	0.82669	0.00227	0.82897
H	8	0.17443	0.00000	0.82394	0.00164	0.82557

H	9	0.16757	0.00000	0.83087	0.00155	0.83243
H	10	0.14680	0.00000	0.85073	0.00247	0.85320
H	11	0.18677	0.00000	0.81071	0.00252	0.81323
H	12	0.18071	0.00000	0.81781	0.00148	0.81929
C	13	-0.09514	1.99815	4.07575	0.02124	6.09514
C	14	0.16538	1.99805	3.81121	0.02536	5.83462
N	15	-0.70774	1.99945	5.68134	0.02695	7.70774
H	16	0.37036	0.00000	0.62702	0.00262	0.62964
* Total *		0.00000	13.99307	37.83354	0.17339	52.00000

TS from singlet 24 to 25 by C shift

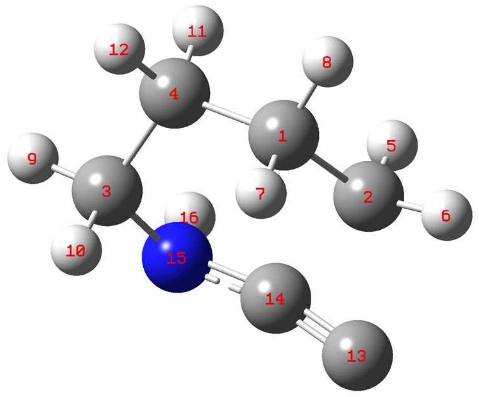

Summary of Natural Population Analysis:

Atom	No	Natural Charge	Natural Population Core	Valence	Rydberg	Total
C	1	-0.35285	1.99935	4.33006	0.02344	6.35285
C	2	-0.32440	1.99925	4.30156	0.02359	6.32440
C	3	-0.10114	1.99936	4.07930	0.02248	6.10114

C	4	-0.35251	1.99940	4.33575	0.01737	6.35251
H	5	0.17715	0.00000	0.82024	0.00261	0.82285
H	6	0.24164	0.00000	0.75656	0.00180	0.75836
H	7	0.18554	0.00000	0.81250	0.00196	0.81446
H	8	0.18382	0.00000	0.81476	0.00141	0.81618
H	9	0.17380	0.00000	0.82466	0.00154	0.82620
H	10	0.17334	0.00000	0.82449	0.00217	0.82666
H	11	0.16455	0.00000	0.83297	0.00248	0.83545
H	12	0.18353	0.00000	0.81477	0.00170	0.81647
C	13	-0.06812	1.99924	4.04302	0.02586	6.06812
C	14	0.04056	1.99892	3.92315	0.03737	5.95944
N	15	-0.69763	1.99938	5.67105	0.02720	7.69763
H	16	0.37272	0.00000	0.62434	0.00294	0.62728
* Total *		0.00000	13.99490	37.80918	0.19592	52.00000

TS from singlet 24 to 25 by N shift

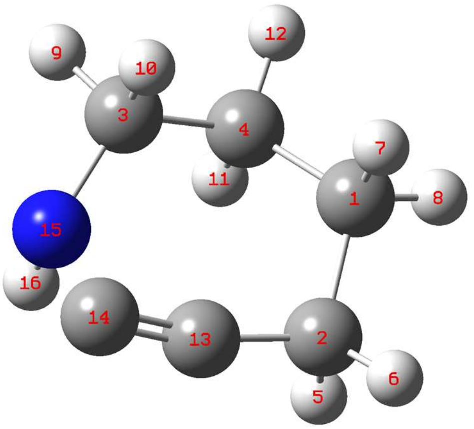

Summary of Natural Population Analysis:

Atom	No	Natural Charge	Natural Population Core	Valence	Rydberg	Total
C	1	-0.31989	1.99938	4.30273	0.01777	6.31989
C	2	-0.44485	1.99922	4.42045	0.02518	6.44485

C	3	-0.12284	1.99937	4.09260	0.03086	6.12284
C	4	-0.35731	1.99939	4.34030	0.01762	6.35731
H	5	0.19800	0.00000	0.79980	0.00220	0.80200
H	6	0.23450	0.00000	0.76412	0.00137	0.76550
H	7	0.17988	0.00000	0.81795	0.00217	0.82012
H	8	0.18154	0.00000	0.81683	0.00163	0.81846
H	9	0.17462	0.00000	0.82419	0.00120	0.82538
H	10	0.17663	0.00000	0.82143	0.00194	0.82337
H	11	0.16333	0.00000	0.83398	0.00269	0.83667
H	12	0.18422	0.00000	0.81395	0.00183	0.81578
C	13	0.14471	1.99909	3.83436	0.02183	5.85529
C	14	-0.09288	1.99877	4.04825	0.04586	6.09288
N	15	-0.65354	1.99958	5.62901	0.02496	7.65354
H	16	0.35388	0.00000	0.64292	0.00320	0.64612
* Total *		-0.00000	13.99480	37.80289	0.20231	52.00000

2-(1-oxacyclohexylidene)carbene (singlet 26)

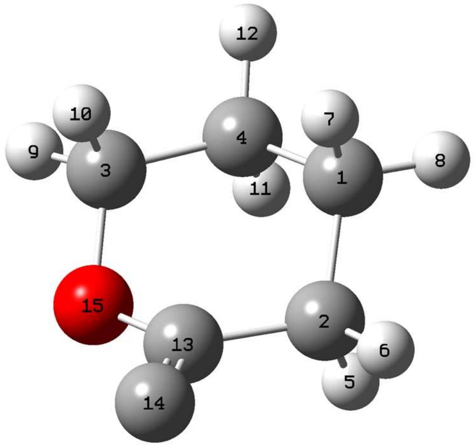

Summary of Natural Population Analysis:

Atom	No	Natural Charge	Natural Population Core	Valence	Rydberg	Total
C	1	-0.33343	1.99939	4.31646	0.01758	6.33343
C	2	-0.38321	1.99932	4.36061	0.02327	6.38321

C	3	0.05869	1.99926	3.91864	0.02341	5.94131
C	4	-0.36839	1.99940	4.35253	0.01646	6.36839
H	5	0.19217	0.00000	0.80586	0.00197	0.80783
H	6	0.20352	0.00000	0.79512	0.00137	0.79648
H	7	0.17570	0.00000	0.82210	0.00221	0.82430
H	8	0.18313	0.00000	0.81518	0.00169	0.81687
H	9	0.16927	0.00000	0.82918	0.00156	0.83073
H	10	0.14733	0.00000	0.85051	0.00216	0.85267
H	11	0.17723	0.00000	0.82057	0.00220	0.82277
H	12	0.18623	0.00000	0.81211	0.00166	0.81377
C	13	0.04757	1.99908	3.90712	0.04624	5.95243
C	14	0.18157	1.99956	3.80429	0.01458	5.81843
O	15	-0.63740	1.99975	6.60912	0.02853	8.63740
* Total *		0.00000	13.99575	37.81938	0.18486	52.00000

1-oxacyclohepty-2-yne (27)

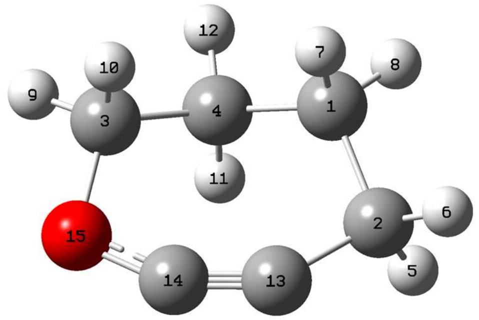

Summary of Natural Population Analysis:

Atom	No	Natural Charge	Natural Population: Core	Valence	Rydberg	Total
C	1	-0.31815	1.99940	4.29991	0.01884	6.31815
C	2	-0.36768	1.99929	4.34698	0.02141	6.36768
C	3	0.06675	1.99922	3.91006	0.02396	5.93325

C	4	-0.38010	1.99937	4.36329	0.01743	6.38010
H	5	0.18893	0.00000	0.80855	0.00252	0.81107
H	6	0.19015	0.00000	0.80788	0.00197	0.80985
H	7	0.17232	0.00000	0.82544	0.00224	0.82768
H	8	0.17612	0.00000	0.82223	0.00165	0.82388
H	9	0.16752	0.00000	0.83088	0.00160	0.83248
H	10	0.15463	0.00000	0.84319	0.00217	0.84537
H	11	0.18813	0.00000	0.80950	0.00237	0.81187
H	12	0.18800	0.00000	0.81047	0.00153	0.81200
C	13	-0.19148	1.99805	4.17199	0.02144	6.19148
C	14	0.37022	1.99773	3.59980	0.03225	5.62978
O	15	-0.60537	1.99974	6.58248	0.02315	8.60537
* Total *		0.00000	13.99279	37.83266	0.17455	52.00000

TS from singlet 26 to 27 by C shift

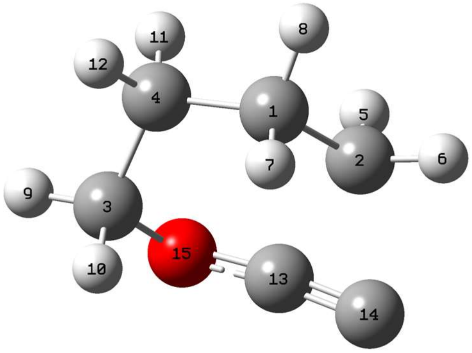

Summary of Natural Population Analysis:

Atom	No	Natural Charge	Natural Population Core	Valence	Rydberg	Total
C	1	-0.35667	1.99935	4.33426	0.02306	6.35667
C	2	-0.32392	1.99924	4.29621	0.02847	6.32392

C	3	0.06052	1.99922	3.91746	0.02280	5.93948
C	4	-0.37046	1.99939	4.35359	0.01748	6.37046
H	5	0.18659	0.00000	0.81115	0.00226	0.81341
H	6	0.24264	0.00000	0.75596	0.00140	0.75736
H	7	0.18778	0.00000	0.81030	0.00192	0.81222
H	8	0.18664	0.00000	0.81190	0.00146	0.81336
H	9	0.17261	0.00000	0.82585	0.00154	0.82739
H	10	0.16155	0.00000	0.83622	0.00223	0.83845
H	11	0.17915	0.00000	0.81849	0.00236	0.82085
H	12	0.18929	0.00000	0.80901	0.00170	0.81071
C	13	0.26316	1.99863	3.70021	0.03800	5.73684
C	14	-0.17701	1.99911	4.14796	0.02994	6.17701
O	15	-0.60186	1.99969	6.57727	0.02490	8.60186
* Total *		-0.00000	13.99463	37.80585	0.19952	52.00000

TS from singlet 26 to 27 by O shift

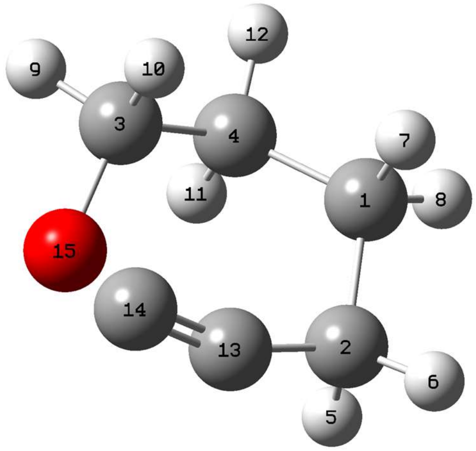

Summary of Natural Population Analysis:

Natural Population

Atom	No	Natural Charge	Core	Valence	Rydberg	Total
C	1	-0.31963	1.99939	4.30307	0.01717	6.31963
C	2	-0.46222	1.99922	4.43843	0.02457	6.46222
C	3	0.04823	1.99931	3.92586	0.02661	5.95177
C	4	-0.36933	1.99939	4.35278	0.01716	6.36933
H	5	0.20936	0.00000	0.78850	0.00214	0.79064
H	6	0.23976	0.00000	0.75888	0.00136	0.76024
H	7	0.17872	0.00000	0.81913	0.00216	0.82128
H	8	0.18240	0.00000	0.81594	0.00166	0.81760
H	9	0.16684	0.00000	0.83157	0.00158	0.83316
H	10	0.15374	0.00000	0.84357	0.00270	0.84626
H	11	0.17786	0.00000	0.81979	0.00236	0.82214
H	12	0.18453	0.00000	0.81380	0.00167	0.81547
C	13	0.22192	1.99916	3.75563	0.02329	5.77808
C	14	0.02203	1.99865	3.93911	0.04021	5.97797
O	15	-0.63419	1.99983	6.61157	0.02279	8.63419
* Total *		0.00000	13.99494	37.81763	0.18743	52.00000

2-(1-thiocyclohexylidene)carbene (singlet 28)

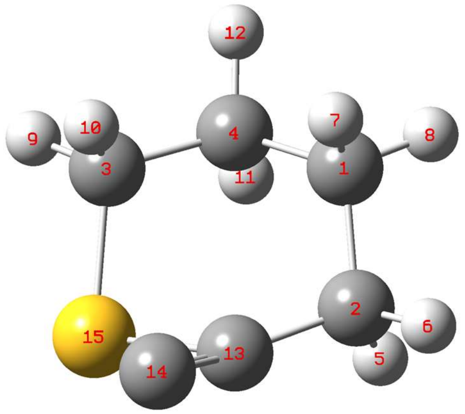

Summary of Natural Population Analysis:

	Natural	Natural Population			

Atom	No	Charge	Core	Valence	Rydberg	Total
C	1	-0.32367	1.99939	4.30563	0.01866	6.32367
C	2	-0.36698	1.99926	4.34180	0.02593	6.36698
C	3	-0.46822	1.99932	4.44607	0.02283	6.46822
C	4	-0.34785	1.99938	4.32889	0.01957	6.34785
H	5	0.19729	0.00000	0.80075	0.00196	0.80271
H	6	0.20545	0.00000	0.79292	0.00164	0.79455
H	7	0.17636	0.00000	0.82139	0.00225	0.82364
H	8	0.18043	0.00000	0.81799	0.00158	0.81957
H	9	0.19681	0.00000	0.80135	0.00183	0.80319
H	10	0.19837	0.00000	0.79880	0.00283	0.80163
H	11	0.17594	0.00000	0.82160	0.00246	0.82406
H	12	0.18321	0.00000	0.81505	0.00174	0.81679
C	13	-0.38364	1.99906	4.33464	0.04994	6.38364
C	14	0.00810	1.99939	3.96606	0.02644	5.99190
S	15	0.36838	9.99914	5.58615	0.04633	15.63162
* Total *		-0.00000	21.99493	37.77908	0.22598	60.00000

1-thiocyclohepty-2-yne (29)

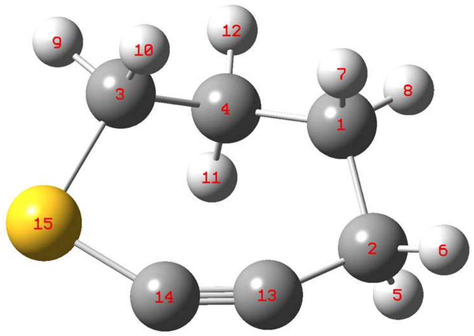

Summary of Natural Population Analysis:

Natural Population

Atom	No	Natural Charge	Core	Valence	Rydberg	Total
C	1	-0.31293	1.99939	4.29456	0.01898	6.31293
C	2	-0.39173	1.99927	4.37242	0.02003	6.39173
C	3	-0.44057	1.99928	4.41893	0.02236	6.44057
C	4	-0.35685	1.99937	4.33750	0.01998	6.35685
H	5	0.19496	0.00000	0.80260	0.00244	0.80504
H	6	0.19870	0.00000	0.79944	0.00186	0.80130
H	7	0.17431	0.00000	0.82356	0.00213	0.82569
H	8	0.17930	0.00000	0.81916	0.00155	0.82070
H	9	0.19074	0.00000	0.80752	0.00174	0.80926
H	10	0.18475	0.00000	0.81244	0.00280	0.81525
H	11	0.18924	0.00000	0.80846	0.00230	0.81076
H	12	0.18401	0.00000	0.81447	0.00152	0.81599
C	13	-0.00363	1.99827	3.98385	0.02150	6.00363
C	14	-0.27353	1.99794	4.25251	0.02307	6.27353
S	15	0.28323	9.99897	5.66365	0.05415	15.71677
* Total *		-0.00000	21.99250	37.81109	0.19641	60.00000

TS from singlet 28 to 29 by C shift

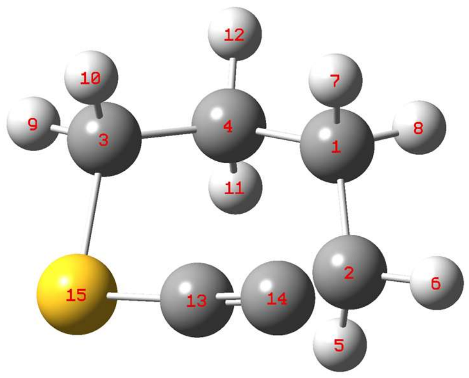

Summary of Natural Population Analysis:

Natural Population

Atom	No	Natural Charge	Core	Valence	Rydberg	Total
C	1	-0.36065	1.99934	4.33660	0.02471	6.36065
C	2	-0.28569	1.99922	4.25767	0.02880	6.28569
C	3	-0.43191	1.99927	4.41036	0.02228	6.43191
C	4	-0.34840	1.99937	4.32504	0.02398	6.34840
H	5	0.19479	0.00000	0.80303	0.00218	0.80521
H	6	0.23908	0.00000	0.75960	0.00133	0.76092
H	7	0.18979	0.00000	0.80832	0.00189	0.81021
H	8	0.18635	0.00000	0.81231	0.00135	0.81365
H	9	0.19667	0.00000	0.80171	0.00162	0.80333
H	10	0.19460	0.00000	0.80269	0.00271	0.80540
H	11	0.17830	0.00000	0.81965	0.00204	0.82170
H	12	0.18519	0.00000	0.81340	0.00141	0.81481
C	13	-0.39221	1.99870	4.35172	0.04180	6.39221
C	14	-0.06009	1.99920	4.03062	0.03027	6.06009
S	15	0.31419	9.99885	5.64031	0.04666	15.68581
* Total *		-0.00000	21.99394	37.77303	0.23303	60.00000

TS from singlet 28 to 29 by S shift

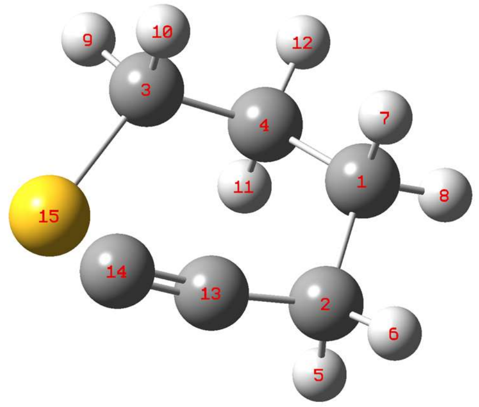

Summary of Natural Population Analysis:

Atom	No	Natural Charge	Natural Population Core	Valence	Rydberg	Total
C	1	-0.31756	1.99938	4.29973	0.01844	6.31756
C	2	-0.43862	1.99918	4.41387	0.02558	6.43862
C	3	-0.46562	1.99929	4.44196	0.02437	6.46562
C	4	-0.35107	1.99937	4.33149	0.02021	6.35107
H	5	0.21025	0.00000	0.78733	0.00242	0.78975
H	6	0.23294	0.00000	0.76556	0.00151	0.76706
H	7	0.17735	0.00000	0.82045	0.00219	0.82265
H	8	0.18233	0.00000	0.81610	0.00157	0.81767
H	9	0.19951	0.00000	0.79877	0.00173	0.80049
H	10	0.19454	0.00000	0.80258	0.00288	0.80546
H	11	0.17853	0.00000	0.81890	0.00257	0.82147
H	12	0.18246	0.00000	0.81574	0.00180	0.81754
C	13	0.03163	1.99902	3.94063	0.02872	5.96837
C	14	-0.29280	1.99872	4.24988	0.04420	6.29280
S	15	0.27614	9.99928	5.67676	0.04782	15.72386
* Total *		0.00000	21.99423	37.77977	0.22600	60.00000
